# Global gene-expression profiles of intracellular survival of the BruAb2_1031 gene mutated *Brucella abortus* in professional phagocytes, RAW 264.7 cells

**DOI:** 10.1186/s12866-018-1223-7

**Published:** 2018-07-31

**Authors:** Myunghwan Jung, Soojin Shim, Young Bin Im, Woo Bin Park, Han Sang Yoo

**Affiliations:** 1 Department of Infectious Diseases, College of Veterinary Medicine, Seoul National University, Seoul, Republic of Korea; 2 Institute of Green Bio Science and Technology, Seoul National University, Pyeongchang, Republic of Korea; 3 Present address: Department of Microbiology, Research Institute of Life Sciences, Gyeongsang National University School of Medicine, Jinju, 52727 Republic of Korea

**Keywords:** BruAb2_1031 gene, Intracellular survival, Defective mutant, *Brucella abortus*

## Abstract

**Background:**

Since recognizing the interaction between *Brucella* and host cells is crucial to the elucidation of the infectious process, *Brucella* researches have prioritized the investigation of genes related to pathogenicity. To demonstrate the roles of *Brucella* genes, RAW 264.7 cells were infected with the *Brucella abortus* wild-type and mutant strains (generated using transposon mutagenesis), after which the different transcriptional responses of the infected cells were determined using microarray.

**Results:**

Following infection, enhanced strategies for intracellular survival, such as down-regulation of genes associated with cytokine responses and apoptosis, were observed in RAW 264.7 cells infected with C3 mutant strain when compared to the transcriptional responses of wild-type infected cells. Using sequence analysis, we determined the mutation site of a C3 mutant strain as the ATP-binding cassette transporter permease (BruAb2_1031). These results were evidenced by an increased level of intracellular survival of the C3 mutant strain.

**Conclusions:**

Characteristics of each mutant strain including bacterial growth rate, abilities to induce cytokine production in macrophages after infection, internalization, and levels of intracellular survival and replication, were investigated by performing RAW 264.7 cell infection experiments. Our results indicate that the BruAb2_1031 gene might be closely related with intracellular survival of *B. abortus* in RAW 264.7 cells.

**Electronic supplementary material:**

The online version of this article (10.1186/s12866-018-1223-7) contains supplementary material, which is available to authorized users.

## Background

*Brucella abortus* (*B. abortus*), a member of the *Alphaproteobacteria* family, is a facultative intracellular bacteria that causes undulant fever, arthritis, endocarditis, and osteomyelitis in humans and abortion and infertility in cattle [1]. Unlike other bacterial pathogens, *Brucella* do not produce classical virulence factors such as exotoxins, cytolysins, capsules, fimbria, plasmids, lysogenic phage, and endotoxic lipopolysaccharide (LPS) molecules [1–3]. Rather, they invade and replicate in professional and non-professional phagocytic cells, thereby eluding the bactericidal immune responses of the host [4].

As facultative intracellular bacteria, *Brucella* can survive and replicate within host macrophages [1, 5]. To establish successful strategies for intracellular survival, *Brucella* often inhibit the normal functions of the host [6–8]. Indeed, *Brucella* utilizes macrophages for infection through mechanisms such as inhibition of apoptosis [6], modification of membrane-bound vesicles [7], and interruption of phagosome-lysosome formation [8]. Hence, identifying the interactions between *Brucella* and macrophages is a key role in understanding its pathogenesis [9, 10]. However, the pathogenic mechanisms of *Brucella* based on these interactions are not well understood.

In *Brucella* infection, macrophages play a central role as the first line of defense of the immune system and the primary target of the pathogen [11, 12]. *Brucella* infected macrophages are activated to promote killing of the bacteria by induction of superoxide anion and hydrogen peroxide [13]. Moreover, the infected macrophages produce pro-inflammatory cytokines (TNF-α, IL-6, and IL-12) and chemokines (GRO- α, IL-8, MCP-1, RANTES, and MIP1 α/β) as critical coordinators of adaptive immunity [5, 14]. Among these biological mediators, TNF-α strongly enhances the bactericidal activity of phagocytes, while IL-12 induces IFN-γ, the major cytokine fighting *Brucella* infection [5, 11]. Along with the cytokine activities, macrophages further respond to *Brucella* infection by inducing apoptosis, thereby exposing the bacterium to the extracellular environment and reducing its replication [6, 14, 15]. Reports indicate that these protective immune responses against *Brucella* are probably associated with several *Brucella* genes which are involved in components or functions of the bacteria, such as lipopolysaccharides, outer membrane proteins, heat shock proteins, ABC-type transporters, and Cu-Zn superoxide dismutase [16, 17]. However, *Brucella* genes associated with the host immune responses and bacterial survival need to be determined more clearly to control brucellosis based on its pathogenesis.

In this study, transcriptional responses of the mouse macrophage cell line (RAW 264.7) infected with *B. abortus* mutant strains were analyzed to demonstrate the role of *Brucella* genes. *B. abortus* mutant strains were previously generated using transposon mutagenesis [18], which is frequently used as a genetic tool to characterize genes of unknown function [19]. Through PCR and alignment analysis, the mutated genes of select mutant strains were revealed as ATP-binding cassette (ABC) transporter permease (BruAb2_1031), ABC transporter substrate-binding protein (BruAb2_0113), and alkyl hydroperoxide reductase D (*ahpD*). As reported previously, BruAb2_1031 and BruAb2_0113 have a role of importing peptides and iron into the bacteria as the ABC transporter system [20]. The *ahpD* is a peroxiredoxin reductase that restores the enzymatic activity of *ahpC*, thereby having an important role as an antioxidants [21]. However, the functional role of these genes is unclear in brucellosis. The transcriptional responses were determined by the microarray approach, which allows understanding of global cell responses. The roles of *Brucella* genes affecting the interactions between host immune cells and the bacteria have been discussed based on differences in the transcriptional responses between macrophages infected with the *B. abortus* mutant strains and the *B. abortus* wild-type.

## Results

### Characteristics of *B. abortus* mutant strains

Among the generated mutant strains, different characteristics were observed in the bacterial growth rates and product levels of NO, IL-6, and TNF-α in RAW 264.7 cells responding to infections of C3, C24 and C30 strains (Additional file 1: Table S1, Additional file 2: Figure S1, and Table 1). The mutant strains were divided into three groups according to the growth rate compared to the wild-type (Additional file 1: Table S1). The C3 mutant strain was selected in group A (mutants showing more than 10% reduction in growth rate) since cells infected by this strain showed lower product levels of NO, IL-6, and TNF-α as compared to others strains in the group. However, RAW 264.7 cells infected with strain C24 in group B (mutants showing similar growth rate) and strain C30 in group C (mutants showing more than 10% increment in growth rate) induced higher levels of NO and TNF-α, and lower levels of IL-6, as compared to other strains in their respective groups. NO and TNF-α are mainly associated with inflammatory reactions, but IL-6 contributes to both pro- and anti-inflammatory responses; hence, the mutant strains C24 and C30 were selected for further studies due to their unique characteristics as compared to other mutants in their group.

**Table 1:** Different characteristics of RAW 264.7 cells infected with mutant strains, as compared to wild-type infected cells

Strains	Nitric oxide (μg/mL)	IL-6 (pg/mL)	TNF-α (pg/mL)	Growth rates (%)
Wild-type	0.57 ± 0.10	3.61 ± 2.64	1219.58 ± 38.91	
C3	0.24 ± 0.03**	N.D.^1)^	1183.69 ± 81.12	87.7 ± 27.9
C24	22.62 ± 0.11**	N.D.	1439.00 ± 71.73**	104.0 ± 1.1
C30	38.81 ± 0.22**	41.64 ± 8.72**	1334.62 ± 43.72*	111.4 ± 16.1

The growth rates are presented as the relative percentage compared to that of the wild-type, when growth rate of wild-type is considered as 100%. N.D.^1)^, non-detected, the product levels of IL-6 in RAW 264.7 cells infected with C3 and C24 mutant strains were close to or below detectable levels of the ELISA system. (*p < 0.05 and **p < 0.01)

**Fig. 1:**
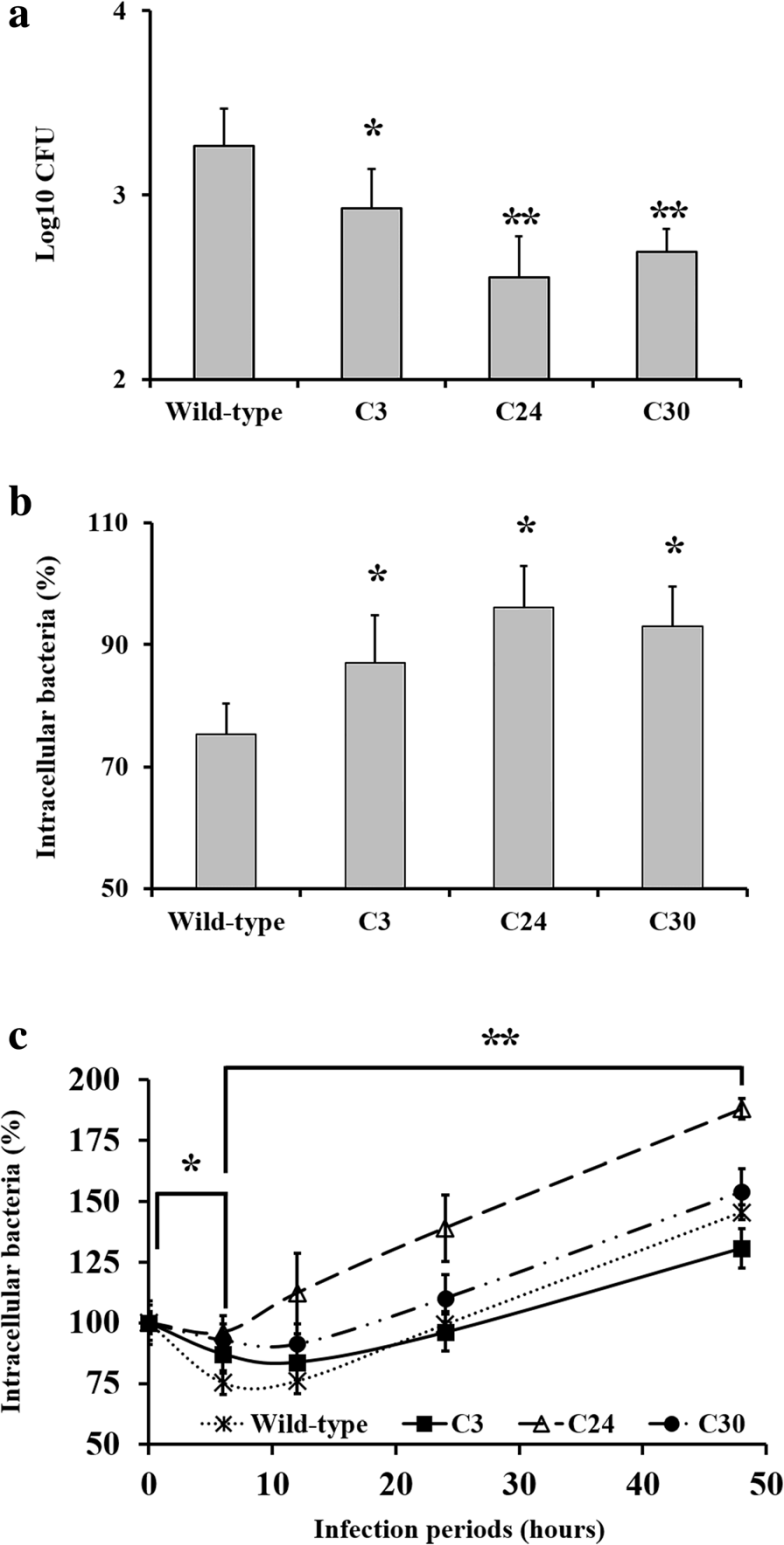
The levels of intracellular *B. abortus* wild-type and mutant strains in infected RAW 264.7 cells. RAW 264.7 cells were infected with wild-type and each mutant strain for 1 h at MOI 100, after which a gentamicin protection assay was conducted. At selected time points, the medium was removed and cells were washed prior to lysis, after which the lysate was plated on to brucella agar. **a** Internalization levels (0 h) of *B. abortus* wild-type and mutant strains in RAW 264.7 cells after gentamicin protection assay. **b** Intracellular CFU level at 6 h after internalization, indicating the levels of intracellular survival. **c** Intracellular CFU level of each strain at selected time points after internalization, indicating the levels of intracellular survival (6 h) and replication (12 h, 24 h, and 48 h) after internalization in RAW 264.7 cells. The CFU levels of (**b**) and (**c**) were expressed as a percentage compared to the respective CFU of internalization of each strain. (*p < 0.05 and **p < 0.01)

The levels of internalization, intracellular survival after internalization, and intracellular replication of each *Brucella* strain in RAW 264.7 cells were investigated (Fig. 1 and Additional file 3: Figure S2). Although the highest internalization level was observed in *B. abortus* wild-type (Fig. 1a, *p* < 0.05 and *p* < 0.01), the decrease was steeper than that observed in of the mutant strains, between 0 h and 6 h (Fig. 1b and c, *p* < 0.05). This steep decrease demonstrated that the wild-type has a lower level of intracellular survival as compared to the mutant strains. According to the CFU slope level increase between 12 h and 48 h, lower levels of intracellular replication were observed in the C3 and C30 mutant strains as compared to the wild-type (Fig. 1c, *p* < 0.01).

### Identification of the transposon insertion site in *B. abortus* mutant strains

The insertion sequence of *B. abortus* mutant C3 was located at the 1,033,916 bp - 1,034,502 bp of chromosome II, *B. abortus* mutant C24 was at the 113,672 bp - 114,206 bp, and *B. abortus* mutant C30 was at the 529,584 bp - 530,364 bp region. From sequencing results, ABC transporter permease (BruAb2_1031), ABC transporter substrate-binding protein (BruAb2_0113) and alkyl hydroperoxide reductase D (*ahpD*) were identified as the genes disrupted by transposon insertion in mutant strains C3, C24, and C30, respectively.

### Determination of differentially expressed genes of infected RAW 264.7 cells

Following infection of *B. abortus* wild-type for 6 h, 12 h, and 24 h, we observed more than 2-fold change in the expression of 58, 202, and 1766 genes, respectively, as compared to uninfected cells (Additional file 4: Table S2). The mutant strain infected RAW 264.7 cells also showed different gene expression levels when compared to uninfected cells (Additional file 5: Figure S3 and Additional file 6: Figure S4). The change in expression levels at 6 h, 12 h, and 24 h, respectively, were observed in: 48, 157, and 1239 genes in C3 mutant strain infected cells; 25, 164, and 1422 genes in C24 mutant strain infected cells; and 58, 202, and 1766 genes in C30 mutant strain infected cells***.***

**Fig. 2:**
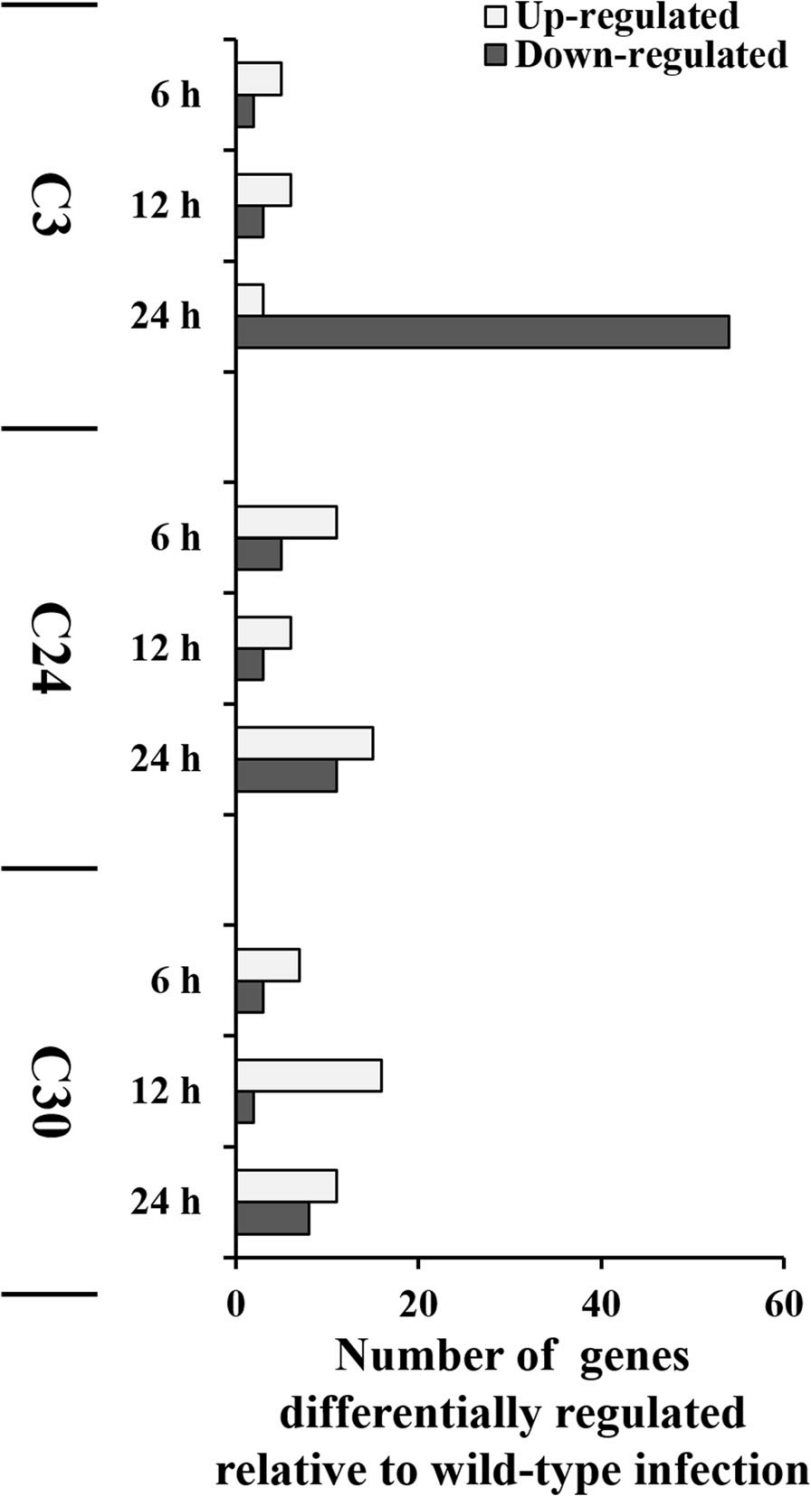
The number of genes showing altered expression in RAW 264.7 cells after mutant strain infection. The gene expressions in *B. abortus* mutant strains of C3, 24, and C30 were compared to each time point of wild-type infection

In comparison to the wild-type infected RAW 264.7 cells, the C3 mutant strain infected cells showed 7, 9, and 57 altered genes at 6 h, 12 h, and 24 h, respectively (Fig. 2 and Additional file 7: Figure S5), whereas C24 and C30 infections showed number of altered genes less than 20 at all-time points (Fig. 2 and Additional file 7: Figure S5). Additional file 7: Figure S5 shows the median of normalized hybridization signals of genes with altered transcription comparing the wild-type and mutant strain infected RAW 264.7 cells at each time point. When compared to wild-type infected RAW 264.7 cells, all genes showing altered expression level of > |2| in *B. abortus* mutant strains infected cells are presented in Additional file 8 Table S3, Additional file 9 Table S4, and Additional file 10 Table S5.

### Gene set enrichment analysis of infected RAW 264.7 cells

**Fig. 3:**
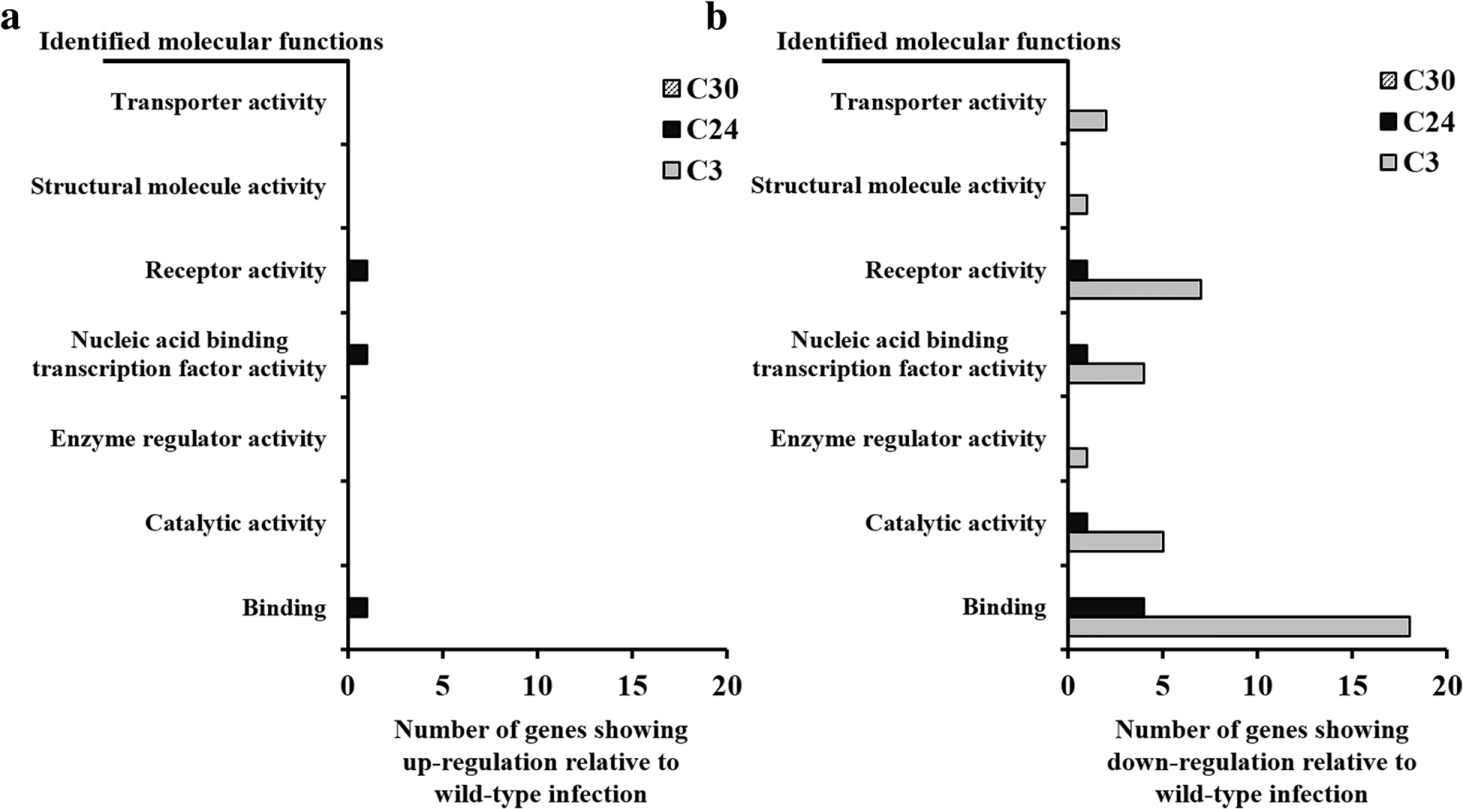
Categorization by molecular function of genes, showing different expression levels between *B. abortus* infections. The different expression levels in *B. abortus* mutant strain infected RAW 264.7 cells were compared to the wild-type infected cells at 24 h after infection. **a** Up-regulated genes. **b** Down regulated genes

**Fig. 4:**
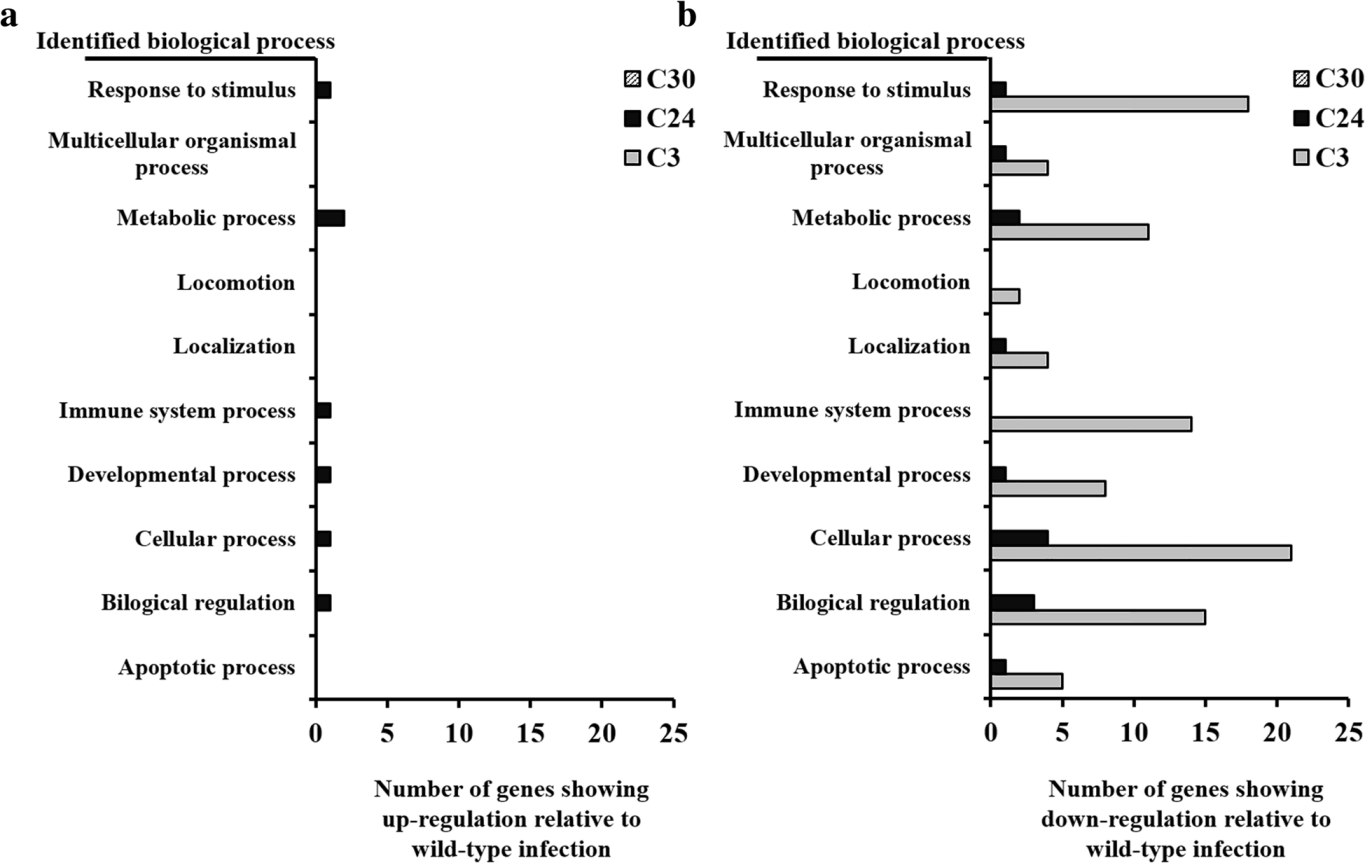
Categorization by biological process of genes, showing different expression levels between *B. abortus* infections. The different expression levels in *B. abortus* mutant strain infected RAW 264.7 cells were compared to wild-type infected cells at 24 h after infection. **a** Up-regulated genes. **b** Down regulated genes

The genes showing altered expression were categorized by gene set enrichment analysis using the Protein Analysis Through Evolutionary Relationships (PANTHER) classification database, to demonstrate coordinated changes in pre-specified sets of related genes. Genes showing different regulation in mutant-infected RAW 264.7cells compared to wild-type infected cells were categorized by molecular functions and biological processes, as shown in Figs. 3 and 4, respectively. Most altered genes in C3 mutant strain infected cells for 24 h were down-regulated, compared to the wild-type infections. These regulated genes were mostly involved in the molecular functions of binding (Fig. 3b) and biological processes of cellular process, response to stimulus, and biological regulation (Fig. 4b). Also, most biological processes of cells were down-regulated in C3 mutant-infected RAW 264.7cells, while only few genes were-upregulated in the C24 mutant-infected cells (Fig. 4a). In case of C24 and C30 mutant strain infected cells, no significant results for pre-specified sets in the PANTHER classification system were observed.

The altered gene expressions in wild-type-infected RAW 264.7 cells compared to uninfected cells were also categorized by the PANTHER classification database. Following *B. abortus* wild-type infection, most of the genes showing different expression were up-regulated at 6 h post infection. Gene enrichment analysis revealed that these genes were associated with the immune system and cell defense processes (*Ccl2*, *Gbp7*, *H2-T24*, *Ier3*, Irg1, *Irf7*, *Kdm6b, Ltb*, *Nfkbia*, *Nfkbiz, Oas2*, *Oasl2*, Ptgs2, *Slfn2*, *Tnf*, *Trim30a*). Among these genes, *Casp4*, *Ier3*, *Ifi204*, *Ltb*, *Nfkbia*, *Tnf*, *Xaf1*, and *Zc3h12a* were also categorized to be associated with apoptosis. At 12 h post infection, RAW 264.7 cells showed down-regulation in 30 genes and up-regulation in 172 genes. The nucleosome core and DNA binding were found to be impacted by the down-regulated genes (*Hist1h2ag*, *Hist1h2bj*, *Hist1h3g*, *Hist1h3c2*, *Serpinb1a*, and *Serpinb9b*). Of the 172 genes up-regulated by *Brucella* infection, the 10 most up-regulated genes were associated with immune response (*Irg1*, *Ifi44l*, *Ifit1*, *Ifi204*, *Oasl2*, *Ifi202b*, *H2-T24*, *Irf7*, *Cmpk2*, and *Usp18*). In addition, genes associated with apoptosis were also up-regulated (*Bcl2a1d*, *Cd40*, *Hck*, *Ltb*, *Nfkbia*, *Pim1*, *Rassf4*, *Stat1*, *Stat2*, *Tnf*, *Tnfrsf1b*, *Tnfrsf9*). Maximum altered gene expression was observed after 24 h infection (Additional file 5: Figure S3 and Additional file 6: Figure S4). Genes showing altered expression were associated with 10 molecular functions and 14 biological processes. Most of the differentially expressed genes were involved in two molecular function categories (catalytic activity and binding) and two biological process categories (metabolic process and cellular process).

### Analyses of affected pathways and gene networks following mutant strain infections

**Fig. 5:**
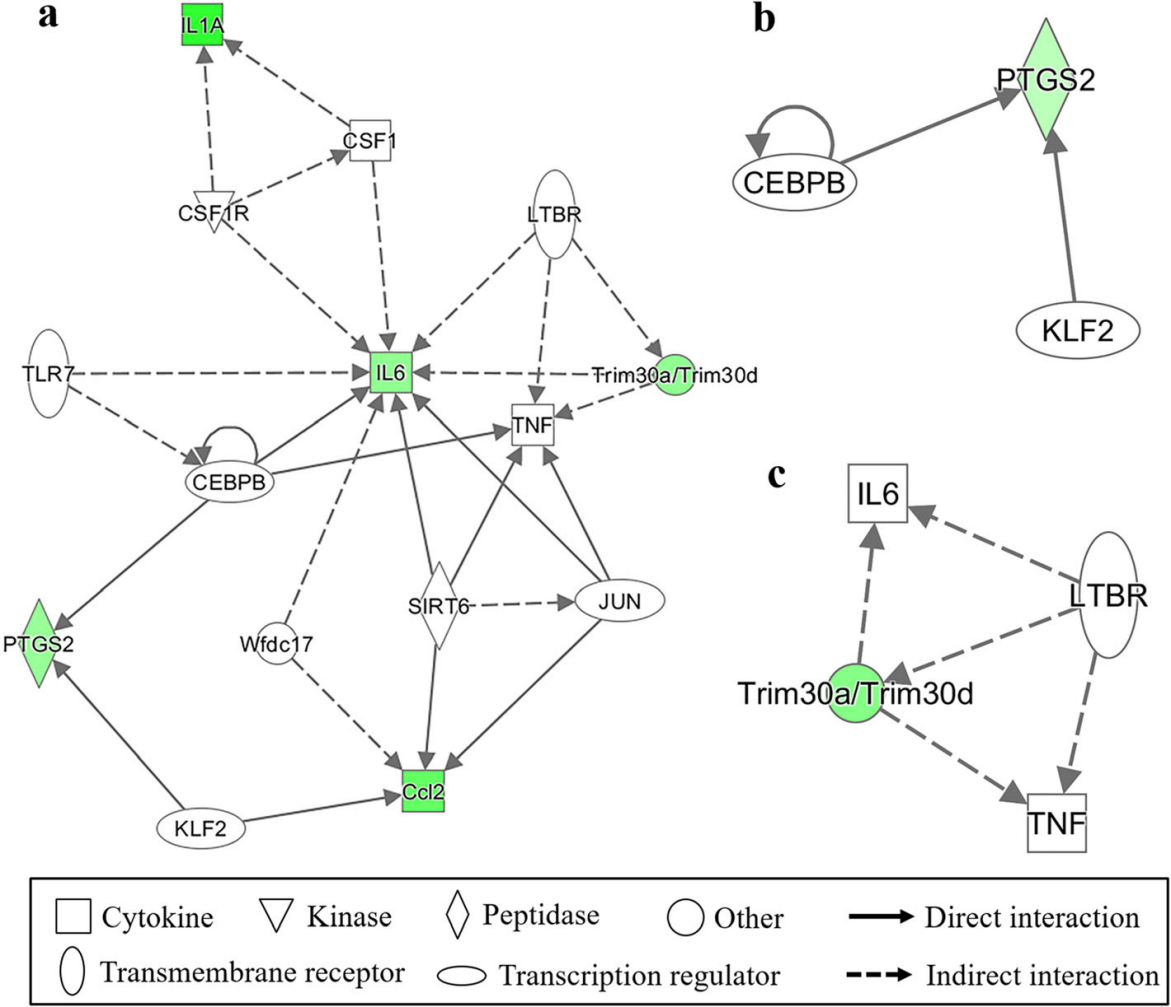
Identified network of the genes with altered expression in mutant strain infected RAW 264.7 cells. The different gene expression levels in *B. abortus* mutant strain infected RAW 264.7 cells at 24 h after infection were compared to the wild-type infected cells. Green color indicates down-regulation and arrows indicate directional relationships. **a** C3. **b** C24. **c** C30

Pathway mapping using the Kyoto Encyclopedia of Genes and Genomes (KEGG) database revealed that 24 h *B. abortus* infection led to cell cycle arrest. When compared to the wild type infected cells, no KEGG pathway was represented by more than 10 genes in the C24 and C30 mutant strain infected RAW 264.7 cells. Conversely, the cytokine-cytokine receptor interaction pathway was demonstrated by 11 down-regulated genes (*Ccl2*, *Ccl5*, *Ccl7*, *Csf2*, *Csf3*, *Cxcl10*, *Cxcl11*, *Il1a*, *Il1b*, *Il6*, and *Tnfrsf1*) in C3 mutant infected RAW 264.7 cells after 24 h. The TNF-like receptor pathway, chemokine-signal pathway, and Toll-like receptor pathway were found to be affected by altered genes in C3 mutant strain infected cells at 24 h. Among the differentially expressed genes in C3, C24, or C30 infected cells, one network was identified by the Ingenuity Pathway Analysis (IPA) in each mutant strain at 24 h after infection, as shown in Fig. 5. The network identified in C3 mutant strain infected cells was mainly associated with cytokine interactions among IL-6, IL-1A, and CCL2. In addition, gene networks of C24 and C30 mutant strains were on the prostaglandin-endoperoxide synthase 2 and tripartite-motif protein 30, respectively.

### Validation of microarray data

**Fig. 6:**
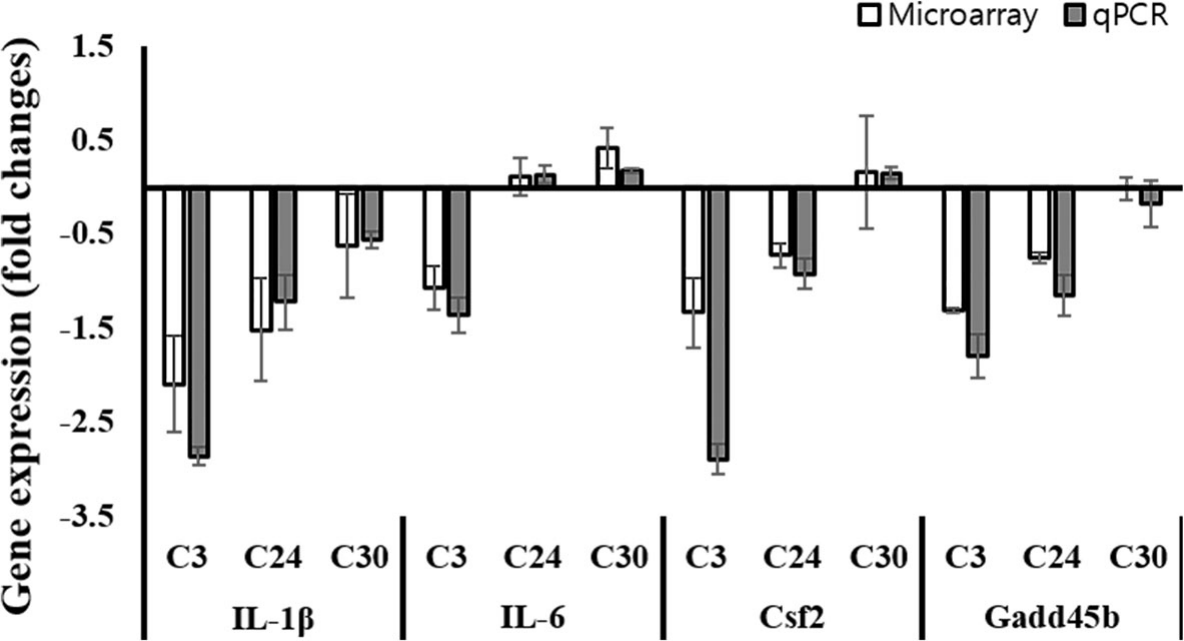
Validation of microarray data via quantitative RT-PCR. Relative expression level was determined by the 2^-∆∆Ct^ method with normalization to the housekeeping gene, β-actin

To verify the microarray results, gene expression levels of *IL-1β*, *IL-6*, *Csf2*, and *Gadd45b* in the microarray RNA samples were investigated by quantitative real time RT-PCR (qRT-PCR). The C3 mutant strain infected RAW 264.7 cells showed significant changes in gene expression level compared to wild-type infected cells. Validation was therefore performed based on these genes showing different expression levels. Furthermore, the microarray results revealed that pathways of “Responses of cytokine and immune defense”, and “Apoptosis process” were down-regulated in C3 mutant strain infected cells compared to wild-type infections. Hence, qRT-PCR for microarray validation was carried out using genes associated with these pathways (*IL-1β*, *IL-6*, *Csf2*, and *Gadd45b*). As shown Fig. 6, all genes evaluated by qRT-PCR showed similar expression levels as the microarray results.

## Discussion

*Brucella* spp. are facultative intracellular bacteria like *Salmonella enterica* and *Listeria monocytogenes*. However, they express uncanonical virulence factors such as virulence regulator proteins and phosphatidylcholine, rather than classic virulence factors such as protease, exotoxin, cytolysins, and capules [1–3]. Moreover, *Brucella* spp. utilize and modulate normal functions of host cells, such as macrophages, to establish successful strategies for intracellular survival [5, 14, 15]. Therefore, characterization of the host macrophage-*Brucella* interaction is very important to identify the pathogenicity and infection mechanism of *Brucella* [22]. Microarray is a powerful approach that enables understanding the host responses at the global gene transcription level, thereby providing a plethora of information regarding the interactions associated with cell responses to antigens at the molecular level [15, 23]. In this study, microarray analysis was used to compare the responses between macrophages infected with *B. abortus* wild-type and *B. abortus* mutant strains, to demonstrate the *Brucella* genes that affect the interactions between host immune cells and the bacteria.

Previous studies of genes associated with pathogenicity of bacteria have been conducted using random insertion mutants generated by transposable elements [18, 19, 23, 24]. *B. abortus* mutant strains used in the current study were generated using transposon mutagenesis and single insertions certified in our previous study [18, 23, 25], in which *Brucella* genes associated with internalization and early host immune responses were investigated using 2 h infection experiments. In this study, we focused on the genes affecting interactions between host immune cells and *Brucella*, which would affect the survival strategy of the bacterium through longer infection period. Infection periods longer than 12 h are sufficient for *Brucella* to form replicative *Brucella*-containing vacuoles [26]. Among the mutant strains generated in our previous study [18], C3, C24, and C30 strains, which showed unique characteristics compared to the wild-type (Additional file 2: Figure S1 and Table 1), were selected for this study. The selection was carried out based on the growth rate and differences in responses of RAW 264.7 cells to infection with wild-type and mutant strains. The product levels of IL-6, TNF-α, and NO in infected cells, which are representative biomaterials associated with bactericidal responses of macrophage to bacterial infection [27–29], were used as criteria for identifying differences in responses of infected cells.

Macrophages have been reported to show early immune responses (such as inflammation) following *B. abortus* infection [6, 14, 15, 23, 30, 31]. Inflammation is a protective response to *B. abortus* infection observed in the early stages of infection, and coordinated by cytokines and chemokines [15, 30]. In this study, wild-type infected RAW 264.7 cells showed up-regulation in genes associated with immune responses (*Ccl2*, *Gbp7*, *H2-T24*, *Ier3*, Irg1, *Irf7*, *Kdm6b, Ltb*, *Nfkbia*, *Nfkbiz, Oas2*, *Oasl2*, Ptgs2, *Slfn2*, *Tnf*, *Trim30a*). Of these, *Casp4*, *Ier3*, *Ifi204*, *Ltb*, *Nfkbia*, *Tnf*, *Xaf1*, and *Zc3h12a* were also categorized as being associated with apoptosis, which plays a major role in responses to brucellosis by exposing the bacterium to the extracellular environments, where it encounters immune system components such as antibodies and the complement system, thereby reducing bacterial replication [6, 14, 15, 31]. In this study, most responses of RAW 264.7 cells at 6 h were focused on defense activities of macrophages. These results corresponded to the intracellular survival and replication after internalization in RAW 264.7 cells, demonstrated by a decrease in intracellular *B. abortus* wild-type at 6 h after internalization (Fig. 1b).

Following 12 h of infection, the nucleosome core and DNA binding were found to be down-regulated (*Hist1h2ag*, *Hist1h2bj*, *Hist1h3g*, *Hist1h3c2*, *Serpinb1a*, and *Serpinb9b*). Considering nucleosomes are selective products during apoptosis [32, 33], it is assumed that the apoptotic process of macrophages was affected by down-regulated genes due to *Brucella* infection. Interestingly, the most up-regulated genes were involved in the immune responses (*Irg1*, *Ifi44l*, *Ifit1*, *Ifi204*, *Oasl2*, *Ifi202b*, *H2-T24*, *Irf7*, *Cmpk2*, and *Usp18*), as were the genes associated with apoptosis (*Bcl2a1d*, *Cd40*, *Hck*, *Ltb*, *Nfkbia*, *Pim1*, *Rassf4*, *Stat1*, *Stat2*, *Tnf*, *Tnfrsf1b*, *Tnfrsf9*). These results indicate that the professional macrophages, namely the RAW 264.7 cells, elicited immune responses to clear *Brucella* infection consistently, concurrent with the activities of *Brucella* to elude the clearance efforts of the host [6, 14, 15, 23, 31, 34].

KEGG pathway mapping showed that *B. abortus* wild-type infected cells were down-regulated in cell cycle through down-regulation of pathways such as cell cycle (42 genes), purine metabolism (26 genes), pyrimidine metabolism (24 genes), and DNA replication (23 genes) at 24 h [35]. The p53 pathway, which inhibits cell proliferation and then induces apoptosis [36, 37], was also down-regulated. Those down-regulations were related with inhibition of cell death, which allows the survival of brucellae by avoiding exposure to the more hostile extracellular environment, thereby inducing more replication of *B. abortus* [31]. Moreover, it was observed that genes involved in phagosome formation were down-regulated (*Scarb1*, *Tuba1a*, *Tuba4a*, *Tuba1c*, *Tubb5*, *Tubb4b*, and *Tubb6*). *Brucella* is known to inhibit phagosome formation and phagosome-lysosome fusion to avoid host immune responses [9, 14, 38, 39]. In addition to these strategies, modification of cell cycle arrest, which may permit inhibition of apoptosis [15, 22], seemed to be more dominant than other down-regulated factors such as cytokine interaction and phagolysosome formation, considering the number of genes involved in cell arrest. Conversely, immune responses of RAW 264.7 cells to *B. abortus* infection for 24 h were focused on the cytokine interactions, as described in previous studies [11, 15, 31]. The up-regulation of the KEGG pathway of TNF-signaling and cytokine-cytokine receptor interaction was observed in this study. Moreover, these pathways involved genes that were differentially expressed by more than 20-fold, such as *Ccf1*, *Il6*, and *Ccl5*.

Although the overall transcriptional responses were similar between mutant strains and wild-type of *B. abortus*, notable difference were observed at the different time points. The 54 down-regulated genes in C3 mutants at 24 h were mainly involved in cell immune responses associated with cytokine-cytokine receptor interaction in the KEGG pathway database, which are mainly involved in immune responses elicited by RAW 264.7 cells to clear *Brucella* [4, 14, 15, 23]. Moreover, these genes were involved in the apoptosis process, which plays an important role in intracellular survival of *Brucella* [4, 15, 40]. The gene network analysis demonstrated that IL-1α, IL-6, tripartite-motif protein 30 (Trim30), prostaglandin-endoperoxide synthase 2 (PTGS2; also known as cyclooxygenase-2, COX-2), and CCL2 were down-regulated, showing interaction. These genes mediate the macrophage polarization to M1, a common response of macrophages to bacterial infections, which includes genes encoding TNF, IL-1β, IL-6, and CCL2 [41]. IL-6 plays a central role in the response of regulatory T cells to microbial infections, and is known to be a predominant mediator of the acute phase response in inflammation triggered by infection [42]. IL-6 synthesis is induced by COX-2 activation [42]. This positive relationship was found in the network of RAW 264.7 cells infected with C3 mutant strain. Several studies have proved the important role of IL-6 in *Brucella* infection. Indeed, IL-6 is known to play a role in the induction of acquired cellular resistance to intracellular bacteria by macrophage activation [43–45]. Important roles of IL-6 were also reported in the regulation of MHC II expression and antigen processing [45, 46]. Previous study further demonstrated that IL-6 inhibited intracellular replication through its control effects on endocytosis and endosome-phagosome fusion [47]. IL-1 has been reported to play an important role in protective responses against *Brucella* infection [48]. Trim30 regulates the NF-κB pathway as a negative feedback via the degradation of TAB2 and TAB3, resulting in inhibition of TRAF6 ubiquitylation, leading to the inhibition of NF-κB activity [49, 50]. Activation of the NF-κB pathway results in the induction of Type I IFN and other pro-inflammatory cytokines, thereby inducing protective effects against infection [51]. Down-regulation of *Trim30d* expression was presumed to be caused by lower NF-κB activity in C3 mutant strain infected cells. This down-regulation of genes associated with the protective immune responses against *Brucella* infection was confirmed by the quantification of NO, IL-6 and TNF-α in C3 mutant strain infected RAW 264.7 cells. In summary, the C3 mutant strain had the ability to inhibit the protective immune responses, via cytokine production.

The load of intracellular bacteria has differing effects on the gene expression in infected RAW 264.7 cells. However, there was no significant difference in the gene expression in the 12 h microarray analysis, between cells infected with wild-type and C24 mutant strains, although the RAW 264.7 cells were infected with higher CFU number of C24 mutants than wild-type strain (Fig. 1). In case of 24 h microarray analysis, even though differences in intracellular bacteria CFU number were observed among the strains of wild-type, C24, and C30 mutants, there was no significant difference of gene expression level in the infected RAW 264.7 cells compared to the wild-type infected cells. These results suggest that different gene expressions observed in the RAW 264.7 cells infected with C3 mutant compared to the cells infected with the wild-type were not significantly affected by the intracellular bacterial CFU load.

The ABC transporter permease (BruAb2_1031) has a role in dipeptide import [20]. Insertion of transposon into BruAb2_1031 was confirmed in the C3 mutant strain by BLASTIN analysis. The disruptive effects of BruAb2_1031 on the pathogenesis of *B. abortus* are not clearly understood. However, considering the important roles of peptide uptake in bacterial nutrition associated with growth and replication, especially in intracellular bacteria, it is plausible that mutation at BruAb2_1031 might affect the growth and virulence of *B. abortus* [52, 53]. These agreed with our results which showed slow growth and lower replication levels of C3 mutant in brucella broth and RAW 264.7 cells, respectively. Although the exact roles of the gene in *B. abortus* infection are not clear, this result implicated that mutation at BruAb2_1031 causes the inhibition in uptake of nutrition, thereby inducing the decrease of growth and intracellular replication.

Along with the effect on bacterial nutrition, disruption of ABC transporter affects the internalization and intracellular survival. Previous studies demonstrated that the mutation at genes associated with the transporter system (such as *VirB*, *cgt*, and *cydC*) reduced the internalization and intercellular survival in *B. abortus* [54–56]. These results implicate that disruption of the ABC transporter system generally exerts a negative effect on internalization, and the intracellular survival and replication of *Brucella*. However, a high level of intracellular survival was observed in the C3 mutant strain at 6 h after internalization, in spite of low levels of internalization and intracellular replication (Fig. 1) [6, 14, 15, 23, 30, 31]. This result was thought to be induced by reduced immune responses of RAW 264.7 cells to C3 mutant strain infection, which were agreeable with the results of microarray.

ABC permease utilizes the periplasmic binding protein (PBPs) to capture substrate and present it at the intake vestibules of the membrane translocator unit [57]. It is therefore presumed that the mutation at the BruAb2_1031 gene could induce changes in constituents of the bacterial envelope, even though they were small. This perturbation in bacterial envelope was speculated to alter the antigens interacting with macrophages, thereby reducing the immune responses of macrophage to infection of C3 mutant strain in this study [14, 31, 58]. This phenomenon was also supported by microarray analysis where down-regulation of the gene expression in C3 mutant infected RAW 264.7 cells was observed, especially in genes associated with M1 polarization of macrophages [58].

In the C24 mutant strain infected RAW 264.7 cells, most genes altered in expression were classified as predicted genes. The IPA network revealed the low immune response of RAW 264.7 cells to C24 mutant strain infection through down-regulation of COX-2, which is normally up-regulated in response to inflammatory and pro-inflammatory responses [59]. However, no other significant differences were observed in immune responses or apoptosis between RAW 264.7 cells infected with C24 mutant strain and wild-type strain. The C30 mutant infected RAW 264.7 cells also showed slight differences in gene expression compared to the wild-type infected cells. Similar to C24 mutant strain infected RAW 264.7 cells, most of the altered genes in the C30 mutant strain infected cells were predicted genes. There were no notable differences between cells infected with wild type or C30 mutant strain, except for down-regulation of *Trim30*. Therefore, further analyses on these predicted genes are required to demonstrate the role of BruAb2_0113 and *ahpD* in brucellosis.

Altogether, the C3 mutant strain showed high intracellular survival in RAW 264.7 cells compared to the wild-type. These cells also showed down-regulation of genes associated with protective immune responses to *Brucella* infection. Our results suggest that the C3 mutant strain has more enhanced strategies for intracellular survival than the wild-type. This enhanced intracellular survival ability could be presumed to be elicited by the mutation of BruAb2_1031, which is considered to be a cause of antigen alteration that could reduce the immune responses of macrophages in brucellosis.

The BruAb2_1031 gene (ABC transporter permease) is known to play a role in the transporter of peptides as a component of the ABC transporter [20]. However, our study showed BruAb2_1031 mutation is effective against Brucellosis. Our findings suggest that the ABC transporter permease could be a potential antigen in the development of *Brucella* vaccine in the future. In addition, to clarify a role of ABC transporter permease, changes in the function of ABC transporter by mutation of BruAb2_1031 gene need to be further investigated, and effects of BruAb2_1031 gene mutation on *B. abortus* should be identified.

## Conclusions

Characteristics of mutant strains generated by transposon mutagenesis were investigated by infection experiments in RAW 264.7 cells. BLASTIN analysis revealed that the ABC transporter permease (BruAb2_1031) was mutated in the C3 mutant strain, which showed a higher intracellular survival rate in RAW 264.7 cells than the wild strain. This enhancement is presumed to be elicited by the mutation of BruAb2_1031, which is considered as a cause of antigen alteration that could reduce the immune responses of macrophages in brucellosis. Also, down-regulation of the protective immune response-related functions were observed in C3 mutant strain infected RAW 264.7 cells. This study reported for the first time that mutation of the BruAb2_1031 gene in *B. abortus* reduces the defense responses of RAW 264.7 cells to *Brucella* infection, indicating that the molecules associated with BruAb2_1031 gene have a role in immune responses of macrophages, and therefore its mutation enhanced the intracellular survival of *B. abortus*.

## Methods

### Bacterial strains and cell line

In our previous study, 132 mutant strains were generated from *B. abortus* 1119–3 strain (wild-type) [18, 23] using EZ-Tn5™ Transposome complexes (Epicentre® Biotechnologies, USA) [18]. *Brucellae* were cultured in brucella broth or agar (Difco, USA) and *Brucella* mutant strains were cultured with 30 μg/mL of kanamycin. The murine leukemic monocyte macrophage line, RAW 264.7, was obtained from the Korea Cell Line Bank (KCLB No.40071, Korea) and grown at 37 °C in 5% CO_2_ atmosphere in Roswell Park Memorial Institute medium (RPMI) 1640 (Gibco, USA) containing 10% fetal bovine serum (FBS; Gibco), penicillin (100 U/mL), and streptomycin (100 μg/mL). All procedures of the bacterial experiment were approved by the Seoul National University Institutional Biosafety Committee (SNUIBC-R160314–1).

### Growth rates of *B. abortus* mutant strains

The bacterial growth rates were measured in brucella broth without antibiotics, according to the standard curve of CFU versus optical density. The level of bacterial growth rates was presented as the relative percentage compared to that of the wild-type, where the growth rate level of wild-type was regarded as 100%. Growth measurement experiments were independently conducted two times.

### Selection of *B. abortus* mutant strains

Among the generated mutants by same electroporation of transposome, 26 mutant stains showed defective internalization (Additional file 2 Figure S1 and Additional file 1: Table S1). Because transposon mutagenesis could cause defective growth [60], the mutant strains were grouped based on the growth rate, as follows: group A of mutant strains showed more than 10% reduction in growth rate; group B of mutant strains showed similar growth rate compared to that of wild-type; group C of mutant strains showed more than 10% increment in growth rate. Among the mutant strains in each group, strains showing distinctive characteristics were selected for further studies.

The selection of mutant strains was carried out based on the differences in production levels of NO, IL-6, and TNF-α in RAW 264.7 cells responding to infection with each strain. Briefly, RAW 264.7 cells were cultured at a concentration of 8.0 × 10^4^ cells/cm^2^ in 6-well culture plates, in 2 mL of media containing 2% FBS. After 8 h-culture, cells were infected with *B. abortus* wild-type or mutant strains at a multiplicity of infection (MOI) of 100 [18]. Following centrifugation at 150×g for 10 min at room temperature, the infected cells were incubated at 37 °C and 5% CO_2_ for 24 h. The NO production in supernatants of infected cells was measured using Griess reaction, as described previously [61]. The amounts of IL-6 and TNF-α in supernatants were measured using a cytokine ELISA kit (eBioscience, USA) according to the manufacturer’s instructions. All experiments were independently carried out twice.

### Internalization, intracellular survival, and intracellular replication in RAW 264.7 cells

The levels of internalization, intracellular survival, and intracellular replication in RAW 264.7 cells were also investigated. RAW 264.7 cells were infected with *B. abortus* wild-type and mutant strains at an MOI of 100 [6, 18]. After 1 h, the medium was removed and replenished with medium containing 2% FBS and 30 μg/ml of gentamicin (gentamicin protection assay). The cells were then washed prior to lysis, and lysates were plated on to brucella agar at 0 h, 6 h, 12 h, 24 h, and 48 h after gentamicin treatment. The levels of internalization (0 h), intracellular survival (6 h) and replication for each strain (12 h, 24 h, and 48 h) were expressed by CFU. The CFU changes of intracellular bacteria were investigated through three repeated experiments.

### Identification of transposon insertion sites

In our previous study, we had confirmed the single insertion of Tn5 transposome by Southern blot analysis [25]. The sequence of insertion site and disrupted gene in mutant strains were identified using PCR and alignment of search tool, as described previously [18]. DNA sequences from the PCR product were used to identify the insertion sites using analysis *B. abortus* chromosome II with circus (http://circos.ca/guide/genomic/).

### Macrophage infection and RNA preparation for microarray

Macrophage infection was conducted using *B. abortus* wild-type and mutant strains at an MOI 10 [62, 63]. Following centrifugation at 150×g for 10 min at room temperature, the infected cells were incubated at 37 °C under 5% CO_2_ atmosphere for 6 h, 12 h, and 24 h. After three washes with DPBS (Gibco, USA), the RNA was extracted from the infected cells at each time point using an RNeasy mini kit (Qiagen, Netherlands), as described by the manufacturer. The purity and integrity of RNA was determined by denaturing gel electrophoresis, the OD260/280 ratio, and analysis on an Agilent 2100 Bioanalyzer (Agilent Technologies, USA). All RNA samples were quantified, divided into aliquots, and stored at − 80 °C until use.

### Microarray hybridization

RNA amplification, labeling, array hybridization, and scanning were conducted by Macrogen Inc. (Seoul, Korea). The Affymetrix Whole Transcript Expression array process was conducted according to the manufacturer’s protocols using a GeneChip Whole Transcript PLUS reagent kit (Affymetrix, USA). Briefly, the GeneChip Whole Transcript Amplification kit (Affymetrix) was used for cDNA synthesis. The sense cDNA was then fragmented and biotin-labeled with terminal deoxynucleotidyl transferase, using a GeneChip Whole Transcript Terminal labeling kit (Affymetrix). Approximately 5.5 μg of labeled DNA target was hybridized to the Affymetrix GeneChip Mouse 2.0 ST Array for 16 h at 45 °C, which covers more than 35,000 transcripts. After washing step, hybridized arrays were stained on a GeneChip Fluidics Station 450 and scanned on a GCS3000 Scanner (Affymetrix). Signal values were computed using the Affymetrix® GenChip™ Command Console software (AGCC).

### Raw data preparation and statistical analysis

Raw data extracted by AGCC were summarized and normalized by the robust multi-average (RMA) method implemented in Affymetrix® Expression Console™ Software. The results were exported with gene level RMA analysis, and subjected to the differentially expressed gene (DEG) analysis. Statistical significance of the expression data was determined using LPE test and fold change, in which the null hypothesis was that no difference exists among groups. False discovery rate was controlled by adjusting the *p*-value using the Benjamini-Hochberg algorithm. The genes that showed different expression levels in RAW 264.7 cells infected with *B. abortus* mutant strains compared to that of wild-type, were determined based on a *p*-value < 0.05 and a fold-change > |2|. Dataset of microarray results has been deposited in Gene Expression Omnibus (GEO, http://www.ncbi.nlm.nih.gov/geo/query/acc.cgi?acc=GSE79264) and are accessible through GEO series accession number GSE79264.

### Microarray data analysis

Genes showing significantly altered expressions were subjected to gene set enrichment analysis using the PANTHER classification database (http://www.pantherdb.org). Differently expressed genes were categorized by biological processes and molecular functions using the PANTHER classification database based on means of Fisher’s exact test, to identify coordinated changes in pre-defined sets of related genes.

The changes in cell process were derived from interactions among the differently expressed genes, which form the functional pathways and networks [64]. Therefore, genes showing altered expressions were analyzed using the KEGG database to identify systemic information representing functional aspects of each gene. For a pathway mapping term to be considered significant, pathways represented by fewer than 10 genes were filtered out for identification of the most affected pathways [65]. In addition, the Qiagen’s IPA (Ingenuity Systems Inc., USA) was performed for biological processes, canonical pathways, and networks analysis.

### Verification of microarray results

To verify the microarray results, three genes associated with responses of cytokines and immune defense against *Brucella* infection, and one gene connected to the apoptosis process of the cell, were selected and subjected to qRT-PCR (Table 2). Total RNA was reverse transcribed using a QuantiTect Reverse Transcription kit (Qiagen), according to the manufacturer’s protocols. The qRT-PCR reaction was carried out with cDNA synthesized from 24 ng of RNA using the Rotor-Gene SYBR Green PCR kit (Qiagen) and Rotor-Gene Q real time PCR cycler (Qiagen), under the following conditions: 45 cycles at 95 °C for 15 s followed by 45 s at 60 °C [35]. The gene expression levels were analyzed by the 2^-∆∆Ct^ method based on the house-keeping gene, β-actin, as a reference [35, 66].

**Table 2:** Primers used for qRT-PCR

Genes		Primers (5′-3′)	Relevant function in brucellosis	Gene Accession
IL-1β	F	CAACCAACAAGTGATATTCTCCATG	Responses of cytokine and immune defense	NM_008361
	R	GATCCACACTCTCCAGCTGC		
IL-6	F	CTCTGCAAGAGACTTCCATCCA	Responses of cytokine and immune defense	NM_031168
	R	GACAGGTCTGTTGGGAGTGG		
Csf2	F	GAGGATGTGGCTGCAGAATTTAC	Responses of cytokine and immune defense	NM_009969
	R	CTTCTACCTCTTCATTCAACGTGAC		
Gadd45b	F	CTGATGAATGTGGACCCCGA	Apoptosis process	NM_008655
	R	CCTCTGCATGCCTGATACCC		

### Statistics

Data are presented as the mean ± standard deviation (SD). Statistical significance was analyzed by Student’s *t*-test or repeated measures of ANOVA (Tukey’s HSD test for post-test of multiple comparison) using SPSS version 23.0 software (SPSS, USA). The statistical significance of differences was set at value of *p* < 0.05.

## Additional files

Additional file 1: **Table S1.** Growth of *B. abortus* wild-type and mutant strains at each time point. CFU was calculated using the standard curve of CFU versus optical density. (PDF 19 kb)

Additional file 2: **Figure S1.** Characteristics of RAW 264.7 cells infected with *B. abortus* mutant strains. (a) Internalization was investigated using RAW 264.7 cells infected with *B. abortus* wild-type and mutant strains at an MOI of 100. (b), (c), (d) Product levels of NO, IL-6, and TNF-α in RAW 264.7 cells responding to infection with each strain (MOI 100) was measured 24 h after infection. Based on the growth rate, mutant strains in this study were divided into the three groups as follow: group A of mutant strains showed more than 10% reduction in growth rate; group B of mutant strains showed similar growth rate compared to that of wild-type; group C of mutant strains showed more than 10% increment in growth rate. In this study, RNA samples from the RAW 264.7 cells infected with mutant strain (C3, C24, and C30) were subjected to microarray analysis. The product level of IL-6 in RAW 264.7 cells infected with C3 and C24 mutant strains were close to or below detectable levels of the ELISA system. (TIF 939 kb)

Additional file 3: **Figure S2.** The CFU numbers of intracellular *B. abortus* wild-type and mutant strains in RAW 264.7 cells. RAW 264.7 cells were infected with wild-type and each mutant strain for 1 h at MOI 100, after which a gentamicin protection assay was conducted. At the selected time points, the medium was removed and cells were washed prior to lysis; the lysate was then plated on to brucella agar. Intracellular CFU (Log10) numbers of each strain at selected time points after internalization was evaluated, which indicates the levels of intracellular survival (6 h) and replication (12 h, 24 h, and 48 h) at each time point after internalization in RAW 264.7 cells (*p < 0.05 and **p < 0.01). (TIF 503 kb)

Additional file 4: **Table S2.** The genes showing altered expression in RAW 264.7 cells after *B. abortus* infection. The different expression levels in *B. abortus* infected RAW 264.7 cells were compared to uninfected cells. (PDF 1128 kb)

Additional file 5: **Figure S3.** Categorization by molecular function of genes showing different expression levels after infection. The different expression levels in *B. abortus* wild-type and mutant strain infected RAW 264.7 cells were compared to uninfected cells. (a) Up-regulated genes. (b) Down regulated genes. (TIF 922 kb)

Additional file 6: **Figure S4.** Categorization by biological process of genes showing different expression levels after infection. The different expression levels in *B. abortus* wild-type and mutant strain infected RAW 264.7 cells were compared to uninfected cells. (a) Up-regulated genes. (b) Down regulated genes. (TIF 936 kb)

Additional file 7: **Figure S5.** Scatter plots showing different gene expressions. The different gene expression levels in *B. abortus* mutant strain infected RAW 264.7 cells were compared to cells infected with wild-type at 6 h, 12 h, and 24 h after infection. Genes showing different expression levels are indicated by red dots. (TIF 2217 kb)

Additional file 8: **Table S3.** The genes showing altered expression in RAW 264.7 cells after C3 mutant strain infection. The different expression levels in *B. abortus* C3 mutant strain infected RAW 264.7 cells were compared to wild-type infected cells. (PDF 52 kb)

Additional file 9: **Table S4.** The genes showing altered expression in RAW 264.7 cells after C24 mutant strain infection. The different expression levels in *B. abortus* C24 mutant strain infected RAW 264.7 cells were compared to wild-type infected cells. (PDF 41 kb)

Additional file 10: **Table S5.** The genes showing altered expression in RAW 264.7 cells after C30 mutant strain infection. The different expression levels in *B. abortus* C30 mutant strain infected RAW 264.7 cells were compared to wild-type infected cells. (PDF 37 kb)

## Abbreviations

CCL2: Chemokine (C-C motif) ligand 2
CFU: Colony forming unit
COX-2: Cyclooxygenase-2
DEG: Differentially expressed gene
DPBS: Dulbecco’s Phosphate-Buffered Saline
FBS: Fetal bovine serum
GRO-α: Growth regulated oncogene alpha
IFN-γ: Interferon-gamma
IL-12: Interleukin-12
IL-6: Interleukin-6
IL-8: Interleukin-8
IPA: Ingenuity Pathway Analysis
KCLB: Korea Cell Line Bank
KEGG: Kyoto Encyclopedia of Genes and Genomes
LPS: Lipopolysaccharide
MCP-1: Monocyte chemotactic protein 1
MIP1 α/β: Macrophage inflammatory protein alpha/beta
MOI: Multiplicity of infection
NO: Nitric oxide
PANTHER: Protein analysis through evolutionary relationships
PCR: Polymerase chain reaction
PTGS2: Prostaglandin-endoperoxide synthase 2
qRT-PCR: Quantitative real time polymerase chain reaction
RANTES: Regulated on activation, Normal T cell expressed and secreted
RPMI: Roswell Park Memorial Institute medium
TNF-α: Tumor necrosis factor alpha
Trim30: Tripartite-motif protein 30
