# Supplementary material for: Global gene-expression profiles of intracellular survival of the BruAb2_1031 gene mutated Brucella abortus in professional phagocytes, RAW 264.7 cells
Source: BMC Microbiol. 2018 Jul 31;18:82. doi: 10.1186/s12866-018-1223-7 (PMC6069796; doi:10.1186/s12866-018-1223-7)
Supplement: Supplementary file 1 — Table S1. Growth of B. abortus wild-type and mutant strains at each time point. CFU was calculated using the standard curve of CFU versus optical density. (PDF 19 kb) [file 12866_2018_1223_MOESM1_ESM.pdf]

Additional Table 1. Growth of *Brucella abortus* wild-type and mutant strains at each time point. CFU was calculated using the standard curve of CFU versus optical density.

	Log_{10}CFU	0 h	4 h	16 h	20 h	24 h	48 h	[1] %
	Wild	0 ± 0	7.50 ± 0.03	8.56 ± 0.15	9.13 ± 0.17	9.31 ± 0.02	9.60 ± 0.08	100 ± 2.43
[2] A	C3	0 ± 0	7.72 ± 0.05	8.06 ± 0.22	8.51 ± 0.60	8.77 ± 0.73	9.56 ± 0.54	87.70 ± 27.89
	C11	0 ± 0	7.43 ± 0.24	7.98 ± 0.05	8.25 ± 0.11	8.45 ± 0.30	9.28 ± 0.66	88.03 ± 22.59
	C12	0 ± 0	7.50 ± 0.08	8.05 ± 0.18	8.39 ± 0.37	8.50 ± 0.46	9.24 ± 0.74	82.71 ± 31.37
	C13	0 ± 0	7.35 ± 0.04	7.83 ± 0.20	7.99 ± 0.35	8.08 ± 0.45	8.88 ± 1.07	72.72 ± 28.80
[3] B	C1	0 ± 0	7.42 ± 0.05	8.32 ± 0.68	8.75 ± 1.02	8.92 ± 1.09	9.53 ± 0.48	100.52 ± 15.06
	C4	0 ± 0	7.44 ± 0.01	8.39 ± 0.65	8.81 ± 0.85	8.96 ± 0.89	9.75 ± 0.06	109.61 ± 2.48
	C5	0 ± 0	7.13 ± 0.18	7.91 ± 0.57	8.24 ± 0.65	8.53 ± 0.77	9.28 ± 0.33	102.36 ± 24.25
	C6	0 ± 0	7.73 ± 0.10	8.83 ± 0.02	9.31 ± 0.19	9.63 ± 0.11	10.02 ± 0.1	109.06 ± 9.49
	C7	0 ± 0	7.42 ± 0.05	8.20 ± 0.45	8.65 ± 0.69	8.84 ± 0.83	9.52 ± 0.23	99.88 ± 13.00
	C8	0 ± 0	7.33 ± 0.17	8.55 ± 0.08	9.16 ± 0.11	9.33 ± 0.23	9.60 ± 0.12	108.04 ± 2.46
	C9	0 ± 0	6.84 ± 0.34	8.25 ± 1.13	8.55 ± 1.25	8.60 ± 1.27	9.14 ± 0.66	109.43 ± 15.49
	C24	0 ± 0	6.15 ± 0.28	7.37 ± 0.25	7.79 ± 0.27	8.03 ± 0.22	8.33 ± 0.02	104.03 ± 1.13
	C26	0 ± 0	6.83 ± 0.18	7.89 ± 0.44	8.25 ± 0.56	8.46 ± 0.58	9.02 ± 0.04	104.16 ± 10.36
	C27	0 ± 0	6.63 ± 0.17	7.90 ± 0.02	8.43 ± 0.13	8.67 ± 0.12	8.91 ± 0.08	108.51 ± 4.23
	C32	0 ± 0	6.23 ± 0.21	6.87 ± 0.08	7.05 ± 0.21	7.19 ± 0.19	8.18 ± 0.42	92.86 ± 9.90
	C36	0 ± 0	7.08 ± 0.25	8.35 ± 0.11	8.82 ± 0.09	9.07 ± 0.07	9.31 ± 0.04	106.08 ± 9.88
	C37	0 ± 0	7.24 ± 0.34	8.29 ± 1.05	8.56 ± 1.28	8.71 ± 1.32	9.51 ± 0.33	107.78 ± 0.15
[4] C	C2	0 ± 0	7.12 ± 0.60	8.37 ± 0.31	8.89 ± 0.65	9.20 ± 0.65	9.91 ± 0.06	132.33 ± 31.43
	C14	0 ± 0	7.08 ± 0.10	8.02 ± 0.09	8.51 ± 0.03	8.82 ± 0.05	9.46 ± 0.18	113.22 ± 13.48
	C18	0 ± 0	6.89 ± 0.16	8.42 ± 0.27	8.83 ± 0.31	9.06 ± 0.26	9.38 ± 0.58	118.36 ± 7.53
	C19	0 ± 0	6.47 ± 0.46	7.58 ± 0.14	8.05 ± 0.12	8.34 ± 0.09	8.85 ± 0.08	113.03 ± 17.96
	C20	0 ± 0	6.38 ± 0.09	7.29 ± 0.57	7.75 ± 0.59	7.96 ± 0.72	8.81 ± 0.01	115.3 ± 4.77
	C21	0 ± 0	7.10 ± 0.21	8.32 ± 0.21	8.79 ± 0.25	9.05 ± 0.23	9.48 ± 0.03	112.86 ± 8.87
	C22	0 ± 0	6.65 ± 0.49	7.85 ± 0.22	8.29 ± 0.28	8.55 ± 0.29	9.02 ± 0.03	112.79 ± 22.01
	C23	0 ± 0	5.87 ± 0.38	7.22 ± 0.14	7.57 ± 0.06	7.86 ± 0.07	8.27 ± 0.06	113.93 ± 15.31
	C30	0 ± 0	5.55 ± 0.49	6.61 ± 0.32	7.00 ± 0.34	7.27 ± 0.37	7.90 ± 0.16	111.42 ± 16.14

[1] The level of growth rate was calculated by a gradient of CFU chagnes and presented as the relative percentage compared to that of the wild-type when regarded the level of wild-type as 100%. According to the relative growth rate, mutant strains were devided into the three groups. [2] Mutant strains in group A showed more thatn 10% reducement in growth rate. [3] Mutant strains in group B showed similar growth rate compared to that of the wild-type. [4] Mutant strains in group C showed more than 10% increasement in growth rate.
