# Supplementary material for: Global gene-expression profiles of intracellular survival of the BruAb2_1031 gene mutated Brucella abortus in professional phagocytes, RAW 264.7 cells
Source: BMC Microbiol. 2018 Jul 31;18:82. doi: 10.1186/s12866-018-1223-7 (PMC6069796; doi:10.1186/s12866-018-1223-7)
Supplement: Supplementary file 4 — Table S2. The genes showing altered expression in RAW 264.7 cells after B. abortus infection. The different expression levels in B. abortus infected RAW 264.7 cells were compared to uninfected cells. (PDF 1128 kb) [file 12866_2018_1223_MOESM4_ESM.pdf]

Additional Table 2. The genes showing altered expression in RAW 264.7 cells after *B. abortus infection*. The different expression levels in *B. abortus* infected RAW 264.7 cells were compared to uninfected cells.

Gene symbol	6 h		12 h		24 h		Gene accession No.	Gene description
	Fold changes (Log2 ratio)	*p* -value	Fold changes (Log2 ratio)	*p* -value	Fold changes (Log2 ratio)	*p* -value		
0610007P14Rik	-0.14 ± 0.05	0.734	-0.36 ± 0.06	0.001	-1.47 ± 0.07	$p < 0.001$	NM_021446	RIKEN cDNA 0610007P14 gene
1110032A03Rik	0.09 ± 0.18	1.000	-0.01 ± 0.32	1.000	1.37 ± 0.14	$p < 0.001$	NM_023483	RIKEN cDNA 1110032A03 gene
1110046J04Rik	0.17 ± 0.40	1.000	0.19 ± 0.42	1.000	1.23 ± 0.43	$p < 0.001$	NR_040707	RIKEN cDNA 1110046J04 gene
1190002N15Rik	0.03 ± 0.07	1.000	0.13 ± 0.04	0.769	-1.46 ± 0.16	$p < 0.001$	NM_001033145	RIKEN cDNA 1190002N15 gene
1500012F01Rik	0.04 ± 0.22	1.000	0.13 ± 0.13	0.917	2.14 ± 0.13	$p < 0.001$	NM_001081005	RIKEN cDNA 1500012F01 gene
1700020N15Rik	0.36 ± 0.24	1.000	0.25 ± 0.50	0.988	1.22 ± 0.28	0.001	NM_029316	RIKEN cDNA 1700020N15 gene
1700025G04Rik	-0.32 ± 0.08	0.017	-0.58 ± 0.06	$p < 0.001$	-1.21 ± 0.02	$p < 0.001$	NM_197990	RIKEN cDNA 1700025G04 gene
1700094D03Rik	-0.20 ± 0.04	1.000	-0.18 ± 0.05	0.995	1.64 ± 0.19	$p < 0.001$	NM_028567	RIKEN cDNA 1700094D03 gene
1700096K18Rik	0.43 ± 0.19	0.757	0.72 ± 0.07	0.030	1.77 ± 0.16	$p < 0.001$	NR_027388	RIKEN cDNA 1700096K18 gene
1700112E06Rik	-0.05 ± 0.15	1.000	0.14 ± 0.10	1.000	1.10 ± 0.07	$p < 0.001$	NM_028275	RIKEN cDNA 1700112E06 gene
1810055G02Rik	-0.14 ± 0.04	0.977	-0.37 ± 0.02	0.005	-1.23 ± 0.18	$p < 0.001$	NM_028077	RIKEN cDNA 1810055G02 gene
1810062G17Rik	-0.14 ± 0.33	1.000	0.14 ± 0.27	1.000	1.05 ± 0.28	$p < 0.001$	NM_028183	RIKEN cDNA 1810062G17 gene
2010002M12Rik	-0.02 ± 0.08	1.000	0.61 ± 0.81	0.228	1.20 ± 0.36	$p < 0.001$	NM_053217	RIKEN cDNA 2010002M12 gene
2310009A05Rik	0.11 ± 0.36	1.000	0.14 ± 0.14	0.989	1.02 ± 0.21	$p < 0.001$	NR_040377	RIKEN cDNA 2310009A05 gene
2310034O05Rik	-0.20 ± 0.11	1.000	-0.23 ± 0.35	0.963	1.02 ± 0.11	0.001	NR_040679	RIKEN cDNA 2310034O05 gene
2410002F23Rik	-0.07 ± 0.09	1.000	-0.55 ± 0.07	$p < 0.001$	-1.11 ± 0.09	$p < 0.001$	XM_006541085	RIKEN cDNA 2410002F23 gene
2410006H16Rik	0.23 ± 0.10	0.273	0.38 ± 0.11	0.002	1.80 ± 0.10	$p < 0.001$	NR_030738	RIKEN cDNA 2410006H16 gene
2410016O06Rik	-0.11 ± 0.07	1.000	-0.41 ± 0.08	0.001	-1.48 ± 0.07	$p < 0.001$	NM_023633	RIKEN cDNA 2410016O06 gene
2610008E11Rik	0.04 ± 0.36	1.000	0.25 ± 0.43	0.866	1.54 ± 0.35	$p < 0.001$	NM_001004362	RIKEN cDNA 2610008E11 gene
2610318N02Rik	-0.16 ± 0.13	0.988	-0.62 ± 0.15	$p < 0.001$	-1.52 ± 0.21	$p < 0.001$	XM_006522537	RIKEN cDNA 2610318N02 gene
2610524H06Rik	-0.22 ± 0.18	0.578	-0.14 ± 0.17	0.760	-1.16 ± 0.27	$p < 0.001$	NM_181075	RIKEN cDNA 2610524H06 gene
2700038G22Rik	-0.09 ± 0.33	1.000	-0.75 ± 0.30	0.010	-1.87 ± 0.30	$p < 0.001$	NR_045040	RIKEN cDNA 2700038G22 gene
2810417H13Rik	-0.13 ± 0.12	1.000	-0.24 ± 0.15	0.160	-1.99 ± 0.45	$p < 0.001$	ENSMUST00000045802	RIKEN cDNA 2810417H13 gene
2900026A02Rik	-0.24 ± 0.10	0.617	-0.70 ± 0.11	$p < 0.001$	-1.65 ± 0.05	$p < 0.001$	NM_172884	RIKEN cDNA 2900026A02 gene
3110043O21Rik	0.17 ± 0.18	0.930	0.68 ± 0.14	$p < 0.001$	1.36 ± 0.12	$p < 0.001$	NM_001081343	RIKEN cDNA 3110043O21 gene
3110045C21Rik	-0.28 ± 0.09	1.000	-0.43 ± 0.23	0.356	-1.35 ± 0.28	$p < 0.001$	NR_040438	RIKEN cDNA 3110045C21 gene
4833417C18Rik	-0.56 ± 0.32	0.248	-0.39 ± 0.17	0.412	-1.18 ± 0.36	$p < 0.001$	NR_045187	RIKEN cDNA 4833417C18 gene
4833419F23Rik	0.28 ± 0.18	1.000	0.51 ± 0.31	0.195	1.51 ± 0.13	$p < 0.001$	NR_040328	RIKEN cDNA 4833419F23 gene
4921524J17Rik	0.08 ± 0.12	1.000	0.10 ± 0.13	1.000	1.94 ± 0.20	$p < 0.001$	NM_025722	RIKEN cDNA 4921524J17 gene
4930427A07Rik	-0.20 ± 0.08	1.000	-0.62 ± 0.17	0.029	-1.04 ± 0.24	$p < 0.001$	NM_134041	RIKEN cDNA 4930427A07 gene
4930440I19Rik	0.03 ± 0.09	1.000	0.29 ± 0.06	0.694	1.63 ± 0.09	$p < 0.001$	NR_108098	RIKEN cDNA 4930440I19 gene
4930455G09Rik	0.08 ± 0.08	1.000	0.41 ± 0.19	0.160	1.63 ± 0.07	$p < 0.001$	XR_140681	RIKEN cDNA 4930455G09 gene
4930579G24Rik	-0.29 ± 0.19	0.140	-0.54 ± 0.10	$p < 0.001$	-1.67 ± 0.14	$p < 0.001$	NM_029482	RIKEN cDNA 4930579G24 gene
4931431B13Rik	0.00 ± 0.22	1.000	0.65 ± 0.14	0.142	1.36 ± 0.20	$p < 0.001$	NR_045183	RIKEN cDNA 4931431B13 gene
4933432I03Rik	0.29 ± 0.25	1.000	0.31 ± 0.02	0.789	1.93 ± 0.19	$p < 0.001$	NR_045657	RIKEN cDNA 4933432I03 gene
5031439G07Rik	-0.13 ± 0.01	1.000	-0.26 ± 0.09	0.103	-1.24 ± 0.04	$p < 0.001$	NM_001033273	RIKEN cDNA 5031439G07 gene
5430416N02Rik	-0.12 ± 0.18	1.000	-0.27 ± 0.24	0.610	1.78 ± 0.15	$p < 0.001$	NR_034038	RIKEN cDNA 5430416N02 gene

5430435G22Rik	-0.28 ± 0.03	0.066	-0.57 ± 0.03	$p < 0.001$	-1.93 ± 0.20	$p < 0.001$	NM_145509	RIKEN cDNA 5430435G22 gene
6430548M08Rik	-0.20 ± 0.01	0.767	-0.41 ± 0.08	0.008	-1.03 ± 0.03	$p < 0.001$	NM_001163760	RIKEN cDNA 6430548M08 gene
9430037G07Rik	-0.17 ± 0.05	1.000	-0.55 ± 0.18	0.296	-1.22 ± 0.30	$p < 0.001$	NR_040766	RIKEN cDNA 9430037G07 gene
9930012K11Rik	0.04 ± 0.08	1.000	-0.21 ± 0.11	0.929	-1.04 ± 0.08	$p < 0.001$	NM_001004155	RIKEN cDNA 9930012K11 gene
9930014A18Rik	0.26 ± 0.04	1.000	-0.35 ± 0.23	0.614	-1.52 ± 0.26	$p < 0.001$	NR_030696	RIKEN cDNA 9930014A18 gene
9930111J21Rik1	0.32 ± 0.33	0.705	0.73 ± 0.17	$p < 0.001$	1.15 ± 0.23	$p < 0.001$	NM_001114679	RIKEN cDNA 9930111J21 gene 1
9930111J21Rik2	0.57 ± 0.23	0.002	1.44 ± 0.23	$p < 0.001$	2.40 ± 0.22	$p < 0.001$	ENSMUST00000101295	RIKEN cDNA 9930111J21 gene 2
A130040M12Rik	0.65 ± 0.07	$p < 0.001$	2.00 ± 0.09	$p < 0.001$	3.50 ± 0.19	$p < 0.001$	AK037717	RIKEN cDNA A130040M12 gene
A1cf	0.06 ± 0.19	1.000	0.38 ± 0.23	0.773	1.16 ± 0.73	0.001	NM_001081074	APOBEC1 complementation factor
A530032D15Rik	0.14 ± 0.08	1.000	0.45 ± 0.12	0.001	1.05 ± 0.13	$p < 0.001$	NM_213615	RIKEN cDNA A530032D15Rik gene
A530040E14Rik	0.40 ± 0.02	0.936	0.68 ± 0.09	0.102	1.18 ± 0.20	$p < 0.001$	ENSMUST00000093501	RIKEN cDNA A530040E14 gene
A630033H20Rik	-0.17 ± 0.17	1.000	0.21 ± 0.22	0.834	1.17 ± 0.16	$p < 0.001$	NM_001122595	RIKEN cDNA A630033H20 gene
A630089N07Rik	-0.15 ± 0.12	1.000	-0.56 ± 0.11	0.011	-1.54 ± 0.14	$p < 0.001$	NR_015491	RIKEN cDNA A630089N07 gene
A730028G07Rik	0.02 ± 0.12	1.000	-0.31 ± 0.11	0.627	-1.06 ± 0.33	$p < 0.001$	AK042831	RIKEN cDNA A730028G07 gene
A730067D02Rik	0.25 ± 0.18	0.980	0.81 ± 0.15	$p < 0.001$	1.23 ± 0.10	$p < 0.001$	XM_006511595	RIKEN cDNA A730067D02 gene
AA987161	-0.10 ± 0.06	1.000	-0.13 ± 0.06	0.991	1.03 ± 0.07	$p < 0.001$	NM_001163246	expressed sequence AA987161
Aacs	-0.25 ± 0.06	0.323	-0.41 ± 0.05	0.003	-1.24 ± 0.04	$p < 0.001$	NM_030210	acetoacetyl-CoA synthetase
Abca5	0.22 ± 0.08	1.000	0.27 ± 0.15	0.767	1.27 ± 0.12	$p < 0.001$	NM_147219	ATP-binding cassette, sub-family A (ABC1), member 5
Abca8b	-0.05 ± 0.33	1.000	-0.04 ± 0.22	1.000	1.30 ± 0.22	$p < 0.001$	NM_013851	ATP-binding cassette, sub-family A (ABC1), member 8b
Abcd1	-0.15 ± 0.23	0.948	-0.46 ± 0.20	$p < 0.001$	-1.26 ± 0.18	$p < 0.001$	NM_007435	ATP-binding cassette, sub-family D (ALD), member 1
Abcf2	0.05 ± 0.06	1.000	-0.11 ± 0.05	0.835	-1.10 ± 0.03	$p < 0.001$	NM_001190443	ATP-binding cassette, sub-family F (GCN20), member 2
Abtb2	0.37 ± 0.01	0.597	0.56 ± 0.04	0.030	1.73 ± 0.12	$p < 0.001$	NM_178890	ankyrin repeat and BTB (POZ) domain containing 2
Acaca	-0.02 ± 0.01	1.000	-0.08 ± 0.06	0.985	-1.50 ± 0.03	$p < 0.001$	NM_133360	acetyl-Coenzyme A carboxylase alpha
Acap1	0.22 ± 0.27	1.000	0.51 ± 0.13	0.194	1.45 ± 0.22	$p < 0.001$	NM_153788	ArfGAP with coiled-coil, ankyrin repeat and PH domains 1
Acat1	0.02 ± 0.03	1.000	0.06 ± 0.04	1.000	-1.09 ± 0.12	$p < 0.001$	NM_144784	acetyl-Coenzyme A acetyltransferase 1
Acat2	-0.21 ± 0.12	0.627	-0.43 ± 0.12	0.002	-1.84 ± 0.25	$p < 0.001$	NM_009338	acetyl-Coenzyme A acetyltransferase 2
Acbd4	0.06 ± 0.16	1.000	0.39 ± 0.14	0.361	1.33 ± 0.16	$p < 0.001$	NM_025988	acyl-Coenzyme A binding domain containing 4
Acly	-0.08 ± 0.08	1.000	-0.19 ± 0.01	0.281	-1.32 ± 0.01	$p < 0.001$	NM_001199296	ATP citrate lyase
Aco1	-0.15 ± 0.18	0.918	-0.21 ± 0.07	0.289	-1.17 ± 0.18	$p < 0.001$	NM_007386	aconitase 1
Acot2	0.07 ± 0.07	1.000	0.27 ± 0.06	0.035	1.97 ± 0.01	$p < 0.001$	NM_134188	acyl-CoA thioesterase 2
Acp5	0.62 ± 0.40	0.292	0.52 ± 0.40	0.277	1.08 ± 0.46	$p < 0.001$	NM_001102404	acid phosphatase 5, tartrate resistant
Acsbg1	-0.24 ± 0.05	1.000	-0.25 ± 0.02	0.731	1.15 ± 0.15	$p < 0.001$	XM_006511588	acyl-CoA synthetase bubblegum family member 1
Acsl5	-0.02 ± 0.06	1.000	-0.16 ± 0.04	0.502	-1.27 ± 0.06	$p < 0.001$	NM_027976	acyl-CoA synthetase long-chain family member 5
Actg-ps1	-0.52 ± 0.13	$p < 0.001$	-0.82 ± 0.13	$p < 0.001$	-1.65 ± 0.15	$p < 0.001$	ENSMUST00000099636	actin, gamma, pseudogene 1
Actn1	-0.13 ± 0.06	0.712	-0.12 ± 0.08	0.586	-1.20 ± 0.06	$p < 0.001$	NM_134156	actinin, alpha 1
Adamts1	0.09 ± 0.09	1.000	0.42 ± 0.19	0.265	2.05 ± 0.11	$p < 0.001$	NM_009621	a disintegrin-like and metallopeptidase (reprolysin type) with thrombospondin type 1 motif, 1
Adamts6	-0.17 ± 0.28	1.000	0.61 ± 0.28	0.110	1.38 ± 0.35	$p < 0.001$	NM_001081020	a disintegrin-like and metallopeptidase (reprolysin type) with thrombospondin type 1 motif, 6
Add3	-0.24 ± 0.10	0.496	-0.17 ± 0.11	0.619	-1.05 ± 0.27	$p < 0.001$	NM_001164099	adducin 3 (gamma)
Adh7	0.36 ± 0.04	0.038	0.58 ± 0.03	$p < 0.001$	1.16 ± 0.07	$p < 0.001$	NM_009626	alcohol dehydrogenase 7 (class IV), mu or sigma polypeptide
Adhfe1	0.16 ± 0.07	1.000	0.43 ± 0.07	0.125	1.12 ± 0.09	$p < 0.001$	NM_175236	alcohol dehydrogenase, iron containing, 1
Adra1a	-0.24 ± 0.10	1.000	0.09 ± 0.15	1.000	1.46 ± 0.09	$p < 0.001$	NM_001271760	adrenergic receptor, alpha 1a

Adsl	-0.02 ± 0.05	1.000	-0.22 ± 0.05	0.216	-2.01 ± 0.05	$p < 0.001$	NM_009634	adenylosuccinate lyase
Adssl1	-0.19 ± 0.15	0.622	-0.62 ± 0.09	$p < 0.001$	-1.50 ± 0.18	$p < 0.001$	NM_007421	adenylosuccinate synthetase like 1
AF251705	-0.44 ± 0.42	0.686	-0.19 ± 0.23	0.977	1.31 ± 0.21	$p < 0.001$	NM_134158	cDNA sequence AF251705
Aff1	0.15 ± 0.09	0.918	0.23 ± 0.08	0.188	1.05 ± 0.07	$p < 0.001$	NM_001080798	AF4/FMR2 family, member 1
Aff4	0.17 ± 0.01	0.716	0.28 ± 0.04	0.054	1.05 ± 0.05	$p < 0.001$	NM_033565	AF4/FMR2 family, member 4
Aftph	0.27 ± 0.11	0.385	0.71 ± 0.12	$p < 0.001$	1.73 ± 0.15	$p < 0.001$	NM_001252503	aftiphilin
Agap1	0.41 ± 0.06	0.025	0.82 ± 0.08	$p < 0.001$	1.99 ± 0.06	$p < 0.001$	NM_001037136	ArfGAP with GTPase domain, ankyrin repeat and PH domain 1
Agfg2	-0.24 ± 0.09	0.397	-0.61 ± 0.11	$p < 0.001$	-1.70 ± 0.23	$p < 0.001$	NM_178162	ArfGAP with FG repeats 2
Aggf1	0.10 ± 0.18	1.000	-0.16 ± 0.17	0.537	-1.60 ± 0.17	$p < 0.001$	NM_025630	angiogenic factor with G patch and FHA domains 1
Ago2	0.15 ± 0.03	0.746	0.25 ± 0.01	0.083	1.03 ± 0.02	$p < 0.001$	NM_153178	argonaute RISC catalytic subunit 2
Agpat4	0.38 ± 0.28	0.481	0.60 ± 0.36	0.009	1.23 ± 0.29	$p < 0.001$	XM_006523345	1-acylglycerol-3-phosphate O-acyltransferase 4 (lysophosphatidic acid acyltransferase, delta)
Agpat9	0.35 ± 0.28	0.972	0.56 ± 0.25	0.162	1.64 ± 0.25	$p < 0.001$	ENSMUST00000092990	1-acylglycerol-3-phosphate O-acyltransferase 9
Ahnak2	0.20 ± 0.15	1.000	0.74 ± 0.11	0.011	3.83 ± 0.10	$p < 0.001$	XM_006516459	AHNAK nucleoprotein 2
Ahsa1	-0.01 ± 0.05	1.000	-0.11 ± 0.03	0.828	-1.10 ± 0.07	$p < 0.001$	NM_146036	AHA1, activator of heat shock protein ATPase 1
AI506816	0.09 ± 0.36	1.000	-0.55 ± 0.43	0.186	-1.19 ± 0.40	$p < 0.001$	NR_038090	expressed sequence AI506816
AI607873	0.53 ± 0.20	0.043	1.23 ± 0.21	$p < 0.001$	2.19 ± 0.19	$p < 0.001$	NM_001204910	expressed sequence AI607873
Aim2	0.12 ± 0.15	1.000	0.36 ± 0.08	0.015	1.05 ± 0.11	$p < 0.001$	NM_001013779	absent in melanoma 2
Ak4	-0.05 ± 0.10	1.000	0.14 ± 0.11	0.691	-1.26 ± 0.10	$p < 0.001$	NM_001177602	adenylate kinase 4
Akap1	-0.12 ± 0.15	1.000	-0.32 ± 0.09	0.077	-1.18 ± 0.17	$p < 0.001$	NM_001042541	A kinase (PRKA) anchor protein 1
Akap8	-0.11 ± 0.13	1.000	-0.39 ± 0.11	0.002	-1.18 ± 0.19	$p < 0.001$	NM_019774	A kinase (PRKA) anchor protein 8
Akna	0.40 ± 0.18	0.055	0.72 ± 0.15	$p < 0.001$	1.69 ± 0.17	$p < 0.001$	NM_001045514	AT-hook transcription factor
Akr1b7	-0.24 ± 0.41	1.000	-0.14 ± 0.46	1.000	2.31 ± 0.43	$p < 0.001$	NM_009731	aldo-keto reductase family 1, member B7
Akt1	-0.09 ± 0.07	1.000	-0.13 ± 0.10	0.706	-1.14 ± 0.06	$p < 0.001$	NM_001165894	thymoma viral proto-oncogene 1
Alad	-0.04 ± 0.06	1.000	-0.15 ± 0.02	0.649	-1.09 ± 0.09	$p < 0.001$	NM_001276446	aminolevulinate, delta-, dehydratase
Aldh1l2	0.42 ± 0.05	0.010	1.11 ± 0.09	$p < 0.001$	1.73 ± 0.05	$p < 0.001$	NM_153543	aldehyde dehydrogenase 1 family, member L2
Aldh4a1	-0.09 ± 0.14	1.000	-0.25 ± 0.13	0.789	-1.01 ± 0.14	$p < 0.001$	NM_175438	aldehyde dehydrogenase 4 family, member A1
Alg6	-0.13 ± 0.13	1.000	-0.48 ± 0.13	0.003	-1.28 ± 0.14	$p < 0.001$	NM_001081264	asparagine-linked glycosylation 6 (alpha-1,3,-glucosyltransferase)
Alms1	0.06 ± 0.06	1.000	-0.01 ± 0.08	1.000	-1.00 ± 0.10	$p < 0.001$	NM_145223	Alstrom syndrome 1
Amn1	0.39 ± 0.15	0.548	0.41 ± 0.17	0.260	1.68 ± 0.16	$p < 0.001$	NM_001113424	antagonist of mitotic exit network 1
Ampd3	-0.04 ± 0.09	1.000	0.04 ± 0.09	1.000	1.80 ± 0.11	$p < 0.001$	NM_001276301	adenosine monophosphate deaminase 3
Anapc2	-0.21 ± 0.03	0.595	-0.29 ± 0.15	0.065	-1.02 ± 0.03	$p < 0.001$	NM_175300	anaphase promoting complex subunit 2
Ang	-0.36 ± 0.12	0.018	-0.47 ± 0.12	$p < 0.001$	-1.13 ± 0.16	$p < 0.001$	NM_007447	angiogenin, ribonuclease, RNase A family, 5
Angptl2	-0.19 ± 0.11	0.799	-0.50 ± 0.06	$p < 0.001$	-1.26 ± 0.06	$p < 0.001$	NM_011923	angiopoietin-like 2
Angptl6	0.19 ± 0.20	1.000	0.46 ± 0.21	0.101	2.00 ± 0.20	$p < 0.001$	NM_145154	angiopoietin-like 6
Ank	-0.41 ± 0.05	$p < 0.001$	-0.66 ± 0.05	$p < 0.001$	-2.27 ± 0.04	$p < 0.001$	NM_020332	progressive ankylosis
Ankle1	-0.29 ± 0.13	0.362	-0.71 ± 0.22	$p < 0.001$	-1.93 ± 0.14	$p < 0.001$	NM_172756	ankyrin repeat and LEM domain containing 1
Ankrd26	0.00 ± 0.19	1.000	-0.25 ± 0.10	0.405	-1.34 ± 0.10	$p < 0.001$	NM_001081112	ankyrin repeat domain 26
Anln	0.11 ± 0.05	1.000	-0.18 ± 0.07	0.308	-2.06 ± 0.07	$p < 0.001$	NM_028390	anillin, actin binding protein
Antxr1	0.17 ± 0.04	1.000	0.47 ± 0.04	0.111	1.37 ± 0.26	$p < 0.001$	NM_054041	anthrax toxin receptor 1
Anxa3	0.33 ± 0.13	0.043	1.13 ± 0.12	$p < 0.001$	1.66 ± 0.15	$p < 0.001$	NM_013470	annexin A3
Aoah	-0.05 ± 0.15	1.000	0.18 ± 0.12	1.000	1.67 ± 0.18	$p < 0.001$	NM_012054	acyloxyacyl hydrolase

Aox2	0.18 ± 0.20	1.000	0.28 ± 0.15	0.814	1.00 ± 0.16	$p < 0.001$	NM_001008419	aldehyde oxidase 2
Ap1ar	-0.11 ± 0.05	0.962	-0.45 ± 0.12	$p < 0.001$	-1.52 ± 0.03	$p < 0.001$	NM_145964	adaptor-related protein complex 1 associated regulatory protein
Ap4b1	-0.11 ± 0.11	1.000	0.33 ± 0.12	0.117	1.11 ± 0.18	$p < 0.001$	NM_001163552	adaptor-related protein complex AP-4, beta 1
Apex1	-0.13 ± 0.04	1.000	-0.28 ± 0.05	0.057	-1.89 ± 0.17	$p < 0.001$	NM_009687	apurinic/apyrimidinic endonuclease 1
Apol9a	0.45 ± 0.17	0.090	1.50 ± 0.17	$p < 0.001$	2.36 ± 0.09	$p < 0.001$	NM_001162883	apolipoprotein L 9a
Apol9b	1.07 ± 0.55	$p < 0.001$	2.14 ± 0.55	$p < 0.001$	3.79 ± 0.55	$p < 0.001$	NM_001168660	apolipoprotein L 9b
Aqp9	0.62 ± 0.11	0.033	1.17 ± 0.12	$p < 0.001$	2.64 ± 0.10	$p < 0.001$	NM_001271843	aquaporin 9
Arap1	0.26 ± 0.12	0.335	0.38 ± 0.07	0.007	1.18 ± 0.03	$p < 0.001$	NM_001040111	ArfGAP with RhoGAP domain, ankyrin repeat and PH domain 1
Arfgap3	-0.14 ± 0.13	1.000	0.18 ± 0.23	0.710	1.10 ± 0.18	$p < 0.001$	NM_025445	ADP-ribosylation factor GTPase activating protein 3
Arg2	0.13 ± 0.21	1.000	0.15 ± 0.33	1.000	1.02 ± 0.20	$p < 0.001$	NM_009705	arginase type II
Arhgap11a	0.12 ± 0.03	1.000	-0.14 ± 0.04	0.666	-1.13 ± 0.10	$p < 0.001$	NM_181416	Rho GTPase activating protein 11A
Arhgap25	0.27 ± 0.07	0.106	-0.06 ± 0.12	1.000	1.14 ± 0.12	$p < 0.001$	NM_001037727	Rho GTPase activating protein 25
Arhgap32	-0.68 ± 0.18	0.017	-0.94 ± 0.17	$p < 0.001$	-1.65 ± 0.20	$p < 0.001$	NM_001195632	Rho GTPase activating protein 32
Arhgef3	0.24 ± 0.12	1.000	0.66 ± 0.12	0.033	1.44 ± 0.15	$p < 0.001$	NM_001289686	Rho guanine nucleotide exchange factor (GEF) 3
Arhgef39	-0.14 ± 0.04	1.000	-0.45 ± 0.06	0.024	-1.90 ± 0.02	$p < 0.001$	ENSMUST00000128754	Rho guanine nucleotide exchange factor (GEF) 39
Arid5a	0.48 ± 0.21	0.497	0.59 ± 0.22	0.093	1.31 ± 0.31	$p < 0.001$	NM_001172205	AT rich interactive domain 5A (MRF1-like)
Arl5b	0.14 ± 0.24	1.000	0.27 ± 0.19	0.386	1.44 ± 0.19	$p < 0.001$	ENSMUST00000069870	ADP-ribosylation factor-like 5B
Arl5c	0.25 ± 0.48	1.000	0.94 ± 0.56	$p < 0.001$	2.14 ± 0.49	$p < 0.001$	NM_207231	ADP-ribosylation factor-like 5C
Arrdc3	0.14 ± 0.18	1.000	-0.02 ± 0.17	1.000	1.03 ± 0.20	$p < 0.001$	NM_001042591	arrestin domain containing 3
Arrdc4	-0.03 ± 0.25	1.000	0.43 ± 0.26	0.002	2.18 ± 0.26	$p < 0.001$	NM_001042592	arrestin domain containing 4
Asb13	-0.20 ± 0.13	1.000	-0.60 ± 0.23	0.004	-1.27 ± 0.13	$p < 0.001$	NM_001267724	ankyrin repeat and SOCS box-containing 13
Ascc2	0.16 ± 0.13	0.958	0.41 ± 0.13	0.002	1.95 ± 0.13	$p < 0.001$	NM_029291	activating signal cointegrator 1 complex subunit 2
Ascc3	0.19 ± 0.03	0.546	0.70 ± 0.03	$p < 0.001$	1.25 ± 0.06	$p < 0.001$	NM_198007	activating signal cointegrator 1 complex subunit 3
Asf1b	-0.13 ± 0.02	0.965	-0.36 ± 0.02	0.002	-1.71 ± 0.12	$p < 0.001$	NM_024184	anti-silencing function 1B histone chaperone
Aspm	0.22 ± 0.03	0.665	-0.10 ± 0.13	0.965	-2.77 ± 0.43	$p < 0.001$	NM_009791	asp (abnormal spindle)-like, microcephaly associated (Drosophila)
Asxl1	0.09 ± 0.14	1.000	-0.14 ± 0.14	0.901	-1.09 ± 0.16	$p < 0.001$	ENSMUST00000109790	additional sex combs like 1
Atad2	-0.29 ± 0.08	0.040	-0.24 ± 0.09	0.088	-1.41 ± 0.23	$p < 0.001$	NM_027435	ATPase family, AAA domain containing 2
Atad3a	-0.06 ± 0.13	1.000	-0.30 ± 0.03	0.057	-1.08 ± 0.11	$p < 0.001$	NM_179203	ATPase family, AAA domain containing 3A
Atad5	-0.28 ± 0.18	0.507	-0.57 ± 0.17	0.001	-1.08 ± 0.23	$p < 0.001$	NM_001029856	ATPase family, AAA domain containing 5
Atf3	0.13 ± 0.14	0.980	0.87 ± 0.04	$p < 0.001$	2.31 ± 0.04	$p < 0.001$	NM_007498	activating transcription factor 3
Atg12	-0.01 ± 0.13	1.000	0.19 ± 0.19	0.807	1.50 ± 0.32	$p < 0.001$	NM_026217	autophagy related 12
Atg13	-0.09 ± 0.11	1.000	0.09 ± 0.09	0.977	1.31 ± 0.13	$p < 0.001$	ENSMUST00000028678	autophagy related 13
Atg9b	-0.11 ± 0.13	1.000	-0.26 ± 0.09	0.671	-1.28 ± 0.10	$p < 0.001$	NM_001002897	autophagy related 9B
Atic	-0.03 ± 0.10	1.000	-0.20 ± 0.10	0.289	-1.05 ± 0.12	$p < 0.001$	NM_026195	5-aminoimidazole-4-carboxamide ribonucleotide formyltransferase/IMP cyclohydrolase
Atm	-0.17 ± 0.10	0.905	0.09 ± 0.07	0.977	-1.06 ± 0.07	$p < 0.001$	NM_007499	ataxia telangiectasia mutated
Atp13a2	0.17 ± 0.02	0.720	0.03 ± 0.03	1.000	-1.58 ± 0.20	$p < 0.001$	NM_001164366	ATPase type 13A2
Atp13a4	0.28 ± 0.13	1.000	0.06 ± 0.21	1.000	2.14 ± 0.15	$p < 0.001$	NM_001164612	ATPase type 13A4
Atp1a3	-0.21 ± 0.05	0.372	-0.27 ± 0.05	0.068	-1.04 ± 0.13	$p < 0.001$	NM_001290469	ATPase, Na+/K+ transporting, alpha 3 polypeptide
Atp1b3	-0.07 ± 0.05	1.000	-0.23 ± 0.05	0.079	-1.08 ± 0.04	$p < 0.001$	NM_007502	ATPase, Na+/K+ transporting, beta 3 polypeptide
Atp5g3	-0.18 ± 0.04	0.601	-0.34 ± 0.06	0.008	-1.22 ± 0.14	$p < 0.001$	NM_175015	ATP synthase, H+ transporting, mitochondrial F0 complex, subunit C3 (subunit 9)
Atp5h	-0.10 ± 0.20	1.000	-0.56 ± 0.26	0.243	-1.24 ± 0.47	$p < 0.001$	ENSMUST00000138779	ATP synthase, H+ transporting, mitochondrial F0 complex, subunit D

Atp5k	0.11 ± 0.06	1.000	-0.07 ± 0.07	1.000	-1.23 ± 0.27	$p < 0.001$	ENSMUST00000049628	ATP synthase, H+ transporting, mitochondrial F1F0 complex, subunit E
Atp6v0a1	0.07 ± 0.04	1.000	-0.02 ± 0.04	1.000	1.08 ± 0.05	$p < 0.001$	NM_001243049	ATPase, H+ transporting, lysosomal V0 subunit A1
Atp6v1d	0.04 ± 0.12	1.000	0.13 ± 0.12	0.731	1.27 ± 0.13	$p < 0.001$	NM_023721	ATPase, H+ transporting, lysosomal V1 subunit D
Atp8b4	0.09 ± 0.05	1.000	0.54 ± 0.04	$p < 0.001$	1.69 ± 0.11	$p < 0.001$	NM_001080944	ATPase, class I, type 8B, member 4
Atp9b	0.04 ± 0.16	1.000	-0.06 ± 0.04	1.000	1.05 ± 0.09	$p < 0.001$	NM_001201569	ATPase, class II, type 9B
Atxn1	-0.03 ± 0.14	1.000	0.33 ± 0.09	0.159	1.36 ± 0.12	$p < 0.001$	NM_001199304	ataxin 1
AU020206	-0.25 ± 0.13	0.268	-0.14 ± 0.13	0.706	-1.37 ± 0.14	$p < 0.001$	ENSMUST00000181224	expressed sequence AU020206
Aurka	0.08 ± 0.13	1.000	-0.29 ± 0.13	0.031	-2.23 ± 0.24	$p < 0.001$	NM_001291185	aurora kinase A
Aurkb	-0.29 ± 0.16	0.056	-0.44 ± 0.09	$p < 0.001$	-1.95 ± 0.10	$p < 0.001$	NM_011496	aurora kinase B
Avil	-0.40 ± 0.04	0.003	-0.63 ± 0.06	$p < 0.001$	1.07 ± 0.13	$p < 0.001$	XM_006513102	advillin
AW011738	0.22 ± 0.29	1.000	0.59 ± 0.29	0.050	1.42 ± 0.33	$p < 0.001$	NR_030671	expressed sequence AW011738
AW549877	-0.11 ± 0.08	1.000	-0.09 ± 0.05	0.946	1.50 ± 0.07	$p < 0.001$	NM_145930	expressed sequence AW549877
B020010K11Rik	0.14 ± 0.40	1.000	-0.21 ± 0.48	0.650	-1.86 ± 0.46	$p < 0.001$	AK139683	RIKEN cDNA B020010K11 gene
B3galt6	0.03 ± 0.20	1.000	-0.14 ± 0.22	0.988	-1.01 ± 0.26	$p < 0.001$	NM_080445	UDP-Gal:betaGal beta 1,3-galactosyltransferase, polypeptide 6
B4galnt1	-0.08 ± 0.13	1.000	-0.37 ± 0.12	0.003	-1.02 ± 0.14	$p < 0.001$	NM_001244617	beta-1,4-N-acetyl-galactosaminyl transferase 1
B4galt3	-0.01 ± 0.07	1.000	0.11 ± 0.07	0.913	1.16 ± 0.08	$p < 0.001$	NM_020579	UDP-Gal:betaGlcNAc beta 1,4-galactosyltransferase, polypeptide 3
B4galt6	-0.20 ± 0.01	0.356	-0.47 ± 0.08	$p < 0.001$	-1.18 ± 0.03	$p < 0.001$	NM_019737	UDP-Gal:betaGlcNAc beta 1,4-galactosyltransferase, polypeptide 6
B630019K06Rik	-0.01 ± 0.09	1.000	-0.28 ± 0.15	0.406	-1.29 ± 0.64	$p < 0.001$	NR_045448	novel protein similar to F-box and leucine-rich repeat protein 17 (Fbxl17)
B930041F14Rik	-0.11 ± 0.21	1.000	-0.63 ± 0.12	0.042	-1.10 ± 0.13	$p < 0.001$	NM_178699	RIKEN cDNA B930041F14 gene
Bank1	0.37 ± 0.12	0.789	0.33 ± 0.11	0.616	1.65 ± 0.20	$p < 0.001$	NM_001033350	B cell scaffold protein with ankyrin repeats 1
Bard1	-0.16 ± 0.06	1.000	-0.28 ± 0.13	0.427	-1.29 ± 0.31	$p < 0.001$	NM_007525	BRCA1 associated RING domain 1
Bbc3	0.17 ± 0.13	1.000	0.36 ± 0.17	0.479	1.18 ± 0.13	$p < 0.001$	NM_133234	BCL2 binding component 3
Bbs4	0.07 ± 0.13	1.000	0.09 ± 0.16	1.000	1.81 ± 0.33	$p < 0.001$	NM_175325	Bardet-Biedl syndrome 4 (human)
BC005537	-0.18 ± 0.06	0.199	-0.22 ± 0.06	0.024	-1.27 ± 0.08	$p < 0.001$	NM_024473	cDNA sequence BC005537
BC030867	-0.04 ± 0.15	1.000	-0.41 ± 0.12	0.057	-1.36 ± 0.15	$p < 0.001$	NM_153544	cDNA sequence BC030867
BC055324	-0.13 ± 0.15	1.000	-0.36 ± 0.20	0.010	-1.42 ± 0.14	$p < 0.001$	XM_006496909	cDNA sequence BC055324
BC094916	0.14 ± 0.28	1.000	-0.07 ± 0.33	1.000	1.35 ± 0.08	$p < 0.001$	ENSMUST00000090406	cDNA sequence BC094916
Bckdk	-0.02 ± 0.10	1.000	-0.20 ± 0.08	0.221	-1.05 ± 0.07	$p < 0.001$	NM_009739	branched chain ketoacid dehydrogenase kinase
Bcl10	0.64 ± 0.08	$p < 0.001$	0.80 ± 0.04	$p < 0.001$	1.40 ± 0.06	$p < 0.001$	NM_009740	B cell leukemia/lymphoma 10
Bcl2a1a	0.16 ± 0.32	1.000	0.73 ± 0.29	$p < 0.001$	2.01 ± 0.29	$p < 0.001$	NM_009742	B cell leukemia/lymphoma 2 related protein A1a
Bcl2a1b	0.26 ± 0.06	0.252	0.84 ± 0.07	$p < 0.001$	1.87 ± 0.06	$p < 0.001$	ENSMUST00000068569	B cell leukemia/lymphoma 2 related protein A1b
Bcl2a1c	0.20 ± 0.35	1.000	0.63 ± 0.38	0.066	1.32 ± 0.27	$p < 0.001$	NM_007535	B cell leukemia/lymphoma 2 related protein A1c
Bcl2a1d	0.52 ± 0.11	$p < 0.001$	1.09 ± 0.13	$p < 0.001$	2.18 ± 0.15	$p < 0.001$	NM_007536	B cell leukemia/lymphoma 2 related protein A1d
Bcl3	0.88 ± 0.02	$p < 0.001$	0.89 ± 0.22	$p < 0.001$	2.00 ± 0.18	$p < 0.001$	NM_033601	B cell leukemia/lymphoma 3
Bcl7a	-0.23 ± 0.23	0.966	-0.25 ± 0.10	0.574	-1.19 ± 0.10	$p < 0.001$	ENSMUST00000120593	B cell CLL/lymphoma 7A
Bco2	0.39 ± 0.08	0.876	0.51 ± 0.07	0.257	1.20 ± 0.15	$p < 0.001$	NM_133217	beta-carotene oxygenase 2
Bend3	0.09 ± 0.24	1.000	-0.29 ± 0.25	0.593	-1.24 ± 0.42	$p < 0.001$	NM_199028	BEN domain containing 3
Bend6	0.18 ± 0.17	1.000	0.06 ± 0.18	1.000	1.03 ± 0.27	0.001	NM_177235	BEN domain containing 6
Bin2	0.08 ± 0.02	1.000	-0.06 ± 0.05	1.000	1.03 ± 0.02	$p < 0.001$	NM_001270537	bridging integrator 2
Birc2	0.16 ± 0.09	1.000	0.31 ± 0.10	0.413	1.05 ± 0.22	$p < 0.001$	NM_007465	baculoviral IAP repeat-containing 2
Birc3	0.58 ± 0.06	0.008	0.63 ± 0.03	$p < 0.001$	1.72 ± 0.13	$p < 0.001$	NM_007464	baculoviral IAP repeat-containing 3

Birc5	-0.12 ± 0.04	1.000	-0.57 ± 0.06	0.002	-2.65 ± 0.20	$p < 0.001$	NM_001012273	baculoviral IAP repeat-containing 5
Blm	-0.19 ± 0.01	0.592	-0.26 ± 0.01	0.082	-1.07 ± 0.10	$p < 0.001$	NM_001042527	Bloom syndrome, RecQ helicase-like
Blnk	0.24 ± 0.15	0.904	0.47 ± 0.11	0.027	2.08 ± 0.18	$p < 0.001$	NM_008528	B cell linker
Bms1	0.09 ± 0.03	1.000	-0.05 ± 0.02	1.000	-1.04 ± 0.02	$p < 0.001$	NM_194339	BMS1 homolog, ribosome assembly protein (yeast)
Bmyc	-0.20 ± 0.14	0.595	-0.51 ± 0.12	$p < 0.001$	-1.63 ± 0.15	$p < 0.001$	NM_023326	brain expressed myelocytomatosis oncogene
Bop1	0.05 ± 0.11	1.000	-0.09 ± 0.11	0.964	-1.08 ± 0.15	$p < 0.001$	NM_013481	block of proliferation 1
Bora	0.19 ± 0.12	0.616	-0.38 ± 0.04	0.004	-1.38 ± 0.06	$p < 0.001$	NM_175265	bora, aurora kinase A activator
Bpifc	-0.05 ± 0.16	1.000	-0.02 ± 0.17	1.000	1.66 ± 0.18	$p < 0.001$	NM_177772	BPI fold containing family C
Brca1	-0.15 ± 0.06	0.959	-0.34 ± 0.05	0.017	-1.61 ± 0.10	$p < 0.001$	NM_009764	breast cancer 1
Brip1	-0.20 ± 0.13	0.802	-0.16 ± 0.05	0.683	-1.86 ± 0.20	$p < 0.001$	NM_178309	BRCA1 interacting protein C-terminal helicase 1
Bst2	0.46 ± 0.23	0.006	1.73 ± 0.25	$p < 0.001$	2.14 ± 0.23	$p < 0.001$	NM_198095	bone marrow stromal cell antigen 2
Btbd6	-0.28 ± 0.12	0.532	-0.56 ± 0.17	0.002	-1.02 ± 0.16	$p < 0.001$	NM_001145900	BTB (POZ) domain containing 6
Btg1	0.32 ± 0.16	0.716	0.61 ± 0.13	0.007	2.20 ± 0.14	$p < 0.001$	NM_007569	B cell translocation gene 1, anti-proliferative
Btg2	0.44 ± 0.33	0.537	0.41 ± 0.33	0.351	1.81 ± 0.29	$p < 0.001$	NM_007570	B cell translocation gene 2, anti-proliferative
Btg3	0.36 ± 0.14	0.607	0.62 ± 0.14	0.008	1.94 ± 0.14	$p < 0.001$	NM_009770	B cell translocation gene 3
Bub1	0.04 ± 0.03	1.000	-0.10 ± 0.05	0.872	-1.07 ± 0.04	$p < 0.001$	NM_001113179	budding uninhibited by benzimidazoles 1 homolog (S. cerevisiae)
Bub1b	0.19 ± 0.08	0.578	-0.05 ± 0.08	1.000	-1.96 ± 0.26	$p < 0.001$	NM_009773	budding uninhibited by benzimidazoles 1 homolog, beta (S. cerevisiae)
C130050O18Rik	-0.41 ± 0.14	0.004	-0.72 ± 0.12	$p < 0.001$	-1.77 ± 0.12	$p < 0.001$	NM_177000	RIKEN cDNA C130050O18 gene
C230013L11Rik	-0.38 ± 0.18	0.980	-0.20 ± 0.07	0.993	1.47 ± 0.07	$p < 0.001$	AK082145	RIKEN cDNA C230013L11 gene
C230052I12Rik	0.14 ± 0.09	1.000	-0.21 ± 0.10	0.410	-1.13 ± 0.09	$p < 0.001$	NM_178643	RIKEN cDNA C230052I12 gene
C3	0.10 ± 0.05	1.000	0.82 ± 0.08	0.004	2.82 ± 0.04	$p < 0.001$	NM_009778	complement component 3
C330027C09Rik	0.02 ± 0.14	1.000	-0.18 ± 0.08	0.413	-1.64 ± 0.07	$p < 0.001$	NM_172616	RIKEN cDNA C330027C09 gene
C3ar1	0.00 ± 0.04	1.000	0.32 ± 0.12	0.009	1.33 ± 0.03	$p < 0.001$	NM_009779	complement component 3a receptor 1
Cad	0.00 ± 0.06	1.000	-0.03 ± 0.05	1.000	-1.52 ± 0.14	$p < 0.001$	NM_001289522	carbamoyl-phosphate synthetase 2, aspartate transcarbamylase, and dihydroorotase
Calcb	0.14 ± 0.49	1.000	0.61 ± 0.06	0.110	2.20 ± 0.06	$p < 0.001$	NM_054084	calcitonin-related polypeptide, beta
Calcrl	0.18 ± 0.06	0.582	0.46 ± 0.07	$p < 0.001$	1.05 ± 0.08	$p < 0.001$	NM_018782	calcitonin receptor-like
Cand1	-0.02 ± 0.04	1.000	-0.23 ± 0.05	0.170	-1.05 ± 0.09	$p < 0.001$	NM_027994	cullin associated and neddylation disassociated 1
Car12	-0.18 ± 0.04	0.876	0.21 ± 0.05	0.359	-1.40 ± 0.17	$p < 0.001$	NM_178396	carbonic anyhydrase 12
Car13	0.31 ± 0.03	0.091	0.88 ± 0.03	$p < 0.001$	1.64 ± 0.09	$p < 0.001$	NM_024495	carbonic anhydrase 13
Car6	0.69 ± 0.08	0.002	1.63 ± 0.02	$p < 0.001$	3.72 ± 0.02	$p < 0.001$	ENSMUST00000030817	carbonic anhydrase 6
Cars2	-0.18 ± 0.02	0.988	-0.32 ± 0.07	0.104	-1.38 ± 0.12	$p < 0.001$	NM_024248	cysteinyl-tRNA synthetase 2 (mitochondrial)(putative)
Casc5	-0.13 ± 0.06	0.975	-0.39 ± 0.05	0.001	-1.58 ± 0.05	$p < 0.001$	NM_029617	cancer susceptibility candidate 5
Casp1	0.12 ± 0.08	1.000	0.46 ± 0.11	$p < 0.001$	1.20 ± 0.22	$p < 0.001$	NM_009807	caspase 1
Casp3	-0.38 ± 0.11	0.059	-0.61 ± 0.08	$p < 0.001$	-1.19 ± 0.14	$p < 0.001$	NM_001284409	caspase 3
Casp4	1.20 ± 0.19	$p < 0.001$	2.30 ± 0.11	$p < 0.001$	4.61 ± 0.14	$p < 0.001$	NM_007609	caspase 4, apoptosis-related cysteine peptidase
Cat	-0.46 ± 0.04	$p < 0.001$	-0.82 ± 0.05	$p < 0.001$	-1.04 ± 0.02	$p < 0.001$	NM_009804	catalase
Cbr3	-0.06 ± 0.22	1.000	-0.01 ± 0.22	1.000	-1.01 ± 0.21	0.001	NM_173047	carbonyl reductase 3
Cbx3	0.03 ± 0.05	1.000	-0.09 ± 0.06	0.951	-1.33 ± 0.06	$p < 0.001$	NM_007624	chromobox 3
Cbx5	-0.10 ± 0.06	1.000	-0.28 ± 0.08	0.052	-1.50 ± 0.04	$p < 0.001$	NM_001076789	chromobox 5
Ccdc130	0.18 ± 0.21	0.960	0.13 ± 0.21	0.896	1.36 ± 0.13	$p < 0.001$	NM_001294281	coiled-coil domain containing 130
Ccdc138	-0.24 ± 0.22	1.000	-0.08 ± 0.07	1.000	-1.13 ± 0.04	$p < 0.001$	NM_001162956	coiled-coil domain containing 138

Ccdc14	-0.16 ± 0.21	1.000	-0.28 ± 0.10	0.469	-1.05 ± 0.21	p < 0.001	NM_172824	coiled-coil domain containing 14
Ccdc186	0.15 ± 0.15	1.000	0.52 ± 0.17	p < 0.001	1.19 ± 0.15	p < 0.001	NM_170757	coiled-coil domain containing 186
Ccdc85c	0.39 ± 0.35	0.380	-0.16 ± 0.36	0.955	-1.31 ± 0.31	p < 0.001	NM_001159910	coiled-coil domain containing 85C
Ccdc88a	0.15 ± 0.14	0.916	0.25 ± 0.03	0.146	1.02 ± 0.08	p < 0.001	NM_176841	coiled coil domain containing 88A
Ccl2	1.05 ± 0.04	p < 0.001	1.39 ± 0.04	p < 0.001	1.14 ± 0.08	p < 0.001	NM_011333	chemokine (C-C motif) ligand 2
Ccl5	0.82 ± 0.48	0.002	2.01 ± 0.37	p < 0.001	6.00 ± 0.32	p < 0.001	NM_013653	chemokine (C-C motif) ligand 5
Ccl7	0.69 ± 0.22	0.029	1.41 ± 0.20	p < 0.001	2.21 ± 0.20	p < 0.001	NM_013654	chemokine (C-C motif) ligand 7
Ccna2	-0.02 ± 0.02	1.000	-0.32 ± 0.02	0.005	-2.92 ± 0.12	p < 0.001	NM_009828	cyclin A2
Ccnb1	0.22 ± 0.06	0.317	-0.22 ± 0.10	0.193	-2.42 ± 0.16	p < 0.001	NM_172301	cyclin B1
Ccnb2	0.14 ± 0.04	0.889	-0.04 ± 0.01	1.000	-2.06 ± 0.04	p < 0.001	NM_007630	cyclin B2
Ccnd1	0.15 ± 0.04	0.511	0.05 ± 0.06	1.000	-2.02 ± 0.12	p < 0.001	NM_007631	cyclin D1
Ccnd3	-0.11 ± 0.02	1.000	-0.16 ± 0.13	0.703	-1.53 ± 0.05	p < 0.001	NM_001081635	cyclin D3
Ccne1	-0.28 ± 0.23	0.193	-0.40 ± 0.25	0.003	-2.38 ± 0.22	p < 0.001	NM_007633	cyclin E1
Ccne2	-0.39 ± 0.05	0.012	-0.55 ± 0.05	p < 0.001	-3.14 ± 0.01	p < 0.001	NM_001037134	cyclin E2
Ccnf	-0.11 ± 0.08	1.000	-0.69 ± 0.06	p < 0.001	-2.39 ± 0.12	p < 0.001	NM_007634	cyclin F
Ccng2	0.00 ± 0.09	1.000	1.04 ± 0.18	p < 0.001	0.53 ± 0.02	0.066	ENSMUST00000031331	cyclin G2
Ccr1	0.50 ± 0.35	0.428	0.58 ± 0.31	0.101	1.62 ± 0.32	p < 0.001	NM_009912	chemokine (C-C motif) receptor 1
Ccrl2	0.61 ± 0.72	0.286	1.77 ± 0.27	p < 0.001	3.96 ± 0.25	p < 0.001	NM_017466	chemokine (C-C motif) receptor-like 2
Cd24a	0.04 ± 0.57	1.000	-1.83 ± 0.57	p < 0.001	-2.24 ± 0.62	p < 0.001	NM_009846	CD24a antigen
Cd274	0.43 ± 0.12	0.189	0.97 ± 0.06	p < 0.001	3.08 ± 0.11	p < 0.001	NM_021893	CD274 antigen
Cd300ld	-0.14 ± 0.11	0.988	0.23 ± 0.10	0.195	1.89 ± 0.10	p < 0.001	NM_145437	CD300 molecule-like family member d
Cd3eap	-0.06 ± 0.04	1.000	-0.18 ± 0.04	0.728	-1.04 ± 0.29	p < 0.001	NM_145822	CD3E antigen, epsilon polypeptide associated protein
Cd40	0.55 ± 0.16	0.300	1.23 ± 0.20	p < 0.001	3.32 ± 0.21	p < 0.001	NR_027852	CD40 antigen
Cd46	0.20 ± 0.05	1.000	0.21 ± 0.15	0.998	1.14 ± 0.09	p < 0.001	NM_010778	CD46 antigen, complement regulatory protein
Cd47	0.09 ± 0.09	1.000	0.60 ± 0.07	p < 0.001	1.23 ± 0.07	p < 0.001	NM_010581	CD47 antigen (Rh-related antigen, integrin-associated signal transducer)
Cd72	-0.27 ± 0.06	0.042	-0.55 ± 0.05	p < 0.001	-1.13 ± 0.16	p < 0.001	NM_001110320	CD72 antigen
Cd80	0.40 ± 0.04	0.003	0.10 ± 0.12	0.912	1.98 ± 0.05	p < 0.001	NM_009855	CD80 antigen
Cd83	0.28 ± 0.16	0.957	0.48 ± 0.15	0.106	3.54 ± 0.14	p < 0.001	NM_001289915	CD83 antigen
Cd86	-0.14 ± 0.19	1.000	0.34 ± 0.25	0.700	2.51 ± 0.21	p < 0.001	XM_006521741	CD86 antigen
Cd9	0.00 ± 0.01	1.000	-0.04 ± 0.02	1.000	-1.33 ± 0.11	p < 0.001	NM_007657	CD9 antigen
Cdan1	0.07 ± 0.15	1.000	-0.23 ± 0.21	0.681	-1.26 ± 0.15	p < 0.001	NM_026891	congenital dyserythropoietic anemia, type I (human)
Cdc20	0.32 ± 0.06	0.019	-0.23 ± 0.08	0.144	-2.27 ± 0.08	p < 0.001	NM_023223	cell division cycle 20
Cdc25a	-0.08 ± 0.06	1.000	-0.47 ± 0.06	p < 0.001	-1.67 ± 0.08	p < 0.001	NM_007658	cell division cycle 25A
Cdc25b	0.17 ± 0.01	0.904	-0.08 ± 0.10	1.000	-1.25 ± 0.15	p < 0.001	NM_001111075	cell division cycle 25B
Cdc25c	0.40 ± 0.14	0.086	-0.09 ± 0.14	1.000	-1.26 ± 0.25	p < 0.001	NM_009860	cell division cycle 25C
Cdc6	-0.39 ± 0.08	0.010	-0.72 ± 0.13	p < 0.001	-2.01 ± 0.09	p < 0.001	NM_001025779	cell division cycle 6
Cdc7	-0.24 ± 0.05	0.723	-0.32 ± 0.06	0.176	-1.41 ± 0.10	p < 0.001	NM_001271566	cell division cycle 7 (S. cerevisiae)
Cdca2	-0.11 ± 0.05	1.000	-0.45 ± 0.09	p < 0.001	-1.95 ± 0.04	p < 0.001	NM_001110162	cell division cycle associated 2
Cdca3	-0.03 ± 0.06	1.000	-0.23 ± 0.04	0.312	-1.26 ± 0.32	p < 0.001	NM_013538	cell division cycle associated 3
Cdca5	-0.21 ± 0.13	0.529	-0.47 ± 0.14	p < 0.001	-1.65 ± 0.31	p < 0.001	NM_026410	cell division cycle associated 5
Cdca7	-0.08 ± 0.09	1.000	-0.28 ± 0.06	0.095	-1.81 ± 0.51	p < 0.001	NM_025866	cell division cycle associated 7

Cdca7l	-0.04 ± 0.06	1.000	-0.05 ± 0.05	1.000	-2.13 ± 0.06	$p < 0.001$	NM_146040	cell division cycle associated 7 like
Cdk1	-0.03 ± 0.02	1.000	-0.19 ± 0.02	0.354	-1.94 ± 0.14	$p < 0.001$	NM_007659	cyclin-dependent kinase 1
Cdk2	-0.22 ± 0.03	0.326	-0.35 ± 0.01	0.005	-1.70 ± 0.01	$p < 0.001$	NM_016756	cyclin-dependent kinase 2
Cdk2ap1	-0.02 ± 0.07	1.000	-0.36 ± 0.04	0.007	-1.19 ± 0.26	$p < 0.001$	ENSMUST00000104959	CDK2 (cyclin-dependent kinase 2)-associated protein 1
Cdkn2c	-0.15 ± 0.17	0.997	-0.52 ± 0.21	$p < 0.001$	-1.79 ± 0.21	$p < 0.001$	NM_007671	cyclin-dependent kinase inhibitor 2C (p18, inhibits CDK4)
Cdsn	-0.21 ± 0.33	1.000	0.07 ± 0.31	1.000	1.93 ± 0.30	$p < 0.001$	NM_001008424	corneodesmosin
Ceacam1	0.16 ± 0.06	1.000	0.67 ± 0.27	$p < 0.001$	1.82 ± 0.08	$p < 0.001$	NM_001039185	carcinoembryonic antigen-related cell adhesion molecule 1
Cebpb	0.37 ± 0.09	0.037	0.69 ± 0.05	$p < 0.001$	1.04 ± 0.09	$p < 0.001$	NM_001287738	CCAAT/enhancer binding protein (C/EBP), beta
Cebpd	0.39 ± 0.15	0.870	0.79 ± 0.37	0.011	1.08 ± 0.36	$p < 0.001$	NM_007679	CCAAT/enhancer binding protein (C/EBP), delta
Cebpg	0.01 ± 0.16	1.000	0.41 ± 0.16	0.003	1.13 ± 0.18	$p < 0.001$	NM_009884	CCAAT/enhancer binding protein (C/EBP), gamma
Cenpa	0.20 ± 0.09	0.549	-0.21 ± 0.06	0.278	-1.47 ± 0.09	$p < 0.001$	NM_007681	centromere protein A
Cenpb	0.07 ± 0.17	1.000	-0.24 ± 0.18	0.464	-1.57 ± 0.18	$p < 0.001$	NM_007682	centromere protein B
Cenpe	0.28 ± 0.11	0.073	-0.05 ± 0.11	1.000	-1.22 ± 0.16	$p < 0.001$	NM_173762	centromere protein E
Cenpf	0.18 ± 0.14	0.741	-0.13 ± 0.14	0.804	-2.34 ± 0.14	$p < 0.001$	NM_001081363	centromere protein F
Cenpi	0.09 ± 0.05	1.000	-0.20 ± 0.16	0.350	-1.52 ± 0.17	$p < 0.001$	NM_145924	centromere protein I
Cenpm	-0.12 ± 0.15	1.000	-0.42 ± 0.20	0.007	-1.46 ± 0.18	$p < 0.001$	NM_001080158	centromere protein M
Cenpt	0.26 ± 0.18	0.782	0.09 ± 0.25	1.000	-1.09 ± 0.19	$p < 0.001$	NM_177150	centromere protein T
Cenpu	-0.03 ± 0.09	1.000	-0.43 ± 0.22	0.078	-1.20 ± 0.08	$p < 0.001$	NM_027973	centromere protein U
Cep164	-0.10 ± 0.18	1.000	-0.25 ± 0.17	0.596	-1.30 ± 0.16	$p < 0.001$	NM_001081373	centrosomal protein 164
Cep55	0.06 ± 0.04	1.000	-0.30 ± 0.03	0.055	-1.83 ± 0.15	$p < 0.001$	NM_001164362	centrosomal protein 55
Cep68	0.02 ± 0.24	1.000	-0.26 ± 0.08	0.653	-1.88 ± 0.14	$p < 0.001$	NM_172260	centrosomal protein 68
Cep78	-0.13 ± 0.04	1.000	-0.38 ± 0.15	0.045	-1.36 ± 0.24	$p < 0.001$	NM_198019	centrosomal protein 78
Cflar	0.26 ± 0.01	0.429	0.57 ± 0.04	$p < 0.001$	2.16 ± 0.06	$p < 0.001$	NM_001289704	CASP8 and FADD-like apoptosis regulator
Chaf1a	-0.08 ± 0.06	1.000	-0.37 ± 0.13	0.028	-1.79 ± 0.21	$p < 0.001$	NM_013733	chromatin assembly factor 1, subunit A (p150)
Chaf1b	-0.27 ± 0.09	0.122	-0.32 ± 0.05	0.020	-1.89 ± 0.14	$p < 0.001$	NM_028083	chromatin assembly factor 1, subunit B (p60)
Chchd5	-0.12 ± 0.19	1.000	0.23 ± 0.16	0.713	1.27 ± 0.21	$p < 0.001$	NM_025395	coiled-coil-helix-coiled-coil-helix domain containing 5
Chd2	0.22 ± 0.08	0.906	0.43 ± 0.06	0.030	1.83 ± 0.12	$p < 0.001$	NM_001081345	chromodomain helicase DNA binding protein 2
Chd7	0.53 ± 0.13	0.002	0.84 ± 0.13	$p < 0.001$	1.09 ± 0.13	$p < 0.001$	NM_001277149	chromodomain helicase DNA binding protein 7
Chek1	-0.23 ± 0.11	0.593	-0.49 ± 0.09	0.001	-1.32 ± 0.21	$p < 0.001$	NM_007691	checkpoint kinase 1
Chek2	-0.12 ± 0.12	1.000	-0.33 ± 0.14	0.080	-1.04 ± 0.16	$p < 0.001$	NM_016681	checkpoint kinase 2
Chic2	0.19 ± 0.12	0.622	0.32 ± 0.12	0.030	1.05 ± 0.18	$p < 0.001$	NM_028850	cysteine-rich hydrophobic domain 2
Chmp4b	0.13 ± 0.07	1.000	0.49 ± 0.05	$p < 0.001$	1.08 ± 0.06	$p < 0.001$	NM_029362	charged multivesicular body protein 4B
Chst10	-0.44 ± 0.11	0.031	-0.68 ± 0.08	$p < 0.001$	-2.07 ± 0.10	$p < 0.001$	AK047378	carbohydrate sulfotransferase 10
Chst12	-0.30 ± 0.08	0.906	-0.38 ± 0.14	0.350	-1.28 ± 0.18	$p < 0.001$	ENSMUST00000043050	carbohydrate sulfotransferase 12
Chst3	-0.03 ± 0.16	1.000	-0.54 ± 0.14	0.176	-1.07 ± 0.17	$p < 0.001$	NM_016803	carbohydrate (chondroitin 6/keratan) sulfotransferase 3
Chtf18	-0.21 ± 0.08	0.831	-0.44 ± 0.08	0.016	-1.76 ± 0.40	$p < 0.001$	NM_145409	CTF18, chromosome transmission fidelity factor 18
Ckap2	0.26 ± 0.12	0.266	-0.07 ± 0.14	1.000	-1.42 ± 0.24	$p < 0.001$	NM_001004140	cytoskeleton associated protein 2
Ckap2l	0.06 ± 0.13	1.000	-0.23 ± 0.14	0.509	-1.74 ± 0.13	$p < 0.001$	NM_181589	cytoskeleton associated protein 2-like
Ckap4	0.01 ± 0.10	1.000	-0.38 ± 0.14	0.003	-1.69 ± 0.07	$p < 0.001$	NM_175451	cytoskeleton-associated protein 4
Clcn5	0.06 ± 0.06	1.000	0.32 ± 0.02	0.082	1.03 ± 0.02	$p < 0.001$	NM_001243762	chloride channel 5
Clcn7	0.06 ± 0.11	1.000	0.21 ± 0.09	0.312	1.47 ± 0.13	$p < 0.001$	NM_011930	chloride channel 7

Cldn34c3	0.03 ± 0.11	1.000	0.01 ± 0.10	1.000	1.23 ± 0.87	0.001	ENSMUST00000178148	predicted gene 5945//predicted gene 5167
Clec4e	0.86 ± 0.06	$p < 0.001$	1.37 ± 0.08	$p < 0.001$	2.04 ± 0.12	$p < 0.001$	NM_019948	C-type lectin domain family 4, member e
Clec7a	0.17 ± 0.26	1.000	0.67 ± 0.29	0.050	1.40 ± 0.23	$p < 0.001$	NM_020008	C-type lectin domain family 7, member a
Cln3	0.12 ± 0.11	1.000	-0.05 ± 0.11	1.000	1.05 ± 0.11	$p < 0.001$	XM_006507295	ceroid lipofuscinosis, neuronal 3, juvenile (Batten, Spielmeyer-Vogt disease)
Cln6	-0.06 ± 0.08	1.000	-0.33 ± 0.16	0.014	-1.33 ± 0.25	$p < 0.001$	NM_001033175	ceroid-lipofuscinosis, neuronal 6
Clspn	-0.22 ± 0.06	0.571	-0.41 ± 0.05	0.003	-1.92 ± 0.15	$p < 0.001$	NM_175554	claspin
Cluh	0.03 ± 0.15	1.000	-0.21 ± 0.14	0.249	-1.17 ± 0.22	$p < 0.001$	NM_001081158	clustered mitochondria (cluA/CLU1) homolog
Cmpk2	1.59 ± 0.04	$p < 0.001$	3.02 ± 0.06	$p < 0.001$	2.77 ± 0.06	$p < 0.001$	NM_020557	cytidine monophosphate (UMP-CMP) kinase 2, mitochondrial
Cnbd2	0.49 ± 0.10	0.595	0.81 ± 0.28	0.012	2.63 ± 0.15	$p < 0.001$	NM_027585	cyclic nucleotide binding domain containing 2
Cnnm3	-0.19 ± 0.13	1.000	-0.26 ± 0.11	0.769	-1.04 ± 0.19	$p < 0.001$	NM_001039551	cyclin M3
Commd6	0.12 ± 0.09	1.000	0.17 ± 0.02	0.605	1.38 ± 0.02	$p < 0.001$	NM_001168592	COMM domain containing 6
Coq9	-0.02 ± 0.07	1.000	0.20 ± 0.10	0.663	1.01 ± 0.10	$p < 0.001$	NM_026452	coenzyme Q9 homolog (yeast)
Coro1a	-0.30 ± 0.09	0.040	-0.21 ± 0.09	0.226	-1.01 ± 0.13	$p < 0.001$	NM_009898	coronin, actin binding protein 1A
Coro1c	-0.16 ± 0.09	0.569	-0.25 ± 0.09	0.053	-1.11 ± 0.09	$p < 0.001$	NM_011779	coronin, actin binding protein 1C
Cox6a2	-0.03 ± 0.12	1.000	-0.17 ± 0.08	0.523	1.23 ± 0.22	$p < 0.001$	NM_009943	cytochrome c oxidase subunit VIa polypeptide 2
Cp	-0.04 ± 0.15	1.000	0.86 ± 0.11	$p < 0.001$	3.01 ± 0.04	$p < 0.001$	NM_001276248	ceruloplasmin
Cpd	0.23 ± 0.06	0.180	0.92 ± 0.07	$p < 0.001$	1.15 ± 0.06	$p < 0.001$	NM_007754	carboxypeptidase D
Cpeb4	-0.10 ± 0.16	1.000	0.10 ± 0.16	0.920	1.23 ± 0.17	$p < 0.001$	NM_001290676	cytoplasmic polyadenylation element binding protein 4
Cpped1	-0.24 ± 0.11	0.983	-0.43 ± 0.07	0.136	-1.60 ± 0.08	$p < 0.001$	XM_006522014	calcineurin-like phosphoesterase domain containing 1
Cpt1a	-0.12 ± 0.04	1.000	-0.39 ± 0.05	0.033	-1.47 ± 0.09	$p < 0.001$	NM_013495	carnitine palmitoyltransferase 1a, liver
Crcp	-0.07 ± 0.10	1.000	0.08 ± 0.08	0.998	1.08 ± 0.07	$p < 0.001$	NM_007761	calcitonin gene-related peptide-receptor component protein
Creb3l2	-0.02 ± 0.27	1.000	0.11 ± 0.19	0.995	1.18 ± 0.18	$p < 0.001$	NM_178661	cAMP responsive element binding protein 3-like 2
Creb3l4	-0.17 ± 0.14	1.000	0.15 ± 0.05	1.000	1.56 ± 0.09	$p < 0.001$	NM_030080	cAMP responsive element binding protein 3-like 4
Creb5	0.47 ± 0.18	0.465	0.58 ± 0.15	0.072	1.98 ± 0.41	$p < 0.001$	NM_172728	cAMP responsive element binding protein 5
Crebrf	-0.24 ± 0.20	1.000	0.23 ± 0.13	0.931	1.39 ± 0.18	$p < 0.001$	NM_029870	CREB3 regulatory factor
Creld2	-0.19 ± 0.02	0.428	-0.31 ± 0.02	0.013	-1.20 ± 0.02	$p < 0.001$	NM_029720	cysteine-rich with EGF-like domains 2
Crem	0.09 ± 0.16	1.000	0.31 ± 0.04	0.747	1.02 ± 0.12	$p < 0.001$	NM_001110850	cAMP responsive element modulator
Crip1	-0.31 ± 0.16	0.084	-0.62 ± 0.09	$p < 0.001$	-2.83 ± 0.28	$p < 0.001$	NM_007763	cysteine-rich protein 1 (intestinal)
Crtap	-0.22 ± 0.03	0.319	-0.44 ± 0.04	$p < 0.001$	-1.69 ± 0.13	$p < 0.001$	NM_019922	cartilage associated protein
Cryl1	-0.13 ± 0.09	1.000	-0.14 ± 0.15	0.826	-1.28 ± 0.06	$p < 0.001$	NM_030004	crystallin, lambda 1
Cryzl1	0.31 ± 0.03	0.332	0.59 ± 0.02	$p < 0.001$	1.22 ± 0.07	$p < 0.001$	NM_026994	crystallin, zeta (quinone reductase)-like 1
Cse1l	-0.07 ± 0.03	1.000	-0.23 ± 0.07	0.071	-1.67 ± 0.06	$p < 0.001$	NM_023565	chromosome segregation 1-like (S. cerevisiae)
Csf1	0.62 ± 0.38	0.083	1.02 ± 0.39	$p < 0.001$	5.01 ± 0.38	$p < 0.001$	NM_001113530	colony stimulating factor 1 (macrophage)
Csf2	0.01 ± 0.13	1.000	0.03 ± 0.13	1.000	1.52 ± 0.14	$p < 0.001$	NM_009969	colony stimulating factor 2 (granulocyte-macrophage)
Csf3	-0.41 ± 0.17	0.793	0.92 ± 0.07	$p < 0.001$	2.24 ± 0.09	$p < 0.001$	NM_009971	colony stimulating factor 3 (granulocyte)
Csf3r	0.41 ± 0.02	0.442	0.94 ± 0.02	$p < 0.001$	1.51 ± 0.16	$p < 0.001$	NM_001252651	colony stimulating factor 3 receptor (granulocyte)
Csk	0.01 ± 0.13	1.000	-0.07 ± 0.04	1.000	-1.18 ± 0.03	$p < 0.001$	NM_007783	c-src tyrosine kinase
Csnk1g2	-0.06 ± 0.03	1.000	-0.16 ± 0.04	0.563	-1.28 ± 0.03	$p < 0.001$	NM_001159591	casein kinase 1, gamma 2
Csprs	0.68 ± 0.06	0.001	1.44 ± 0.08	$p < 0.001$	3.45 ± 0.09	$p < 0.001$	NM_033616	component of Sp100-rs
Csrnp1	-0.04 ± 0.18	1.000	0.52 ± 0.14	0.083	2.24 ± 0.16	$p < 0.001$	NM_153287	cysteine-serine-rich nuclear protein 1
Csrp2bp	-0.14 ± 0.12	1.000	-0.21 ± 0.15	0.310	-2.22 ± 0.20	$p < 0.001$	NM_181417	cysteine and glycine-rich protein 2 binding protein

Cth	0.56 ± 0.03	$p < 0.001$	1.23 ± 0.06	$p < 0.001$	1.68 ± 0.10	$p < 0.001$	NM_145953	cystathionase (cystathionine gamma-lyase)
Ctps	-0.04 ± 0.04	1.000	-0.07 ± 0.05	1.000	-1.51 ± 0.05	$p < 0.001$	NM_016748	cytidine 5-triphosphate synthase
Cwc27	0.13 ± 0.13	1.000	0.44 ± 0.14	0.004	1.15 ± 0.21	$p < 0.001$	NM_026072	CWC27 spliceosome-associated protein homolog (S. cerevisiae)
Cx3cr1	0.04 ± 0.11	1.000	-0.32 ± 0.12	0.310	-1.52 ± 0.24	$p < 0.001$	NM_009987	chemokine (C-X3-C motif) receptor 1
Cxcl10	0.91 ± 0.36	0.001	1.65 ± 0.33	$p < 0.001$	3.94 ± 0.30	$p < 0.001$	NM_021274	chemokine (C-X-C motif) ligand 10
Cxcl11	-0.20 ± 0.33	1.000	0.59 ± 0.40	0.116	2.97 ± 0.33	$p < 0.001$	NM_019494	chemokine (C-X-C motif) ligand 11
Cxcl16	-0.20 ± 0.41	1.000	0.19 ± 0.11	0.935	1.87 ± 0.09	$p < 0.001$	NM_023158	chemokine (C-X-C motif) ligand 16
Cxcr4	-0.51 ± 0.29	0.091	-0.74 ± 0.31	0.001	-1.07 ± 0.33	$p < 0.001$	NM_009911	chemokine (C-X-C motif) receptor 4
Cyb5r1	0.40 ± 0.07	0.005	0.80 ± 0.07	$p < 0.001$	1.63 ± 0.02	$p < 0.001$	NM_028057	cytochrome b5 reductase 1
Cybb	0.43 ± 0.04	$p < 0.001$	1.11 ± 0.03	$p < 0.001$	1.55 ± 0.08	$p < 0.001$	NM_007807	cytochrome b-245, beta polypeptide
Cyp2s1	-0.24 ± 0.20	1.000	-0.64 ± 0.06	0.038	-1.42 ± 0.15	$p < 0.001$	NM_028775	cytochrome P450, family 2, subfamily s, polypeptide 1
Cyp4f13	-0.08 ± 0.13	1.000	0.01 ± 0.02	1.000	1.08 ± 0.12	$p < 0.001$	NM_130882	cytochrome P450, family 4, subfamily f, polypeptide 13
Cyp4v3	0.26 ± 0.07	0.799	0.77 ± 0.07	$p < 0.001$	1.55 ± 0.08	$p < 0.001$	NM_133969	cytochrome P450, family 4, subfamily v, polypeptide 3
Cyp51	-0.26 ± 0.08	0.139	-0.63 ± 0.09	$p < 0.001$	-2.39 ± 0.07	$p < 0.001$	NM_020010	cytochrome P450, family 51
Cyth4	0.06 ± 0.02	1.000	-0.19 ± 0.07	0.174	-1.21 ± 0.02	$p < 0.001$	NM_028195	cytohesin 4
D17H6S56E-5	0.33 ± 0.05	0.014	0.05 ± 0.14	1.000	-1.84 ± 0.04	$p < 0.001$	AK088284	DNA segment, Chr 17, human D6S56E 5
D2Wsu81e	-0.07 ± 0.04	1.000	0.01 ± 0.12	1.000	-1.59 ± 0.11	$p < 0.001$	NM_172660	DNA segment, Chr 2, Wayne State University 81, expressed
D330050G23Rik	-0.10 ± 0.24	1.000	0.06 ± 0.19	1.000	1.38 ± 0.03	$p < 0.001$	NR_040335	RIKEN cDNA D330050G23 gene
D430020J02Rik	-0.03 ± 0.14	1.000	-0.22 ± 0.11	0.792	-1.48 ± 0.11	$p < 0.001$	NR_028421	RIKEN cDNA D430020J02 gene
D430036J16Rik	-0.18 ± 0.18	1.000	-0.71 ± 0.22	0.022	-1.20 ± 0.37	$p < 0.001$	NR_040393	RIKEN cDNA D430036J16 gene
Daam1	0.17 ± 0.08	1.000	0.57 ± 0.04	0.001	1.50 ± 0.04	$p < 0.001$	NM_001286452	dishevelled associated activator of morphogenesis 1
Dab2	-0.10 ± 0.04	1.000	-0.19 ± 0.07	0.138	-1.14 ± 0.06	$p < 0.001$	NM_001008702	disabled 2, mitogen-responsive phosphoprotein
Dag1	-0.16 ± 0.03	0.684	-0.17 ± 0.02	0.425	-1.29 ± 0.13	$p < 0.001$	NM_001276482	dystroglycan 1
Daxx	0.69 ± 0.22	$p < 0.001$	1.35 ± 0.25	$p < 0.001$	1.08 ± 0.26	$p < 0.001$	NM_001199733	Fas death domain-associated protein
Dbi	-0.05 ± 0.14	1.000	-0.06 ± 0.03	1.000	-1.01 ± 0.22	$p < 0.001$	NM_001037999	diazepam binding inhibitor
Dcaf6	0.20 ± 0.09	0.868	0.40 ± 0.06	0.020	1.11 ± 0.08	$p < 0.001$	NM_028759	DDB1 and CUL4 associated factor 6
Dcbld2	0.33 ± 0.07	0.150	0.58 ± 0.07	$p < 0.001$	1.26 ± 0.07	$p < 0.001$	NM_028523	discoidin, CUB and LCCL domain containing 2
Dclre1c	0.23 ± 0.05	0.909	0.62 ± 0.02	$p < 0.001$	1.10 ± 0.09	$p < 0.001$	NM_001110214	DNA cross-link repair 1C, PSO2 homolog (S. cerevisiae)
Dcstamp	0.05 ± 0.11	1.000	-0.30 ± 0.14	0.023	-1.39 ± 0.11	$p < 0.001$	NM_001289506	dentrocyte expressed seven transmembrane protein
Dctd	-0.17 ± 0.10	0.779	-0.35 ± 0.02	0.009	-1.72 ± 0.12	$p < 0.001$	NR_027759	dCMP deaminase
Dctpp1	0.01 ± 0.15	1.000	-0.36 ± 0.06	0.020	-1.09 ± 0.41	$p < 0.001$	NM_023203	dCTP pyrophosphatase 1
Ddb1	-0.01 ± 0.08	1.000	0.00 ± 0.09	1.000	-1.08 ± 0.13	$p < 0.001$	NM_015735	damage specific DNA binding protein 1
Ddit3	0.67 ± 0.18	0.001	1.42 ± 0.07	$p < 0.001$	3.03 ± 0.10	$p < 0.001$	NM_001290183	DNA-damage inducible transcript 3
Ddr2	0.28 ± 0.08	0.621	0.70 ± 0.12	$p < 0.001$	1.12 ± 0.08	$p < 0.001$	NM_022563	discoidin domain receptor family, member 2
Ddx11	0.12 ± 0.16	1.000	-0.09 ± 0.15	1.000	-1.37 ± 0.12	$p < 0.001$	NM_001003919	DEAD/H (Asp-Glu-Ala-Asp/His) box helicase 11
Ddx18	0.13 ± 0.01	0.972	-0.07 ± 0.02	0.994	-1.08 ± 0.06	$p < 0.001$	NM_025860	DEAD (Asp-Glu-Ala-Asp) box polypeptide 18
Ddx58	1.28 ± 0.20	$p < 0.001$	2.30 ± 0.19	$p < 0.001$	2.81 ± 0.21	$p < 0.001$	NM_172689	DEAD (Asp-Glu-Ala-Asp) box polypeptide 58
Ddx60	0.82 ± 0.29	0.047	2.13 ± 0.30	$p < 0.001$	3.63 ± 0.27	$p < 0.001$	NM_001293783	DEAD (Asp-Glu-Ala-Asp) box polypeptide 60
Deb1	-0.06 ± 0.03	1.000	-0.18 ± 0.01	0.630	1.11 ± 0.12	$p < 0.001$	NM_026794	differentially expressed in B16F10 1
Dennd4a	0.04 ± 0.05	1.000	0.32 ± 0.07	0.022	1.16 ± 0.03	$p < 0.001$	NM_001162917	DENN/MADD domain containing 4A
Denr	0.08 ± 0.08	1.000	0.27 ± 0.08	0.063	1.15 ± 0.18	$p < 0.001$	ENSMUST00000023869	density-regulated protein

Depdc1a	0.37 ± 0.11	0.025	-0.01 ± 0.11	1.000	-1.70 ± 0.26	$p < 0.001$	NM_001172092	DEP domain containing 1a
Depdc1b	0.27 ± 0.14	0.896	-0.14 ± 0.15	1.000	-1.56 ± 0.14	$p < 0.001$	NM_178683	DEP domain containing 1B
Dgcr8	-0.22 ± 0.08	0.771	-0.59 ± 0.06	$p < 0.001$	-1.48 ± 0.33	$p < 0.001$	NM_033324	DiGeorge syndrome critical region gene 8
Dgka	-0.07 ± 0.16	1.000	0.14 ± 0.08	0.996	1.42 ± 0.12	$p < 0.001$	NM_016811	diacylglycerol kinase, alpha
Dhcr24	-0.18 ± 0.06	0.440	-0.62 ± 0.03	$p < 0.001$	-4.00 ± 0.16	$p < 0.001$	NM_053272	24-dehydrocholesterol reductase
Dhcr7	-0.23 ± 0.08	0.250	-0.28 ± 0.06	0.042	-1.57 ± 0.10	$p < 0.001$	NM_007856	7-dehydrocholesterol reductase
Dhrs13	0.25 ± 0.21	1.000	-0.01 ± 0.19	1.000	-1.04 ± 0.20	$p < 0.001$	NM_183286	dehydrogenase/reductase (SDR family) member 13
Dhrs3	0.26 ± 0.14	0.117	0.82 ± 0.15	$p < 0.001$	1.40 ± 0.17	$p < 0.001$	ENSMUST00000154208	dehydrogenase/reductase (SDR family) member 3
Dhx40	0.12 ± 0.20	1.000	0.35 ± 0.07	0.580	1.30 ± 0.07	$p < 0.001$	ENSMUST00000131798	DEAH (Asp-Glu-Ala-His) box polypeptide 40
Dhx58	1.40 ± 0.20	$p < 0.001$	2.58 ± 0.21	$p < 0.001$	2.11 ± 0.22	$p < 0.001$	NM_030150	DEXH (Asp-Glu-X-His) box polypeptide 58
Dhx9	-0.14 ± 0.08	0.742	-0.36 ± 0.05	0.001	-2.25 ± 0.06	$p < 0.001$	NM_007842	DEAH (Asp-Glu-Ala-His) box polypeptide 9
Diap2	0.26 ± 0.09	0.502	0.91 ± 0.09	$p < 0.001$	1.74 ± 0.09	$p < 0.001$	NM_172493	diaphanous homolog 2 (Drosophila)
Dirc2	-0.09 ± 0.18	1.000	-0.13 ± 0.08	1.000	2.33 ± 0.20	$p < 0.001$	NM_153550	disrupted in renal carcinoma 2 (human)
Dkc1	-0.01 ± 0.01	1.000	-0.34 ± 0.01	0.007	-1.72 ± 0.08	$p < 0.001$	NM_001030307	dyskeratosis congenita 1, dyskerin
Dleu2	-0.36 ± 0.21	0.902	-0.91 ± 0.17	0.003	-1.09 ± 0.17	$p < 0.001$	AK137724	deleted in lymphocytic leukemia, 2
Dlg3	-0.10 ± 0.07	1.000	-0.38 ± 0.03	0.446	-1.27 ± 0.09	$p < 0.001$	NM_001177778	discs, large homolog 3 (Drosophila)
Dlgap5	0.12 ± 0.08	1.000	-0.27 ± 0.08	0.052	-1.49 ± 0.19	$p < 0.001$	NM_144553	discs, large (Drosophila) homolog-associated protein 5
Dmpk	-0.52 ± 0.16	0.055	-0.94 ± 0.14	$p < 0.001$	-1.83 ± 0.18	$p < 0.001$	NM_001190491	dystrophia myotonica-protein kinase
Dna2	-0.19 ± 0.08	0.694	-0.29 ± 0.09	0.068	-2.10 ± 0.12	$p < 0.001$	NM_177372	DNA replication helicase 2 homolog (yeast)
Dnajc9	-0.09 ± 0.03	1.000	-0.49 ± 0.10	$p < 0.001$	-1.44 ± 0.20	$p < 0.001$	NM_134081	DnaJ (Hsp40) homolog, subfamily C, member 9
Dnm1	0.17 ± 0.26	1.000	0.16 ± 0.16	0.935	1.30 ± 0.25	$p < 0.001$	NM_010065	dynamin 1
Dnmt1	-0.11 ± 0.05	1.000	-0.13 ± 0.05	0.627	-1.75 ± 0.07	$p < 0.001$	NM_001199431	DNA methyltransferase (cytosine-5) 1
Dnmt3aos	-0.21 ± 0.10	1.000	-0.54 ± 0.10	0.026	-1.51 ± 0.30	$p < 0.001$	NR_045884	DNA methyltransferase 3A, opposite strand
Dnph1	-0.07 ± 0.02	1.000	-0.31 ± 0.08	0.032	-1.64 ± 0.01	$p < 0.001$	NM_207161	2-deoxynucleoside 5-phosphate N-hydrolase 1
Dock10	0.04 ± 0.01	1.000	0.33 ± 0.03	0.009	1.41 ± 0.04	$p < 0.001$	NM_001285927	dedicator of cytokinesis 10
Dpep2	-0.25 ± 0.17	0.293	0.07 ± 0.07	1.000	1.39 ± 0.09	$p < 0.001$	NM_176913	dipeptidase 2
Dpf2	0.09 ± 0.06	1.000	0.08 ± 0.06	0.995	1.34 ± 0.05	$p < 0.001$	NM_001291078	D4, zinc and double PHD fingers family 2
Dpy19l1	-0.13 ± 0.12	1.000	-0.35 ± 0.10	0.007	-1.53 ± 0.11	$p < 0.001$	NM_172920	dpy-19-like 1 (C. elegans)
Dpy30	-0.06 ± 0.10	1.000	-0.30 ± 0.08	0.053	-1.04 ± 0.09	$p < 0.001$	NM_001146222	dpy-30 homolog (C. elegans)
Dscc1	-0.14 ± 0.23	1.000	-0.41 ± 0.09	0.343	-1.38 ± 0.43	$p < 0.001$	NM_183089	defective in sister chromatid cohesion 1 homolog (S. cerevisiae)
Dst	0.05 ± 0.10	1.000	0.68 ± 0.09	0.002	1.50 ± 0.09	$p < 0.001$	NM_001276764	dystonin
Dtl	-0.30 ± 0.03	0.071	-0.48 ± 0.03	$p < 0.001$	-1.54 ± 0.21	$p < 0.001$	NM_029766	denticleless homolog (Drosophila)
Dtwd1	0.40 ± 0.15	0.595	0.67 ± 0.19	0.010	1.07 ± 0.28	$p < 0.001$	NM_026981	DTW domain containing 1
Dtx3	0.24 ± 0.27	0.948	0.29 ± 0.16	0.413	1.02 ± 0.11	$p < 0.001$	XM_006514336	deltex 3 homolog (Drosophila)
Dtx3l	0.41 ± 0.09	0.039	1.02 ± 0.07	$p < 0.001$	0.93 ± 0.11	$p < 0.001$	NM_001013371	deltex 3-like (Drosophila)
Dtymk	-0.13 ± 0.04	1.000	-0.38 ± 0.04	0.004	-1.73 ± 0.10	$p < 0.001$	NM_001105667	deoxythymidylate kinase
Dus1l	-0.05 ± 0.18	1.000	-0.34 ± 0.11	0.044	-1.10 ± 0.07	$p < 0.001$	NM_026824	dihydrouridine synthase 1-like (S. cerevisiae)
Dusp1	0.20 ± 0.08	0.849	0.31 ± 0.02	0.114	2.94 ± 0.02	$p < 0.001$	NM_013642	dual specificity phosphatase 1
Dusp10	0.18 ± 0.16	1.000	0.53 ± 0.13	0.111	1.52 ± 0.15	$p < 0.001$	NM_022019	dual specificity phosphatase 10
Dusp16	0.43 ± 0.32	0.214	0.64 ± 0.32	0.001	2.16 ± 0.32	$p < 0.001$	NM_001048054	dual specificity phosphatase 16
Dusp4	0.18 ± 0.04	0.506	0.37 ± 0.08	0.001	1.22 ± 0.01	$p < 0.001$	NM_176933	dual specificity phosphatase 4

Dusp5	0.87 ± 0.08	$p < 0.001$	0.43 ± 0.13	0.050	1.73 ± 0.06	$p < 0.001$	ENSMUST00000038287	dual specificity phosphatase 5
Dusp8	0.14 ± 0.12	1.000	-0.05 ± 0.07	1.000	2.07 ± 0.07	$p < 0.001$	NM_008748	dual specificity phosphatase 8
Dut	-0.23 ± 0.10	0.401	-0.35 ± 0.10	0.010	-1.61 ± 0.10	$p < 0.001$	NM_001159646	deoxyuridine triphosphatase
E030011O05Rik	-0.13 ± 0.25	1.000	0.17 ± 0.20	1.000	1.03 ± 0.14	$p < 0.001$	NR_015511	RIKEN cDNA E030011O05 gene
E130112N10Rik	0.18 ± 0.05	1.000	0.33 ± 0.06	0.626	1.17 ± 0.05	$p < 0.001$	NR_015604	RIKEN cDNA E130112N10 gene
E2f1	-0.16 ± 0.09	0.872	-0.17 ± 0.10	0.518	-1.07 ± 0.13	$p < 0.001$	NM_001291105	E2F transcription factor 1
E2f7	-0.28 ± 0.10	0.625	-0.43 ± 0.15	0.044	-1.49 ± 0.15	$p < 0.001$	NM_178609	E2F transcription factor 7
E2f8	-0.23 ± 0.10	0.306	-0.52 ± 0.09	$p < 0.001$	-2.08 ± 0.14	$p < 0.001$	NM_001013368	E2F transcription factor 8
Ebi3	0.39 ± 0.05	0.094	1.19 ± 0.03	$p < 0.001$	2.59 ± 0.04	$p < 0.001$	NM_015766	Epstein-Barr virus induced gene 3
Ebna1bp2	0.04 ± 0.14	1.000	-0.06 ± 0.10	1.000	-1.31 ± 0.15	$p < 0.001$	NM_026932	EBNA1 binding protein 2
Ebp	0.07 ± 0.04	1.000	-0.29 ± 0.02	0.058	-1.41 ± 0.00	$p < 0.001$	NM_007898	phenylalkylamine Ca2+ antagonist (emopamil) binding protein
Echdc1	-0.01 ± 0.07	1.000	-0.01 ± 0.07	1.000	-1.08 ± 0.07	$p < 0.001$	NM_001110195	enoyl Coenzyme A hydratase domain containing 1
Ect2	-0.05 ± 0.11	1.000	-0.59 ± 0.10	$p < 0.001$	-1.73 ± 0.24	$p < 0.001$	NM_001177625	ect2 oncogene
Efr3b	-0.35 ± 0.02	0.015	-0.85 ± 0.09	$p < 0.001$	-2.77 ± 0.07	$p < 0.001$	NM_001082483	EFR3 homolog B (S. cerevisiae)
Egr1	0.82 ± 0.02	$p < 0.001$	0.68 ± 0.05	$p < 0.001$	2.02 ± 0.10	$p < 0.001$	NM_007913	early growth response 1
Ehd1	0.76 ± 0.08	$p < 0.001$	0.99 ± 0.12	$p < 0.001$	1.58 ± 0.17	$p < 0.001$	ENSMUST00000025684	EH-domain containing 1
Eif2ak2	0.59 ± 0.12	$p < 0.001$	1.20 ± 0.12	$p < 0.001$	1.25 ± 0.14	$p < 0.001$	NM_011163	eukaryotic translation initiation factor 2-alpha kinase 2
Eif3b	-0.07 ± 0.05	1.000	-0.10 ± 0.08	0.844	-1.20 ± 0.09	$p < 0.001$	NM_133916	eukaryotic translation initiation factor 3, subunit B
Eif3l	0.02 ± 0.02	1.000	-0.18 ± 0.01	0.348	-1.11 ± 0.07	$p < 0.001$	NM_145139	eukaryotic translation initiation factor 3, subunit L
Eif4g1	-0.03 ± 0.06	1.000	-0.03 ± 0.06	1.000	-1.06 ± 0.08	$p < 0.001$	NM_001005331	eukaryotic translation initiation factor 4, gamma 1
Eldr	-0.26 ± 0.27	1.000	-0.36 ± 0.36	0.642	-1.28 ± 0.28	$p < 0.001$	NR_110421	Egfr long non-coding downstream RNA
Elmo2	0.00 ± 0.04	1.000	-0.09 ± 0.04	0.966	-1.00 ± 0.10	$p < 0.001$	NM_080287	engulfment and cell motility 2
Elovl5	-0.18 ± 0.07	0.442	-0.24 ± 0.07	0.067	-1.26 ± 0.06	$p < 0.001$	NM_134255	ELOVL family member 5, elongation of long chain fatty acids (yeast)
Elovl6	-0.29 ± 0.01	0.056	-0.89 ± 0.16	$p < 0.001$	-2.38 ± 0.05	$p < 0.001$	NM_130450	ELOVL family member 6, elongation of long chain fatty acids (yeast)
Emc8	0.02 ± 0.08	1.000	-0.36 ± 0.11	0.007	-1.25 ± 0.14	$p < 0.001$	NM_010926	ER membrane protein complex subunit 8
Eme1	-0.21 ± 0.25	1.000	-0.86 ± 0.19	0.002	-1.97 ± 0.22	$p < 0.001$	NM_177752	essential meiotic endonuclease 1 homolog 1 (S. pombe)
Enc1	-0.29 ± 0.19	0.310	-0.62 ± 0.12	$p < 0.001$	-1.07 ± 0.12	$p < 0.001$	NM_007930	ectodermal-neural cortex 1
Endod1	-0.14 ± 0.03	1.000	-0.24 ± 0.06	0.455	-1.10 ± 0.05	$p < 0.001$	NM_028013	endonuclease domain containing 1
Enkd1	-0.17 ± 0.03	1.000	-0.15 ± 0.05	0.937	-1.06 ± 0.08	$p < 0.001$	NM_198299	enkurin domain containing 1
Eno2	-0.01 ± 0.04	1.000	0.45 ± 0.02	0.003	2.22 ± 0.04	$p < 0.001$	NM_013509	enolase 2, gamma neuronal
Entpd7	-0.17 ± 0.02	0.630	-0.27 ± 0.05	0.053	-1.18 ± 0.02	$p < 0.001$	NM_053103	ectonucleoside triphosphate diphosphohydrolase 7
Epg5	-0.02 ± 0.08	1.000	0.23 ± 0.02	0.719	1.39 ± 0.03	$p < 0.001$	NM_001195633	ectopic P-granules autophagy protein 5 homolog (C. elegans)
Epsti1	0.63 ± 0.17	0.002	1.84 ± 0.13	$p < 0.001$	1.03 ± 0.16	$p < 0.001$	NM_029495	epithelial stromal interaction 1 (breast)
Erbb2ip	0.22 ± 0.14	0.230	0.53 ± 0.09	$p < 0.001$	1.35 ± 0.13	$p < 0.001$	NM_001005868	Erbb2 interacting protein
Ercc6l	0.17 ± 0.04	1.000	-0.29 ± 0.20	0.643	-1.53 ± 0.04	$p < 0.001$	NM_146235	excision repair cross-complementing rodent repair deficiency complementation group 6 like
Erf	0.24 ± 0.09	1.000	-0.12 ± 0.45	1.000	-1.13 ± 0.08	$p < 0.001$	NM_010155	Ets2 repressor factor
Erlin1	-0.02 ± 0.06	1.000	0.03 ± 0.20	1.000	1.32 ± 0.16	$p < 0.001$	NM_001164359	ER lipid raft associated 1
Ern1	0.16 ± 0.04	1.000	0.51 ± 0.19	$p < 0.001$	2.00 ± 0.04	$p < 0.001$	NM_023913	endoplasmic reticulum (ER) to nucleus signalling 1
Ero1l	0.29 ± 0.03	0.010	1.38 ± 0.03	$p < 0.001$	1.72 ± 0.04	$p < 0.001$	NM_015774	ERO1-like (S. cerevisiae)
Erp29	-0.25 ± 0.02	0.158	-0.47 ± 0.03	$p < 0.001$	-2.03 ± 0.07	$p < 0.001$	NM_026129	endoplasmic reticulum protein 29
Errfi1	0.30 ± 0.04	0.893	0.19 ± 0.14	0.922	1.39 ± 0.10	$p < 0.001$	ENSMUST00000073600	ERBB receptor feedback inhibitor 1

Esco2	-0.34 ± 0.06	0.175	-0.61 ± 0.06	p < 0.001	-2.44 ± 0.25	p < 0.001	BC033303	establishment of cohesion 1 homolog 2 (S. cerevisiae)
Espl1	0.17 ± 0.04	0.960	-0.12 ± 0.15	0.890	-1.73 ± 0.06	p < 0.001	NM_001014976	extra spindle pole bodies 1 (S. cerevisiae)
Etaa1	-0.04 ± 0.13	1.000	-0.11 ± 0.10	1.000	-1.37 ± 0.18	p < 0.001	NM_026576	Ewing tumor-associated antigen 1
Ets2	0.80 ± 0.03	p < 0.001	0.99 ± 0.08	p < 0.001	1.80 ± 0.03	p < 0.001	NM_011809	E26 avian leukemia oncogene 2, 3 domain
Exo1	-0.22 ± 0.09	0.475	-0.71 ± 0.10	p < 0.001	-2.22 ± 0.35	p < 0.001	NM_012012	exonuclease 1
Exosc2	-0.23 ± 0.10	0.327	-0.39 ± 0.06	0.002	-1.01 ± 0.07	p < 0.001	NM_144886	exosome component 2
Extl1	0.44 ± 0.17	0.575	0.76 ± 0.16	0.007	1.43 ± 0.19	p < 0.001	NM_019578	exostoses (multiple)-like 1
Eya4	-0.33 ± 0.10	0.736	-0.12 ± 0.10	1.000	2.20 ± 0.14	p < 0.001	NM_010167	eyes absent 4 homolog (Drosophila)
Ezh2	-0.08 ± 0.03	1.000	-0.15 ± 0.03	0.628	-1.28 ± 0.18	p < 0.001	NM_001146689	enhancer of zeste homolog 2 (Drosophila)
F3	-0.08 ± 0.25	1.000	0.79 ± 0.20	0.019	1.10 ± 0.23	p < 0.001	NM_010171	coagulation factor III
Fads2	-0.11 ± 0.07	0.887	-0.43 ± 0.02	p < 0.001	-2.26 ± 0.06	p < 0.001	NM_019699	fatty acid desaturase 2
Fam102a	-0.20 ± 0.15	0.379	-0.48 ± 0.16	p < 0.001	-1.46 ± 0.17	p < 0.001	NM_153560	family with sequence similarity 102, member A
Fam111a	0.04 ± 0.05	1.000	-0.11 ± 0.00	0.862	-1.30 ± 0.12	p < 0.001	ENSMUST00000025595	family with sequence similarity 111, member A
Fam135a	0.21 ± 0.08	0.927	0.25 ± 0.13	0.422	1.35 ± 0.12	p < 0.001	NM_026604	family with sequence similarity 135, member A
Fam171b	-0.05 ± 0.16	1.000	0.39 ± 0.24	0.076	3.29 ± 0.15	p < 0.001	NM_175514	family with sequence similarity 171, member B
Fam185a	-0.17 ± 0.07	1.000	-0.45 ± 0.21	0.142	-1.16 ± 0.28	p < 0.001	NM_177869	family with sequence similarity 185, member A
Fam198b	-0.42 ± 0.11	0.001	-1.16 ± 0.06	p < 0.001	-2.16 ± 0.12	p < 0.001	NM_133187	family with sequence similarity 198, member B
Fam19a2	0.14 ± 0.18	1.000	0.15 ± 0.28	0.990	1.25 ± 0.24	p < 0.001	NR_045514	family with sequence similarity 19, member A2
Fam46c	0.07 ± 0.24	1.000	-0.19 ± 0.38	0.995	2.47 ± 0.10	p < 0.001	NM_001142952	family with sequence similarity 46, member C
Fam49a	0.04 ± 0.17	1.000	0.53 ± 0.18	0.085	2.34 ± 0.22	p < 0.001	NM_001146119	family with sequence similarity 49, member A
Fam50a	0.15 ± 0.05	0.865	0.27 ± 0.02	0.080	1.14 ± 0.13	p < 0.001	NM_138607	family with sequence similarity 50, member A
Fam57a	-0.17 ± 0.06	0.936	-0.29 ± 0.10	0.116	-1.04 ± 0.06	p < 0.001	NM_027773	family with sequence similarity 57, member A
Fam60a	0.10 ± 0.13	1.000	0.00 ± 0.05	1.000	-1.04 ± 0.14	p < 0.001	NM_019643	family with sequence similarity 60, member A
Fam64a	-0.03 ± 0.13	1.000	-0.36 ± 0.10	0.111	-1.29 ± 0.16	p < 0.001	NM_144526	family with sequence similarity 64, member A
Fam78a	-0.12 ± 0.10	1.000	-0.09 ± 0.12	1.000	-1.56 ± 0.26	p < 0.001	NM_175511	family with sequence similarity 78, member A
Fam83d	-0.05 ± 0.19	1.000	-0.35 ± 0.08	0.235	-2.16 ± 0.05	p < 0.001	NM_027975	family with sequence similarity 83, member D
Fanca	-0.09 ± 0.03	1.000	-0.49 ± 0.10	0.003	-2.27 ± 0.23	p < 0.001	NM_016925	Fanconi anemia, complementation group A
Fancb	-0.21 ± 0.11	1.000	-0.77 ± 0.10	0.001	-1.63 ± 0.12	p < 0.001	NM_001146081	Fanconi anemia, complementation group B
Fancd2	0.02 ± 0.06	1.000	-0.25 ± 0.05	0.130	-1.72 ± 0.13	p < 0.001	NM_001033244	Fanconi anemia, complementation group D2
Fanci	-0.14 ± 0.04	1.000	-0.56 ± 0.02	0.001	-1.98 ± 0.34	p < 0.001	NM_145946	Fanconi anemia, complementation group I
Fancm	-0.17 ± 0.09	0.963	-0.45 ± 0.02	0.002	-1.36 ± 0.19	p < 0.001	NM_178912	Fanconi anemia, complementation group M
Fas	0.44 ± 0.14	0.780	0.81 ± 0.14	0.017	3.27 ± 0.22	p < 0.001	NM_007987	Fas (TNF receptor superfamily member 6)
Fasn	-0.19 ± 0.06	0.387	-0.51 ± 0.03	p < 0.001	-3.24 ± 0.01	p < 0.001	NM_007988	fatty acid synthase
Fbxl14	-0.11 ± 0.09	1.000	-0.04 ± 0.09	1.000	-1.04 ± 0.12	p < 0.001	NM_133940	F-box and leucine-rich repeat protein 14
Fbxo21	-0.30 ± 0.01	0.163	-0.22 ± 0.10	0.293	-1.72 ± 0.21	p < 0.001	NM_145564	F-box protein 21
Fbxo31	-0.03 ± 0.07	1.000	-0.17 ± 0.08	0.755	-1.29 ± 0.14	p < 0.001	NM_133765	F-box protein 31
Fbxo5	-0.27 ± 0.07	0.198	-0.48 ± 0.06	p < 0.001	-2.59 ± 0.08	p < 0.001	NM_025995	F-box protein 5
Fcgr1	0.35 ± 0.10	0.012	1.19 ± 0.08	p < 0.001	1.91 ± 0.13	p < 0.001	NM_010186	Fc receptor, IgG, high affinity I
Fcgr2b	0.69 ± 0.18	p < 0.001	0.95 ± 0.18	p < 0.001	1.16 ± 0.18	p < 0.001	NM_001077189	Fc receptor, IgG, low affinity IIb
Fcgr4	0.10 ± 0.11	1.000	0.85 ± 0.08	p < 0.001	2.17 ± 0.08	p < 0.001	NM_144559	Fc receptor, IgG, low affinity IV
Fcrl1	-0.07 ± 0.05	1.000	-0.47 ± 0.04	p < 0.001	-2.07 ± 0.10	p < 0.001	NM_001136236	Fc receptor-like 1

Fdft1	-0.16 ± 0.11	0.692	-0.42 ± 0.09	p < 0.001	-1.65 ± 0.11	p < 0.001	NM_010191	farnesyl diphosphate farnesyl transferase 1
Fen1	-0.22 ± 0.09	0.630	-0.53 ± 0.10	p < 0.001	-1.26 ± 0.16	p < 0.001	ENSMUST00000025651	flap structure specific endonuclease 1
Fermt3	-0.05 ± 0.10	1.000	-0.22 ± 0.07	0.137	-1.06 ± 0.09	p < 0.001	NM_153795	fermitin family homolog 3 (Drosophila)
Fes	0.03 ± 0.03	1.000	-0.13 ± 0.19	0.815	-1.31 ± 0.02	p < 0.001	NM_010194	feline sarcoma oncogene
Fgd6	0.03 ± 0.08	1.000	0.32 ± 0.08	0.248	1.01 ± 0.11	p < 0.001	NM_053072	FYVE, RhoGEF and PH domain containing 6
Fh1	-0.12 ± 0.09	0.989	-0.27 ± 0.10	0.052	-1.17 ± 0.12	p < 0.001	NM_010209	fumarate hydratase 1
Fignl1	-0.19 ± 0.15	1.000	-0.45 ± 0.15	0.047	-1.53 ± 0.16	p < 0.001	NM_001163359	fidgetin-like 1
Filip1l	0.26 ± 0.09	1.000	0.30 ± 0.07	0.844	1.10 ± 0.40	p < 0.001	NM_001040397	filamin A interacting protein 1-like
Flrt2	0.05 ± 0.09	1.000	0.52 ± 0.11	p < 0.001	1.25 ± 0.10	p < 0.001	NM_201518	fibronectin leucine rich transmembrane protein 2
Fn3krp	-0.04 ± 0.22	1.000	-0.16 ± 0.17	0.981	-1.24 ± 0.35	p < 0.001	NM_181420	fructosamine 3 kinase related protein
Fos	0.37 ± 0.10	0.664	0.35 ± 0.16	0.455	1.27 ± 0.10	p < 0.001	ENSMUST00000134311	FBJ osteosarcoma oncogene
Foxk1	-0.10 ± 0.17	1.000	-0.15 ± 0.20	0.735	-1.06 ± 0.17	p < 0.001	NM_199068	forkhead box K1
Foxk2	-0.06 ± 0.06	1.000	-0.09 ± 0.05	0.935	-1.23 ± 0.06	p < 0.001	NM_001080932	forkhead box K2
Foxm1	-0.07 ± 0.11	1.000	-0.34 ± 0.10	0.023	-1.87 ± 0.16	p < 0.001	NM_008021	forkhead box M1
Foxn3	0.05 ± 0.15	1.000	0.49 ± 0.06	0.010	1.19 ± 0.05	p < 0.001	NM_183186	forkhead box N3
Fto	-0.14 ± 0.07	1.000	-0.12 ± 0.04	0.977	-1.02 ± 0.05	p < 0.001	NM_011936	fat mass and obesity associated
Fubp1	-0.20 ± 0.06	0.481	-0.33 ± 0.06	0.010	-1.39 ± 0.14	p < 0.001	NM_057172	far upstream element (FUSE) binding protein 1
Furin	0.04 ± 0.14	1.000	0.18 ± 0.11	0.690	1.79 ± 0.08	p < 0.001	NM_001081454	furin (paired basic amino acid cleaving enzyme)
Fus	-0.07 ± 0.06	1.000	-0.12 ± 0.06	0.779	-1.29 ± 0.19	p < 0.001	NM_139149	fused in sarcoma
Fyb	0.11 ± 0.07	1.000	0.36 ± 0.06	0.005	1.18 ± 0.07	p < 0.001	NM_001278269	FYN binding protein
Fzd7	-0.47 ± 0.11	0.027	-0.64 ± 0.11	p < 0.001	-1.74 ± 0.17	p < 0.001	NM_008057	frizzled homolog 7 (Drosophila)
G2e3	0.01 ± 0.12	1.000	-0.26 ± 0.11	0.147	-1.21 ± 0.13	p < 0.001	NM_001015099	G2/M-phase specific E3 ubiquitin ligase
G3bp1	0.07 ± 0.09	1.000	-0.19 ± 0.11	0.345	-1.36 ± 0.14	p < 0.001	ENSMUST00000018727	GTPase activating protein (SH3 domain) binding protein 1
G630090E17Rik	0.17 ± 0.79	1.000	0.28 ± 0.79	0.822	1.22 ± 0.76	p < 0.001	NM_001173500	RIKEN cDNA G630090E17 gene
Gab2	0.39 ± 0.16	0.005	0.71 ± 0.07	p < 0.001	1.39 ± 0.10	p < 0.001	NM_001162477	growth factor receptor bound protein 2-associated protein 2
Gabarapl1	-0.03 ± 0.09	1.000	0.07 ± 0.08	1.000	1.02 ± 0.08	p < 0.001	NM_020590	gamma-aminobutyric acid (GABA) A receptor-associated protein-like 1
Gadd45a	0.08 ± 0.08	1.000	0.41 ± 0.10	0.103	1.82 ± 0.02	p < 0.001	NM_007836	growth arrest and DNA-damage-inducible 45 alpha
Gadd45b	0.13 ± 0.07	1.000	0.51 ± 0.04	p < 0.001	3.32 ± 0.04	p < 0.001	NM_008655	growth arrest and DNA-damage-inducible 45 beta
Galnt3	0.48 ± 0.14	0.047	1.04 ± 0.06	p < 0.001	1.68 ± 0.06	p < 0.001	NM_015736	UDP-N-acetyl-alpha-D-galactosamine:polypeptide N-acetylgalactosaminyltransferase 3
Galnt6	0.00 ± 0.15	1.000	0.03 ± 0.12	1.000	1.02 ± 0.13	p < 0.001	NM_001161767	UDP-N-acetyl-alpha-D-galactosamine:polypeptide N-acetylgalactosaminyltransferase 6
Gamt	-0.25 ± 0.32	0.664	-0.43 ± 0.32	0.023	-1.62 ± 0.34	p < 0.001	NM_010255	guanidinoacetate methyltransferase
Gart	-0.08 ± 0.03	1.000	-0.31 ± 0.03	0.019	-2.40 ± 0.13	p < 0.001	ENSMUST00000120450	phosphoribosylglycinamide formyltransferase
Gas5	-0.42 ± 0.08	0.057	-0.68 ± 0.05	p < 0.001	-1.04 ± 0.25	p < 0.001	NR_002840	growth arrest specific 5
Gatm	-0.09 ± 0.09	1.000	0.03 ± 0.09	1.000	-1.15 ± 0.13	p < 0.001	NM_025961	glycine amidinotransferase (L-arginine:glycine amidinotransferase)
Gbp10	0.33 ± 0.15	0.703	0.80 ± 0.02	p < 0.001	1.62 ± 0.02	p < 0.001	NM_001039646	guanylate-binding protein 10
Gbp11	0.25 ± 0.32	1.000	0.16 ± 0.19	1.000	1.12 ± 0.23	0.002	NM_001039647	guanylate binding protein 11
Gbp2	0.50 ± 0.10	0.564	0.93 ± 0.14	0.001	1.77 ± 0.14	p < 0.001	NM_010260	guanylate binding protein 2
Gbp2b	0.41 ± 0.17	0.857	1.19 ± 0.22	p < 0.001	2.61 ± 0.31	p < 0.001	NM_010259	guanylate binding protein 2b
Gbp3	0.01 ± 0.21	1.000	1.29 ± 0.11	p < 0.001	2.38 ± 0.32	p < 0.001	NM_001289492	guanylate binding protein 3
Gbp5	0.23 ± 0.28	1.000	0.48 ± 0.30	0.490	1.67 ± 0.35	p < 0.001	NM_153564	guanylate binding protein 5
Gbp7	1.27 ± 0.05	p < 0.001	2.78 ± 0.10	p < 0.001	3.63 ± 0.16	p < 0.001	NM_001083312	guanylate binding protein 7

Gbp8	-0.01 ± 0.38	1.000	2.08 ± 0.12	$p < 0.001$	4.78 ± 0.18	$p < 0.001$	NM_029509	guanylate-binding protein 8
Gbp9	0.22 ± 0.30	1.000	1.52 ± 0.29	$p < 0.001$	2.90 ± 0.24	$p < 0.001$	NM_172777	guanylate-binding protein 9
Gcat	0.08 ± 0.08	1.000	-0.09 ± 0.08	1.000	-1.33 ± 0.48	$p < 0.001$	NM_013847	glycine C-acetyltransferase (2-amino-3-ketobutyrate-coenzyme A ligase)
Gch1	0.57 ± 0.03	0.001	1.16 ± 0.07	$p < 0.001$	1.42 ± 0.07	$p < 0.001$	NM_008102	GTP cyclohydrolase 1
Gclm	-0.60 ± 0.05	$p < 0.001$	-0.94 ± 0.02	$p < 0.001$	-1.80 ± 0.10	$p < 0.001$	NM_008129	glutamate-cysteine ligase, modifier subunit
Gdpd1	0.53 ± 0.03	0.002	0.98 ± 0.03	$p < 0.001$	1.90 ± 0.01	$p < 0.001$	NM_025638	glycerophosphodiester phosphodiesterase domain containing 1
Gdpd5	0.42 ± 0.23	0.049	0.50 ± 0.21	0.002	1.18 ± 0.21	$p < 0.001$	NM_201352	glycerophosphodiester phosphodiesterase domain containing 5
Gem	-0.23 ± 0.27	1.000	-0.20 ± 0.35	0.956	1.14 ± 0.23	$p < 0.001$	NM_010276	GTP binding protein (gene overexpressed in skeletal muscle)
Gemin4	0.22 ± 0.31	0.387	-0.29 ± 0.14	0.050	-1.42 ± 0.20	$p < 0.001$	NM_177367	gem (nuclear organelle) associated protein 4
Gen1	-0.07 ± 0.07	1.000	-0.38 ± 0.08	0.003	-1.63 ± 0.09	$p < 0.001$	NM_177331	Gen homolog 1, endonuclease (Drosophila)
Ghdc	0.08 ± 0.10	1.000	0.18 ± 0.13	0.900	1.02 ± 0.09	$p < 0.001$	NM_031871	GH3 domain containing
Ghitm	0.13 ± 0.09	0.858	0.41 ± 0.02	$p < 0.001$	1.11 ± 0.10	$p < 0.001$	NM_001199122	growth hormone inducible transmembrane protein
Gins1	-0.18 ± 0.09	0.859	-0.08 ± 0.10	1.000	-1.05 ± 0.09	$p < 0.001$	NM_027014	GINS complex subunit 1 (Psf1 homolog)
Gins2	-0.05 ± 0.10	1.000	-0.18 ± 0.13	0.559	-1.51 ± 0.10	$p < 0.001$	ENSMUST00000034278	GINS complex subunit 2 (Psf2 homolog)
Gins4	-0.06 ± 0.12	1.000	-0.18 ± 0.15	0.412	-1.16 ± 0.21	$p < 0.001$	NM_024240	GINS complex subunit 4 (Sld5 homolog)
Git1	-0.12 ± 0.19	1.000	-0.21 ± 0.20	0.304	-1.23 ± 0.19	$p < 0.001$	NM_001004144	G protein-coupled receptor kinase-interactor 1
Glce	0.22 ± 0.10	0.396	0.65 ± 0.11	$p < 0.001$	1.46 ± 0.12	$p < 0.001$	NM_033320	glucuronyl C5-epimerase
Gle1	0.02 ± 0.03	1.000	-0.20 ± 0.04	0.278	-1.07 ± 0.16	$p < 0.001$	NM_028923	GLE1 RNA export mediator (yeast)
Glipr2	0.41 ± 0.32	0.690	0.88 ± 0.31	0.001	1.37 ± 0.31	$p < 0.001$	NM_027450	GLI pathogenesis-related 2
Glrp1	0.38 ± 0.11	0.175	0.57 ± 0.20	0.001	1.11 ± 0.11	$p < 0.001$	NM_008132	glutamine repeat protein 1
Glud1	-0.18 ± 0.10	0.492	-0.23 ± 0.11	0.095	-1.00 ± 0.13	$p < 0.001$	NM_008133	glutamate dehydrogenase 1
Gm10384	-0.13 ± 0.01	1.000	-0.34 ± 0.03	0.750	-1.18 ± 0.78	$p < 0.001$	ENSMUST00000100713	predicted gene 10384
Gm10499	0.50 ± 0.48	0.049	0.47 ± 0.69	0.036	1.22 ± 0.47	$p < 0.001$	XM_006544796	predicted gene 10499
Gm10615	-0.65 ± 0.28	0.247	-0.61 ± 0.27	0.162	-1.06 ± 0.27	0.001	XR_140969	predicted gene 10615
Gm10718	-0.74 ± 0.25	$p < 0.001$	-1.06 ± 0.22	$p < 0.001$	-0.38 ± 0.49	0.015	ENSMUST00000099046	predicted gene 10718
Gm11084	-0.39 ± 1.42	0.928	-0.08 ± 0.65	1.000	-1.05 ± 0.64	0.002	ENSMUST00000112062	predicted gene 11084
Gm11496	0.03 ± 0.04	1.000	0.37 ± 0.04	0.590	1.12 ± 0.26	$p < 0.001$	ENSMUST00000128078	predicted gene 11496
Gm11517	-0.10 ± 0.64	1.000	-0.62 ± 0.60	0.334	1.25 ± 1.05	$p < 0.001$	NR_033523	ubiquitin A-52 residue ribosomal protein fusion product 1 pseudogene
Gm11545	-0.29 ± 0.10	0.299	-0.48 ± 0.09	0.002	-1.33 ± 0.17	$p < 0.001$	NM_001105561	predicted gene 11545
Gm11563	0.32 ± 0.54	1.000	0.43 ± 0.61	0.675	1.36 ± 0.54	$p < 0.001$	NM_001126320	predicted gene 11563
Gm11613	-0.23 ± 0.07	0.848	-0.23 ± 0.05	0.542	1.70 ± 0.07	$p < 0.001$	ENSMUST00000134427	predicted gene 11613
Gm11709	0.07 ± 0.43	1.000	-0.15 ± 0.42	1.000	1.49 ± 0.67	$p < 0.001$	ENSMUST00000106578	predicted gene 11709
Gm11710	-0.21 ± 0.11	0.663	0.13 ± 0.14	0.818	1.65 ± 0.12	$p < 0.001$	NM_001101656	predicted gene 11710
Gm11772	1.08 ± 0.32	$p < 0.001$	1.94 ± 0.27	$p < 0.001$	1.48 ± 0.27	$p < 0.001$	ENSMUST00000136542	predicted gene 11772
Gm11787	0.27 ± 0.19	1.000	0.96 ± 0.29	$p < 0.001$	1.14 ± 0.25	$p < 0.001$	XM_006538385	predicted gene 11787
Gm12003	-0.74 ± 0.09	$p < 0.001$	-0.76 ± 0.07	$p < 0.001$	-1.48 ± 0.28	$p < 0.001$	ENSMUST00000118518	predicted gene 12003
Gm12606	0.00 ± 0.13	1.000	0.16 ± 0.15	0.961	1.44 ± 0.20	$p < 0.001$	ENSMUST00000129527	predicted gene 12606
Gm12715	-0.30 ± 0.20	$p < 0.001$	-0.49 ± 0.19	$p < 0.001$	-1.10 ± 0.20	$p < 0.001$	ENSMUST00000121430	predicted gene 12715
Gm12744	-0.13 ± 0.20	1.000	-0.17 ± 0.06	0.986	-1.56 ± 0.14	$p < 0.001$	ENSMUST00000146976	predicted gene 12744
Gm12764	0.06 ± 0.18	1.000	0.28 ± 0.18	0.884	1.10 ± 0.28	$p < 0.001$	ENSMUST00000145961	predicted gene 12764
Gm12840	0.10 ± 0.49	1.000	0.20 ± 0.52	1.000	2.94 ± 0.10	$p < 0.001$	ENSMUST00000156081	predicted gene 12840

Gm13073	0.10 ± 0.20	1.000	0.28 ± 0.12	0.878	1.57 ± 0.22	$p < 0.001$	ENSMUST00000134059	predicted gene 13073
Gm13205	0.18 ± 0.04	1.000	0.12 ± 0.22	1.000	1.60 ± 0.14	$p < 0.001$	ENSMUST00000123548	predicted gene 13205
Gm13421	0.17 ± 0.17	1.000	0.39 ± 0.30	0.520	1.17 ± 0.17	$p < 0.001$	ENSMUST00000142511	predicted gene 13421
Gm13822	0.49 ± 0.15	0.639	0.53 ± 0.09	0.275	2.03 ± 0.07	$p < 0.001$	ENSMUST00000127563	predicted gene 13822
Gm14322	-0.07 ± 0.30	1.000	-0.55 ± 0.31	0.077	-1.34 ± 0.31	$p < 0.001$	NM_001243903	predicted gene 14322
Gm15441	-0.04 ± 0.12	1.000	0.15 ± 0.11	1.000	1.10 ± 0.29	$p < 0.001$	NR_040409	predicted gene 15441
Gm15466	0.01 ± 0.04	1.000	-0.06 ± 0.04	1.000	-1.10 ± 0.01	$p < 0.001$	XM_006543731	predicted gene 15466
Gm15649	0.33 ± 0.38	1.000	0.38 ± 0.35	0.595	1.40 ± 0.35	$p < 0.001$	ENSMUST00000133496	predicted gene 15649
Gm15856	0.33 ± 0.15	1.000	1.00 ± 0.36	$p < 0.001$	0.97 ± 0.14	$p < 0.001$	ENSMUST00000124853	predicted gene 15856
Gm16094	0.65 ± 0.53	0.247	0.90 ± 0.49	0.006	2.28 ± 0.43	$p < 0.001$	ENSMUST00000162784	predicted gene 16094
Gm16340	0.34 ± 0.11	1.000	0.54 ± 0.42	0.323	1.18 ± 0.31	$p < 0.001$	XM_006497081	predicted gene 16340
Gm16587	-0.08 ± 0.13	1.000	-0.11 ± 0.05	1.000	1.16 ± 0.23	0.001	ENSMUST00000161943	predicted gene 16587
Gm17035	-0.09 ± 0.09	1.000	0.51 ± 0.03	0.022	1.98 ± 0.11	$p < 0.001$	ENSMUST00000170557	predicted gene 17035
Gm17484	0.38 ± 0.18	0.981	0.18 ± 0.14	1.000	1.56 ± 0.29	$p < 0.001$	ENSMUST00000167899	predicted gene, 17484
Gm17757	0.73 ± 0.20	0.064	2.28 ± 0.19	$p < 0.001$	2.65 ± 0.19	$p < 0.001$	NR_040453	GTPase, very large interferon inducible 1 pseudogene
Gm20125	0.25 ± 0.12	1.000	0.33 ± 0.08	0.686	1.85 ± 0.26	$p < 0.001$	NR_038020	predicted gene, 20125
Gm20337	0.08 ± 0.55	1.000	0.32 ± 0.49	0.820	1.42 ± 0.50	$p < 0.001$	NR_045057	predicted gene, 20337
Gm20536	0.02 ± 0.17	1.000	0.05 ± 0.17	1.000	1.06 ± 0.48	$p < 0.001$	ENSMUST00000172588	predicted gene 20536
Gm20559	0.62 ± 0.05	0.006	1.32 ± 0.04	$p < 0.001$	2.64 ± 0.11	$p < 0.001$	AK035387	predicted gene, 20559
Gm20939	0.23 ± 0.19	0.793	0.17 ± 0.15	0.746	1.03 ± 0.13	$p < 0.001$	NM_001024731	predicted gene, 20939
Gm22069	0.55 ± 0.67	0.729	0.23 ± 0.67	1.000	1.04 ± 0.93	0.007	ENSMUST00000179911	predicted gene, 22069
Gm22077	0.33 ± 0.15	0.049	-0.01 ± 0.03	1.000	-1.32 ± 0.04	$p < 0.001$	ENSMUST00000157594	predicted gene, 22077
Gm22229	0.40 ± 0.14	1.000	0.26 ± 0.41	0.964	1.21 ± 0.57	$p < 0.001$	ENSMUST00000082987	predicted gene, 22229
Gm22303	-0.24 ± 0.07	0.196	-0.40 ± 0.06	0.001	-2.74 ± 0.06	$p < 0.001$	ENSMUST00000082686	predicted gene, 22303
Gm22327	-0.63 ± 0.29	0.396	-1.01 ± 0.53	0.008	0.05 ± 0.53	0.975	ENSMUST00000158899	predicted gene, 22327
Gm22455	-0.17 ± 0.45	1.000	-0.14 ± 0.43	0.984	-1.18 ± 0.48	$p < 0.001$	ENSMUST00000082580	predicted gene, 22455
Gm22502	1.34 ± 0.58	0.001	0.81 ± 0.57	0.104	0.91 ± 0.59	0.026	ENSMUST00000082488	predicted gene, 22502
Gm22503	0.05 ± 0.22	1.000	0.09 ± 0.08	1.000	1.03 ± 0.83	0.008	ENSMUST00000158983	predicted gene, 22503
Gm22571	0.19 ± 0.28	1.000	-0.10 ± 0.07	1.000	-1.43 ± 0.32	$p < 0.001$	ENSMUST00000082984	predicted gene, 22571
Gm22620	-0.24 ± 0.24	0.604	-0.73 ± 0.20	$p < 0.001$	-2.28 ± 0.34	$p < 0.001$	ENSMUST00000082700	predicted gene, 22620
Gm22645	-0.41 ± 0.59	1.000	-0.30 ± 0.61	0.936	1.25 ± 0.67	$p < 0.001$	ENSMUST00000180010	predicted gene, 22645
Gm22711	-0.10 ± 0.30	1.000	-0.85 ± 0.30	0.013	-1.93 ± 0.56	$p < 0.001$	ENSMUST00000116890	predicted gene, 22711
Gm22715	-0.07 ± 0.04	1.000	-0.52 ± 0.32	$p < 0.001$	-1.65 ± 0.78	$p < 0.001$	ENSMUST00000116899	predicted gene, 22715
Gm22740	-0.14 ± 0.17	1.000	0.73 ± 0.14	0.082	1.68 ± 0.15	$p < 0.001$	ENSMUST00000083439	predicted gene, 22740
Gm22743	-0.45 ± 0.52	0.804	-0.70 ± 0.61	0.110	-1.02 ± 0.49	0.004	ENSMUST00000083445	predicted gene, 22743
Gm22749	0.19 ± 0.45	1.000	0.53 ± 0.38	0.464	1.99 ± 0.62	$p < 0.001$	ENSMUST00000082490	predicted gene, 22749
Gm22762	0.13 ± 0.17	1.000	0.41 ± 0.20	0.700	1.02 ± 0.44	0.004	ENSMUST00000157395	predicted gene, 22762
Gm22771	-0.26 ± 0.38	1.000	-0.08 ± 0.16	1.000	-1.14 ± 1.06	0.001	ENSMUST00000104449	predicted gene, 22771
Gm22805	-0.21 ± 0.71	1.000	0.97 ± 0.90	0.012	1.89 ± 0.71	$p < 0.001$	ENSMUST00000082739	predicted gene, 22805
Gm22806	0.10 ± 0.60	1.000	-0.64 ± 0.65	0.141	-1.09 ± 0.75	0.001	ENSMUST00000082738	predicted gene, 22806
Gm22808	1.05 ± 0.50	0.016	0.80 ± 0.36	0.072	1.15 ± 0.29	0.001	ENSMUST00000082736	predicted gene, 22808

Gm22888	-0.29 ± 0.15	1.000	-0.73 ± 0.12	0.022	-1.18 ± 0.40	p < 0.001	ENSMUST00000158640	predicted gene, 22888
Gm22900	-0.51 ± 0.61	0.570	-0.71 ± 0.04	0.062	-1.10 ± 0.59	p < 0.001	ENSMUST00000104538	predicted gene, 22900
Gm22935	-0.75 ± 0.23	p < 0.001	-1.68 ± 0.29	p < 0.001	-3.82 ± 0.50	p < 0.001	ENSMUST00000103960	predicted gene, 22935
Gm22965	0.81 ± 0.59	0.099	0.83 ± 0.66	0.033	1.22 ± 0.60	p < 0.001	ENSMUST00000157958	predicted gene, 22965
Gm22972	0.55 ± 0.40	0.187	0.78 ± 0.30	0.002	1.37 ± 0.43	p < 0.001	ENSMUST00000083297	predicted gene, 22972
Gm23130	0.07 ± 0.16	1.000	-0.37 ± 0.25	0.036	-1.63 ± 0.25	p < 0.001	ENSMUST00000104252	predicted gene, 23130
Gm23166	0.71 ± 0.55	0.387	0.62 ± 0.46	0.323	1.11 ± 0.53	0.004	ENSMUST00000157164	predicted gene, 23166
Gm23294	-0.20 ± 0.17	1.000	-0.38 ± 0.21	0.763	1.16 ± 0.21	p < 0.001	ENSMUST00000104150	predicted gene, 23294
Gm23320	-0.06 ± 0.22	1.000	0.17 ± 0.67	1.000	1.35 ± 0.28	p < 0.001	ENSMUST00000104462	predicted gene, 23320
Gm23375	-0.72 ± 0.06	p < 0.001	-1.08 ± 0.08	p < 0.001	-2.04 ± 0.03	p < 0.001	ENSMUST00000175407	predicted gene, 23375
Gm23406	-0.16 ± 0.26	1.000	-0.41 ± 0.17	0.066	-1.99 ± 0.09	p < 0.001	ENSMUST00000082537	predicted gene, 23406
Gm23482	-0.09 ± 0.22	1.000	-0.29 ± 0.19	0.883	-1.27 ± 0.14	p < 0.001	ENSMUST00000104172	predicted gene, 23482
Gm23503	0.52 ± 0.40	0.428	0.36 ± 0.08	0.612	1.03 ± 0.20	p < 0.001	ENSMUST00000083903	predicted gene, 23503
Gm23613	-0.58 ± 0.16	0.108	-0.34 ± 0.27	0.439	-1.21 ± 0.47	p < 0.001	ENSMUST00000082596	predicted gene, 23613
Gm23722	0.33 ± 0.34	1.000	0.76 ± 0.31	0.017	1.13 ± 0.33	p < 0.001	ENSMUST00000083002	predicted gene, 23722
Gm23813	-0.53 ± 0.44	p < 0.001	-0.42 ± 0.41	0.001	-1.01 ± 0.44	p < 0.001	ENSMUST00000175153	predicted gene, 23813
Gm23817	0.21 ± 0.11	1.000	0.12 ± 0.23	1.000	1.13 ± 0.15	0.002	ENSMUST00000158449	predicted gene, 23817
Gm23917	0.31 ± 0.39	1.000	0.46 ± 0.09	0.640	1.10 ± 0.14	0.003	ENSMUST00000175256	predicted gene, 23917
Gm23957	1.02 ± 0.49	0.027	-0.52 ± 0.37	0.541	0.05 ± 0.42	0.981	ENSMUST00000157209	predicted gene, 23957
Gm24067	-0.15 ± 0.17	0.958	-0.69 ± 0.23	p < 0.001	-1.94 ± 0.49	p < 0.001	ENSMUST00000104514	predicted gene, 24067
Gm24082	0.59 ± 0.11	0.607	0.79 ± 0.66	0.090	1.03 ± 0.22	0.005	ENSMUST00000175175	predicted gene, 24082
Gm24089	-1.04 ± 0.45	p < 0.001	0.19 ± 0.41	0.385	-0.33 ± 0.40	0.025	ENSMUST00000093696	predicted gene, 24089
Gm24095	-0.05 ± 0.05	1.000	-0.68 ± 0.09	p < 0.001	-2.12 ± 0.19	p < 0.001	ENSMUST00000083063	predicted gene, 24095
Gm24104	-0.34 ± 0.40	0.696	-0.48 ± 0.45	0.102	-1.01 ± 0.40	p < 0.001	ENSMUST00000174935	predicted gene, 24104
Gm24124	-1.01 ± 1.23	0.056	-1.00 ± 1.32	0.023	-0.78 ± 1.24	0.070	ENSMUST00000104103	predicted gene, 24124
Gm24183	0.20 ± 0.18	1.000	0.48 ± 0.12	0.570	1.26 ± 0.12	p < 0.001	ENSMUST00000122572	predicted gene, 24183
Gm24225	0.82 ± 0.31	0.160	0.86 ± 0.29	0.048	1.08 ± 0.30	0.003	ENSMUST00000158825	predicted gene, 24225
Gm2427	-0.31 ± 0.29	1.000	0.28 ± 0.32	0.817	1.20 ± 0.38	p < 0.001	XM_001473524	predicted gene 2427
Gm24355	-0.27 ± 0.39	1.000	-0.20 ± 0.38	0.964	-2.25 ± 0.34	p < 0.001	ENSMUST00000082858	predicted gene, 24355
Gm24400	-0.20 ± 0.04	1.000	-0.54 ± 0.12	0.034	-1.27 ± 0.10	p < 0.001	ENSMUST00000157865	predicted gene, 24400
Gm24412	-0.15 ± 0.23	1.000	-0.30 ± 0.23	0.633	-1.33 ± 0.23	p < 0.001	ENSMUST00000083426	predicted gene, 24412
Gm24455	-0.05 ± 0.12	1.000	-0.53 ± 0.14	0.038	-1.52 ± 0.24	p < 0.001	ENSMUST00000083348	predicted gene, 24455
Gm24480	0.10 ± 0.24	1.000	0.64 ± 0.50	0.254	1.13 ± 0.47	0.002	ENSMUST00000157442	predicted gene, 24480
Gm24508	-1.12 ± 0.24	0.011	-0.99 ± 0.19	0.013	-0.16 ± 0.52	0.888	ENSMUST00000180104	predicted gene, 24508
Gm24517	0.55 ± 0.87	0.686	0.47 ± 0.82	0.580	1.67 ± 0.47	p < 0.001	ENSMUST00000122560	predicted gene, 24517
Gm24588	0.12 ± 0.21	1.000	0.10 ± 0.05	0.955	-1.46 ± 0.40	p < 0.001	ENSMUST00000116696	predicted gene, 24588
Gm24655	0.08 ± 0.42	1.000	0.03 ± 0.63	1.000	1.25 ± 0.72	p < 0.001	ENSMUST00000178790	predicted gene, 24655
Gm24831	0.25 ± 0.42	1.000	0.15 ± 0.23	1.000	1.16 ± 0.46	0.001	ENSMUST00000116778	predicted gene, 24831
Gm24888	-0.65 ± 0.30	p < 0.001	-0.56 ± 0.08	p < 0.001	-1.72 ± 0.08	p < 0.001	ENSMUST00000104387	predicted gene, 24888
Gm24916	-0.54 ± 0.29	0.428	-0.72 ± 0.09	0.044	-1.33 ± 0.27	p < 0.001	ENSMUST00000157287	predicted gene, 24916
Gm24959	1.02 ± 0.30	0.010	0.37 ± 0.30	0.733	0.41 ± 0.44	0.443	ENSMUST00000157924	predicted gene, 24959

Gm24996	-0.60 ± 0.55	0.635	-0.50 ± 0.75	0.561	-1.05 ± 0.56	0.007	ENSMUST00000158390	predicted gene, 24996
Gm25188	0.13 ± 0.17	1.000	-0.30 ± 0.17	0.694	-1.23 ± 0.14	p < 0.001	ENSMUST00000083913	predicted gene, 25188
Gm25335	0.83 ± 0.89	0.106	0.07 ± 0.89	1.000	1.45 ± 0.98	p < 0.001	ENSMUST00000158043	predicted gene, 25335
Gm25432	0.10 ± 0.20	1.000	-0.45 ± 0.22	p < 0.001	-2.17 ± 0.11	p < 0.001	ENSMUST00000158284	predicted gene, 25432
Gm25482	-0.74 ± 0.61	0.146	-1.60 ± 0.57	p < 0.001	-0.51 ± 0.88	0.230	ENSMUST00000083225	predicted gene, 25482
Gm25544	-0.29 ± 0.10	1.000	-1.06 ± 0.18	0.001	-1.98 ± 0.30	p < 0.001	ENSMUST00000122676	predicted gene, 25544
Gm25794	0.19 ± 0.23	1.000	1.11 ± 0.74	0.005	0.55 ± 0.77	0.297	ENSMUST00000083286	predicted gene, 25794
Gm25834	-0.29 ± 0.22	1.000	0.21 ± 0.22	1.000	1.33 ± 0.42	p < 0.001	ENSMUST00000158916	predicted gene, 25834
Gm25909	-0.05 ± 0.58	1.000	0.17 ± 0.88	1.000	1.58 ± 0.50	p < 0.001	ENSMUST00000157619	predicted gene, 25909
Gm25930	-0.31 ± 0.46	1.000	-0.42 ± 0.66	0.610	1.26 ± 0.50	p < 0.001	ENSMUST00000082785	predicted gene, 25930
Gm25987	0.31 ± 0.35	1.000	0.30 ± 0.57	0.771	1.01 ± 0.67	p < 0.001	ENSMUST00000083234	predicted gene, 25987
Gm26052	1.23 ± 0.66	0.002	0.34 ± 0.78	0.851	0.66 ± 0.58	0.140	ENSMUST00000104646	predicted gene, 26052
Gm26143	0.94 ± 0.45	0.001	1.45 ± 0.53	p < 0.001	2.36 ± 0.47	p < 0.001	ENSMUST00000104480	predicted gene, 26143
Gm26225	-0.69 ± 0.27	0.008	-0.58 ± 0.24	0.014	-1.42 ± 0.12	p < 0.001	ENSMUST00000082508	predicted gene, 26225
Gm26387	-0.17 ± 0.03	1.000	-0.45 ± 0.07	0.016	-1.02 ± 0.18	p < 0.001	ENSMUST00000104238	predicted gene, 26387
Gm26526	0.07 ± 0.21	1.000	0.25 ± 0.12	0.890	1.07 ± 0.12	p < 0.001	ENSMUST00000181075	predicted gene, 26526
Gm26611	0.71 ± 0.22	0.381	0.50 ± 0.19	0.548	1.04 ± 0.35	0.007	ENSMUST00000181449	predicted gene, 26611
Gm26797	0.65 ± 0.30	0.351	0.92 ± 0.32	0.011	1.95 ± 0.21	p < 0.001	ENSMUST00000180685	predicted gene, 26797
Gm26825	0.00 ± 0.07	1.000	-0.11 ± 0.06	0.823	-2.37 ± 0.07	p < 0.001	ENSMUST00000181751	predicted gene, 26825
Gm3150	0.20 ± 0.11	0.552	0.48 ± 0.05	p < 0.001	1.07 ± 0.08	p < 0.001	ENSMUST00000166418	predicted gene 3150
Gm4070	1.09 ± 0.16	p < 0.001	2.45 ± 0.15	p < 0.001	3.00 ± 0.17	p < 0.001	NM_001243039	predicted gene 4070
Gm4610	0.30 ± 0.49	1.000	0.21 ± 0.29	0.993	1.87 ± 0.27	p < 0.001	XR_168405	predicted gene 4610
Gm5086	-0.63 ± 0.23	0.111	-1.01 ± 0.24	p < 0.001	-1.15 ± 0.25	p < 0.001	NR_046157	predicted gene 5086
Gm5431	0.52 ± 0.60	0.724	1.76 ± 0.44	p < 0.001	0.50 ± 0.07	0.333	ENSMUST00000109212	predicted gene 5431
Gm5593	0.22 ± 0.19	0.642	-0.32 ± 0.28	0.088	-2.17 ± 0.50	p < 0.001	XM_485921	predicted gene 5593
Gm5620	0.04 ± 0.05	1.000	-0.17 ± 0.04	0.234	-1.67 ± 0.13	p < 0.001	ENSMUST00000077991	predicted gene 5620
Gm5801	0.14 ± 0.21	1.000	0.51 ± 0.16	0.059	1.10 ± 0.16	p < 0.001	NR_002889	ubiquitin-conjugating enzyme E2, J2 homolog pseudogene
Gm6104	0.22 ± 0.11	1.000	-0.07 ± 0.27	1.000	-1.29 ± 0.59	p < 0.001	ENSMUST00000078030	predicted gene 6104
Gm614	0.60 ± 0.34	0.273	0.13 ± 0.38	1.000	1.03 ± 0.35	p < 0.001	NM_001033362	predicted gene 614
Gm6329	-0.26 ± 0.06	0.214	0.07 ± 0.04	1.000	1.67 ± 0.11	p < 0.001	NR_040690	predicted gene 6329
Gm6377	0.43 ± 0.16	0.732	0.79 ± 0.15	0.012	2.36 ± 0.29	p < 0.001	NM_001037917	predicted gene 6377
Gm6548	0.59 ± 0.17	p < 0.001	1.05 ± 0.16	p < 0.001	1.68 ± 0.16	p < 0.001	NR_003363	eukaryotic translation elongation factor 1 alpha 1 pseudogene
Gm6594	0.08 ± 0.01	1.000	-0.06 ± 0.10	1.000	-1.04 ± 0.26	p < 0.001	ENSMUST00000097278	predicted pseudogene 6594
Gm6634	-0.37 ± 0.41	0.859	-0.01 ± 0.41	1.000	1.80 ± 0.41	p < 0.001	NR_040556	predicted gene 6634
Gm6682	-0.13 ± 0.13	0.988	-0.35 ± 0.10	0.007	-1.70 ± 0.15	p < 0.001	NR_033599	tubulin, alpha 1C pseudogene
Gm7072	0.19 ± 0.36	1.000	0.34 ± 0.32	0.287	1.18 ± 0.37	p < 0.001	XR_385455	predicted gene 7072
Gm7609	0.80 ± 0.24	p < 0.001	1.71 ± 0.23	p < 0.001	3.48 ± 0.23	p < 0.001	NM_001081746	predicted pseudogene 7609
Gm8094	0.19 ± 0.58	1.000	0.59 ± 0.73	0.325	1.05 ± 0.85	0.004	XM_006519825	predicted gene 8094
Gm8399	-0.40 ± 0.15	p < 0.001	-0.80 ± 0.13	p < 0.001	-1.75 ± 0.12	p < 0.001	ENSMUST00000082300	predicted gene 8399
Gm8979	1.30 ± 0.17	p < 0.001	2.54 ± 0.18	p < 0.001	3.12 ± 0.21	p < 0.001	NR_030719	very large inducible GTPase 1 pseudogene
Gm8995	1.23 ± 0.30	p < 0.001	2.55 ± 0.27	p < 0.001	3.09 ± 0.28	p < 0.001	XR_378400	predicted gene 8995

Gm9199	0.02 ± 0.10	1.000	0.08 ± 0.19	0.986	-1.03 ± 0.56	p < 0.001	NR_027860	glycine cleavage system protein H (aminomethyl carrier) pseudogene
Gm9320	0.40 ± 0.16	0.789	-0.01 ± 0.35	1.000	1.22 ± 0.19	p < 0.001	ENSMUST00000176549	predicted gene 9320
Gmeb2	0.26 ± 0.15	0.451	0.09 ± 0.04	1.000	1.01 ± 0.05	p < 0.001	NM_198169	glucocorticoid modulatory element binding protein 2
Gmnn	-0.13 ± 0.20	1.000	-0.16 ± 0.16	0.745	-1.67 ± 0.13	p < 0.001	NM_020567	geminin
Gnb1l	-0.05 ± 0.21	1.000	-0.42 ± 0.25	0.145	-1.00 ± 0.22	p < 0.001	NM_001081682	guanine nucleotide binding protein (G protein), beta polypeptide 1-like
Gnpda2	0.00 ± 0.14	1.000	0.24 ± 0.14	0.728	1.08 ± 0.13	p < 0.001	ENSMUST00000154018	glucosamine-6-phosphate deaminase 2
Gnpnat1	0.11 ± 0.15	1.000	0.37 ± 0.10	0.004	1.63 ± 0.11	p < 0.001	NM_019425	glucosamine-phosphate N-acetyltransferase 1
Gnptab	-0.41 ± 0.12	0.001	-0.60 ± 0.02	p < 0.001	-1.50 ± 0.02	p < 0.001	NM_001004164	N-acetylglucosamine-1-phosphate transferase, alpha and beta subunits
Gp49a	0.44 ± 0.07	p < 0.001	0.49 ± 0.03	p < 0.001	1.35 ± 0.04	p < 0.001	NM_001291892	glycoprotein 49 A
Gpatch4	-0.09 ± 0.14	1.000	-0.43 ± 0.18	0.001	-1.04 ± 0.22	p < 0.001	NM_001110809	G patch domain containing 4
Gpd1l	-0.13 ± 0.26	1.000	-0.17 ± 0.25	0.747	-1.46 ± 0.24	p < 0.001	NM_175380	glycerol-3-phosphate dehydrogenase 1-like
Gpr176	-0.33 ± 0.07	0.036	-0.89 ± 0.06	p < 0.001	-1.52 ± 0.11	p < 0.001	NM_201367	G protein-coupled receptor 176
Gpr180	-0.15 ± 0.08	1.000	-0.18 ± 0.04	0.769	-1.16 ± 0.05	p < 0.001	NM_021434	G protein-coupled receptor 180
Gpr183	-0.28 ± 0.16	0.081	-0.61 ± 0.08	p < 0.001	-1.51 ± 0.14	p < 0.001	NM_183031	G protein-coupled receptor 183
Gpr84	1.73 ± 0.06	p < 0.001	1.76 ± 0.06	p < 0.001	0.17 ± 0.21	0.389	NM_030720	G protein-coupled receptor 84
Gpr85	0.30 ± 0.09	1.000	0.29 ± 0.14	0.862	1.10 ± 0.36	p < 0.001	ENSMUST00000060442	G protein-coupled receptor 85
Gprc5a	0.42 ± 0.44	0.554	0.79 ± 0.36	0.001	2.99 ± 0.38	p < 0.001	NM_181444	G protein-coupled receptor, family C, group 5, member A
Gps1	-0.12 ± 0.05	1.000	-0.40 ± 0.07	0.001	-1.22 ± 0.10	p < 0.001	NM_001177874	G protein pathway suppressor 1
Gpsm2	0.09 ± 0.04	1.000	0.01 ± 0.04	1.000	-1.21 ± 0.05	p < 0.001	NM_029522	G-protein signalling modulator 2 (AGS3-like, C. elegans)
Grap	0.27 ± 0.06	0.343	0.52 ± 0.08	p < 0.001	2.27 ± 0.05	p < 0.001	NM_027817	GRB2-related adaptor protein
Grina	0.08 ± 0.16	1.000	0.31 ± 0.09	0.022	1.35 ± 0.14	p < 0.001	NM_023168	glutamate receptor, ionotropic, N-methyl D-aspartate-associated protein 1 (glutamate binding)
Grk6	0.03 ± 0.07	1.000	-0.11 ± 0.13	0.940	-1.43 ± 0.18	p < 0.001	NM_001112711	G protein-coupled receptor kinase 6
Grwd1	0.10 ± 0.16	1.000	-0.31 ± 0.13	0.126	-1.37 ± 0.15	p < 0.001	NM_153419	glutamate-rich WD repeat containing 1
Gsap	1.04 ± 0.00	p < 0.001	1.61 ± 0.02	p < 0.001	1.80 ± 0.06	p < 0.001	NM_175437	gamma-secretase activating protein
Gsg2	-0.12 ± 0.14	1.000	-0.39 ± 0.16	0.333	-1.58 ± 0.15	p < 0.001	NM_010353	germ cell-specific gene 2
Gsn	-0.11 ± 0.08	1.000	-0.19 ± 0.08	0.228	-1.51 ± 0.10	p < 0.001	NM_001206367	gelsolin
Gsr	-0.27 ± 0.12	0.070	-0.36 ± 0.08	0.002	-1.22 ± 0.14	p < 0.001	NM_010344	glutathione reductase
Gtpbp2	0.45 ± 0.22	0.023	1.12 ± 0.22	p < 0.001	2.57 ± 0.22	p < 0.001	NM_001145979	GTP binding protein 2
Gtpbp8	-0.07 ± 0.08	1.000	0.04 ± 0.13	1.000	1.05 ± 0.08	p < 0.001	NM_001159329	GTP-binding protein 8 (putative)
Gtse1	0.08 ± 0.11	1.000	-0.41 ± 0.15	0.001	-1.09 ± 0.09	p < 0.001	NM_001168672	G two S phase expressed protein 1
Gyg	-0.09 ± 0.05	1.000	0.08 ± 0.06	0.984	1.14 ± 0.08	p < 0.001	NM_013755	glycogenin
H2afx	-0.07 ± 0.07	1.000	-0.54 ± 0.07	p < 0.001	-2.80 ± 0.16	p < 0.001	NM_010436	H2A histone family, member X
H2-M3	0.12 ± 0.06	1.000	0.47 ± 0.07	p < 0.001	1.23 ± 0.09	p < 0.001	NM_013819	histocompatibility 2, M region locus 3
H2-Q4	0.39 ± 0.02	0.160	0.42 ± 0.16	0.045	1.51 ± 0.09	p < 0.001	NM_001143689	histocompatibility 2, Q region locus 4
H2-Q5	0.22 ± 0.23	0.334	0.43 ± 0.24	p < 0.001	1.58 ± 0.24	p < 0.001	ENSMUST00000172979	histocompatibility 2, Q region locus 5
H2-T22	0.25 ± 0.23	0.690	1.18 ± 0.21	p < 0.001	1.38 ± 0.17	p < 0.001	NM_010397	histocompatibility 2, T region locus 22
H2-T23	0.38 ± 0.14	0.643	1.23 ± 0.08	p < 0.001	2.19 ± 0.08	p < 0.001	NM_010398	histocompatibility 2, T region locus 23
H2-T24	1.39 ± 0.24	p < 0.001	3.11 ± 0.22	p < 0.001	3.06 ± 0.25	p < 0.001	NM_008207	histocompatibility 2, T region locus 24
H3f3b	0.18 ± 0.08	1.000	0.32 ± 0.13	0.280	1.12 ± 0.04	p < 0.001	NM_008211	H3 histone, family 3B
H60b	0.45 ± 0.12	0.133	0.32 ± 0.14	0.331	1.85 ± 0.12	p < 0.001	NM_001177775	histocompatibility 60b
Hadh	-0.11 ± 0.09	1.000	-0.40 ± 0.06	0.001	-1.44 ± 0.04	p < 0.001	NM_008212	hydroxyacyl-Coenzyme A dehydrogenase

Hat1	0.00 ± 0.04	1.000	0.02 ± 0.03	1.000	-1.09 ± 0.03	$p < 0.001$	NM_026115	histone aminotransferase 1
Haus4	0.04 ± 0.17	1.000	-0.17 ± 0.07	0.885	-1.55 ± 0.09	$p < 0.001$	NM_145462	HAUS augmin-like complex, subunit 4
Haus5	-0.16 ± 0.09	0.933	-0.49 ± 0.01	$p < 0.001$	-1.93 ± 0.35	$p < 0.001$	NM_027999	HAUS augmin-like complex, subunit 5
Haus8	-0.08 ± 0.05	1.000	-0.21 ± 0.08	0.401	-1.60 ± 0.10	$p < 0.001$	NM_001163042	4HAUS augmin-like complex, subunit 8
Havcr2	-0.53 ± 0.09	0.001	-1.42 ± 0.02	$p < 0.001$	0.25 ± 0.04	0.177	NM_134250	hepatitis A virus cellular receptor 2
Hbp1	-0.10 ± 0.07	1.000	0.24 ± 0.06	0.415	1.35 ± 0.11	$p < 0.001$	NM_177993	high mobility group box transcription factor 1
Hcar2	0.45 ± 0.35	0.639	1.45 ± 0.47	$p < 0.001$	1.77 ± 0.35	$p < 0.001$	NM_030701	hydroxycarboxylic acid receptor 2
Hck	0.70 ± 0.12	$p < 0.001$	1.67 ± 0.08	$p < 0.001$	2.11 ± 0.07	$p < 0.001$	NM_001172117	hemopoietic cell kinase
Hdac5	-0.25 ± 0.03	0.221	-0.60 ± 0.05	$p < 0.001$	-1.34 ± 0.11	$p < 0.001$	NM_001077696	histone deacetylase 5
Heatr2	0.06 ± 0.02	1.000	-0.13 ± 0.03	0.738	-1.31 ± 0.06	$p < 0.001$	NM_001081265	HEAT repeat containing 2
Heatr5a	-0.12 ± 0.10	1.000	-0.24 ± 0.14	0.196	-1.04 ± 0.06	$p < 0.001$	NM_177171	HEAT repeat containing 5A
Heatr9	-0.11 ± 0.33	1.000	-0.05 ± 0.11	1.000	1.60 ± 0.32	$p < 0.001$	NM_001045543	HEAT repeat containing 9
Hecw2	-0.01 ± 0.25	1.000	0.15 ± 0.27	1.000	1.78 ± 0.27	$p < 0.001$	NM_172655	HECT, C2 and WW domain containing E3 ubiquitin protein ligase 2
Hells	-0.15 ± 0.09	0.856	-0.42 ± 0.08	0.001	-1.67 ± 0.13	$p < 0.001$	NM_008234	helicase, lymphoid specific
Helz2	0.86 ± 0.20	$p < 0.001$	1.82 ± 0.21	$p < 0.001$	2.35 ± 0.22	$p < 0.001$	NM_183162	helicase with zinc finger 2, transcriptional coactivator
Herc6	0.22 ± 0.20	0.870	1.15 ± 0.19	$p < 0.001$	1.45 ± 0.19	$p < 0.001$	NM_025992	hect domain and RLD 6
Hexim1	0.10 ± 0.07	1.000	-0.36 ± 0.07	0.017	-1.17 ± 0.06	$p < 0.001$	NM_138753	hexamethylene bis-acetamide inducible 1
Hid1	0.14 ± 0.21	1.000	0.57 ± 0.24	0.144	1.31 ± 0.21	$p < 0.001$	NM_175454	HID1 domain containing
Hilpda	0.28 ± 0.19	0.588	0.79 ± 0.21	$p < 0.001$	1.30 ± 0.17	$p < 0.001$	NM_001190461	hypoxia inducible lipid droplet associated
Hirip3	-0.07 ± 0.16	1.000	-0.33 ± 0.09	0.093	-1.82 ± 0.10	$p < 0.001$	NM_172746	HIRA interacting protein 3
Hist1h1a	-0.03 ± 0.04	1.000	-0.18 ± 0.01	1.000	-1.24 ± 0.30	$p < 0.001$	NM_030609	histone cluster 1, H1a
Hist1h1b	-0.14 ± 0.23	0.980	-0.44 ± 0.23	$p < 0.001$	-3.44 ± 0.72	$p < 0.001$	NM_020034	histone cluster 1, H1b
Hist1h2ab	-0.29 ± 0.06	0.014	-0.60 ± 0.13	$p < 0.001$	-3.36 ± 0.09	$p < 0.001$	NM_175660	histone cluster 1, H2ab
Hist1h2af	-0.06 ± 0.04	1.000	-0.49 ± 0.05	$p < 0.001$	-2.23 ± 0.07	$p < 0.001$	NM_175661	histone cluster 1, H2af
Hist1h2ag	-0.26 ± 0.05	0.170	-1.02 ± 0.06	$p < 0.001$	-4.29 ± 0.07	$p < 0.001$	NM_178186	histone cluster 1, H2ag
Hist1h2an	-0.05 ± 0.04	1.000	-0.33 ± 0.08	$p < 0.001$	-1.51 ± 0.10	$p < 0.001$	NM_178184	histone cluster 1, H2an
Hist1h2ao	-0.12 ± 0.06	0.480	-0.25 ± 0.03	0.001	-1.21 ± 0.02	$p < 0.001$	NM_001177544	histone cluster 1, H2ao
Hist1h2bb	-0.23 ± 0.07	0.255	-0.36 ± 0.08	0.003	-2.15 ± 0.37	$p < 0.001$	NM_175664	histone cluster 1, H2bb
Hist1h2bg	0.10 ± 0.13	1.000	-0.83 ± 0.12	$p < 0.001$	-1.22 ± 0.15	$p < 0.001$	BC060304	histone cluster 1, H2bg
Hist1h2bh	-0.02 ± 0.14	1.000	-0.39 ± 0.16	0.507	-1.44 ± 0.16	$p < 0.001$	NM_178197	histone cluster 1, H2bh
Hist1h2bj	-0.48 ± 0.55	0.705	-1.20 ± 0.43	$p < 0.001$	-1.39 ± 0.43	$p < 0.001$	ENSMUST00000110452	histone cluster 1, H2bj
Hist1h2bk	-0.14 ± 0.04	0.920	-0.67 ± 0.08	$p < 0.001$	-3.99 ± 0.08	$p < 0.001$	NM_175665	histone cluster 1, H2bk
Hist1h2bl	-0.03 ± 0.03	1.000	-0.27 ± 0.05	$p < 0.001$	-1.94 ± 0.01	$p < 0.001$	NM_178199	histone cluster 1, H2bl
Hist1h2bm	-0.18 ± 0.14	1.000	-0.95 ± 0.06	0.004	-1.02 ± 0.29	0.001	BC139381	histone cluster 1, H2bm
Hist1h2bn	-0.11 ± 0.07	0.697	-0.30 ± 0.04	$p < 0.001$	-2.08 ± 0.05	$p < 0.001$	NM_178201	histone cluster 1, H2bn
Hist1h2bq	0.02 ± 0.10	1.000	-0.15 ± 0.11	1.000	-1.23 ± 0.07	$p < 0.001$	NM_001097979	histone cluster 1, H2bq
Hist1h3g	-0.23 ± 0.20	1.000	-1.21 ± 0.27	$p < 0.001$	-2.28 ± 0.29	$p < 0.001$	NM_145073	histone cluster 1, H3g
Hist1h4k	0.00 ± 0.07	1.000	-0.30 ± 0.07	$p < 0.001$	-1.01 ± 0.16	$p < 0.001$	NM_178211	histone cluster 1, H4k
Hist1h4m	-0.06 ± 0.06	1.000	-0.20 ± 0.06	0.776	-1.23 ± 0.20	$p < 0.001$	NM_001195421	histone cluster 1, H4m
Hist2h2ab	0.00 ± 0.07	1.000	-0.27 ± 0.02	0.055	-1.17 ± 0.04	$p < 0.001$	NM_178213	histone cluster 2, H2ab
Hist2h2ac	-0.07 ± 0.03	1.000	-0.30 ± 0.06	0.018	-1.57 ± 0.03	$p < 0.001$	ENSMUST00000090782	histone cluster 2, H2ac

Hist2h2bb	0.00 ± 0.06	1.000	-0.23 ± 0.06	0.189	-2.06 ± 0.09	p < 0.001	NM_175666	histone cluster 2, H2bb
Hist2h3c1	-0.22 ± 0.04	0.665	-0.43 ± 0.04	0.006	-1.11 ± 0.04	p < 0.001	ENSMUST00000090779	histone cluster 2, H3c1
Hist2h3c2	-0.44 ± 0.05	0.151	-1.47 ± 0.02	p < 0.001	-1.78 ± 0.43	p < 0.001	ENSMUST00000167403	histone cluster 2, H3c2
Hist4h4	-0.31 ± 0.05	0.026	-0.53 ± 0.02	p < 0.001	-1.90 ± 0.14	p < 0.001	NM_175652	histone cluster 4, H4
Hivep1	0.42 ± 0.18	0.077	0.69 ± 0.14	p < 0.001	1.69 ± 0.06	p < 0.001	NM_007772	human immunodeficiency virus type I enhancer binding protein 1
Hivep2	0.15 ± 0.20	1.000	0.24 ± 0.17	0.880	1.20 ± 0.18	p < 0.001	NM_010437	human immunodeficiency virus type I enhancer binding protein 2
Hjurp	0.06 ± 0.04	1.000	-0.20 ± 0.12	0.314	-1.06 ± 0.17	p < 0.001	NM_198652	Holliday junction recognition protein
Hmbs	-0.07 ± 0.10	1.000	-0.10 ± 0.12	0.943	-1.05 ± 0.13	p < 0.001	NM_001110251	hydroxymethylbilane synthase
Hmgb2	0.04 ± 0.10	1.000	-0.20 ± 0.05	0.655	-1.27 ± 0.05	p < 0.001	NM_008252	high mobility group box 2
Hmgcr	-0.27 ± 0.01	0.025	-0.51 ± 0.07	p < 0.001	-2.05 ± 0.11	p < 0.001	NM_008255	3-hydroxy-3-methylglutaryl-Coenzyme A reductase
Hmgcs1	-0.39 ± 0.05	p < 0.001	-0.68 ± 0.07	p < 0.001	-2.63 ± 0.12	p < 0.001	NM_001291439	3-hydroxy-3-methylglutaryl-Coenzyme A synthase 1
Hmgn2	0.21 ± 0.13	0.690	-0.06 ± 0.25	1.000	-1.02 ± 0.55	p < 0.001	ENSMUST00000075602	high mobility group nucleosomal binding domain 2
Hmmr	0.21 ± 0.06	0.642	-0.16 ± 0.07	0.650	-2.08 ± 0.20	p < 0.001	NM_013552	hyaluronan mediated motility receptor (RHAMM)
Hn1	-0.17 ± 0.07	0.682	-0.28 ± 0.17	0.060	-1.16 ± 0.07	p < 0.001	NM_008258	hematological and neurological expressed sequence 1
Hnrnpa0	-0.01 ± 0.10	1.000	-0.14 ± 0.10	0.641	-1.38 ± 0.12	p < 0.001	NM_029872	heterogeneous nuclear ribonucleoprotein A0
Hnrnpa2b1	0.03 ± 0.05	1.000	-0.03 ± 0.06	1.000	-1.23 ± 0.28	p < 0.001	NM_016806	heterogeneous nuclear ribonucleoprotein A2/B1
Hnrnpab	0.01 ± 0.02	1.000	-0.03 ± 0.02	1.000	-1.46 ± 0.08	p < 0.001	ENSMUST00000101249	heterogeneous nuclear ribonucleoprotein A/B
Hnrnpr	-0.06 ± 0.13	1.000	-0.27 ± 0.13	0.102	-1.17 ± 0.26	p < 0.001	NM_001277121	heterogeneous nuclear ribonucleoprotein R
Hnrnpu	-0.38 ± 0.25	0.058	-0.95 ± 0.12	p < 0.001	-1.92 ± 0.05	p < 0.001	XR_373612	heterogeneous nuclear ribonucleoprotein U
Hs3st3b1	0.26 ± 0.04	0.299	-0.06 ± 0.02	1.000	-1.10 ± 0.07	p < 0.001	NM_018805	heparan sulfate (glucosamine) 3-O-sulfotransferase 3B1
Hsd17b7	-0.15 ± 0.06	0.834	-0.34 ± 0.08	0.006	-1.91 ± 0.18	p < 0.001	NM_010476	hydroxysteroid (17-beta) dehydrogenase 7
Hsp90ab1	-0.10 ± 0.07	1.000	-0.10 ± 0.02	0.833	-1.46 ± 0.08	p < 0.001	NM_008302	heat shock protein 90 alpha (cytosolic), class B member 1
Hspa8	0.02 ± 0.04	1.000	-0.10 ± 0.02	0.673	-1.99 ± 0.06	p < 0.001	NM_031165	heat shock protein 8
Hspb7	-0.36 ± 0.11	0.058	-0.79 ± 0.16	p < 0.001	-1.16 ± 0.10	p < 0.001	NM_013868	heat shock protein family, member 7 (cardiovascular)
Hsph1	0.00 ± 0.05	1.000	-0.19 ± 0.10	0.327	-1.27 ± 0.08	p < 0.001	NM_013559	heat shock 105kDa/110kDa protein 1
Htr1b	-0.41 ± 0.02	0.004	-1.02 ± 0.04	p < 0.001	-2.00 ± 0.03	p < 0.001	ENSMUST00000051005	5-hydroxytryptamine (serotonin) receptor 1B
Htra2	-0.15 ± 0.03	0.616	-0.52 ± 0.09	p < 0.001	-1.19 ± 0.05	p < 0.001	NM_019752	HtrA serine peptidase 2
Hyal3	0.15 ± 0.18	1.000	0.49 ± 0.18	0.172	1.45 ± 0.25	p < 0.001	NM_178020	hyaluronoglucosaminidase 3
Hyls1	-0.11 ± 0.10	1.000	-0.17 ± 0.15	0.752	-1.26 ± 0.10	p < 0.001	NM_029762	hydrolethalus syndrome 1
I830077J02Rik	0.26 ± 0.19	0.173	0.59 ± 0.19	p < 0.001	1.70 ± 0.26	p < 0.001	NM_001033780	RIKEN cDNA I830077J02 gene
Icam1	0.47 ± 0.09	p < 0.001	0.63 ± 0.16	p < 0.001	1.35 ± 0.05	p < 0.001	NM_010493	intercellular adhesion molecule 1
Icosl	-0.07 ± 0.09	1.000	0.14 ± 0.10	1.000	2.92 ± 0.11	p < 0.001	NM_015790	icos ligand
Idh1	-0.09 ± 0.12	1.000	-0.12 ± 0.10	0.779	-1.10 ± 0.22	p < 0.001	NM_010497	isocitrate dehydrogenase 1 (NADP+), soluble
Idh3a	-0.09 ± 0.04	1.000	-0.15 ± 0.03	0.534	-1.07 ± 0.18	p < 0.001	NM_029573	isocitrate dehydrogenase 3 (NAD+) alpha
Idi1	-0.23 ± 0.08	0.391	-0.45 ± 0.05	p < 0.001	-2.35 ± 0.17	p < 0.001	NM_145360	isopentenyl-diphosphate delta isomerase
Ier2	0.52 ± 0.11	0.025	0.44 ± 0.09	0.045	1.48 ± 0.10	p < 0.001	NM_010499	immediate early response 2
Ier3	1.41 ± 0.13	p < 0.001	1.92 ± 0.13	p < 0.001	2.24 ± 0.12	p < 0.001	NM_133662	immediate early response 3
Ier5	0.16 ± 0.10	1.000	0.04 ± 0.11	1.000	1.76 ± 0.03	p < 0.001	NM_010500	immediate early response 5
Ifi202b	1.65 ± 0.16	p < 0.001	3.57 ± 0.21	p < 0.001	4.32 ± 0.16	p < 0.001	NM_008327	interferon activated gene 202B
Ifi203	0.83 ± 0.15	0.001	2.13 ± 0.10	p < 0.001	3.23 ± 0.05	p < 0.001	NM_008328	interferon activated gene 203
Ifi204	1.88 ± 0.50	p < 0.001	3.68 ± 0.48	p < 0.001	4.29 ± 0.52	p < 0.001	NM_008329	interferon activated gene 204

Ifi205	0.41 ± 0.56	0.873	0.93 ± 0.54	0.003	1.57 ± 0.54	$p < 0.001$	NM_172648	interferon activated gene 205
Ifi27l2a	0.48 ± 0.18	0.061	1.55 ± 0.22	$p < 0.001$	1.88 ± 0.19	$p < 0.001$	NM_029803	interferon, alpha-inducible protein 27 like 2A
Ifi30	-0.16 ± 0.04	0.431	-0.44 ± 0.07	$p < 0.001$	-1.57 ± 0.04	$p < 0.001$	NM_023065	interferon gamma inducible protein 30
Ifi35	0.66 ± 0.13	$p < 0.001$	1.43 ± 0.07	$p < 0.001$	0.95 ± 0.10	$p < 0.001$	NM_027320	interferon-induced protein 35
Ifi44	1.18 ± 0.09	$p < 0.001$	2.39 ± 0.05	$p < 0.001$	2.48 ± 0.07	$p < 0.001$	NM_133871	interferon-induced protein 44
Ifi44l	1.75 ± 0.17	$p < 0.001$	3.77 ± 0.17	$p < 0.001$	2.76 ± 0.17	$p < 0.001$	ENSMUST00000046739	interferon-induced protein 44 like
Ifih1	1.10 ± 0.25	$p < 0.001$	2.30 ± 0.25	$p < 0.001$	3.64 ± 0.25	$p < 0.001$	NM_001164477	interferon induced with helicase C domain 1
Ifit1	1.95 ± 0.34	$p < 0.001$	3.75 ± 0.34	$p < 0.001$	4.16 ± 0.36	$p < 0.001$	NM_008331	interferon-induced protein with tetratricopeptide repeats 1
Ifit2	0.00 ± 0.05	1.000	1.40 ± 0.11	$p < 0.001$	1.74 ± 0.17	$p < 0.001$	NM_008332	interferon-induced protein with tetratricopeptide repeats 2
Ifitm6	0.34 ± 0.15	0.081	1.16 ± 0.02	$p < 0.001$	2.12 ± 0.03	$p < 0.001$	NM_001033632	interferon induced transmembrane protein 6
Ifna7	0.24 ± 0.16	1.000	-0.57 ± 0.10	0.197	-1.03 ± 0.06	0.001	NM_008334	interferon alpha 7
Ifnz	-0.10 ± 0.52	1.000	0.25 ± 0.53	0.918	1.45 ± 0.57	$p < 0.001$	NM_197889	interferon zeta
Ifrd2	-0.06 ± 0.07	1.000	-0.20 ± 0.09	0.290	-1.23 ± 0.07	$p < 0.001$	NM_025903	interferon-related developmental regulator 2
Igf1	-0.44 ± 0.12	0.121	-1.03 ± 0.03	$p < 0.001$	-1.90 ± 0.04	$p < 0.001$	NM_001111274	insulin-like growth factor 1
Igf2bp3	0.02 ± 0.04	1.000	0.22 ± 0.03	0.941	1.34 ± 0.07	$p < 0.001$	NM_023670	insulin-like growth factor 2 mRNA binding protein 3
Ighj1	-0.12 ± 0.02	1.000	-0.02 ± 0.05	1.000	-1.35 ± 0.20	$p < 0.001$	ENSMUST00000103430	immunoglobulin heavy joining 1
Ighj2	-0.64 ± 0.12	0.399	-0.19 ± 0.03	1.000	-1.26 ± 0.51	$p < 0.001$	ENSMUST00000103429	immunoglobulin heavy joining 2
Ighm	-0.16 ± 0.08	0.664	-0.13 ± 0.08	0.682	-1.23 ± 0.09	$p < 0.001$	ENSMUST00000103426	immunoglobulin heavy constant mu
Igh-V7183	0.54 ± 0.42	0.681	0.11 ± 0.29	1.000	1.02 ± 0.79	0.005	ENSMUST00000103450	immunoglobulin heavy chain (V7183 family)
Ighv8-5	0.10 ± 0.44	1.000	0.37 ± 0.50	0.831	1.09 ± 0.35	0.004	ENSMUST00000103520	immunoglobulin heavy variable V8-5
Igtp	1.35 ± 0.46	$p < 0.001$	2.46 ± 0.46	$p < 0.001$	2.34 ± 0.48	$p < 0.001$	NM_018738	interferon gamma induced GTPase
Iigp1	0.47 ± 0.24	0.779	0.82 ± 0.24	0.033	1.30 ± 0.20	$p < 0.001$	NM_001146275	interferon inducible GTPase 1
Ikbke	0.97 ± 0.03	$p < 0.001$	1.85 ± 0.03	$p < 0.001$	2.70 ± 0.11	$p < 0.001$	NM_019777	inhibitor of kappaB kinase epsilon
Il10ra	0.71 ± 0.36	0.085	1.58 ± 0.36	$p < 0.001$	2.34 ± 0.36	$p < 0.001$	NM_008348	interleukin 10 receptor, alpha
Il13ra1	-0.16 ± 0.20	1.000	0.40 ± 0.21	0.007	1.79 ± 0.24	$p < 0.001$	NM_133990	interleukin 13 receptor, alpha 1
Il13ra2	0.57 ± 0.28	0.467	1.38 ± 0.26	$p < 0.001$	2.74 ± 0.30	$p < 0.001$	XM_006528707	interleukin 13 receptor, alpha 2
Il15	0.65 ± 0.48	0.247	1.46 ± 0.49	$p < 0.001$	3.45 ± 0.49	$p < 0.001$	NM_001254747	interleukin 15
Il18	0.30 ± 0.21	0.802	1.46 ± 0.14	$p < 0.001$	1.87 ± 0.14	$p < 0.001$	NM_008360	interleukin 18
Il1a	-0.15 ± 0.52	1.000	0.22 ± 0.51	0.986	3.29 ± 0.33	$p < 0.001$	NM_010554	interleukin 1 alpha
Il1b	-0.24 ± 0.34	1.000	0.09 ± 0.35	1.000	2.60 ± 0.60	$p < 0.001$	NM_008361	interleukin 1 beta
Il1r1	0.26 ± 0.40	1.000	0.10 ± 0.36	1.000	2.64 ± 0.40	$p < 0.001$	NM_001123382	interleukin 1 receptor, type I
Il1rn	0.35 ± 0.01	0.047	0.50 ± 0.10	$p < 0.001$	1.60 ± 0.03	$p < 0.001$	NM_001039701	interleukin 1 receptor antagonist
Il23a	0.21 ± 0.24	1.000	0.06 ± 0.18	1.000	3.70 ± 0.24	$p < 0.001$	NM_031252	interleukin 23, alpha subunit p19
Il2rg	0.21 ± 0.03	0.381	0.32 ± 0.02	0.012	1.25 ± 0.04	$p < 0.001$	NM_013563	interleukin 2 receptor, gamma chain
Il6	-0.05 ± 0.26	1.000	0.36 ± 0.26	0.830	4.62 ± 0.33	$p < 0.001$	NM_031168	interleukin 6
Il7r	-0.11 ± 0.07	1.000	0.02 ± 0.05	1.000	1.40 ± 0.08	$p < 0.001$	NM_008372	interleukin 7 receptor
Ilf2	0.14 ± 0.08	0.882	0.01 ± 0.12	1.000	-1.72 ± 0.05	$p < 0.001$	NM_026374	interleukin enhancer binding factor 2
Impdh2	0.00 ± 0.05	1.000	-0.18 ± 0.04	0.317	-1.10 ± 0.12	$p < 0.001$	NM_011830	inosine 5-phosphate dehydrogenase 2
Incenp	-0.10 ± 0.09	1.000	-0.32 ± 0.09	0.017	-2.09 ± 0.09	$p < 0.001$	NM_016692	inner centromere protein
Insig1	-0.34 ± 0.05	0.002	-0.77 ± 0.03	$p < 0.001$	-2.23 ± 0.14	$p < 0.001$	NM_153526	insulin induced gene 1
Ipo11	-0.07 ± 0.06	1.000	-0.19 ± 0.04	0.386	-1.03 ± 0.07	$p < 0.001$	NM_029665	importin 11

Ippk	-0.12	±	0.09	1.000	-0.44	±	0.14	0.171	-1.24	±	0.09	$p < 0.001$	NM_001276399	inositol 1,3,4,5,6-pentakisphosphate 2-kinase
Iqgap3	0.03	±	0.11	1.000	-0.22	±	0.10	0.255	-1.81	±	0.15	$p < 0.001$	NM_001033484	IQ motif containing GTPase activating protein 3
Irak2	0.62	±	0.17	$p < 0.001$	1.04	±	0.17	$p < 0.001$	1.53	±	0.16	$p < 0.001$	NM_172161	interleukin-1 receptor-associated kinase 2
Irak3	0.31	±	0.07	0.490	0.64	±	0.11	$p < 0.001$	2.12	±	0.14	$p < 0.001$	NM_028679	interleukin-1 receptor-associated kinase 3
Irf7	1.38	±	0.18	$p < 0.001$	3.07	±	0.16	$p < 0.001$	3.77	±	0.16	$p < 0.001$	NM_001252600	interferon regulatory factor 7
Irf9	0.94	±	0.05	$p < 0.001$	1.27	±	0.06	$p < 0.001$	1.70	±	0.05	$p < 0.001$	NM_001159417	interferon regulatory factor 9
Irg1	1.79	±	0.40	$p < 0.001$	3.81	±	0.41	$p < 0.001$	6.92	±	0.40	$p < 0.001$	NM_008392	immunoresponsive gene 1
Irgm1	1.27	±	0.19	$p < 0.001$	1.82	±	0.17	$p < 0.001$	1.50	±	0.17	$p < 0.001$	NM_008326	immunity-related GTPase family M member 1
Irgm2	1.27	±	0.20	$p < 0.001$	2.26	±	0.19	$p < 0.001$	2.66	±	0.21	$p < 0.001$	NM_019440	immunity-related GTPase family M member 2
Isg15	1.47	±	0.28	$p < 0.001$	2.94	±	0.26	$p < 0.001$	3.23	±	0.28	$p < 0.001$	NM_015783	ISG15 ubiquitin-like modifier
Isg20	0.59	±	0.29	0.107	1.99	±	0.31	$p < 0.001$	2.75	±	0.28	$p < 0.001$	NM_001291220	interferon-stimulated protein
Isyna1	-0.18	±	0.04	0.425	-0.54	±	0.02	$p < 0.001$	-1.89	±	0.19	$p < 0.001$	NM_023627	myo-inositol 1-phosphate synthase A1
Itgam	0.11	±	0.07	1.000	0.72	±	0.09	$p < 0.001$	1.31	±	0.03	$p < 0.001$	NM_001082960	integrin alpha M
Itgb7	0.05	±	0.03	1.000	0.08	±	0.05	1.000	1.35	±	0.06	$p < 0.001$	NM_013566	integrin beta 7
Itpr2	0.21	±	0.14	0.998	0.62	±	0.09	0.001	1.26	±	0.09	$p < 0.001$	NM_010586	inositol 1,4,5-triphosphate receptor 2
Itpr3	-0.07	±	0.02	1.000	-0.11	±	0.12	0.841	-1.03	±	0.07	$p < 0.001$	NM_080553	inositol 1,4,5-triphosphate receptor 3
Itpripl1	0.08	±	0.09	1.000	-0.20	±	0.09	0.333	-1.74	±	0.08	$p < 0.001$	NM_001163527	inositol 1,4,5-triphosphate receptor interacting protein-like 1
Jade1	-0.25	±	0.11	0.607	-0.29	±	0.12	0.218	-1.41	±	0.16	$p < 0.001$	NM_001130184	jade family PHD finger 1
Jak2	0.36	±	0.03	0.030	0.88	±	0.06	$p < 0.001$	2.21	±	0.13	$p < 0.001$	NM_001048177	Janus kinase 2
Jun	-0.05	±	0.06	1.000	-0.08	±	0.03	0.977	1.69	±	0.06	$p < 0.001$	NM_010591	jun proto-oncogene
Junb	0.64	±	0.11	$p < 0.001$	0.88	±	0.06	$p < 0.001$	1.96	±	0.12	$p < 0.001$	NM_008416	jun B proto-oncogene
Kank2	-0.09	±	0.17	1.000	-0.56	±	0.07	0.026	-1.20	±	0.20	$p < 0.001$	NM_145611	KN motif and ankyrin repeat domains 2
Kat2a	0.04	±	0.07	1.000	-0.10	±	0.07	0.918	-1.40	±	0.14	$p < 0.001$	NM_001038010	K(lysine) acetyltransferase 2A
Kcnab2	-0.18	±	0.06	0.774	-0.34	±	0.05	0.016	-1.18	±	0.05	$p < 0.001$	NM_001252654	potassium voltage-gated channel, shaker-related subfamily, beta member 2
Kcnn4	0.09	±	0.05	1.000	-0.28	±	0.08	0.030	-2.03	±	0.08	$p < 0.001$	NM_001163510	potassium intermediate/small conductance calcium-activated channel, subfamily N, member 4
Kctd6	-0.08	±	0.23	1.000	0.36	±	0.17	0.531	1.41	±	0.37	$p < 0.001$	NM_027782	potassium channel tetramerisation domain containing 6
Kdm5b	0.22	±	0.11	1.000	0.95	±	0.07	$p < 0.001$	1.95	±	0.07	$p < 0.001$	NM_152895	lysine (K)-specific demethylase 5B
Kdm6b	1.23	±	0.16	$p < 0.001$	1.14	±	0.23	$p < 0.001$	2.62	±	0.14	$p < 0.001$	NM_001017426	KDM1 lysine (K)-specific demethylase 6B
Kdm7a	0.40	±	0.13	0.241	0.85	±	0.13	$p < 0.001$	1.60	±	0.13	$p < 0.001$	NM_001033430	lysine (K)-specific demethylase 7A
Khdrbs1	-0.15	±	0.02	0.988	-0.09	±	0.05	0.958	-1.03	±	0.21	$p < 0.001$	NM_011317	KH domain containing, RNA binding, signal transduction associated 1
Khsrp	-0.04	±	0.05	1.000	-0.09	±	0.09	0.922	-1.04	±	0.06	$p < 0.001$	NM_010613	KH-type splicing regulatory protein
Kif11	-0.09	±	0.05	1.000	-0.46	±	0.06	$p < 0.001$	-2.11	±	0.19	$p < 0.001$	NM_010615	kinesin family member 11
Kif14	0.14	±	0.02	1.000	-0.16	±	0.09	0.621	-2.11	±	0.20	$p < 0.001$	NM_001287179	kinesin family member 14
Kif15	0.02	±	0.02	1.000	-0.37	±	0.04	0.007	-2.03	±	0.12	$p < 0.001$	NM_010620	kinesin family member 15
Kif18a	0.21	±	0.03	0.595	-0.08	±	0.06	0.992	-1.43	±	0.20	$p < 0.001$	NM_139303	kinesin family member 18A
Kif18b	0.06	±	0.11	1.000	-0.42	±	0.08	0.001	-1.27	±	0.12	$p < 0.001$	BC049272	kinesin family member 18B
Kif20a	0.10	±	0.04	1.000	-0.45	±	0.05	$p < 0.001$	-3.30	±	0.25	$p < 0.001$	NM_001166406	kinesin family member 20A
Kif20b	0.03	±	0.05	1.000	-0.44	±	0.04	$p < 0.001$	-2.23	±	0.05	$p < 0.001$	NM_183046	kinesin family member 20B
Kif22	-0.01	±	0.13	1.000	-0.12	±	0.14	0.819	-1.65	±	0.11	$p < 0.001$	NM_145588	kinesin family member 22
Kif23	0.11	±	0.08	1.000	-0.18	±	0.09	0.362	-2.15	±	0.15	$p < 0.001$	NM_024245	kinesin family member 23
Kif24	-0.14	±	0.12	1.000	-0.18	±	0.05	0.824	-1.49	±	0.11	$p < 0.001$	NM_024241	kinesin family member 24

Kif2c	0.22 ± 0.09	0.290	-0.37 ± 0.04	0.003	-2.82 ± 0.12	$p < 0.001$	NM_001290662	kinesin family member 2C
Kif4	-0.08 ± 0.23	1.000	-0.34 ± 0.06	0.015	-1.42 ± 0.18	$p < 0.001$	NM_008446	kinesin family member 4
Klc4	-0.33 ± 0.09	0.163	-0.47 ± 0.15	0.003	-1.14 ± 0.07	$p < 0.001$	NM_029091	kinesin light chain 4
Klf10	0.14 ± 0.08	1.000	0.36 ± 0.10	0.094	1.36 ± 0.12	$p < 0.001$	NM_013692	Kruppel-like factor 10
Klf3	0.06 ± 0.11	1.000	0.54 ± 0.32	0.226	1.19 ± 0.11	$p < 0.001$	NM_008453	Kruppel-like factor 3 (basic)
Klf6	0.09 ± 0.18	1.000	0.30 ± 0.15	0.059	1.74 ± 0.16	$p < 0.001$	NM_011803	Kruppel-like factor 6
Klhl24	-0.18 ± 0.13	1.000	0.67 ± 0.09	0.001	1.86 ± 0.12	$p < 0.001$	NM_029436	kelch-like 24
Klhl38	-0.07 ± 0.17	1.000	0.01 ± 0.36	1.000	1.23 ± 0.09	$p < 0.001$	NM_177755	kelch-like 38
Klhl6	-0.64 ± 0.14	$p < 0.001$	-0.89 ± 0.14	$p < 0.001$	-1.31 ± 0.37	$p < 0.001$	NM_183390	kelch-like 6
Klk1b27	-1.07 ± 0.42	0.029	-0.92 ± 0.43	0.039	-0.36 ± 0.70	0.598	NM_020268	kallikrein 1-related peptidase b27
Klra2	0.03 ± 0.20	1.000	0.32 ± 0.29	0.862	1.34 ± 0.20	$p < 0.001$	NM_001170851	killer cell lectin-like receptor, subfamily A, member 2
Klrg2	-0.17 ± 0.08	1.000	-0.43 ± 0.07	0.041	-1.41 ± 0.07	$p < 0.001$	NM_001033171	killer cell lectin-like receptor subfamily G, member 2
Knstrn	0.08 ± 0.08	1.000	-0.21 ± 0.05	0.271	-1.21 ± 0.05	$p < 0.001$	XR_374487	kinetochore-localized astrin/SPAG5 binding
Kpna2	0.02 ± 0.01	1.000	-0.25 ± 0.02	0.115	-1.82 ± 0.01	$p < 0.001$	NM_010655	karyopherin (importin) alpha 2
Kpnb1	-0.10 ± 0.02	1.000	-0.24 ± 0.04	0.064	-1.19 ± 0.06	$p < 0.001$	NM_008379	karyopherin (importin) beta 1
Kti12	-0.11 ± 0.07	1.000	-0.40 ± 0.04	0.002	-1.28 ± 0.03	$p < 0.001$	NM_029571	KTI12 homolog, chromatin associated (S. cerevisiae)
Ktn1	0.14 ± 0.04	1.000	0.22 ± 0.09	0.367	1.17 ± 0.14	$p < 0.001$	NM_001293635	kinectin 1
L3mbtl2	0.06 ± 0.12	1.000	-0.35 ± 0.08	0.011	-1.23 ± 0.23	$p < 0.001$	NM_001289711	l(3)mbt-like 2 (Drosophila)
Lamtor3	0.03 ± 0.10	1.000	0.05 ± 0.08	1.000	1.02 ± 0.13	$p < 0.001$	NM_019920	late endosomal/lysosomal adaptor, MAPK and MTOR activator 3
Laptm4b	0.53 ± 0.47	0.637	0.19 ± 0.46	1.000	1.94 ± 0.18	$p < 0.001$	NM_033521	lysosomal-associated protein transmembrane 4B
Layn	-0.32 ± 0.05	0.127	-0.33 ± 0.13	0.038	-1.67 ± 0.26	$p < 0.001$	NM_001033534	layilin
Lbr	-0.02 ± 0.05	1.000	-0.06 ± 0.05	1.000	-1.60 ± 0.06	$p < 0.001$	NM_133815	lamin B receptor
Lcn2	-0.56 ± 0.07	0.027	0.01 ± 0.18	1.000	4.04 ± 0.09	$p < 0.001$	NM_008491	lipocalin 2
Lcorl	0.19 ± 0.08	1.000	0.48 ± 0.25	0.033	1.17 ± 0.08	$p < 0.001$	NM_001163073	ligand dependent nuclear receptor corepressor-like
Lcp2	0.63 ± 0.06	$p < 0.001$	1.01 ± 0.04	$p < 0.001$	1.40 ± 0.04	$p < 0.001$	AK088552	lymphocyte cytosolic protein 2
Ldb1	-0.04 ± 0.02	1.000	-0.19 ± 0.09	0.333	-1.05 ± 0.02	$p < 0.001$	NM_001113408	LIM domain binding 1
Ldlr	0.02 ± 0.10	1.000	-0.05 ± 0.09	1.000	-1.97 ± 0.10	$p < 0.001$	NM_001252658	low density lipoprotein receptor
Leprel1	0.32 ± 0.21	1.000	0.59 ± 0.13	0.221	2.87 ± 0.25	$p < 0.001$	NM_173379	leprecan-like 1
Leprotl1	0.13 ± 0.12	0.908	0.36 ± 0.13	0.001	1.26 ± 0.12	$p < 0.001$	NM_026609	leptin receptor overlapping transcript-like 1
Letm2	0.11 ± 0.17	1.000	0.61 ± 0.04	0.101	2.21 ± 0.14	$p < 0.001$	NM_173012	leucine zipper-EF-hand containing transmembrane protein 2
Lfng	-0.09 ± 0.05	1.000	-0.33 ± 0.05	0.044	-1.74 ± 0.10	$p < 0.001$	NM_008494	LFNG O-fucosylpeptide 3-beta-N-acetylglucosaminyltransferase
Lgals3bp	0.45 ± 0.11	$p < 0.001$	0.98 ± 0.08	$p < 0.001$	1.18 ± 0.10	$p < 0.001$	NM_011150	lectin, galactoside-binding, soluble, 3 binding protein
Lgals8	0.25 ± 0.11	0.175	0.56 ± 0.07	$p < 0.001$	1.30 ± 0.12	$p < 0.001$	NM_001199043	lectin, galactose binding, soluble 8
Lgals9	0.56 ± 0.07	$p < 0.001$	1.88 ± 0.11	$p < 0.001$	2.64 ± 0.09	$p < 0.001$	NM_001159301	lectin, galactose binding, soluble 9
Lig1	-0.17 ± 0.09	0.774	-0.28 ± 0.09	0.070	-1.32 ± 0.08	$p < 0.001$	NM_001083188	ligase I, DNA, ATP-dependent
Lims2	-0.04 ± 0.11	1.000	-0.26 ± 0.15	0.293	1.71 ± 0.07	$p < 0.001$	NM_144862	LIM and senescent cell antigen like domains 2
Lipa	-0.33 ± 0.05	$p < 0.001$	-0.54 ± 0.04	$p < 0.001$	-1.07 ± 0.08	$p < 0.001$	NM_001111100	lysosomal acid lipase A
Lix1	0.46 ± 0.23	0.737	0.90 ± 0.18	0.007	1.93 ± 0.18	$p < 0.001$	BC063057	limb expression 1 homolog (chicken)
Lmbrd1	0.05 ± 0.00	1.000	0.27 ± 0.05	0.100	1.35 ± 0.12	$p < 0.001$	NM_026719	LMBR1 domain containing 1
Lmcd1	-0.23 ± 0.02	0.062	-1.17 ± 0.05	$p < 0.001$	-4.12 ± 0.13	$p < 0.001$	NM_144799	LIM and cysteine-rich domains 1
Lmln	-0.06 ± 0.13	1.000	-0.12 ± 0.22	1.000	-1.28 ± 0.13	$p < 0.001$	NM_172823	leishmanolysin-like (metallopeptidase M8 family)

Lmna	-0.13 ± 0.03	0.988	-0.37 ± 0.07	0.003	-1.33 ± 0.04	$p < 0.001$	NM_001002011	lamin A
Lmnb1	0.10 ± 0.05	1.000	-0.17 ± 0.13	0.464	-1.83 ± 0.15	$p < 0.001$	NM_010721	lamin B1
Lmnb2	-0.05 ± 0.17	1.000	-0.26 ± 0.17	0.505	-1.27 ± 0.17	$p < 0.001$	NM_010722	lamin B2
Lnx2	-0.15 ± 0.10	1.000	-0.03 ± 0.07	1.000	1.34 ± 0.10	$p < 0.001$	NM_080795	ligand of numb-protein X 2
LOC100038947	0.24 ± 0.42	1.000	1.15 ± 0.42	0.002	0.11 ± 0.56	0.947	ENSMUST00000108349	signal-regulatory protein beta 1-like
LOC100041057	0.05 ± 0.49	1.000	0.35 ± 0.40	0.075	1.26 ± 0.34	$p < 0.001$	ENSMUST00000111360	nuclear body protein SP140-like
LOC100041708	0.26 ± 0.36	1.000	0.73 ± 0.13	0.071	1.33 ± 0.48	$p < 0.001$	XM_006535862	nuclear body protein SP140-like
LOC101055855	0.18 ± 0.40	1.000	0.41 ± 0.23	0.670	1.12 ± 0.23	0.001	XM_006508663	keratin-associated protein 5-5-like
LOC102631744	0.24 ± 0.22	1.000	-0.01 ± 0.19	1.000	1.04 ± 0.18	0.005	XM_006519861	disks large homolog 5-like
LOC102631833	0.30 ± 0.12	0.997	0.49 ± 0.12	0.159	1.09 ± 0.20	$p < 0.001$	XR_400210	uncharacterized LOC102631833
LOC102633071	0.15 ± 0.25	1.000	0.34 ± 0.11	0.605	1.66 ± 0.39	$p < 0.001$	XR_400342	uncharacterized LOC102633071
LOC102634459	-0.35 ± 0.34	1.000	-0.19 ± 0.40	1.000	1.13 ± 0.22	$p < 0.001$	XR_387206	uncharacterized LOC102634459
LOC102634822	0.02 ± 0.13	1.000	0.18 ± 0.10	0.996	1.18 ± 0.22	$p < 0.001$	XR_390079	uncharacterized LOC102634822
LOC102634900	0.08 ± 0.32	1.000	0.24 ± 0.28	0.922	1.01 ± 0.19	$p < 0.001$	XR_400650	uncharacterized LOC102634900
LOC102639005	0.09 ± 0.38	1.000	0.21 ± 0.07	0.954	1.85 ± 0.20	$p < 0.001$	XR_379290	uncharacterized LOC102639005
LOC102640811	-0.15 ± 0.21	1.000	-0.14 ± 0.09	1.000	1.59 ± 0.37	$p < 0.001$	XR_388974	uncharacterized LOC102640811
LOC102641139	-0.17 ± 0.18	0.956	-0.60 ± 0.11	$p < 0.001$	-1.62 ± 0.08	$p < 0.001$	XR_406654	uncharacterized LOC102641139
LOC664787	0.12 ± 0.16	1.000	0.57 ± 0.06	0.236	1.06 ± 0.15	0.001	ENSMUST00000111423	Sp110 nuclear body protein-like
LOC677525	0.10 ± 0.05	1.000	0.62 ± 0.11	$p < 0.001$	1.07 ± 0.09	$p < 0.001$	XM_006535876	sp110 nuclear body protein-like
Loxl3	-0.25 ± 0.14	0.221	-0.88 ± 0.12	$p < 0.001$	-1.32 ± 0.21	$p < 0.001$	NM_013586	lysyl oxidase-like 3
Lpar5	0.01 ± 0.11	1.000	-0.33 ± 0.05	0.097	-1.74 ± 0.10	$p < 0.001$	NM_001163268	lysophosphatidic acid receptor 5
Lpcat1	-0.12 ± 0.11	1.000	-0.47 ± 0.20	$p < 0.001$	-1.71 ± 0.12	$p < 0.001$	NM_145376	lysophosphatidylcholine acyltransferase 1
Lpcat4	0.15 ± 0.20	1.000	0.05 ± 0.13	1.000	-1.01 ± 0.23	$p < 0.001$	NM_207206	lysophosphatidylcholine acyltransferase 4
Lphn1	-0.18 ± 0.34	1.000	-0.36 ± 0.33	0.444	-1.16 ± 0.53	$p < 0.001$	NM_181039	latrophilin 1
Lphn2	-0.05 ± 0.18	1.000	0.39 ± 0.24	0.485	1.75 ± 0.32	$p < 0.001$	NM_001081298	latrophilin 2
Lpp	-0.05 ± 0.19	1.000	0.41 ± 0.17	0.105	1.71 ± 0.14	$p < 0.001$	NM_001145952	LIM domain containing preferred translocation partner in lipoma
Lpxn	0.04 ± 0.10	1.000	0.23 ± 0.10	0.113	1.20 ± 0.13	$p < 0.001$	NM_134152	leupaxin
Lrig2	-0.03 ± 0.20	1.000	0.18 ± 0.16	0.928	1.08 ± 0.18	$p < 0.001$	NM_001025067	leucine-rich repeats and immunoglobulin-like domains 2
Lrp5	0.10 ± 0.08	1.000	-0.03 ± 0.07	1.000	-1.39 ± 0.18	$p < 0.001$	NM_008513	low density lipoprotein receptor-related protein 5
Lrp8	-0.18 ± 0.03	0.627	-0.49 ± 0.09	$p < 0.001$	-2.19 ± 0.07	$p < 0.001$	NM_001080926	low density lipoprotein receptor-related protein 8, apolipoprotein e receptor
Lrr1	-0.31 ± 0.12	0.920	-0.70 ± 0.07	0.013	-1.91 ± 0.12	$p < 0.001$	NM_001081406	leucine rich repeat protein 1
Lrrc25	0.59 ± 0.21	0.007	1.30 ± 0.07	$p < 0.001$	1.61 ± 0.06	$p < 0.001$	NM_153074	leucine rich repeat containing 25
Lrrc32	-0.01 ± 0.26	1.000	0.05 ± 0.35	1.000	1.57 ± 0.44	$p < 0.001$	NM_001113379	leucine rich repeat containing 32
Lrrc39	-0.55 ± 0.16	0.014	-1.08 ± 0.15	$p < 0.001$	-1.43 ± 0.14	$p < 0.001$	NM_175413	leucine rich repeat containing 39
Lrrc45	-0.02 ± 0.08	1.000	-0.20 ± 0.07	0.786	-1.00 ± 0.11	$p < 0.001$	NM_153545	leucine rich repeat containing 45
Lrrc51	-0.05 ± 0.29	1.000	0.26 ± 0.11	0.785	1.11 ± 0.52	$p < 0.001$	NM_001162973	leucine rich repeat containing 51
Lrrc63	0.31 ± 0.11	1.000	0.40 ± 0.29	0.705	1.06 ± 0.31	0.003	NM_027581	leucine rich repeat containing 63
Lrrfip2	0.35 ± 0.05	0.075	0.65 ± 0.08	$p < 0.001$	1.20 ± 0.07	$p < 0.001$	NM_001164838	leucine rich repeat (in FLII) interacting protein 2
Lsm2	-0.11 ± 0.14	1.000	-0.53 ± 0.14	$p < 0.001$	-1.54 ± 0.15	$p < 0.001$	NM_001110101	LSM2 homolog, U6 small nuclear RNA associated (S. cerevisiae)
Lsm7	-0.09 ± 0.05	1.000	-0.20 ± 0.06	0.318	-1.49 ± 0.09	$p < 0.001$	NM_025349	LSM7 homolog, U6 small nuclear RNA associated (S. cerevisiae)
Lsp1	-0.26 ± 0.04	0.182	-0.67 ± 0.05	$p < 0.001$	-1.56 ± 0.05	$p < 0.001$	NM_001136071	lymphocyte specific 1

Lss	-0.10 ± 0.11	1.000	-0.24 ± 0.06	0.126	-1.73 ± 0.14	$p < 0.001$	XM_006513283	lanosterol synthase
Lst1	0.26 ± 0.05	0.874	0.98 ± 0.05	$p < 0.001$	1.56 ± 0.03	$p < 0.001$	NM_010734	leukocyte specific transcript 1
Ltb	1.40 ± 0.18	$p < 0.001$	2.16 ± 0.19	$p < 0.001$	1.75 ± 0.20	$p < 0.001$	NM_008518	lymphotoxin B
Lyar	-0.03 ± 0.08	1.000	-0.35 ± 0.08	0.011	-1.20 ± 0.32	$p < 0.001$	NM_025281	Ly1 antibody reactive clone
Lyrm1	0.08 ± 0.25	1.000	0.37 ± 0.18	0.538	1.23 ± 0.19	$p < 0.001$	NM_001285959	LYR motif containing 1
Lyrm4	0.14 ± 0.20	1.000	0.16 ± 0.11	0.613	1.47 ± 0.09	$p < 0.001$	NM_201358	LYR motif containing 4
Lysmd3	0.23 ± 0.25	0.910	0.44 ± 0.08	0.035	1.17 ± 0.09	$p < 0.001$	NM_030257	LysM, putative peptidoglycan-binding, domain containing 3
Lyst	0.16 ± 0.05	1.000	0.64 ± 0.06	$p < 0.001$	1.66 ± 0.04	$p < 0.001$	NM_010748	lysosomal trafficking regulator
Mad1l1	0.05 ± 0.04	1.000	-0.23 ± 0.03	0.252	-1.10 ± 0.07	$p < 0.001$	NM_010752	MAD1 mitotic arrest deficient 1-like 1
Man1c1	-0.10 ± 0.10	1.000	-0.40 ± 0.09	$p < 0.001$	-1.31 ± 0.09	$p < 0.001$	NM_207237	mannosidase, alpha, class 1C, member 1
Manbal	-0.02 ± 0.06	1.000	0.02 ± 0.08	1.000	1.18 ± 0.01	$p < 0.001$	NM_026968	mannosidase, beta A, lysosomal-like
Map3k2	0.03 ± 0.12	1.000	0.35 ± 0.08	0.018	1.47 ± 0.02	$p < 0.001$	NM_011946	mitogen-activated protein kinase kinase kinase 2
Map3k8	0.72 ± 0.23	0.004	0.86 ± 0.21	$p < 0.001$	1.17 ± 0.21	$p < 0.001$	NM_007746	mitogen-activated protein kinase kinase kinase 8
Map4k4	0.22 ± 0.11	0.399	0.40 ± 0.03	0.001	1.03 ± 0.07	$p < 0.001$	NM_001252200	mitogen-activated protein kinase kinase kinase kinase 4
March11	0.12 ± 0.51	1.000	0.45 ± 0.64	0.454	1.41 ± 0.48	$p < 0.001$	NM_177597	membrane-associated ring finger (C3HC4) 11
Marcks	-0.07 ± 0.08	1.000	-0.04 ± 0.08	1.000	1.04 ± 0.06	$p < 0.001$	NM_008538	myristoylated alanine rich protein kinase C substrate
Marcksl1	0.48 ± 0.16	0.003	0.87 ± 0.13	$p < 0.001$	1.35 ± 0.31	$p < 0.001$	NM_010807	MARCKS-like 1
Mastl	-0.15 ± 0.06	1.000	-0.21 ± 0.10	0.392	-1.71 ± 0.10	$p < 0.001$	NM_025979	microtubule associated serine/threonine kinase-like
Mat2a	-0.07 ± 0.02	1.000	-0.57 ± 0.07	$p < 0.001$	-1.67 ± 0.25	$p < 0.001$	NM_145569	methionine adenosyltransferase II, alpha
Mboat1	-0.13 ± 0.10	1.000	-0.42 ± 0.17	0.001	-2.30 ± 0.24	$p < 0.001$	NM_153546	membrane bound O-acyltransferase domain containing 1
Mcm10	-0.14 ± 0.04	0.962	-0.44 ± 0.04	$p < 0.001$	-1.12 ± 0.15	$p < 0.001$	NM_027290	minichromosome maintenance deficient 10 (S. cerevisiae)
Mcm2	-0.11 ± 0.10	1.000	-0.26 ± 0.10	0.066	-1.71 ± 0.12	$p < 0.001$	NM_008564	minichromosome maintenance deficient 2 mitotin (S. cerevisiae)
Mcm3	-0.13 ± 0.05	0.893	-0.25 ± 0.05	0.067	-1.45 ± 0.14	$p < 0.001$	NM_008563	minichromosome maintenance deficient 3 (S. cerevisiae)
Mcm4	-0.21 ± 0.09	0.319	-0.38 ± 0.07	0.001	-1.99 ± 0.07	$p < 0.001$	NM_008565	minichromosome maintenance deficient 4 homolog (S. cerevisiae)
Mcm5	-0.20 ± 0.03	0.354	-0.37 ± 0.05	0.001	-1.67 ± 0.09	$p < 0.001$	NM_008566	minichromosome maintenance deficient 5, cell division cycle 46 (S. cerevisiae)
Mcm6	-0.18 ± 0.08	0.523	-0.18 ± 0.10	0.326	-2.38 ± 0.29	$p < 0.001$	NM_008567	minichromosome maintenance deficient 6 (MIS5 homolog, S. pombe) (S. cerevisiae)
Mcm7	-0.27 ± 0.02	0.093	-0.43 ± 0.03	$p < 0.001$	-2.08 ± 0.03	$p < 0.001$	NM_008568	minichromosome maintenance deficient 7 (S. cerevisiae)
Mcm8	-0.15 ± 0.19	1.000	-0.10 ± 0.18	0.972	-1.11 ± 0.23	$p < 0.001$	NM_001291054	minichromosome maintenance deficient 8 (S. cerevisiae)
Mcm9	0.07 ± 0.17	1.000	0.07 ± 0.09	1.000	1.44 ± 0.16	$p < 0.001$	NM_027830	minichromosome maintenance complex component 9
Mcmbp	-0.09 ± 0.04	1.000	-0.08 ± 0.04	0.954	-1.27 ± 0.04	$p < 0.001$	NM_145955	MCM (minichromosome maintenance deficient) binding protein
Mdc1	-0.05 ± 0.07	1.000	-0.06 ± 0.09	1.000	-1.07 ± 0.10	$p < 0.001$	NM_001010833	mediator of DNA damage checkpoint 1
Mdm2	-0.03 ± 0.07	1.000	0.14 ± 0.13	0.696	1.19 ± 0.03	$p < 0.001$	NM_001288586	transformed mouse 3T3 cell double minute 2
Meaf6	-0.35 ± 0.09	0.168	-0.47 ± 0.02	0.005	-1.03 ± 0.10	$p < 0.001$	ENSMUST00000154689	MYST/Esa1-associated factor 6
Mecp2	0.01 ± 0.18	1.000	0.07 ± 0.20	1.000	1.07 ± 0.16	$p < 0.001$	NM_001081979	methyl CpG binding protein 2
Med14	-0.07 ± 0.09	1.000	-0.10 ± 0.02	0.907	-1.02 ± 0.02	$p < 0.001$	NM_001048208	mediator complex subunit 14
Melk	0.09 ± 0.08	1.000	-0.27 ± 0.10	0.094	-2.61 ± 0.12	$p < 0.001$	NM_010790	maternal embryonic leucine zipper kinase
Men1	-0.18 ± 0.07	0.674	-0.24 ± 0.06	0.147	-1.05 ± 0.15	$p < 0.001$	NM_001168488	multiple endocrine neoplasia 1
Metrnl	0.29 ± 0.07	0.434	0.51 ± 0.01	0.001	1.53 ± 0.03	$p < 0.001$	NM_144797	meteorin, glial cell differentiation regulator-like
Mettl22	0.28 ± 0.07	0.583	0.34 ± 0.10	0.137	1.05 ± 0.06	$p < 0.001$	NM_146247	methyltransferase like 22
Mettl5	0.17 ± 0.03	1.000	0.36 ± 0.12	0.191	1.22 ± 0.14	$p < 0.001$	NM_029280	methyltransferase like 5
Mettl6	0.10 ± 0.08	1.000	0.48 ± 0.22	0.007	1.36 ± 0.09	$p < 0.001$	NM_025907	methyltransferase like 6

Mfsd2a	0.31 ± 0.10	0.128	-0.02 ± 0.10	1.000	-1.62 ± 0.14	$p < 0.001$	NM_029662	major facilitator superfamily domain containing 2A
Mfsd7a	-0.05 ± 0.41	1.000	0.09 ± 0.35	1.000	1.89 ± 0.35	$p < 0.001$	NM_172883	major facilitator superfamily domain containing 7A
Mfsd8	0.37 ± 0.03	0.734	0.24 ± 0.02	0.847	1.13 ± 0.13	$p < 0.001$	NM_028140	major facilitator superfamily domain containing 8
Mgat2	-0.10 ± 0.07	1.000	-0.32 ± 0.04	0.015	-1.18 ± 0.17	$p < 0.001$	NM_146035	mannoside acetylglucosaminyltransferase 2
Mgst3	-0.17 ± 0.10	0.927	-0.48 ± 0.10	0.002	-1.13 ± 0.08	$p < 0.001$	NM_025569	microsomal glutathione S-transferase 3
Mir155	0.58 ± 0.43	0.292	0.43 ± 0.32	0.413	2.41 ± 0.32	$p < 0.001$	NR_029565	microRNA 155
Mir17hg	-0.33 ± 0.14	0.460	-0.59 ± 0.18	0.004	-1.14 ± 0.13	$p < 0.001$	ENSMUST00000134140	Mir17 host gene 1 (non-protein coding)
Mir18	0.32 ± 0.13	1.000	-0.41 ± 0.23	0.501	-1.68 ± 0.26	$p < 0.001$	NR_029736	microRNA 18
Mir181b-1	0.48 ± 0.08	0.891	0.04 ± 0.24	1.000	1.12 ± 0.18	0.002	NR_029820	microRNA 181b-1
Mir186	0.02 ± 0.16	1.000	-0.88 ± 0.57	0.030	-1.25 ± 0.42	$p < 0.001$	NR_029572	microRNA 186
Mir1949	-0.19 ± 0.27	0.960	-0.53 ± 0.27	0.002	-1.80 ± 0.54	$p < 0.001$	NR_035472	microRNA 1949
Mir1970	-0.70 ± 0.96	0.202	-0.37 ± 0.68	0.670	-1.12 ± 0.71	0.001	NR_035497	microRNA 1970
Mir19a	-0.09 ± 0.54	1.000	-0.54 ± 0.36	0.432	-1.13 ± 0.34	0.003	NR_029786	microRNA 19a
Mir20a	-0.10 ± 0.46	1.000	-0.20 ± 0.46	1.000	-1.11 ± 0.48	0.003	NR_029737	microRNA 20a
Mir21a	0.10 ± 0.07	1.000	0.33 ± 0.02	0.074	1.28 ± 0.12	$p < 0.001$	NR_029738	microRNA 21a
Mir466p	0.52 ± 0.99	0.788	0.70 ± 0.57	0.174	1.12 ± 0.71	0.002	NR_105742	microRNA 466p
Mir505	-0.40 ± 0.07	0.989	-0.31 ± 0.14	0.862	-1.10 ± 0.70	0.003	NR_030499	microRNA 505
Mir5098	-1.16 ± 0.40	0.004	-0.83 ± 0.54	0.038	-0.09 ± 1.02	0.950	NR_039557	microRNA 5098
Mir5127	0.68 ± 0.15	0.354	0.49 ± 0.28	0.529	1.29 ± 0.15	$p < 0.001$	NR_039589	microRNA 5127
Mir5133	0.13 ± 0.22	1.000	0.63 ± 0.15	0.001	1.39 ± 0.15	$p < 0.001$	NR_039595	microRNA 5133
Mir677	-0.05 ± 0.32	1.000	-0.17 ± 0.18	1.000	-1.11 ± 0.44	0.001	NR_030442	microRNA 677
Mir697	-0.10 ± 0.49	1.000	-0.54 ± 0.23	0.252	-1.14 ± 0.42	$p < 0.001$	NR_030479	microRNA 697
Mis18bp1	0.00 ± 0.05	1.000	-0.38 ± 0.08	0.003	-2.00 ± 0.07	$p < 0.001$	NM_172578	MIS18 binding protein 1
Mki67	-0.03 ± 0.02	1.000	-0.19 ± 0.03	0.190	-2.04 ± 0.05	$p < 0.001$	NM_001081117	antigen identified by monoclonal antibody Ki 67
Mknk1	0.01 ± 0.09	1.000	0.51 ± 0.08	0.063	1.34 ± 0.08	$p < 0.001$	NM_001285487	MAP kinase-interacting serine/threonine kinase 1
Mknk2	-0.26 ± 0.05	0.203	-0.63 ± 0.10	$p < 0.001$	-1.04 ± 0.04	$p < 0.001$	NM_021462	MAP kinase-interacting serine/threonine kinase 2
Mmp19	-0.16 ± 0.08	1.000	0.11 ± 0.08	1.000	1.83 ± 0.22	$p < 0.001$	NM_001164197	matrix metallopeptidase 19
Mmp9	0.44 ± 0.14	0.682	1.82 ± 0.15	$p < 0.001$	3.07 ± 0.08	$p < 0.001$	NM_013599	matrix metallopeptidase 9
Mms22l	-0.12 ± 0.04	1.000	-0.34 ± 0.04	0.032	-1.70 ± 0.10	$p < 0.001$	NM_199467	MMS22-like, DNA repair protein
Mnda	1.25 ± 0.56	$p < 0.001$	2.99 ± 0.48	$p < 0.001$	3.65 ± 0.49	$p < 0.001$	NM_001033450	myeloid cell nuclear differentiation antigen
Mndal	0.54 ± 0.20	$p < 0.001$	1.33 ± 0.19	$p < 0.001$	2.38 ± 0.19	$p < 0.001$	NM_001170853	myeloid nuclear differentiation antigen like
Mogs	0.03 ± 0.08	1.000	-0.19 ± 0.08	0.380	-1.25 ± 0.30	$p < 0.001$	NM_020619	mannosyl-oligosaccharide glucosidase
Mospd2	0.04 ± 0.09	1.000	0.30 ± 0.39	0.344	1.09 ± 0.14	$p < 0.001$	NM_001290523	motile sperm domain containing 2
Mov10	0.27 ± 0.15	0.428	1.25 ± 0.15	$p < 0.001$	0.59 ± 0.15	$p < 0.001$	NM_001163440	Moloney leukemia virus 10
Mpeg1	0.42 ± 0.04	$p < 0.001$	1.17 ± 0.02	$p < 0.001$	1.53 ± 0.06	$p < 0.001$	NM_010821	macrophage expressed gene 1
Mpv17l	0.23 ± 0.18	1.000	0.29 ± 0.12	0.547	1.17 ± 0.20	$p < 0.001$	NM_001289561	Mpv17 transgene, kidney disease mutant-like
Mr1	0.02 ± 0.04	1.000	0.61 ± 0.01	$p < 0.001$	1.48 ± 0.04	$p < 0.001$	NM_008209	major histocompatibility complex, class I-related
Mrpl12	-0.22 ± 0.03	0.484	-0.24 ± 0.05	0.164	-1.34 ± 0.19	$p < 0.001$	NM_027204	mitochondrial ribosomal protein L12
Mrpl15	-0.23 ± 0.03	0.450	-0.28 ± 0.10	0.094	-1.16 ± 0.17	$p < 0.001$	NM_001177658	mitochondrial ribosomal protein L15
Mrpl18	-0.12 ± 0.10	1.000	-0.38 ± 0.13	0.002	-1.16 ± 0.09	$p < 0.001$	NM_026310	mitochondrial ribosomal protein L18
Mrpl41	-0.29 ± 0.22	0.494	-0.40 ± 0.23	0.050	-1.00 ± 0.28	$p < 0.001$	NM_001031808	mitochondrial ribosomal protein L41

Mrpl49	-0.20 ± 0.06	0.616	-0.40 ± 0.09	0.002	-1.02 ± 0.05	$p < 0.001$	NM_026246	mitochondrial ribosomal protein L49
Ms4a6c	0.14 ± 0.43	1.000	0.54 ± 0.21	0.080	1.05 ± 0.23	$p < 0.001$	NM_028595	membrane-spanning 4-domains, subfamily A, member 6C
Ms4a6d	-0.05 ± 0.13	1.000	0.42 ± 0.06	0.002	1.93 ± 0.07	$p < 0.001$	NM_026835	membrane-spanning 4-domains, subfamily A, member 6D
Msh2	0.04 ± 0.20	1.000	-0.13 ± 0.24	0.961	-1.78 ± 0.38	$p < 0.001$	NM_008628	mutS homolog 2 (E. coli)
Msh6	-0.05 ± 0.01	1.000	-0.40 ± 0.05	0.002	-1.71 ± 0.18	$p < 0.001$	NM_010830	mutS homolog 6 (E. coli)
Msmo1	-0.13 ± 0.01	0.970	-0.21 ± 0.03	0.226	-1.41 ± 0.09	$p < 0.001$	ENSMUST00000034015	methylsterol monoxygenase 1
Msr1	0.31 ± 0.06	0.113	1.36 ± 0.08	$p < 0.001$	1.61 ± 0.22	$p < 0.001$	NM_001113326	macrophage scavenger receptor 1
Msto1	-0.13 ± 0.08	0.982	-0.31 ± 0.09	0.025	-1.86 ± 0.15	$p < 0.001$	NM_144898	misato homolog 1 (Drosophila)
Mt1	-0.08 ± 0.03	1.000	-0.21 ± 0.03	0.051	-1.15 ± 0.07	$p < 0.001$	NM_013602	metallothionein 1
Mt2	0.00 ± 0.08	1.000	-0.11 ± 0.07	0.509	-1.03 ± 0.09	$p < 0.001$	NM_008630	metallothionein 2
Mtbp	0.04 ± 0.06	1.000	-0.04 ± 0.07	1.000	-1.12 ± 0.07	$p < 0.001$	NM_134092	Mdm2, transformed 3T3 cell double minute p53 binding protein
Mtfr2	0.01 ± 0.33	1.000	-0.44 ± 0.35	0.170	-1.38 ± 0.48	$p < 0.001$	NM_027930	mitochondrial fission regulator 2
Mthfd1	-0.15 ± 0.07	0.846	-0.24 ± 0.07	0.139	-1.54 ± 0.18	$p < 0.001$	NM_138745	methylenetetrahydrofolate dehydrogenase (NADP+ dependent), methenyltetrahydrofolate cyclohydrolase
Mthfd1l	-0.17 ± 0.07	0.672	-0.06 ± 0.05	1.000	-1.34 ± 0.09	$p < 0.001$	NM_001170785	methylenetetrahydrofolate dehydrogenase (NADP+ dependent) 1-like
Mtm1	0.12 ± 0.24	1.000	0.53 ± 0.03	0.034	1.10 ± 0.06	$p < 0.001$	NM_001164190	X-linked myotubular myopathy gene 1
Mtmr4	-0.25 ± 0.15	0.549	-0.35 ± 0.15	0.053	-1.37 ± 0.25	$p < 0.001$	NM_133215	myotubularin related protein 4
mt-Tc	-0.91 ± 0.25	0.013	-1.49 ± 0.25	$p < 0.001$	-1.25 ± 0.43	$p < 0.001$	ENSMUST00000082400	mitochondrially encoded tRNA cysteine
mt-Tf	-0.42 ± 0.09	0.001	-0.76 ± 0.13	$p < 0.001$	-2.36 ± 0.11	$p < 0.001$	ENSMUST00000082387	mitochondrially encoded tRNA phenylalanine
mt-Th	-0.60 ± 0.09	$p < 0.001$	-0.59 ± 0.13	$p < 0.001$	-1.37 ± 0.23	$p < 0.001$	ENSMUST00000082415	mitochondrially encoded tRNA histidine
mt-Tm	-0.78 ± 0.14	$p < 0.001$	-1.23 ± 0.18	$p < 0.001$	-1.91 ± 0.17	$p < 0.001$	ENSMUST00000082395	mitochondrially encoded tRNA methionine
mt-Tq	-0.52 ± 0.41	0.574	-2.01 ± 0.98	$p < 0.001$	-1.12 ± 1.43	0.001	ENSMUST00000082394	mitochondrially encoded tRNA glutamine
mt-Tr	-0.56 ± 0.15	$p < 0.001$	-0.80 ± 0.35	$p < 0.001$	-1.72 ± 0.27	$p < 0.001$	ENSMUST00000082412	mitochondrially encoded tRNA arginine
mt-Ts1	-0.46 ± 0.14	0.554	-1.17 ± 0.13	$p < 0.001$	-0.17 ± 0.10	0.836	ENSMUST00000082403	mitochondrially encoded tRNA serine 1
mt-Ts2	-0.55 ± 0.06	0.019	-0.91 ± 0.05	$p < 0.001$	-1.51 ± 0.54	$p < 0.001$	ENSMUST00000082416	mitochondrially encoded tRNA serine 2
mt-Ty	-0.69 ± 0.10	$p < 0.001$	-1.40 ± 0.11	$p < 0.001$	-1.18 ± 0.27	$p < 0.001$	ENSMUST00000082401	mitochondrially encoded tRNA tyrosine
Mum1	-0.01 ± 0.22	1.000	-0.18 ± 0.07	0.743	-2.00 ± 0.07	$p < 0.001$	NM_023431	melanoma associated antigen (mutated) 1
Mup4	0.44 ± 0.15	0.972	0.37 ± 0.62	0.814	1.10 ± 0.68	0.003	NM_008648	major urinary protein 4
Mvd	-0.29 ± 0.13	0.078	-0.26 ± 0.07	0.096	-2.04 ± 0.13	$p < 0.001$	NM_138656	mevalonate (diphospho) decarboxylase
Mvk	-0.01 ± 0.17	1.000	-0.20 ± 0.06	0.623	-1.18 ± 0.15	$p < 0.001$	NM_023556	mevalonate kinase
Mx1	0.31 ± 0.31	1.000	0.60 ± 0.28	0.276	1.18 ± 0.28	$p < 0.001$	NM_010846	myxovirus (influenza virus) resistance 1
Mx2	1.94 ± 0.77	$p < 0.001$	2.88 ± 0.77	$p < 0.001$	3.54 ± 0.80	$p < 0.001$	NM_013606	myxovirus (influenza virus) resistance 2
Mxd1	0.10 ± 0.15	1.000	0.61 ± 0.15	$p < 0.001$	1.34 ± 0.17	$p < 0.001$	NM_010751	MAX dimerization protein 1
Mybl2	-0.21 ± 0.18	0.327	-0.55 ± 0.11	$p < 0.001$	-2.79 ± 0.18	$p < 0.001$	NM_008652	myeloblastosis oncogene-like 2
Myc	0.23 ± 0.24	1.000	0.47 ± 0.23	0.095	2.27 ± 0.28	$p < 0.001$	ENSMUST00000160009	myelocytomatosis oncogene
Mycn	-0.16 ± 0.05	0.778	-0.22 ± 0.02	0.193	-1.87 ± 0.10	$p < 0.001$	ENSMUST00000130990	v-myc myelocytomatosis viral related oncogene, neuroblastoma derived (avian)
Myo19	-0.02 ± 0.20	1.000	-0.35 ± 0.10	0.019	-1.21 ± 0.17	$p < 0.001$	XM_006533927	myosin XIX
Naga	0.16 ± 0.07	0.903	0.31 ± 0.03	0.034	1.15 ± 0.04	$p < 0.001$	NM_008669	N-acetyl galactosaminidase, alpha
Nagpa	-0.23 ± 0.02	0.229	-0.49 ± 0.13	$p < 0.001$	-1.20 ± 0.06	$p < 0.001$	NM_013796	N-acetylglucosamine-1-phosphodiester alpha-N-acetylglucosaminidase
Naip2	0.24 ± 0.09	1.000	0.63 ± 0.16	0.022	1.28 ± 0.13	$p < 0.001$	NM_001126182	NLR family, apoptosis inhibitory protein 2
Naip5	0.53 ± 0.08	0.297	0.68 ± 0.11	0.020	1.52 ± 0.19	$p < 0.001$	NM_010870	NLR family, apoptosis inhibitory protein 5
Nanp	0.01 ± 0.30	1.000	0.29 ± 0.31	0.589	-1.19 ± 0.48	$p < 0.001$	NM_026086	N-acetylneuraminic acid phosphatase

Napb	0.12 ± 0.16	1.000	-0.18 ± 0.19	0.888	3.01 ± 0.20	$p < 0.001$	NM_019632	N-ethylmaleimide sensitive fusion protein attachment protein beta
Nasp	0.05 ± 0.10	1.000	-0.10 ± 0.04	0.915	-1.73 ± 0.03	$p < 0.001$	NM_001081475	nuclear autoantigenic sperm protein (histone-binding)
Nbeal1	0.02 ± 0.08	1.000	0.27 ± 0.09	0.621	1.10 ± 0.06	$p < 0.001$	NM_173444	neurobeachin like 1
Nbr1	-0.02 ± 0.15	1.000	0.21 ± 0.07	0.268	1.19 ± 0.07	$p < 0.001$	NM_001252220	neighbor of Brca1 gene 1
Ncapd2	0.01 ± 0.06	1.000	-0.46 ± 0.06	$p < 0.001$	-2.09 ± 0.22	$p < 0.001$	NM_146171	non-SMC condensin I complex, subunit D2
Ncapd3	-0.18 ± 0.10	0.614	-0.23 ± 0.09	0.162	-1.17 ± 0.08	$p < 0.001$	NM_178113	non-SMC condensin II complex, subunit D3
Ncaph	-0.01 ± 0.07	1.000	-0.28 ± 0.07	0.061	-1.25 ± 0.20	$p < 0.001$	NM_144818	non-SMC condensin I complex, subunit H
Ncaph2	-0.12 ± 0.26	1.000	-0.25 ± 0.22	0.108	-1.12 ± 0.25	$p < 0.001$	NM_001115132	non-SMC condensin II complex, subunit H2
Ndc1	-0.05 ± 0.02	1.000	-0.30 ± 0.03	0.021	-1.27 ± 0.02	$p < 0.001$	NM_028355	NDC1 transmembrane nucleoporin
Ndc80	0.06 ± 0.07	1.000	-0.21 ± 0.05	0.234	-1.37 ± 0.15	$p < 0.001$	NM_023294	NDC80 homolog, kinetochore complex component (S. cerevisiae)
Nde1	0.08 ± 0.18	1.000	-0.24 ± 0.16	0.184	-1.32 ± 0.16	$p < 0.001$	ENSMUST00000149232	nuclear distribution gene E homolog 1 (A nidulans)
Ndfip2	0.08 ± 0.05	1.000	0.16 ± 0.05	0.709	1.00 ± 0.07	$p < 0.001$	NM_001190989	Nedd4 family interacting protein 2
Ndrg1	-0.16 ± 0.02	0.634	0.31 ± 0.03	0.004	1.41 ± 0.04	$p < 0.001$	ENSMUST00000163496	N-myc downstream regulated gene 1
Ndrg2	-0.42 ± 0.05	0.006	-0.42 ± 0.08	0.001	-1.12 ± 0.05	$p < 0.001$	NM_001145959	N-myc downstream regulated gene 2
Neat1	-0.87 ± 0.01	$p < 0.001$	-1.05 ± 0.06	$p < 0.001$	-0.29 ± 0.19	0.025	NR_003513	nuclear paraspeckle assembly transcript 1 (non-protein coding)
Nedd4l	0.06 ± 0.32	1.000	0.14 ± 0.31	0.933	2.52 ± 0.31	$p < 0.001$	NM_001114386	neural precursor cell expressed, developmentally down-regulated gene 4-like
Neil3	-0.16 ± 0.10	1.000	-0.33 ± 0.13	0.234	-1.38 ± 0.10	$p < 0.001$	NM_146208	nei like 3 (E. coli)
Nek2	0.21 ± 0.04	0.424	-0.23 ± 0.04	0.174	-1.94 ± 0.04	$p < 0.001$	NM_010892	NIMA (never in mitosis gene a)-related expressed kinase 2
Neurl3	0.59 ± 0.45	0.002	0.22 ± 0.12	0.630	2.51 ± 0.13	$p < 0.001$	NM_153408	neuralized homolog 3 homolog (Drosophila)
Nf1	0.12 ± 0.16	1.000	0.30 ± 0.17	0.145	1.01 ± 0.16	$p < 0.001$	NM_010897	neurofibromatosis 1
Nf2	0.22 ± 0.08	0.551	0.11 ± 0.11	0.886	-1.14 ± 0.12	$p < 0.001$	NM_001252250	neurofibromatosis 2
Nfkb1	0.55 ± 0.07	$p < 0.001$	0.88 ± 0.00	$p < 0.001$	1.73 ± 0.04	$p < 0.001$	NM_008689	nuclear factor of kappa light polypeptide gene enhancer in B cells 1, p105
Nfkb2	0.86 ± 0.08	$p < 0.001$	0.89 ± 0.08	$p < 0.001$	1.65 ± 0.09	$p < 0.001$	NM_001177369	nuclear factor of kappa light polypeptide gene enhancer in B cells 2, p49/p100
Nfkbia	1.22 ± 0.08	$p < 0.001$	1.64 ± 0.15	$p < 0.001$	2.57 ± 0.08	$p < 0.001$	NM_010907	nuclear factor of kappa light polypeptide gene enhancer in B cells inhibitor, alpha
Nfkbib	0.32 ± 0.05	0.086	0.46 ± 0.11	$p < 0.001$	1.43 ± 0.05	$p < 0.001$	ENSMUST00000032815	nuclear factor of kappa light polypeptide gene enhancer in B cells inhibitor, beta
Nfkbie	0.58 ± 0.09	0.055	0.74 ± 0.20	0.001	2.10 ± 0.09	$p < 0.001$	NM_008690	nuclear factor of kappa light polypeptide gene enhancer in B cells inhibitor, epsilon
Nfkbiz	2.01 ± 0.04	$p < 0.001$	2.55 ± 0.10	$p < 0.001$	4.77 ± 0.03	$p < 0.001$	NM_001159394	nuclear factor of kappa light polypeptide gene enhancer in B cells inhibitor, zeta
Nfxl1	0.15 ± 0.03	1.000	0.34 ± 0.08	0.087	1.06 ± 0.03	$p < 0.001$	NM_133921	nuclear transcription factor, X-box binding-like 1
Ngdn	0.03 ± 0.12	1.000	-0.09 ± 0.11	0.985	1.19 ± 0.15	$p < 0.001$	NM_026890	neuroguidin, EIF4E binding protein
Nhlrc3	0.13 ± 0.15	1.000	0.03 ± 0.15	1.000	1.48 ± 0.15	$p < 0.001$	NM_172501	NHL repeat containing 3
Nhp2l1	-0.12 ± 0.08	0.909	-0.13 ± 0.15	0.621	-1.16 ± 0.09	$p < 0.001$	NM_011482	NHP2 non-histone chromosome protein 2-like 1 (S. cerevisiae)
Nkain1	-0.11 ± 0.13	1.000	-0.23 ± 0.16	0.175	-1.01 ± 0.13	$p < 0.001$	NM_025998	Na+/K+ transporting ATPase interacting 1
Nkrf	-0.05 ± 0.12	1.000	-0.20 ± 0.13	0.574	-1.01 ± 0.17	$p < 0.001$	NM_029891	NF-kappaB repressing factor
Nlrc5	0.31 ± 0.07	0.901	0.79 ± 0.11	0.001	1.45 ± 0.06	$p < 0.001$	NM_001033207	NLR family, CARD domain containing 5
Nlrp10	0.00 ± 0.23	1.000	-0.33 ± 0.09	0.098	-1.24 ± 0.14	$p < 0.001$	NM_175532	NLR family, pyrin domain containing 10
Nlrp3	0.98 ± 0.09	$p < 0.001$	0.96 ± 0.08	$p < 0.001$	2.02 ± 0.08	$p < 0.001$	NM_145827	NLR family, pyrin domain containing 3
Nme3	-0.01 ± 0.08	1.000	-0.34 ± 0.20	0.094	-1.34 ± 0.08	$p < 0.001$	NM_019730	NME/NM23 nucleoside diphosphate kinase 3
Nmi	0.35 ± 0.19	0.380	0.98 ± 0.16	$p < 0.001$	1.69 ± 0.13	$p < 0.001$	NM_001141948	N-myc (and STAT) interactor
Noa1	0.01 ± 0.02	1.000	-0.12 ± 0.11	0.833	-1.05 ± 0.04	$p < 0.001$	NM_019836	nitric oxide associated 1
Noc4l	-0.09 ± 0.10	1.000	-0.44 ± 0.04	0.001	-1.01 ± 0.08	$p < 0.001$	NM_153570	nucleolar complex associated 4 homolog (S. cerevisiae)
Nod2	0.17 ± 0.13	1.000	0.40 ± 0.12	0.338	1.13 ± 0.17	$p < 0.001$	NM_145857	nucleotide-binding oligomerization domain containing 2

Nop2	-0.07 ± 0.05	1.000	-0.38 ± 0.06	0.001	-1.21 ± 0.08	$p < 0.001$	NM_138747	NOP2 nucleolar protein
Nos2	0.19 ± 0.11	1.000	1.01 ± 0.42	0.001	1.23 ± 0.12	$p < 0.001$	NM_010927	nitric oxide synthase 2, inducible
Nox1	-0.14 ± 0.03	1.000	0.11 ± 0.06	1.000	1.04 ± 0.30	0.001	NM_172203	NADPH oxidase 1
Nox3	0.48 ± 0.25	0.370	0.51 ± 0.16	0.144	2.51 ± 0.06	$p < 0.001$	NM_198958	NADPH oxidase 3
Nr1d1	0.44 ± 0.13	0.523	0.80 ± 0.15	0.002	2.77 ± 0.20	$p < 0.001$	NM_145434	nuclear receptor subfamily 1, group D, member 1
Nr1d2	-0.16 ± 0.21	1.000	0.45 ± 0.18	0.119	1.21 ± 0.18	$p < 0.001$	NM_011584	nuclear receptor subfamily 1, group D, member 2
Nr4a3	-0.22 ± 0.08	1.000	-0.11 ± 0.21	1.000	1.27 ± 0.22	$p < 0.001$	ENSMUST00000030025	nuclear receptor subfamily 4, group A, member 3
n-R5s127	-1.02 ± 0.13	0.016	-0.05 ± 0.25	1.000	-0.58 ± 0.79	0.169	ENSMUST00000179185	nuclear encoded rRNA 5S 127
n-R5s155	-1.04 ± 0.19	0.018	0.11 ± 0.13	1.000	0.63 ± 0.59	0.095	ENSMUST00000122531	nuclear encoded rRNA 5S 155
n-R5s29	0.33 ± 0.37	1.000	0.39 ± 0.36	0.734	1.00 ± 0.71	0.005	ENSMUST00000082836	nuclear encoded rRNA 5S 29
n-R5s48	0.11 ± 0.34	1.000	1.08 ± 0.23	0.002	1.21 ± 0.24	$p < 0.001$	ENSMUST00000122482	nuclear encoded rRNA 5S 48
Nrm	-0.09 ± 0.10	1.000	-0.30 ± 0.15	0.048	-1.16 ± 0.10	$p < 0.001$	NM_134122	nurim (nuclear envelope membrane protein)
Nsdhl	-0.27 ± 0.05	0.074	-0.54 ± 0.09	$p < 0.001$	-2.46 ± 0.05	$p < 0.001$	NM_010941	NAD(P) dependent steroid dehydrogenase-like
Nsf	-0.09 ± 0.06	1.000	-0.32 ± 0.08	0.012	-1.12 ± 0.06	$p < 0.001$	NM_008740	N-ethylmaleimide sensitive fusion protein
Nsmce4a	0.05 ± 0.08	1.000	-0.05 ± 0.02	1.000	-1.69 ± 0.10	$p < 0.001$	NM_001162855	non-SMC element 4 homolog A (S. cerevisiae)
Nt5c2	-0.21 ± 0.04	0.537	-0.56 ± 0.07	$p < 0.001$	-1.59 ± 0.07	$p < 0.001$	NM_001164363	5-nucleotidase, cytosolic II
Nt5dc2	-0.15 ± 0.07	1.000	-0.54 ± 0.22	$p < 0.001$	-1.72 ± 0.10	$p < 0.001$	NM_027289	5-nucleotidase domain containing 2
Nucks1	-0.03 ± 0.06	1.000	-0.22 ± 0.06	0.157	-1.05 ± 0.11	$p < 0.001$	NM_001145804	nuclear casein kinase and cyclin-dependent kinase substrate 1
Nudt2	-0.05 ± 0.07	1.000	0.20 ± 0.07	0.517	-1.17 ± 0.16	$p < 0.001$	NM_025539	nudix (nucleoside diphosphate linked moiety X)-type motif 2
Nudt4	-0.01 ± 0.14	1.000	-0.24 ± 0.11	0.166	-1.11 ± 0.13	$p < 0.001$	NM_027722	nudix (nucleoside diphosphate linked moiety X)-type motif 4
Nuf2	0.09 ± 0.08	1.000	-0.38 ± 0.08	0.001	-1.98 ± 0.12	$p < 0.001$	NM_023284	NUF2, NDC80 kinetochore complex component, homolog (S. cerevisiae)
Nup107	0.01 ± 0.06	1.000	-0.26 ± 0.06	0.090	-1.66 ± 0.05	$p < 0.001$	NM_134010	nucleoporin 107
Nup133	-0.08 ± 0.09	1.000	-0.42 ± 0.07	0.019	-1.79 ± 0.16	$p < 0.001$	NM_172288	nucleoporin 133
Nup155	-0.18 ± 0.05	0.541	-0.34 ± 0.04	0.006	-1.53 ± 0.08	$p < 0.001$	NM_133227	nucleoporin 155
Nup205	0.12 ± 0.05	1.000	0.10 ± 0.04	0.919	-1.48 ± 0.05	$p < 0.001$	NM_027513	nucleoporin 205
Nup43	-0.11 ± 0.08	1.000	-0.20 ± 0.11	0.466	-2.10 ± 0.08	$p < 0.001$	NM_145706	nucleoporin 43
Nup85	0.04 ± 0.07	1.000	-0.38 ± 0.07	0.012	-2.15 ± 0.30	$p < 0.001$	NM_001002929	nucleoporin 85
Nupr1	0.27 ± 0.05	0.223	0.57 ± 0.08	$p < 0.001$	2.18 ± 0.09	$p < 0.001$	ENSMUST00000032961	nuclear protein transcription regulator 1
Nusap1	0.08 ± 0.04	1.000	-0.13 ± 0.06	0.839	-1.02 ± 0.07	$p < 0.001$	NM_001042652	nucleolar and spindle associated protein 1
Oas1a	0.75 ± 0.43	$p < 0.001$	1.23 ± 0.41	$p < 0.001$	1.49 ± 0.45	$p < 0.001$	NM_145211	2-5 oligoadenylate synthetase 1A
Oas1b	0.49 ± 0.15	0.439	1.35 ± 0.17	$p < 0.001$	2.05 ± 0.14	$p < 0.001$	NR_003507	2-5 oligoadenylate synthetase 1B
Oas1g	0.73 ± 0.19	$p < 0.001$	1.28 ± 0.20	$p < 0.001$	1.80 ± 0.19	$p < 0.001$	NM_011852	2-5 oligoadenylate synthetase 1G
Oas2	1.12 ± 0.20	$p < 0.001$	1.57 ± 0.19	$p < 0.001$	1.84 ± 0.19	$p < 0.001$	NM_145227	2-5 oligoadenylate synthetase 2
Oas3	0.93 ± 0.10	$p < 0.001$	2.13 ± 0.10	$p < 0.001$	1.86 ± 0.10	$p < 0.001$	NM_145226	2-5 oligoadenylate synthetase 3
Oasl1	0.90 ± 0.15	$p < 0.001$	1.36 ± 0.19	$p < 0.001$	2.75 ± 0.15	$p < 0.001$	NM_145209	2-5 oligoadenylate synthetase-like 1
Oasl2	1.51 ± 0.11	$p < 0.001$	3.62 ± 0.11	$p < 0.001$	3.72 ± 0.08	$p < 0.001$	NM_011854	2-5 oligoadenylate synthetase-like 2
Oat	-0.13 ± 0.07	0.952	-0.20 ± 0.09	0.283	-1.33 ± 0.09	$p < 0.001$	NM_016978	ornithine aminotransferase
Oit3	-0.18 ± 0.21	1.000	0.09 ± 0.17	1.000	1.56 ± 0.10	$p < 0.001$	NM_010959	oncoprotein induced transcript 3
Olfm1	-0.03 ± 0.07	1.000	-0.15 ± 0.05	0.541	-2.08 ± 0.13	$p < 0.001$	ENSMUST00000113920	olfactomedin 1
Olfr218	0.55 ± 0.50	0.684	0.29 ± 0.56	0.927	1.03 ± 0.57	0.005	NM_001001809	olfactory receptor 218
Olfr433	0.04 ± 0.28	1.000	0.01 ± 0.34	1.000	1.13 ± 0.49	0.002	NM_146717	olfactory receptor 433

Olfr869	0.46 ± 0.10	0.794	0.45 ± 0.18	0.529	1.05 ± 0.42	0.001	NM_146557	olfactory receptor 869
Olfr99	0.36 ± 0.24	1.000	0.47 ± 0.27	0.601	1.04 ± 0.55	0.005	NM_146515	olfactory receptor 99
Orai3	-0.23 ± 0.12	0.755	-0.54 ± 0.16	0.002	-1.11 ± 0.18	*p* < 0.001	NM_198424	ORAI calcium release-activated calcium modulator 3
Orc6	-0.24 ± 0.08	0.319	-0.46 ± 0.02	*p* < 0.001	-1.22 ± 0.08	*p* < 0.001	NM_001163791	origin recognition complex, subunit 6
Osbp2	-0.05 ± 0.31	1.000	-0.02 ± 0.22	1.000	2.45 ± 0.36	*p* < 0.001	NM_152818	oxysterol binding protein 2
Osbpl2	0.16 ± 0.05	0.801	0.62 ± 0.07	*p* < 0.001	1.69 ± 0.07	*p* < 0.001	NM_144500	oxysterol binding protein-like 2
Osgin2	0.49 ± 0.02	*p* < 0.001	0.94 ± 0.04	*p* < 0.001	1.61 ± 0.02	*p* < 0.001	NM_145950	oxidative stress induced growth inhibitor family member 2
Osm	0.48 ± 0.17	0.169	0.38 ± 0.24	0.262	1.77 ± 0.18	*p* < 0.001	NM_001013365	oncostatin M
P2rx4	0.29 ± 0.16	0.175	0.73 ± 0.15	*p* < 0.001	1.72 ± 0.06	*p* < 0.001	NM_011026	purinergic receptor P2X, ligand-gated ion channel 4
P2ry6	0.29 ± 0.03	0.046	0.10 ± 0.09	0.872	-1.77 ± 0.14	*p* < 0.001	NM_183168	pyrimidinergic receptor P2Y, G-protein coupled, 6
Paics	-0.16 ± 0.06	0.815	-0.23 ± 0.04	0.159	-1.02 ± 0.15	*p* < 0.001	NM_025939	phosphoribosylaminoimidazole carboxylase
Palb2	-0.24 ± 0.19	0.967	-0.46 ± 0.12	0.074	-1.63 ± 0.28	*p* < 0.001	NM_001081238	partner and localizer of BRCA2
Pank1	0.04 ± 0.12	1.000	-0.27 ± 0.19	0.669	-1.25 ± 0.12	*p* < 0.001	NM_001114339	pantothenate kinase 1
Paqr3	0.30 ± 0.16	0.877	0.89 ± 0.21	*p* < 0.001	1.86 ± 0.17	*p* < 0.001	XM_006534874	progestin and adipoQ receptor family member III
Parp1	0.02 ± 0.07	1.000	-0.03 ± 0.08	1.000	-1.98 ± 0.18	*p* < 0.001	NM_007415	poly (ADP-ribose) polymerase family, member 1
Parp10	0.57 ± 0.09	0.006	1.48 ± 0.10	*p* < 0.001	1.54 ± 0.10	*p* < 0.001	NM_001163575	poly (ADP-ribose) polymerase family, member 10
Parp12	0.51 ± 0.03	*p* < 0.001	1.43 ± 0.06	*p* < 0.001	0.37 ± 0.03	0.003	NM_172893	poly (ADP-ribose) polymerase family, member 12
Parp14	1.58 ± 0.17	*p* < 0.001	2.69 ± 0.18	*p* < 0.001	2.85 ± 0.23	*p* < 0.001	NM_001039530	poly (ADP-ribose) polymerase family, member 14
Parp4	0.23 ± 0.09	0.834	0.62 ± 0.11	*p* < 0.001	1.54 ± 0.09	*p* < 0.001	NM_001145978	poly (ADP-ribose) polymerase family, member 4
Parp9	0.61 ± 0.09	*p* < 0.001	1.33 ± 0.05	*p* < 0.001	0.79 ± 0.09	*p* < 0.001	NM_030253	poly (ADP-ribose) polymerase family, member 9
Parpbp	0.01 ± 0.13	1.000	-0.26 ± 0.15	0.263	-1.80 ± 0.13	*p* < 0.001	NM_029249	PARP1 binding protein
Patz1	0.00 ± 0.10	1.000	-0.38 ± 0.10	0.247	-1.02 ± 0.14	*p* < 0.001	NM_001253690	POZ (BTB) and AT hook containing zinc finger 1
Paxip1	-0.09 ± 0.04	1.000	-0.29 ± 0.05	0.049	-2.02 ± 0.03	*p* < 0.001	NM_018878	PAX interacting (with transcription-activation domain) protein 1
Pbk	-0.12 ± 0.08	1.000	-0.34 ± 0.10	0.008	-2.13 ± 0.14	*p* < 0.001	NM_023209	PDZ binding kinase
Pcbp1	0.12 ± 0.02	0.974	0.04 ± 0.05	1.000	-1.06 ± 0.11	*p* < 0.001	NM_011865	poly(rC) binding protein 1
Pcna	-0.18 ± 0.01	0.267	-0.23 ± 0.01	0.026	-1.14 ± 0.14	*p* < 0.001	ENSMUST00000028817	proliferating cell nuclear antigen
Pcyox1l	-0.02 ± 0.21	1.000	-0.26 ± 0.24	0.212	-1.52 ± 0.21	*p* < 0.001	NM_172832	prenylcysteine oxidase 1 like
Pcyt2	-0.25 ± 0.12	0.248	-0.45 ± 0.10	*p* < 0.001	-2.21 ± 0.14	*p* < 0.001	NM_024229	phosphate cytidylyltransferase 2, ethanolamine
Pde2a	-0.13 ± 0.04	0.801	-0.34 ± 0.02	0.002	-1.20 ± 0.03	*p* < 0.001	NM_001008548	phosphodiesterase 2A, cGMP-stimulated
Pde7b	-0.12 ± 0.09	1.000	-0.55 ± 0.11	*p* < 0.001	-1.25 ± 0.15	*p* < 0.001	NM_013875	phosphodiesterase 7B
Pdgfb	0.17 ± 0.02	0.666	-0.25 ± 0.03	0.113	-1.75 ± 0.03	*p* < 0.001	NM_011057	platelet derived growth factor, B polypeptide
Pdp2	-0.11 ± 0.30	1.000	-0.09 ± 0.30	1.000	-1.17 ± 0.28	*p* < 0.001	NM_001024606	pyruvate dehyrogenase phosphatase catalytic subunit 2
Pdpr	-0.13 ± 0.15	1.000	-0.13 ± 0.17	0.929	-1.40 ± 0.28	*p* < 0.001	NM_198308	pyruvate dehydrogenase phosphatase regulatory subunit
Pdss1	0.11 ± 0.08	1.000	-0.21 ± 0.08	0.651	-1.28 ± 0.13	*p* < 0.001	XM_006498189	prenyl (solanesyl) diphosphate synthase, subunit 1
Pdxk	-0.15 ± 0.09	0.887	-0.40 ± 0.08	0.001	-1.05 ± 0.20	*p* < 0.001	NM_172134	pyridoxal (pyridoxine, vitamin B6) kinase
Pdxp	-0.19 ± 0.08	1.000	-0.25 ± 0.09	0.569	-1.17 ± 0.20	*p* < 0.001	NM_020271	pyridoxal (pyridoxine, vitamin B6) phosphatase
Peli1	0.12 ± 0.12	1.000	0.40 ± 0.09	0.006	2.25 ± 0.08	*p* < 0.001	ENSMUST00000101477	pellino 1
Peo1	-0.28 ± 0.02	0.711	-0.32 ± 0.06	0.301	-1.20 ± 0.25	*p* < 0.001	NM_153796	progressive external ophthalmoplegia 1 (human)
Pex10	-0.31 ± 0.07	0.354	-0.36 ± 0.06	0.075	-1.05 ± 0.28	*p* < 0.001	ENSMUST00000125432	peroxisomal biogenesis factor 10
Pfas	-0.04 ± 0.13	1.000	-0.12 ± 0.11	0.954	-1.27 ± 0.13	*p* < 0.001	NM_001159519	phosphoribosylformylglycinamidine synthase (FGAR amidotransferase)
Pfdn6	0.02 ± 0.17	1.000	-0.06 ± 0.11	1.000	-1.18 ± 0.13	*p* < 0.001	NM_001185182	prefoldin subunit 6

Pfkl	-0.09 ± 0.07	1.000	0.04 ± 0.10	1.000	-1.34 ± 0.12	$p < 0.001$	NM_008826	phosphofructokinase, liver, B-type
Pgam1	-0.21 ± 0.14	0.538	-0.34 ± 0.14	0.021	-1.06 ± 0.21	$p < 0.001$	NM_023418	phosphoglycerate mutase 1
Pgd	-0.36 ± 0.00	$p < 0.001$	-0.61 ± 0.01	$p < 0.001$	-2.13 ± 0.07	$p < 0.001$	NM_001081274	phosphogluconate dehydrogenase
Pgf	0.58 ± 0.32	0.565	0.41 ± 0.45	0.684	1.16 ± 0.30	0.001	NM_001271705	placental growth factor
Pgp	-0.07 ± 0.03	1.000	-0.30 ± 0.05	0.039	-2.14 ± 0.10	$p < 0.001$	NM_025954	phosphoglycolate phosphatase
Phf11b	0.38 ± 0.24	0.553	1.51 ± 0.20	$p < 0.001$	0.91 ± 0.19	$p < 0.001$	NM_001164327	PHD finger protein 11B
Phf11c	0.07 ± 0.36	1.000	0.66 ± 0.33	0.057	1.52 ± 0.15	$p < 0.001$	NM_001164289	PHD finger protein 11C
Phf11d	1.06 ± 0.11	$p < 0.001$	2.03 ± 0.09	$p < 0.001$	1.71 ± 0.12	$p < 0.001$	NM_199015	PHD finger protein 11D
Phka2	-0.08 ± 0.01	1.000	-0.33 ± 0.05	0.018	-1.30 ± 0.07	$p < 0.001$	NM_001177878	phosphorylase kinase alpha 2
Phlda1	0.70 ± 0.23	0.023	0.51 ± 0.18	0.122	1.56 ± 0.20	$p < 0.001$	NM_009344	pleckstrin homology-like domain, family A, member 1
Phospho2	-0.12 ± 0.18	1.000	0.01 ± 0.11	1.000	1.10 ± 0.22	$p < 0.001$	ENSMUST00000028494	phosphatase, orphan 2
Phyhd1	0.07 ± 0.14	1.000	0.67 ± 0.09	0.002	2.50 ± 0.17	$p < 0.001$	NM_001252568	phytanoyl-CoA dioxygenase domain containing 1
Picalm	0.15 ± 0.10	1.000	0.61 ± 0.15	0.028	1.34 ± 0.20	$p < 0.001$	AK147969	phosphatidylinositol binding clathrin assembly protein
Pidd1	-0.30 ± 0.11	0.442	-0.55 ± 0.06	0.003	-1.60 ± 0.06	$p < 0.001$	NM_022654	p53 induced death domain protein 1
Pif1	0.04 ± 0.09	1.000	-0.49 ± 0.08	0.041	-1.83 ± 0.19	$p < 0.001$	NM_172453	PIF1 5-to-3 DNA helicase homolog (S. cerevisiae)
Pigv	0.29 ± 0.22	0.771	0.29 ± 0.13	0.494	1.20 ± 0.18	$p < 0.001$	NM_178698	phosphatidylinositol glycan anchor biosynthesis, class V
Pik3r2	-0.01 ± 0.26	1.000	-0.04 ± 0.26	1.000	-1.30 ± 0.26	$p < 0.001$	NM_008841	phosphatidylinositol 3-kinase, regulatory subunit, polypeptide 2 (p85 beta)
Pim1	0.91 ± 0.23	$p < 0.001$	1.40 ± 0.22	$p < 0.001$	2.80 ± 0.19	$p < 0.001$	NM_008842	proviral integration site 1
Pip5k1c	-0.06 ± 0.03	1.000	-0.40 ± 0.03	0.001	-1.39 ± 0.11	$p < 0.001$	NM_001146687	phosphatidylinositol-4-phosphate 5-kinase, type 1 gamma
Pisd	0.44 ± 0.32	0.245	0.20 ± 0.33	0.860	1.24 ± 0.33	$p < 0.001$	NM_177298	phosphatidylserine decarboxylase
Pitpna	-0.05 ± 0.08	1.000	-0.06 ± 0.10	1.000	-1.01 ± 0.06	$p < 0.001$	NM_008850	phosphatidylinositol transfer protein, alpha
Pkmyt1	-0.09 ± 0.23	1.000	-0.59 ± 0.08	$p < 0.001$	-1.17 ± 0.18	$p < 0.001$	NM_023058	protein kinase, membrane associated tyrosine/threonine 1
Pkp2	0.03 ± 0.10	1.000	0.33 ± 0.13	0.037	1.03 ± 0.08	$p < 0.001$	NM_026163	plakophilin 2
Pla2g5	0.08 ± 0.09	1.000	0.43 ± 0.13	0.173	1.23 ± 0.17	$p < 0.001$	ENSMUST00000102513	phospholipase A2, group V
Plau	-0.03 ± 0.08	1.000	-0.27 ± 0.11	0.001	-1.88 ± 0.10	$p < 0.001$	NM_008873	plasminogen activator, urokinase
Plekhn1	-0.01 ± 0.09	1.000	0.25 ± 0.09	0.339	1.67 ± 0.11	$p < 0.001$	NM_001008233	pleckstrin homology domain containing, family N member 1
Plk1	0.12 ± 0.00	0.966	-0.40 ± 0.02	$p < 0.001$	-2.91 ± 0.03	$p < 0.001$	NM_011121	polo-like kinase 1
Plk2	0.13 ± 0.02	0.723	0.11 ± 0.03	0.731	1.12 ± 0.04	$p < 0.001$	NM_152804	polo-like kinase 2
Plxna1	0.49 ± 0.15	$p < 0.001$	1.03 ± 0.15	$p < 0.001$	1.03 ± 0.15	$p < 0.001$	NM_008881	plexin A1
Plxnc1	-0.10 ± 0.21	1.000	0.24 ± 0.22	0.720	1.37 ± 0.24	$p < 0.001$	NM_018797	plexin C1
Pmaip1	0.06 ± 0.06	1.000	0.22 ± 0.21	0.603	1.05 ± 0.01	$p < 0.001$	NM_021451	phorbol-12-myristate-13-acetate-induced protein 1
Pmf1	-0.06 ± 0.20	1.000	-0.15 ± 0.21	0.617	-1.96 ± 0.21	$p < 0.001$	NM_025928	polyamine-modulated factor 1
Pml	0.45 ± 0.04	0.001	1.02 ± 0.05	$p < 0.001$	0.40 ± 0.04	0.003	NM_008884	promyelocytic leukemia
Pmvk	-0.34 ± 0.06	0.025	-0.80 ± 0.09	$p < 0.001$	-1.47 ± 0.06	$p < 0.001$	NM_026784	phosphomevalonate kinase
Pnn	-0.38 ± 0.22	0.004	-0.54 ± 0.22	$p < 0.001$	-1.31 ± 0.26	$p < 0.001$	NM_008891	pinin
Pnrc1	0.17 ± 0.06	1.000	0.41 ± 0.06	0.315	1.28 ± 0.18	$p < 0.001$	NM_001033225	proline-rich nuclear receptor coactivator 1
Pola2	-0.16 ± 0.03	0.777	-0.41 ± 0.07	0.001	-1.51 ± 0.04	$p < 0.001$	NM_001164057	polymerase (DNA directed), alpha 2
Pold1	-0.03 ± 0.07	1.000	-0.18 ± 0.08	0.494	-1.86 ± 0.30	$p < 0.001$	NM_011131	polymerase (DNA directed), delta 1, catalytic subunit
Pold2	-0.11 ± 0.10	1.000	-0.21 ± 0.10	0.270	-1.51 ± 0.19	$p < 0.001$	NM_008894	polymerase (DNA directed), delta 2, regulatory subunit
Pole	-0.05 ± 0.02	1.000	-0.14 ± 0.06	0.713	-1.31 ± 0.06	$p < 0.001$	NM_011132	polymerase (DNA directed), epsilon
Pole2	-0.32 ± 0.08	0.075	-0.23 ± 0.09	0.209	-2.29 ± 0.11	$p < 0.001$	NM_011133	polymerase (DNA directed), epsilon 2 (p59 subunit)

Polh	-0.28 ± 0.00	0.138	-0.53 ± 0.01	$p < 0.001$	-2.21 ± 0.20	$p < 0.001$	NM_030715	polymerase (DNA directed), eta (RAD 30 related)
Polq	-0.17 ± 0.03	1.000	-0.41 ± 0.03	0.050	-1.38 ± 0.23	$p < 0.001$	NM_001159369	polymerase (DNA directed), theta
Polr1b	0.04 ± 0.11	1.000	-0.45 ± 0.14	0.013	-1.03 ± 0.07	$p < 0.001$	NM_009086	polymerase (RNA) I polypeptide B
Polr3g	-0.08 ± 0.09	1.000	-0.54 ± 0.13	0.004	-1.74 ± 0.11	$p < 0.001$	NM_001081176	polymerase (RNA) III (DNA directed) polypeptide G
Pom121	0.03 ± 0.14	1.000	-0.28 ± 0.08	0.067	-1.21 ± 0.07	$p < 0.001$	NM_148932	nuclear pore membrane protein 121
Pomgnt1	-0.10 ± 0.01	1.000	-0.42 ± 0.02	0.014	-1.03 ± 0.01	$p < 0.001$	NM_001290658	protein O-linked mannose beta 1,2-N-acetylglucosaminyltransferase
Pop1	-0.14 ± 0.09	1.000	-0.25 ± 0.13	0.454	-1.71 ± 0.07	$p < 0.001$	NM_026340	processing of precursor 1, ribonuclease P/MRP family, (S. cerevisiae)
Pou2f2	0.66 ± 0.33	0.084	1.62 ± 0.26	$p < 0.001$	1.72 ± 0.27	$p < 0.001$	NM_001163554	POU domain, class 2, transcription factor 2
Ppan	-0.03 ± 0.01	1.000	-0.20 ± 0.08	0.351	-1.84 ± 0.04	$p < 0.001$	NM_145610	peter pan homolog (Drosophila)
Ppapdc1b	-0.06 ± 0.17	1.000	0.26 ± 0.08	0.103	1.03 ± 0.07	$p < 0.001$	NM_001293703	phosphatidic acid phosphatase type 2 domain containing 1B
Ppargc1b	-0.04 ± 0.06	1.000	-0.34 ± 0.16	0.448	-1.06 ± 0.33	$p < 0.001$	NM_133249	peroxisome proliferative activated receptor, gamma, coactivator 1 beta
Ppat	-0.12 ± 0.13	1.000	-0.36 ± 0.03	0.005	-1.63 ± 0.11	$p < 0.001$	NM_172146	phosphoribosyl pyrophosphate amidotransferase
Pphln1	-0.08 ± 0.06	1.000	-0.13 ± 0.07	0.781	-1.13 ± 0.07	$p < 0.001$	NM_001083114	periphilin 1
Ppif	-0.05 ± 0.03	1.000	-0.42 ± 0.04	$p < 0.001$	-1.42 ± 0.08	$p < 0.001$	NM_134084	peptidylprolyl isomerase F (cyclophilin F)
Ppil1	-0.26 ± 0.08	0.353	-0.62 ± 0.24	$p < 0.001$	-1.69 ± 0.10	$p < 0.001$	NM_026845	peptidylprolyl isomerase (cyclophilin)-like 1
Ppm1e	-0.32 ± 0.15	0.301	-0.56 ± 0.02	0.001	-1.01 ± 0.09	$p < 0.001$	NM_177167	protein phosphatase 1E (PP2C domain containing)
Ppm1g	-0.04 ± 0.02	1.000	-0.18 ± 0.08	0.342	-1.14 ± 0.03	$p < 0.001$	NM_008014	protein phosphatase 1G (formerly 2C), magnesium-dependent, gamma isoform
Ppm1k	0.42 ± 0.15	0.306	0.80 ± 0.15	$p < 0.001$	1.22 ± 0.16	$p < 0.001$	NM_175523	protein phosphatase 1K (PP2C domain containing)
Ppp1r15a	0.77 ± 0.22	0.002	1.15 ± 0.07	$p < 0.001$	3.97 ± 0.05	$p < 0.001$	NM_008654	protein phosphatase 1, regulatory (inhibitor) subunit 15A
Prc1	0.09 ± 0.05	1.000	-0.42 ± 0.05	$p < 0.001$	-1.73 ± 0.09	$p < 0.001$	NM_001285997	protein regulator of cytokinesis 1
Prdx5	0.29 ± 0.01	0.177	0.52 ± 0.01	$p < 0.001$	1.05 ± 0.04	$p < 0.001$	ENSMUST00000025904	peroxiredoxin 5
Prim1	-0.13 ± 0.16	1.000	-0.35 ± 0.10	0.009	-1.03 ± 0.38	$p < 0.001$	NM_008921	DNA primase, p49 subunit
Prim2	-0.08 ± 0.10	1.000	-0.21 ± 0.10	0.263	-1.13 ± 0.16	$p < 0.001$	NM_008922	DNA primase, p58 subunit
Prkar2a	-0.10 ± 0.04	1.000	-0.26 ± 0.07	0.097	-1.14 ± 0.09	$p < 0.001$	NM_008924	protein kinase, cAMP dependent regulatory, type II alpha
Prkar2b	-0.20 ± 0.14	1.000	-0.35 ± 0.14	0.544	-1.15 ± 0.22	$p < 0.001$	XM_006515014	protein kinase, cAMP dependent regulatory, type II beta
Prnp	-0.01 ± 0.12	1.000	0.25 ± 0.08	0.534	1.27 ± 0.11	$p < 0.001$	NM_001278256	prion protein
Prpf19	-0.01 ± 0.05	1.000	-0.16 ± 0.03	0.468	-1.25 ± 0.16	$p < 0.001$	NM_001253843	PRP19/PSO4 pre-mRNA processing factor 19 homolog (S. cerevisiae)
Prpf31	-0.04 ± 0.05	1.000	-0.34 ± 0.10	0.005	-1.40 ± 0.14	$p < 0.001$	NM_001159714	PRP31 pre-mRNA processing factor 31 homolog (yeast)
Prpf4	-0.24 ± 0.09	0.379	-0.59 ± 0.11	$p < 0.001$	-1.30 ± 0.13	$p < 0.001$	NM_027297	PRP4 pre-mRNA processing factor 4 homolog (yeast)
Prps2	-0.04 ± 0.12	1.000	-0.21 ± 0.12	0.303	-1.87 ± 0.12	$p < 0.001$	NM_026662	phosphoribosyl pyrophosphate synthetase 2
Prr11	0.31 ± 0.13	0.036	0.02 ± 0.05	1.000	-1.07 ± 0.04	$p < 0.001$	NM_175563	proline rich 11
Prrg4	0.04 ± 0.13	1.000	0.03 ± 0.12	1.000	1.55 ± 0.11	$p < 0.001$	NM_178695	proline rich Gla (G-carboxyglutamic acid) 4 (transmembrane)
Psat1	0.15 ± 0.08	0.799	0.22 ± 0.12	0.160	-1.05 ± 0.10	$p < 0.001$	NM_177420	phosphoserine aminotransferase 1
Psmd1	-0.15 ± 0.13	0.786	-0.17 ± 0.16	0.379	-1.05 ± 0.13	$p < 0.001$	NM_027357	proteasome (prosome, macropain) 26S subunit, non-ATPase, 1
Psmd10	0.31 ± 0.08	0.143	0.67 ± 0.11	$p < 0.001$	1.41 ± 0.07	$p < 0.001$	NM_001164177	proteasome (prosome, macropain) 26S subunit, non-ATPase, 10
Pspc1	-0.15 ± 0.12	1.000	-0.58 ± 0.06	$p < 0.001$	-1.27 ± 0.24	$p < 0.001$	NM_025682	paraspeckle protein 1
Ptcd3	0.02 ± 0.06	1.000	-0.25 ± 0.03	0.107	-1.30 ± 0.05	$p < 0.001$	NM_027275	pentatricopeptide repeat domain 3
Ptger4	-0.16 ± 0.06	1.000	-0.17 ± 0.12	0.951	-1.11 ± 0.12	$p < 0.001$	NM_001136079	prostaglandin E receptor 4 (subtype EP4)
Ptges	0.55 ± 0.21	0.322	1.02 ± 0.36	$p < 0.001$	1.78 ± 0.34	$p < 0.001$	XM_006498216	prostaglandin E synthase
Ptgir	0.22 ± 0.13	0.494	0.35 ± 0.13	0.011	1.45 ± 0.13	$p < 0.001$	NM_008967	prostaglandin I receptor (IP)
Ptgs2	1.19 ± 0.10	$p < 0.001$	2.03 ± 0.08	$p < 0.001$	5.02 ± 0.07	$p < 0.001$	NM_011198	prostaglandin-endoperoxide synthase 2

Ptgs2os2	0.45 ± 0.22	0.549	0.68 ± 0.23	0.020	1.74 ± 0.19	p < 0.001	NR_110420	prostaglandin-endoperoxide synthase 2, opposite strand 2
Ptma	-0.05 ± 0.05	1.000	0.00 ± 0.11	1.000	-1.26 ± 0.02	p < 0.001	NM_008972	prothymosin alpha
Ptms	-0.29 ± 0.04	0.646	-0.49 ± 0.06	0.026	-1.03 ± 0.01	p < 0.001	NM_026988	parathymosin
Ptov1	-0.24 ± 0.17	0.562	-0.29 ± 0.07	0.149	-1.17 ± 0.19	p < 0.001	NM_133949	prostate tumor over expressed gene 1
Ptprj	0.34 ± 0.06	0.030	0.83 ± 0.07	p < 0.001	1.10 ± 0.09	p < 0.001	NM_001135657	protein tyrosine phosphatase, receptor type, J
Pttg1	0.40 ± 0.09	0.026	0.30 ± 0.08	0.089	-1.06 ± 0.50	p < 0.001	NM_001131054	pituitary tumor-transforming gene 1
Pvrl3	-0.10 ± 0.13	1.000	-0.35 ± 0.11	0.293	-1.13 ± 0.36	p < 0.001	NM_021495	poliovirus receptor-related 3
Pygb	-0.13 ± 0.09	1.000	-0.25 ± 0.12	0.143	-1.88 ± 0.12	p < 0.001	NM_153781	brain glycogen phosphorylase
Pyhin1	0.44 ± 0.70	0.794	2.41 ± 0.69	p < 0.001	3.18 ± 0.72	p < 0.001	NM_175026	pyrin and HIN domain family, member 1
Qpct	-0.01 ± 0.23	1.000	0.32 ± 0.14	0.407	1.24 ± 0.16	p < 0.001	NM_027455	glutaminyl-peptide cyclotransferase (glutaminyl cyclase)
Rab11fip1	0.17 ± 0.17	1.000	0.56 ± 0.19	0.012	2.33 ± 0.23	p < 0.001	NM_001080813	RAB11 family interacting protein 1 (class I)
Rab20	0.37 ± 0.13	0.375	0.43 ± 0.11	0.087	1.24 ± 0.10	p < 0.001	ENSMUST00000033900	RAB20, member RAS oncogene family
Rab26os	-0.22 ± 0.07	0.488	-1.19 ± 0.08	p < 0.001	-2.03 ± 0.34	p < 0.001	NR_045289	RAB26, member RAS oncogene family, opposite strand
Rab27a	-0.17 ± 0.18	1.000	-0.27 ± 0.20	0.338	-1.18 ± 0.31	p < 0.001	NM_023635	RAB27A, member RAS oncogene family
Rab30	0.21 ± 0.26	1.000	0.20 ± 0.26	0.999	1.28 ± 0.26	p < 0.001	NM_029494	RAB30, member RAS oncogene family
Rab3a	-0.14 ± 0.07	1.000	-0.41 ± 0.19	0.240	-1.47 ± 0.17	p < 0.001	NM_001166399	RAB3A, member RAS oncogene family
Rab3il1	-0.06 ± 0.10	1.000	-0.06 ± 0.12	1.000	-1.11 ± 0.12	p < 0.001	XM_006527412	RAB3A interacting protein (rabin3)-like 1
Rab8b	0.23 ± 0.14	0.367	0.19 ± 0.05	0.380	1.14 ± 0.04	p < 0.001	NM_173413	RAB8B, member RAS oncogene family
Racgap1	0.08 ± 0.08	1.000	-0.27 ± 0.08	0.069	-1.49 ± 0.26	p < 0.001	NM_001253808	Rac GTPase-activating protein 1
Rad51	-0.07 ± 0.12	1.000	-0.22 ± 0.12	0.357	-1.32 ± 0.23	p < 0.001	NM_011234	RAD51 homolog
Rad51ap1	-0.19 ± 0.24	1.000	-0.52 ± 0.19	0.020	-1.21 ± 0.36	p < 0.001	NM_009013	RAD51 associated protein 1
Rad54b	-0.24 ± 0.06	1.000	-0.18 ± 0.07	0.912	-1.24 ± 0.06	p < 0.001	NM_001039556	RAD54 homolog B (S. cerevisiae)
Rad54l	-0.19 ± 0.03	0.575	-0.39 ± 0.03	0.001	-2.53 ± 0.14	p < 0.001	NM_001122958	RAD54 like (S. cerevisiae)
Ralgapa2	0.21 ± 0.17	1.000	0.47 ± 0.14	0.082	1.03 ± 0.15	p < 0.001	NM_001033348	Ral GTPase activating protein, alpha subunit 2 (catalytic)
Rangap1	0.00 ± 0.06	1.000	-0.29 ± 0.09	0.037	-1.51 ± 0.04	p < 0.001	NM_011241	RAN GTPase activating protein 1
Rapgef2	0.96 ± 0.08	p < 0.001	1.45 ± 0.08	p < 0.001	2.13 ± 0.07	p < 0.001	XM_006502252	Rap guanine nucleotide exchange factor (GEF) 2
Rapgef5	0.40 ± 0.17	0.296	0.68 ± 0.23	0.001	1.02 ± 0.17	p < 0.001	NM_175930	Rap guanine nucleotide exchange factor (GEF) 5
Rasa3	-0.08 ± 0.09	1.000	-0.12 ± 0.10	0.789	-1.26 ± 0.16	p < 0.001	NM_009025	RAS p21 protein activator 3
Rasgef1b	0.07 ± 0.04	1.000	0.34 ± 0.12	0.025	2.07 ± 0.05	p < 0.001	NM_145839	RasGEF domain family, member 1B
Rasgrp3	-0.16 ± 0.04	0.453	-0.48 ± 0.06	p < 0.001	-1.63 ± 0.09	p < 0.001	NM_001166493	RAS, guanyl releasing protein 3
Rasgrp4	0.03 ± 0.31	1.000	0.43 ± 0.27	0.235	1.08 ± 0.15	p < 0.001	NM_001174155	RAS guanyl releasing protein 4
Rassf2	-0.11 ± 0.04	1.000	-0.21 ± 0.11	0.431	-1.05 ± 0.14	p < 0.001	NM_175445	Ras association (RalGDS/AF-6) domain family member 2
Rassf4	0.49 ± 0.06	p < 0.001	1.27 ± 0.06	p < 0.001	2.62 ± 0.11	p < 0.001	NM_178045	Ras association (RalGDS/AF-6) domain family member 4
Rassf8	0.07 ± 0.05	1.000	-0.19 ± 0.14	0.345	-1.12 ± 0.03	p < 0.001	ENSMUST00000111704	Ras association (RalGDS/AF-6) domain family (N-terminal) member 8
Rbl1	-0.13 ± 0.06	0.945	-0.16 ± 0.06	0.499	-1.22 ± 0.13	p < 0.001	NM_011249	retinoblastoma-like 1 (p107)
Rbm14	-0.25 ± 0.05	0.159	-0.64 ± 0.06	p < 0.001	-1.55 ± 0.07	p < 0.001	NM_019869	RNA binding motif protein 14
Rbmxl1	-0.08 ± 0.08	1.000	-0.26 ± 0.17	0.077	-1.06 ± 0.13	p < 0.001	NM_009033	RNA binding motif protein, X linked-like-1
Rbpj	0.20 ± 0.13	1.000	0.46 ± 0.09	0.054	1.19 ± 0.01	p < 0.001	ENSMUST00000037618	recombination signal binding protein for immunoglobulin kappa J region
Rcan1	-0.16 ± 0.11	1.000	-0.57 ± 0.10	0.006	1.21 ± 0.16	p < 0.001	NM_001081549	regulator of calcineurin 1
Rchy1	-0.01 ± 0.05	1.000	0.16 ± 0.14	0.661	1.25 ± 0.17	p < 0.001	NM_001271797	ring finger and CHY zinc finger domain containing 1
Rcn1	-0.09 ± 0.18	1.000	0.04 ± 0.06	1.000	-1.00 ± 0.04	p < 0.001	NM_009037	reticulocalbin 1

Rdh11	-0.13 ± 0.03	1.000	-0.29 ± 0.06	0.045	-1.12 ± 0.05	$p < 0.001$	NM_021557	retinol dehydrogenase 11
Recql	0.08 ± 0.06	1.000	-0.26 ± 0.05	0.374	-1.03 ± 0.27	$p < 0.001$	NM_001204906	RecQ protein-like
Reep4	0.20 ± 0.06	0.454	-0.23 ± 0.11	0.162	-1.33 ± 0.09	$p < 0.001$	NM_180588	receptor accessory protein 4
Rel	0.32 ± 0.04	0.031	0.61 ± 0.05	$p < 0.001$	1.92 ± 0.09	$p < 0.001$	NM_009044	reticuloendotheliosis oncogene
Relb	0.52 ± 0.04	0.027	0.52 ± 0.06	0.010	1.12 ± 0.07	$p < 0.001$	ENSMUST00000131759	avian reticuloendotheliosis viral (v-rel) oncogene related B
Rfc1	-0.12 ± 0.06	1.000	-0.17 ± 0.06	0.463	-1.01 ± 0.21	$p < 0.001$	NM_011258	replication factor C (activator 1) 1
Rfc2	-0.15 ± 0.06	0.981	-0.30 ± 0.12	0.057	-1.32 ± 0.30	$p < 0.001$	NM_020022	replication factor C (activator 1) 2
Rfc5	-0.06 ± 0.09	1.000	-0.35 ± 0.08	0.011	-1.19 ± 0.08	$p < 0.001$	ENSMUST00000086461	replication factor C (activator 1) 5
Rfwd2	0.02 ± 0.03	1.000	0.13 ± 0.03	0.737	1.03 ± 0.08	$p < 0.001$	NM_011931	ring finger and WD repeat domain 2
Rfwd3	-0.08 ± 0.05	1.000	-0.32 ± 0.02	0.012	-1.56 ± 0.12	$p < 0.001$	NM_146218	ring finger and WD repeat domain 3
Rgcc	-0.81 ± 0.08	0.018	-0.66 ± 0.16	0.031	-1.23 ± 0.08	$p < 0.001$	NM_025427	regulator of cell cycle
Rgl1	0.24 ± 0.10	0.692	0.50 ± 0.11	0.002	1.22 ± 0.11	$p < 0.001$	NM_016846	ral guanine nucleotide dissociation stimulator,-like 1
Rgs1	-0.07 ± 0.02	1.000	0.00 ± 0.03	1.000	1.23 ± 0.19	$p < 0.001$	NM_015811	regulator of G-protein signaling 1
Rgs18	0.20 ± 0.08	0.653	0.32 ± 0.08	0.042	1.30 ± 0.23	$p < 0.001$	NM_022881	regulator of G-protein signaling 18
Rhbdd1	0.04 ± 0.07	1.000	0.28 ± 0.04	0.070	1.35 ± 0.04	$p < 0.001$	NM_001122685	rhomboid domain containing 1
Rhob	0.34 ± 0.13	0.778	0.19 ± 0.09	0.927	1.99 ± 0.09	$p < 0.001$	NM_007483	ras homolog gene family, member B
Rhoc	0.19 ± 0.09	0.475	0.29 ± 0.11	0.029	1.07 ± 0.19	$p < 0.001$	NM_001291859	ras homolog gene family, member C
Rhod	0.11 ± 0.05	1.000	0.06 ± 0.08	1.000	2.10 ± 0.05	$p < 0.001$	NM_007485	ras homolog gene family, member D
Rhoq	0.24 ± 0.11	0.864	0.66 ± 0.07	$p < 0.001$	1.65 ± 0.14	$p < 0.001$	NM_145491	ras homolog gene family, member Q
Rilpl2	0.44 ± 0.20	0.234	1.26 ± 0.13	$p < 0.001$	1.75 ± 0.13	$p < 0.001$	NM_030259	Rab interacting lysosomal protein-like 2
Rin3	0.01 ± 0.07	1.000	-0.17 ± 0.11	0.879	-1.11 ± 0.19	$p < 0.001$	NM_001161365	Ras and Rab interactor 3
Riok3	0.01 ± 0.03	1.000	0.25 ± 0.03	0.118	1.28 ± 0.06	$p < 0.001$	NM_024182	RIO kinase 3
Rit1	-0.22 ± 0.05	0.599	0.08 ± 0.08	0.995	1.22 ± 0.02	$p < 0.001$	NM_001163310	Ras-like without CAAX 1
Rnf114	0.46 ± 0.16	0.004	1.04 ± 0.15	$p < 0.001$	1.29 ± 0.11	$p < 0.001$	NM_030743	ring finger protein 114
Rnf126	0.04 ± 0.03	1.000	0.00 ± 0.06	1.000	-1.22 ± 0.10	$p < 0.001$	NM_144528	ring finger protein 126
Rnf149	0.32 ± 0.06	0.066	0.53 ± 0.06	$p < 0.001$	1.17 ± 0.03	$p < 0.001$	NM_001033135	ring finger protein 149
Rnf19b	0.57 ± 0.02	$p < 0.001$	0.81 ± 0.08	$p < 0.001$	1.26 ± 0.06	$p < 0.001$	NM_029219	ring finger protein 19B
Rnf213	0.82 ± 0.01	$p < 0.001$	2.29 ± 0.03	$p < 0.001$	1.89 ± 0.01	$p < 0.001$	ENSMUST00000093902	ring finger protein 213
Rpa1	-0.20 ± 0.02	0.421	-0.41 ± 0.01	$p < 0.001$	-1.87 ± 0.09	$p < 0.001$	NM_001164223	replication protein A1
Rpa2	-0.16 ± 0.11	0.796	-0.39 ± 0.12	0.002	-1.87 ± 0.13	$p < 0.001$	NM_011284	replication protein A2
Rpa3	-0.06 ± 0.23	1.000	-0.40 ± 0.40	0.049	-1.07 ± 0.24	$p < 0.001$	NM_026632	replication protein A3
Rpl18a	0.04 ± 0.19	1.000	-0.26 ± 0.09	0.877	-1.01 ± 0.38	0.001	NM_029751	ribosomal protein L18A
Rpl21	-0.14 ± 0.12	1.000	-0.71 ± 0.14	0.006	-1.02 ± 0.16	$p < 0.001$	XM_006504815	ribosomal protein L21
Rpl7a-ps8	0.62 ± 0.22	0.508	0.14 ± 0.43	1.000	1.36 ± 0.62	$p < 0.001$	ENSMUST00000094754	ribosomal protein L7A, pseudogene 8
Rpusd1	-0.19 ± 0.30	1.000	-0.47 ± 0.15	0.025	-1.33 ± 0.57	$p < 0.001$	NM_028009	RNA pseudouridylate synthase domain containing 1
Rrm1	-0.12 ± 0.02	0.909	-0.40 ± 0.03	$p < 0.001$	-1.48 ± 0.21	$p < 0.001$	NM_009103	ribonucleotide reductase M1
Rrm2	-0.20 ± 0.11	0.416	-0.67 ± 0.06	$p < 0.001$	-2.29 ± 0.13	$p < 0.001$	NM_009104	ribonucleotide reductase M2
Rrp1b	0.07 ± 0.05	1.000	-0.19 ± 0.02	0.501	-1.03 ± 0.11	$p < 0.001$	NM_001163734	ribosomal RNA processing 1 homolog B (S. cerevisiae)
Rsad2	0.61 ± 0.17	0.070	1.98 ± 0.15	$p < 0.001$	3.50 ± 0.19	$p < 0.001$	ENSMUST00000137792	radical S-adenosyl methionine domain containing 2
Rtel1	-0.10 ± 0.05	1.000	-0.37 ± 0.06	0.012	-1.11 ± 0.13	$p < 0.001$	NM_001001882	regulator of telomere elongation helicase 1
Rtp4	1.87 ± 0.16	$p < 0.001$	2.60 ± 0.16	$p < 0.001$	3.21 ± 0.15	$p < 0.001$	NM_023386	receptor transporter protein 4

S100a4	-0.08 ± 0.10	1.000	-0.31 ± 0.07	0.004	-2.57 ± 0.13	p < 0.001	NM_011311	S100 calcium binding protein A4
Saa3	0.30 ± 0.34	0.846	1.62 ± 0.23	p < 0.001	4.63 ± 0.28	p < 0.001	NM_011315	serum amyloid A 3
Samd1	0.12 ± 0.09	1.000	-0.06 ± 0.11	1.000	-1.20 ± 0.11	p < 0.001	NM_001081415	sterile alpha motif domain containing 1
Samd9l	0.68 ± 0.23	0.007	1.40 ± 0.23	p < 0.001	1.90 ± 0.23	p < 0.001	NM_010156	sterile alpha motif domain containing 9-like
Samsn1	0.33 ± 0.06	0.664	0.59 ± 0.10	0.009	1.46 ± 0.07	p < 0.001	NM_023380	SAM domain, SH3 domain and nuclear localization signals, 1
Sapcd2	0.14 ± 0.12	1.000	-0.28 ± 0.13	0.456	-1.04 ± 0.09	p < 0.001	NM_001081085	suppressor APC domain containing 2
Sat1	0.26 ± 0.07	0.223	0.87 ± 0.11	p < 0.001	1.97 ± 0.11	p < 0.001	NM_001291865	spermidine/spermine N1-acetyl transferase 1
Scarb1	-0.07 ± 0.03	1.000	-0.29 ± 0.06	0.030	-1.40 ± 0.03	p < 0.001	NM_001205082	scavenger receptor class B, member 1
Scd2	-0.26 ± 0.09	0.004	-0.40 ± 0.06	p < 0.001	-2.31 ± 0.05	p < 0.001	NM_009128	stearoyl-Coenzyme A desaturase 2
Scd3	-0.82 ± 0.41	0.014	-1.07 ± 0.29	p < 0.001	-1.45 ± 0.53	p < 0.001	NM_024450	stearoyl-coenzyme A desaturase 3
Scimp	0.43 ± 0.39	0.763	0.77 ± 0.12	0.021	1.24 ± 0.15	p < 0.001	NM_001045526	SLP adaptor and CSK interacting membrane protein
Scnm1	0.16 ± 0.09	0.846	0.20 ± 0.09	0.347	1.52 ± 0.09	p < 0.001	NM_001163573	sodium channel modifier 1
Sdc3	0.20 ± 0.27	0.995	1.44 ± 0.12	p < 0.001	1.54 ± 0.10	p < 0.001	NM_011520	syndecan 3
Sdf2l1	-0.17 ± 0.01	0.633	-0.19 ± 0.03	0.272	-1.22 ± 0.03	p < 0.001	NM_022324	stromal cell-derived factor 2-like 1
Sele	-0.08 ± 0.20	1.000	0.10 ± 0.22	1.000	1.84 ± 0.05	p < 0.001	NM_011345	selectin, endothelial cell
Sephs2	0.14 ± 0.02	0.853	0.15 ± 0.04	0.525	-1.11 ± 0.18	p < 0.001	NM_009266	selenophosphate synthetase 2
Sept6	-0.25 ± 0.25	1.000	-0.17 ± 0.24	0.958	-1.14 ± 0.27	p < 0.001	NM_001253706	septin 6
Sepw1	-0.20 ± 0.17	0.925	-0.05 ± 0.25	1.000	1.32 ± 0.07	p < 0.001	NM_009156	selenoprotein W, muscle 1
Serinc2	-0.12 ± 0.18	1.000	-0.63 ± 0.17	p < 0.001	-1.17 ± 0.18	p < 0.001	XM_006536985	serine incorporator 2
Serpinb1a	-0.65 ± 0.15	p < 0.001	-1.74 ± 0.16	p < 0.001	-3.10 ± 0.41	p < 0.001	NM_025429	serine (or cysteine) peptidase inhibitor, clade B, member 1a
Serpinb7	-0.13 ± 0.31	1.000	-0.07 ± 0.40	1.000	2.00 ± 0.39	p < 0.001	NM_027548	serine (or cysteine) peptidase inhibitor, clade B, member 7
Serpinb9b	-0.75 ± 0.14	p < 0.001	-1.32 ± 0.15	p < 0.001	-1.13 ± 0.10	p < 0.001	NM_011452	serine (or cysteine) peptidase inhibitor, clade B, member 9b
Set	-0.07 ± 0.09	1.000	-0.04 ± 0.10	1.000	-1.18 ± 0.36	p < 0.001	ENSMUST00000102866	SET nuclear oncogene
Sfpq	-0.09 ± 0.09	1.000	-0.40 ± 0.10	0.021	-1.11 ± 0.15	p < 0.001	NM_023603	splicing factor proline/glutamine rich (polypyrimidine tract binding protein associated)
Sfxn1	-0.24 ± 0.03	0.196	-0.33 ± 0.12	0.008	-1.07 ± 0.04	p < 0.001	NM_027324	sideroflexin 1
Sfxn2	-0.09 ± 0.11	1.000	0.08 ± 0.13	1.000	-1.50 ± 0.18	p < 0.001	NM_053196	sideroflexin 2
Sgol1	0.11 ± 0.09	1.000	-0.28 ± 0.11	0.416	-1.43 ± 0.18	p < 0.001	NM_028232	shugoshin-like 1 (S. pombe)
Sgol2	0.25 ± 0.09	1.000	-0.34 ± 0.17	0.469	-1.67 ± 0.33	p < 0.001	NM_001177867	shugoshin-like 2 (S. pombe)
Sh2b3	0.25 ± 0.10	0.222	-0.01 ± 0.09	1.000	-1.32 ± 0.10	p < 0.001	ENSMUST00000086310	SH2B adaptor protein 3
Sh2d6	0.39 ± 0.27	0.340	1.40 ± 0.30	p < 0.001	3.68 ± 0.18	p < 0.001	XM_006506832	SH2 domain containing 6
Shcbp1	0.17 ± 0.06	0.782	-0.17 ± 0.10	0.557	-1.73 ± 0.54	p < 0.001	NM_011369	Shc SH2-domain binding protein 1
Shmt1	0.08 ± 0.08	1.000	0.07 ± 0.14	1.000	-1.10 ± 0.10	p < 0.001	NM_009171	serine hydroxymethyltransferase 1 (soluble)
Shmt2	0.20 ± 0.03	0.109	0.11 ± 0.04	0.534	-1.03 ± 0.04	p < 0.001	NM_001252316	serine hydroxymethyltransferase 2 (mitochondrial)
Siglece	-0.15 ± 0.10	1.000	-0.21 ± 0.16	0.980	1.19 ± 0.02	p < 0.001	NM_031181	sialic acid binding Ig-like lectin E
Sigmar1	-0.05 ± 0.13	1.000	-0.46 ± 0.10	0.014	-1.56 ± 0.10	p < 0.001	NM_001286538	sigma non-opioid intracellular receptor 1
Sin3a	-0.07 ± 0.06	1.000	-0.04 ± 0.02	1.000	-1.15 ± 0.02	p < 0.001	NM_001110350	transcriptional regulator, SIN3A (yeast)
Sirt2	0.15 ± 0.04	0.846	0.25 ± 0.04	0.103	1.15 ± 0.03	p < 0.001	NM_001122765	sirtuin 2
Sirt6	0.09 ± 0.12	1.000	0.29 ± 0.15	0.069	1.11 ± 0.14	p < 0.001	NM_001163430	sirtuin 6
Ska2	0.02 ± 0.21	1.000	-0.27 ± 0.21	0.104	-1.46 ± 0.30	p < 0.001	NM_025377	spindle and kinetochore associated complex subunit 2
Ska3	-0.11 ± 0.02	1.000	-0.49 ± 0.13	0.009	-1.33 ± 0.17	p < 0.001	NM_198605	spindle and kinetochore associated complex subunit 3
Skil	0.68 ± 0.08	0.031	0.93 ± 0.13	p < 0.001	1.86 ± 0.22	p < 0.001	NM_001039090	SKI-like

Skp2	-0.23 ± 0.11	0.273	-0.54 ± 0.12	p < 0.001	-1.35 ± 0.15	p < 0.001	NM_001285980	S-phase kinase-associated protein 2 (p45)
Sla	0.23 ± 0.24	1.000	0.84 ± 0.21	0.001	1.68 ± 0.21	p < 0.001	NM_009192	src-like adaptor
Slbp	-0.20 ± 0.09	0.523	-0.22 ± 0.17	0.212	-1.33 ± 0.14	p < 0.001	NM_001289724	stem-loop binding protein
Slc10a3	-0.08 ± 0.14	1.000	-0.45 ± 0.14	0.005	-1.31 ± 0.26	p < 0.001	NM_001256104	solute carrier family 10 (sodium/bile acid cotransporter family), member 3
Slc11a2	0.54 ± 0.03	p < 0.001	0.87 ± 0.04	p < 0.001	1.64 ± 0.01	p < 0.001	NM_001146161	solute carrier family 11 (proton-coupled divalent metal ion transporters), member 2
Slc15a3	0.86 ± 0.22	p < 0.001	1.69 ± 0.23	p < 0.001	3.75 ± 0.22	p < 0.001	NM_023044	solute carrier family 15, member 3
Slc16a13	0.05 ± 0.08	1.000	-0.18 ± 0.12	0.465	-1.27 ± 0.30	p < 0.001	NM_172371	solute carrier family 16 (monocarboxylic acid transporters), member 13
Slc16a3	-0.20 ± 0.04	0.396	-0.18 ± 0.05	0.375	-1.16 ± 0.04	p < 0.001	ENSMUST00000168579	solute carrier family 16 (monocarboxylic acid transporters), member 3
Slc16a6	0.08 ± 0.10	1.000	-0.17 ± 0.20	0.492	-1.33 ± 0.14	p < 0.001	NM_001029842	solute carrier family 16 (monocarboxylic acid transporters), member 6
Slc25a10	-0.42 ± 0.10	0.084	-0.54 ± 0.08	0.003	-1.92 ± 0.26	p < 0.001	NM_013770	solute carrier family 25 (mitochondrial carrier, dicarboxylate transporter), member 10
Slc25a13	-0.05 ± 0.05	1.000	-0.22 ± 0.03	0.190	-1.24 ± 0.10	p < 0.001	NM_001177572	solute carrier family 25 (mitochondrial carrier, adenine nucleotide translocator), member 13
Slc25a33	0.27 ± 0.14	0.658	0.68 ± 0.05	p < 0.001	1.31 ± 0.19	p < 0.001	NM_027460	solute carrier family 25, member 33
Slc25a37	0.12 ± 0.02	1.000	0.53 ± 0.15	p < 0.001	1.37 ± 0.03	p < 0.001	NM_026331	solute carrier family 25, member 37
Slc29a2	-0.20 ± 0.08	0.989	-0.67 ± 0.17	p < 0.001	-1.45 ± 0.10	p < 0.001	NM_007854	solute carrier family 29 (nucleoside transporters), member 2
Slc31a2	0.39 ± 0.04	0.002	1.05 ± 0.10	p < 0.001	1.74 ± 0.03	p < 0.001	NM_025286	solute carrier family 31, member 2
Slc35a4	-0.14 ± 0.13	1.000	-0.54 ± 0.13	0.001	-1.00 ± 0.15	p < 0.001	NM_001083317	solute carrier family 35, member A4
Slc35g1	-0.32 ± 0.18	0.186	-0.68 ± 0.27	p < 0.001	-1.29 ± 0.25	p < 0.001	NM_175507	solute carrier family 35, member G1
Slc37a2	-0.71 ± 0.08	p < 0.001	-1.16 ± 0.12	p < 0.001	-0.67 ± 0.09	p < 0.001	NM_001145960	solute carrier family 37 (glycerol-3-phosphate transporter), member 2
Slc37a4	-0.25 ± 0.11	0.479	-0.22 ± 0.11	0.357	-1.35 ± 0.14	p < 0.001	NM_001293630	solute carrier family 37 (glucose-6-phosphate transporter), member 4
Slc38a7	0.28 ± 0.08	0.113	0.29 ± 0.04	0.061	1.41 ± 0.05	p < 0.001	NM_172758	solute carrier family 38, member 7
Slc39a2	0.03 ± 0.34	1.000	0.14 ± 0.29	1.000	3.02 ± 0.29	p < 0.001	NM_001039676	solute carrier family 39 (zinc transporter), member 2
Slc40a1	-0.93 ± 0.06	p < 0.001	-1.08 ± 0.06	p < 0.001	-0.74 ± 0.12	p < 0.001	NM_016917	solute carrier family 40 (iron-regulated transporter), member 1
Slc43a3	0.13 ± 0.08	1.000	0.54 ± 0.09	p < 0.001	1.47 ± 0.10	p < 0.001	NM_021398	solute carrier family 43, member 3
Slc46a3	0.13 ± 0.09	1.000	0.69 ± 0.15	p < 0.001	2.18 ± 0.02	p < 0.001	NM_027872	solute carrier family 46, member 3
Slc4a7	0.56 ± 0.10	p < 0.001	1.11 ± 0.08	p < 0.001	1.35 ± 0.07	p < 0.001	NM_001033270	solute carrier family 4, sodium bicarbonate cotransporter, member 7
Slc5a6	0.05 ± 0.08	1.000	-0.36 ± 0.12	0.265	-1.43 ± 0.57	p < 0.001	NM_001177621	solute carrier family 5 (sodium-dependent vitamin transporter), member 6
Slc7a11	0.29 ± 0.08	0.090	0.66 ± 0.10	p < 0.001	1.18 ± 0.06	p < 0.001	NM_011990	solute carrier family 7 (cationic amino acid transporter, y+ system), member 11
Slc7a7	0.47 ± 0.18	0.089	0.81 ± 0.11	p < 0.001	1.97 ± 0.02	p < 0.001	NM_001253680	solute carrier family 7 (cationic amino acid transporter, y+ system), member 7
Slc7a8	0.29 ± 0.06	0.219	0.77 ± 0.06	p < 0.001	1.61 ± 0.03	p < 0.001	NM_016972	solute carrier family 7 (cationic amino acid transporter, y+ system), member 8
Slc9a4	0.05 ± 0.23	1.000	0.17 ± 0.31	0.837	1.71 ± 0.22	p < 0.001	NM_177084	solute carrier family 9 (sodium/hydrogen exchanger), member 4
Slc9a9	0.41 ± 0.34	0.504	0.56 ± 0.09	0.043	2.09 ± 0.11	p < 0.001	NM_177909	solute carrier family 9 (sodium/hydrogen exchanger), member 9
Slco4a1	-0.11 ± 0.11	1.000	-0.19 ± 0.12	0.402	-2.02 ± 0.16	p < 0.001	NM_148933	solute carrier organic anion transporter family, member 4a1
Slfn10-ps	0.25 ± 0.35	1.000	0.45 ± 0.35	0.105	1.81 ± 0.40	p < 0.001	NR_073523	schlafen 10, pseudogene
Slfn2	1.01 ± 0.03	p < 0.001	1.54 ± 0.07	p < 0.001	2.24 ± 0.13	p < 0.001	NM_011408	schlafen 2
Slfn4	0.80 ± 0.18	p < 0.001	2.05 ± 0.07	p < 0.001	3.41 ± 0.06	p < 0.001	NM_011410	schlafen 4
Slfn5	1.00 ± 0.11	p < 0.001	1.77 ± 0.10	p < 0.001	1.66 ± 0.08	p < 0.001	NM_183201	schlafen 5
Slfn9	0.10 ± 0.11	1.000	0.35 ± 0.15	0.010	-1.07 ± 0.11	p < 0.001	NM_172796	schlafen 9
Slpi	-0.04 ± 0.26	1.000	0.43 ± 0.29	0.392	1.05 ± 0.33	p < 0.001	NM_011414	secretory leukocyte peptidase inhibitor
Smad7	0.33 ± 0.34	1.000	0.09 ± 0.35	1.000	1.62 ± 0.22	p < 0.001	NM_001042660	SMAD family member 7
Smarcc1	0.05 ± 0.07	1.000	0.01 ± 0.08	1.000	-1.42 ± 0.06	p < 0.001	NM_009211	SWI/SNF related, matrix associated, actin dependent regulator of chromatin, subfamily c, member 1
Smarcd2	-0.16 ± 0.15	1.000	-0.22 ± 0.15	0.836	-1.24 ± 0.16	p < 0.001	NM_001130187	SWI/SNF related, matrix associated, actin dependent regulator of chromatin, subfamily d, member 2

Smc2	-0.02 ± 0.01	1.000	-0.25 ± 0.04	0.092	-1.56 ± 0.14	$p < 0.001$	NM_008017	structural maintenance of chromosomes 2
Smg9	0.09 ± 0.07	1.000	0.26 ± 0.13	0.336	1.19 ± 0.07	$p < 0.001$	NM_028047	smg-9 homolog, nonsense mediated mRNA decay factor (C. elegans)
Smim3	0.15 ± 0.06	1.000	0.61 ± 0.09	0.001	1.96 ± 0.17	$p < 0.001$	NM_134133	small integral membrane protein 3
Smox	0.09 ± 0.11	1.000	0.49 ± 0.11	0.004	1.53 ± 0.12	$p < 0.001$	NM_001177833	spermine oxidase
Smpdl3b	0.02 ± 0.07	1.000	-0.01 ± 0.07	1.000	-1.52 ± 0.15	$p < 0.001$	NM_133888	sphingomyelin phosphodiesterase, acid-like 3B
Sms	-0.47 ± 0.20	0.273	-0.34 ± 0.32	0.413	-1.27 ± 0.27	$p < 0.001$	XM_006528775	spermine synthase
Snhg12	-0.20 ± 0.04	1.000	-0.53 ± 0.07	0.078	1.65 ± 0.16	$p < 0.001$	NR_029468	small nucleolar RNA host gene 12
Snhg3	-0.22 ± 0.11	0.834	-0.73 ± 0.12	$p < 0.001$	-1.28 ± 0.20	$p < 0.001$	NR_003270	small nucleolar RNA host gene 3
Snhg7	-0.20 ± 0.06	1.000	-0.54 ± 0.14	0.029	-1.05 ± 0.08	$p < 0.001$	ENSMUST00000181621	small nucleolar RNA host gene 7//small nucleolar RNA, H/ACA box 17
Snora17	-0.17 ± 0.15	0.885	-0.22 ± 0.12	0.313	-1.24 ± 0.16	$p < 0.001$	NR_028571	small nucleolar RNA, H/ACA box 17
Snora23	-0.37 ± 0.14	0.004	-0.71 ± 0.09	$p < 0.001$	-1.97 ± 0.14	$p < 0.001$	NR_033336	small nucleolar RNA, H/ACA box 23
Snora2b	-0.28 ± 0.35	1.000	-0.64 ± 0.35	0.128	-1.16 ± 0.07	$p < 0.001$	NR_034052	small nucleolar RNA, H/ACA box 2B
Snora33	-0.29 ± 0.12	0.994	-0.81 ± 0.12	0.003	-1.43 ± 0.52	$p < 0.001$	NR_037680	small nucleolar RNA, H/ACA box 33
Snora34	-1.28 ± 0.54	$p < 0.001$	-0.56 ± 0.28	$p < 0.001$	-0.76 ± 0.27	$p < 0.001$	NR_034051	small nucleolar RNA, H/ACA box 34
Snora68	-0.03 ± 0.22	1.000	-0.16 ± 0.22	0.663	-1.36 ± 0.19	$p < 0.001$	NR_002901	small nucleolar RNA, H/ACA box 68
Snora69	-0.46 ± 0.38	0.304	-0.71 ± 0.29	0.005	-1.45 ± 0.34	$p < 0.001$	NR_002900	small nucleolar RNA, H/ACA box 69
Snora78	-0.18 ± 0.10	1.000	-0.49 ± 0.12	0.005	-1.24 ± 0.11	$p < 0.001$	NR_028515	small nucleolar RNA, H/ACA box 7
Snord43	0.01 ± 0.31	1.000	-0.52 ± 0.22	0.055	-1.79 ± 0.21	$p < 0.001$	NR_028281	small nucleolar RNA, C/D box 43
Snord45b	0.18 ± 0.46	0.985	1.10 ± 0.32	$p < 0.001$	0.47 ± 0.33	0.004	NR_028561	small nucleolar RNA, C/D box 45B
Snord4a	-0.50 ± 0.12	0.524	-0.87 ± 0.11	0.006	-1.75 ± 0.26	$p < 0.001$	NR_030702	small nucleolar RNA, C/D box 4A
Snord52	-0.08 ± 0.22	1.000	-0.52 ± 0.22	0.223	-1.58 ± 0.25	$p < 0.001$	NR_028527	small nucleolar RNA, C/D box 52
Snord88a	-0.17 ± 0.39	1.000	-0.51 ± 0.43	0.163	-1.01 ± 0.39	$p < 0.001$	NR_028533	small nucleolar RNA, C/D box 88A
Snord93	-0.36 ± 0.09	0.014	-0.50 ± 0.08	$p < 0.001$	-1.40 ± 0.15	$p < 0.001$	NR_028536	small nucleolar RNA, C/D box 93
Snrpd1	0.10 ± 0.15	1.000	-0.15 ± 0.17	0.575	-1.24 ± 0.21	$p < 0.001$	NM_009226	small nuclear ribonucleoprotein D1
Snx20	0.14 ± 0.19	1.000	0.41 ± 0.08	0.004	1.27 ± 0.17	$p < 0.001$	NM_027840	sorting nexin 20
Snx32	-0.19 ± 0.21	1.000	0.04 ± 0.27	1.000	1.72 ± 0.19	$p < 0.001$	NM_001024560	sorting nexin 32
Snx33	0.12 ± 0.16	1.000	0.38 ± 0.17	0.351	1.24 ± 0.15	$p < 0.001$	NM_175483	sorting nexin 33
Soat2	0.49 ± 0.10	0.025	0.75 ± 0.10	$p < 0.001$	2.55 ± 0.09	$p < 0.001$	NM_146064	sterol O-acyltransferase 2
Sp100	0.50 ± 0.10	0.044	1.64 ± 0.05	$p < 0.001$	1.85 ± 0.08	$p < 0.001$	NM_013673	nuclear antigen Sp100
Spag5	0.26 ± 0.07	0.175	0.06 ± 0.14	1.000	-1.30 ± 0.23	$p < 0.001$	NM_017407	sperm associated antigen 5
Spata1	0.27 ± 0.10	1.000	0.22 ± 0.20	0.970	1.07 ± 0.16	$p < 0.001$	NM_027617	spermatogenesis associated 1
Spc25	0.06 ± 0.10	1.000	-0.41 ± 0.09	0.003	-1.25 ± 0.12	$p < 0.001$	AK146727	SPC25, NDC80 kinetochore complex component, homolog (S. cerevisiae)
Sphk2	0.11 ± 0.16	1.000	0.06 ± 0.11	1.000	1.38 ± 0.03	$p < 0.001$	NM_001172561	sphingosine kinase 2
Sppl2b	-0.05 ± 0.14	1.000	-0.29 ± 0.14	0.310	-1.03 ± 0.24	$p < 0.001$	AK157906	signal peptide peptidase like 2B
Spred3	0.18 ± 0.25	1.000	0.30 ± 0.28	0.309	1.39 ± 0.27	$p < 0.001$	NM_182927	sprouty-related, EVH1 domain containing 3
Spty2d1	0.40 ± 0.07	0.095	0.48 ± 0.16	0.005	1.18 ± 0.08	$p < 0.001$	NM_175318	SPT2, Suppressor of Ty, domain containing 1 (S. cerevisiae)
Sqle	-0.17 ± 0.03	0.569	-0.25 ± 0.02	0.072	-1.74 ± 0.11	$p < 0.001$	NM_009270	squalene epoxidase
Sqstm1	0.11 ± 0.09	0.917	0.42 ± 0.10	$p < 0.001$	1.40 ± 0.10	$p < 0.001$	NM_001290769	sequestosome 1
Srd5a1	-0.32 ± 0.42	0.934	-0.60 ± 0.25	0.068	-1.39 ± 0.25	$p < 0.001$	NM_175283	steroid 5 alpha-reductase 1
Srebf1	-0.24 ± 0.07	0.426	-0.33 ± 0.09	0.026	-1.59 ± 0.08	$p < 0.001$	NM_011480	sterol regulatory element binding transcription factor 1
Srm	-0.06 ± 0.03	1.000	-0.38 ± 0.09	$p < 0.001$	-1.89 ± 0.07	$p < 0.001$	NM_009272	spermidine synthase

Srrt	0.19 ± 0.03	0.523	0.14 ± 0.11	0.621	-1.26 ± 0.04	$p < 0.001$	NM_001109909	serrate RNA effector molecule homolog (Arabidopsis)
Srsf1	-0.12 ± 0.02	1.000	-0.30 ± 0.06	0.023	-1.28 ± 0.02	$p < 0.001$	NM_001078167	serine/arginine-rich splicing factor 1
Srsf2	-0.19 ± 0.04	0.201	-0.49 ± 0.06	$p < 0.001$	-1.28 ± 0.01	$p < 0.001$	NM_011358	serine/arginine-rich splicing factor 2
Ssu72	0.69 ± 0.13	0.116	1.00 ± 0.21	$p < 0.001$	1.37 ± 0.13	$p < 0.001$	ENSMUST00000178987	Ssu72 RNA polymerase II CTD phosphatase homolog (yeast)
Ssx2ip	-0.10 ± 0.19	1.000	-0.17 ± 0.18	0.689	-1.62 ± 0.21	$p < 0.001$	NM_001253768	synovial sarcoma, X breakpoint 2 interacting protein
St18	0.01 ± 0.14	1.000	0.39 ± 0.12	0.114	1.73 ± 0.17	$p < 0.001$	NM_001244692	suppression of tumorigenicity 18
St3gal1	0.57 ± 0.14	0.002	0.70 ± 0.13	$p < 0.001$	2.16 ± 0.13	$p < 0.001$	NM_009177	ST3 beta-galactoside alpha-2,3-sialyltransferase 1
St7	0.01 ± 0.05	1.000	0.30 ± 0.06	0.595	1.45 ± 0.05	$p < 0.001$	NM_001083315	suppression of tumorigenicity 7
Stard4	-0.09 ± 0.06	1.000	-0.10 ± 0.13	0.991	-1.50 ± 0.21	$p < 0.001$	XM_006525694	StAR-related lipid transfer (START) domain containing 4
Stard5	-0.04 ± 0.12	1.000	0.41 ± 0.16	0.122	1.28 ± 0.09	$p < 0.001$	NM_023377	StAR-related lipid transfer (START) domain containing 5
Stat1	0.75 ± 0.03	$p < 0.001$	1.85 ± 0.04	$p < 0.001$	1.55 ± 0.08	$p < 0.001$	NM_001205313	signal transducer and activator of transcription 1
Stat2	0.64 ± 0.08	$p < 0.001$	1.79 ± 0.09	$p < 0.001$	1.84 ± 0.09	$p < 0.001$	NM_019963	signal transducer and activator of transcription 2
Steap3	-0.19 ± 0.22	1.000	-0.56 ± 0.22	0.018	-1.21 ± 0.23	$p < 0.001$	NM_001085409	STEAP family member 3
Stil	-0.06 ± 0.07	1.000	-0.24 ± 0.08	0.159	-1.30 ± 0.19	$p < 0.001$	NM_009185	Scl/Tal1 interrupting locus
Stk17b	0.10 ± 0.07	1.000	0.26 ± 0.07	0.278	1.07 ± 0.11	$p < 0.001$	NM_133810	serine/threonine kinase 17b (apoptosis-inducing)
Stk40	0.49 ± 0.13	0.004	0.56 ± 0.13	$p < 0.001$	2.15 ± 0.13	$p < 0.001$	NM_001145827	serine/threonine kinase 40
Stmn1	-0.20 ± 0.12	1.000	-0.39 ± 0.14	0.145	-1.56 ± 0.13	$p < 0.001$	ENSMUST00000105868	stathmin 1
Stom	0.33 ± 0.01	0.006	0.59 ± 0.04	$p < 0.001$	1.21 ± 0.10	$p < 0.001$	NM_013515	stomatin
Stx11	0.50 ± 0.06	0.254	0.61 ± 0.08	0.030	1.66 ± 0.10	$p < 0.001$	NM_001163590	syntaxin 11
Stxbp3a	0.19 ± 0.06	0.724	0.40 ± 0.05	0.003	1.34 ± 0.15	$p < 0.001$	NM_011504	syntaxin binding protein 3A
Sumo2	0.10 ± 0.10	1.000	-0.18 ± 0.18	0.769	-1.49 ± 0.17	$p < 0.001$	NM_133354	SMT3 suppressor of mif two 3 homolog 2 (yeast)
Suv39h1	-0.09 ± 0.17	1.000	-0.39 ± 0.18	0.009	-1.51 ± 0.14	$p < 0.001$	NM_001290716	suppressor of variegation 3-9 homolog 1 (Drosophila)
Suv39h2	-0.21 ± 0.14	1.000	-0.38 ± 0.13	0.295	-1.42 ± 0.13	$p < 0.001$	NM_022724	suppressor of variegation 3-9 homolog 2 (Drosophila)
Syce2	0.04 ± 0.06	1.000	0.07 ± 0.09	1.000	-1.32 ± 0.07	$p < 0.001$	NM_001168244	synaptonemal complex central element protein 2
Syk	0.09 ± 0.14	1.000	0.11 ± 0.09	0.989	1.15 ± 0.09	$p < 0.001$	NM_001198977	spleen tyrosine kinase
Syne1	0.11 ± 0.11	1.000	0.45 ± 0.11	0.017	1.08 ± 0.20	$p < 0.001$	NM_001079686	spectrin repeat containing, nuclear envelope 1
Tacc3	0.06 ± 0.01	1.000	-0.32 ± 0.03	0.018	-2.08 ± 0.09	$p < 0.001$	NM_001040435	transforming, acidic coiled-coil containing protein 3
Tada1	-0.16 ± 0.07	0.843	-0.29 ± 0.16	0.042	-1.10 ± 0.10	$p < 0.001$	NM_030245	transcriptional adaptor 1
Taf1b	0.10 ± 0.04	1.000	0.05 ± 0.05	1.000	1.14 ± 0.17	$p < 0.001$	NM_020614	TATA box binding protein (Tbp)-associated factor, RNA polymerase I, B
Tagln2	-0.07 ± 0.01	1.000	-0.23 ± 0.02	0.156	-1.90 ± 0.09	$p < 0.001$	NM_178598	transgelin 2
Tamm41	-0.09 ± 0.15	1.000	-0.20 ± 0.09	0.726	-1.38 ± 0.12	$p < 0.001$	NM_026894	TAM41, mitochondrial translocator assembly and maintenance protein, homolog (S. cerevisiae)
Tanc2	0.29 ± 0.14	0.989	0.47 ± 0.15	0.171	1.04 ± 0.06	$p < 0.001$	NM_181071	tetratricopeptide repeat, ankyrin repeat and coiled-coil containing 2
Tank	0.27 ± 0.08	0.653	0.50 ± 0.06	0.007	1.51 ± 0.09	$p < 0.001$	NM_001164071	TRAF family member-associated Nf-kappa B activator
Taok2	-0.22 ± 0.08	0.523	-0.48 ± 0.13	$p < 0.001$	-1.22 ± 0.04	$p < 0.001$	NM_001163774	TAO kinase 2
Tap1	0.37 ± 0.08	0.016	1.01 ± 0.08	$p < 0.001$	1.62 ± 0.07	$p < 0.001$	NM_001161730	transporter 1, ATP-binding cassette, sub-family B (MDR/TAP)
Tapbp	0.19 ± 0.01	0.550	0.76 ± 0.01	$p < 0.001$	1.00 ± 0.10	$p < 0.001$	NM_001025313	TAP binding protein
Tardbp	-0.07 ± 0.02	1.000	-0.24 ± 0.03	0.149	-1.29 ± 0.03	$p < 0.001$	NM_001003898	TAR DNA binding protein
Tarm1	-0.25 ± 0.23	1.000	0.30 ± 0.06	0.718	1.87 ± 0.28	$p < 0.001$	NM_177363	T cell-interacting, activating receptor on myeloid cells 1
Tarsl2	-0.08 ± 0.07	1.000	-0.06 ± 0.06	1.000	-1.05 ± 0.39	$p < 0.001$	NM_172310	threonyl-tRNA synthetase-like 2
Tatdn1	0.14 ± 0.12	1.000	0.25 ± 0.27	0.469	1.60 ± 0.11	$p < 0.001$	NM_175151	TatD DNase domain containing 1
Tbc1d9	0.11 ± 0.33	1.000	0.13 ± 0.06	1.000	1.69 ± 0.08	$p < 0.001$	NM_001111304	TBC1 domain family, member 9

Tbcb	-0.12 ± 0.07	1.000	-0.28 ± 0.07	0.057	-1.17 ± 0.08	p < 0.001	NM_025548	tubulin folding cofactor B
Tbl2	-0.07 ± 0.08	1.000	-0.06 ± 0.08	1.000	-1.16 ± 0.11	p < 0.001	NM_013763	transducin (beta)-like 2
Tbl3	0.06 ± 0.05	1.000	-0.28 ± 0.04	0.087	-1.03 ± 0.15	p < 0.001	NM_145396	transducin (beta)-like 3
Tcf19	-0.36 ± 0.03	0.010	-0.55 ± 0.03	p < 0.001	-1.55 ± 0.05	p < 0.001	NM_001163763	transcription factor 19
Tcf3	-0.03 ± 0.22	1.000	0.12 ± 0.20	0.846	-1.27 ± 0.21	p < 0.001	NM_001164147	transcription factor 3
Tcta	-0.40 ± 0.26	0.692	-0.06 ± 0.20	1.000	1.29 ± 0.05	p < 0.001	NM_133986	T cell leukemia translocation altered gene
Tdrd7	0.61 ± 0.11	0.030	1.50 ± 0.20	p < 0.001	1.42 ± 0.14	p < 0.001	NM_001290475	tudor domain containing 7
Tecpr1	0.13 ± 0.19	1.000	0.28 ± 0.19	0.351	1.26 ± 0.23	p < 0.001	NM_027410	tectonin beta-propeller repeat containing 1
Tenm4	0.16 ± 0.09	1.000	0.27 ± 0.18	0.880	1.90 ± 0.09	p < 0.001	ENSMUST00000107162	teneurin transmembrane protein 4
Tet2	0.20 ± 0.11	0.661	0.68 ± 0.14	p < 0.001	1.19 ± 0.11	p < 0.001	NM_001040400	tet methylcytosine dioxygenase 2
Tfap4	-0.45 ± 0.19	0.032	-0.41 ± 0.20	0.024	-1.73 ± 0.20	p < 0.001	NM_031182	transcription factor AP4
Tfcp2l1	0.07 ± 0.12	1.000	0.16 ± 0.14	0.887	1.16 ± 0.07	p < 0.001	NM_023755	transcription factor CP2-like 1
Tfdp1	-0.02 ± 0.06	1.000	-0.23 ± 0.06	0.119	-1.71 ± 0.16	p < 0.001	NM_001291765	transcription factor Dp 1
Tfdp2	-0.34 ± 0.22	0.594	-0.40 ± 0.14	0.191	-1.48 ± 0.33	p < 0.001	NM_001184706	transcription factor Dp 2
Thada	0.06 ± 0.09	1.000	-0.15 ± 0.07	0.728	-1.04 ± 0.08	p < 0.001	NM_183021	thyroid adenoma associated
Thap6	0.20 ± 0.23	1.000	0.21 ± 0.15	0.844	1.78 ± 0.13	p < 0.001	NR_028429	THAP domain containing 6
Them4	0.38 ± 0.21	0.807	0.49 ± 0.19	0.208	2.45 ± 0.23	p < 0.001	NM_029431	thioesterase superfamily member 4
Them6	0.03 ± 0.03	1.000	-0.23 ± 0.02	0.194	-1.37 ± 0.22	p < 0.001	NM_198607	thioesterase superfamily member 6
Themis2	0.03 ± 0.05	1.000	0.08 ± 0.05	0.991	-1.51 ± 0.05	p < 0.001	NM_001033308	thymocyte selection associated family member 2
Thop1	0.04 ± 0.09	1.000	-0.04 ± 0.07	1.000	-1.80 ± 0.03	p < 0.001	NM_022653	thimet oligopeptidase 1
Ticam2	0.23 ± 0.15	1.000	1.11 ± 0.15	p < 0.001	1.39 ± 0.16	p < 0.001	NM_173394	toll-like receptor adaptor molecule 2
Ticrr	-0.05 ± 0.06	1.000	-0.29 ± 0.06	0.107	-1.50 ± 0.12	p < 0.001	NM_029835	TOPBP1-interacting checkpoint and replication regulator
Tigd2	0.17 ± 0.03	1.000	0.32 ± 0.04	0.314	1.60 ± 0.20	p < 0.001	NM_001081145	tigger transposable element derived 2
Timeless	-0.20 ± 0.05	0.756	-0.03 ± 0.08	1.000	-1.28 ± 0.08	p < 0.001	NM_001136082	timeless circadian clock 1
Timm50	0.04 ± 0.09	1.000	-0.13 ± 0.13	0.749	-1.07 ± 0.25	p < 0.001	NM_025616	translocase of inner mitochondrial membrane 50
Timm8a1	0.10 ± 0.11	1.000	-0.08 ± 0.12	0.989	-1.06 ± 0.09	p < 0.001	ENSMUST00000052902	translocase of inner mitochondrial membrane 8A1
Tipin	0.02 ± 0.09	1.000	-0.09 ± 0.05	0.953	-1.36 ± 0.01	p < 0.001	NM_025372	timeless interacting protein
Tk1	-0.21 ± 0.05	0.376	-0.37 ± 0.01	0.002	-1.92 ± 0.11	p < 0.001	NM_001271729	thymidine kinase 1
Tlcd1	-0.34 ± 0.44	0.682	-0.37 ± 0.41	0.325	-1.32 ± 0.48	p < 0.001	NM_001291235	TLC domain containing 1
Tlr1	-0.02 ± 0.17	1.000	0.23 ± 0.16	0.920	1.87 ± 0.20	p < 0.001	NM_030682	toll-like receptor 1
Tlr13	-0.40 ± 0.06	0.002	-0.38 ± 0.21	0.002	-1.02 ± 0.15	p < 0.001	NM_205820	toll-like receptor 13
Tlr3	0.33 ± 0.08	0.113	1.22 ± 0.12	p < 0.001	0.90 ± 0.08	p < 0.001	NM_126166	toll-like receptor 3
Tlr6	0.10 ± 0.07	1.000	0.39 ± 0.23	0.215	1.11 ± 0.02	p < 0.001	NM_011604	toll-like receptor 6
Tm6sf1	0.00 ± 0.07	1.000	0.00 ± 0.09	1.000	-1.10 ± 0.07	p < 0.001	NM_001291282	transmembrane 6 superfamily member 1
Tm9sf4	0.13 ± 0.03	0.939	0.51 ± 0.01	p < 0.001	1.16 ± 0.03	p < 0.001	NM_133847	transmembrane 9 superfamily protein member 4
Tmem126a	0.27 ± 0.13	0.152	0.49 ± 0.17	p < 0.001	1.08 ± 0.13	p < 0.001	NM_025460	transmembrane protein 126A
Tmem140	0.39 ± 0.21	0.941	0.82 ± 0.73	0.015	2.54 ± 0.21	p < 0.001	NM_197986	transmembrane protein 140
Tmem156	0.22 ± 0.06	1.000	0.22 ± 0.02	0.753	1.02 ± 0.03	p < 0.001	XM_006543959	transmembrane protein 156
Tmem158	-0.28 ± 0.15	0.918	-0.64 ± 0.11	0.013	-1.14 ± 0.05	p < 0.001	NM_001002267	transmembrane protein 158
Tmem168	-0.01 ± 0.19	1.000	0.16 ± 0.12	0.827	1.27 ± 0.14	p < 0.001	XM_006504952	transmembrane protein 168
Tmem171	0.55 ± 0.02	p < 0.001	0.60 ± 0.16	p < 0.001	1.15 ± 0.14	p < 0.001	NM_001025606	transmembrane protein 171

Tmem176b	0.25 ± 0.09	0.229	0.44 ± 0.10	p < 0.001	1.06 ± 0.09	p < 0.001	NM_001164207	transmembrane protein 176B
Tmem201	0.04 ± 0.08	1.000	-0.18 ± 0.05	0.602	-1.36 ± 0.06	p < 0.001	NM_001284270	transmembrane protein 201
Tmem238	-0.17 ± 0.18	1.000	-0.62 ± 0.09	0.002	-1.14 ± 0.07	p < 0.001	ENSMUST00000168578	transmembrane protein 238
Tmem254b	-0.31 ± 0.06	0.314	-0.75 ± 0.05	p < 0.001	-1.76 ± 0.02	p < 0.001	NM_001270495	transmembrane protein 254b
Tmem38b	0.15 ± 0.14	1.000	0.65 ± 0.11	0.002	1.51 ± 0.11	p < 0.001	NM_028053	transmembrane protein 38B
Tmem41b	-0.25 ± 0.06	0.177	-0.39 ± 0.12	0.002	-1.02 ± 0.14	p < 0.001	NM_153525	transmembrane protein 41B
Tmem50b	-0.24 ± 0.06	0.300	-0.60 ± 0.06	p < 0.001	-1.14 ± 0.07	p < 0.001	NM_030018	transmembrane protein 50B
Tmem51	-0.47 ± 0.15	0.244	-0.44 ± 0.13	0.140	-1.29 ± 0.12	p < 0.001	NM_145402	transmembrane protein 51
Tmem64	-0.13 ± 0.13	1.000	-0.20 ± 0.07	0.333	-1.28 ± 0.10	p < 0.001	NM_181401	transmembrane protein 64
Tmem68	0.24 ± 0.10	0.482	0.43 ± 0.07	0.002	1.21 ± 0.09	p < 0.001	NM_028097	transmembrane protein 68
Tmem71	-0.09 ± 0.39	1.000	0.06 ± 0.12	1.000	1.38 ± 0.07	p < 0.001	NM_172514	transmembrane protein 71
Tmem74	0.40 ± 0.04	0.659	0.39 ± 0.19	0.415	1.30 ± 0.27	p < 0.001	NM_175502	transmembrane protein 74
Tmpo	-0.07 ± 0.03	1.000	-0.26 ± 0.04	0.076	-1.28 ± 0.11	p < 0.001	NM_001080129	thymopoietin
Tmtc4	0.00 ± 0.16	1.000	-0.18 ± 0.16	0.919	-1.19 ± 0.25	p < 0.001	NM_028651	transmembrane and tetratricopeptide repeat containing 4
Tnf	1.73 ± 0.07	p < 0.001	2.04 ± 0.05	p < 0.001	2.57 ± 0.06	p < 0.001	NM_001278601	tumor necrosis factor
Tnfaip2	0.50 ± 0.05	p < 0.001	0.72 ± 0.07	p < 0.001	1.13 ± 0.05	p < 0.001	NM_009396	tumor necrosis factor, alpha-induced protein 2
Tnfaip3	0.88 ± 0.21	p < 0.001	1.20 ± 0.17	p < 0.001	3.15 ± 0.18	p < 0.001	NM_001166402	tumor necrosis factor, alpha-induced protein 3
Tnfaip8l1	0.02 ± 0.08	1.000	-0.22 ± 0.10	0.871	-1.05 ± 0.07	p < 0.001	NM_025566	tumor necrosis factor, alpha-induced protein 8-like 1
Tnfrsf11a	-0.23 ± 0.06	0.399	-0.16 ± 0.10	0.611	-1.04 ± 0.07	p < 0.001	NM_009399	tumor necrosis factor receptor superfamily, member 11a, NFKB activator
Tnfrsf1b	0.28 ± 0.23	0.890	1.33 ± 0.12	p < 0.001	3.92 ± 0.13	p < 0.001	NM_011610	tumor necrosis factor receptor superfamily, member 1b
Tnfrsf22	-0.24 ± 0.12	0.500	-0.33 ± 0.12	0.055	-1.39 ± 0.21	p < 0.001	ENSMUST00000075588	tumor necrosis factor receptor superfamily, member 22
Tnfrsf26	0.02 ± 0.02	1.000	0.39 ± 0.06	0.002	1.20 ± 0.05	p < 0.001	NM_175649	tumor necrosis factor receptor superfamily, member 26
Tnfrsf9	0.31 ± 0.15	0.877	1.35 ± 0.20	p < 0.001	3.15 ± 0.24	p < 0.001	NM_001077509	tumor necrosis factor receptor superfamily, member 9
Tnip1	0.91 ± 0.15	p < 0.001	1.13 ± 0.13	p < 0.001	2.00 ± 0.14	p < 0.001	NM_001199275	TNFAIP3 interacting protein 1
Tomm40	-0.02 ± 0.08	1.000	-0.22 ± 0.12	0.192	-1.11 ± 0.08	p < 0.001	NM_001109748	translocase of outer mitochondrial membrane 40 homolog (yeast)
Tomm5	-0.20 ± 0.12	0.389	-0.30 ± 0.12	0.017	-1.28 ± 0.30	p < 0.001	NM_001099675	translocase of outer mitochondrial membrane 5 homolog (yeast)
Tonsl	-0.29 ± 0.09	0.682	-0.61 ± 0.16	0.005	-1.35 ± 0.16	p < 0.001	NM_183091	tonsoku-like, DNA repair protein
Top2a	-0.07 ± 0.03	1.000	-0.18 ± 0.09	0.336	-1.72 ± 0.21	p < 0.001	NM_011623	topoisomerase (DNA) II alpha
Topbp1	-0.07 ± 0.02	1.000	-0.28 ± 0.05	0.040	-2.02 ± 0.05	p < 0.001	NM_176979	topoisomerase (DNA) II binding protein 1
Tor3a	0.66 ± 0.02	p < 0.001	1.20 ± 0.09	p < 0.001	1.38 ± 0.08	p < 0.001	NM_023141	torsin family 3, member A
Tpcn1	-0.26 ± 0.13	0.098	-0.58 ± 0.11	p < 0.001	-2.17 ± 0.10	p < 0.001	NM_145853	two pore channel 1
Tpgs2	-0.10 ± 0.02	1.000	-0.39 ± 0.02	0.002	-1.18 ± 0.18	p < 0.001	NM_001004361	tubulin polyglutamylase complex subunit 2
Tprkb	0.03 ± 0.12	1.000	-0.04 ± 0.10	1.000	1.11 ± 0.16	p < 0.001	NM_001170488	Tp53rk binding protein
Tprn	0.03 ± 0.17	1.000	-0.48 ± 0.15	0.172	-1.03 ± 0.18	p < 0.001	NM_175286	taperin
Tpx2	0.12 ± 0.02	1.000	-0.21 ± 0.03	0.216	-1.67 ± 0.06	p < 0.001	NM_001141975	TPX2, microtubule-associated protein homolog (Xenopus laevis)
Traf1	0.75 ± 0.25	0.003	0.86 ± 0.25	p < 0.001	3.43 ± 0.42	p < 0.001	NM_009421	TNF receptor-associated factor 1
Traf7	0.01 ± 0.03	1.000	-0.10 ± 0.03	0.906	-1.26 ± 0.03	p < 0.001	NM_001172113	TNF receptor-associated factor 7
Trafd1	0.62 ± 0.20	0.002	1.68 ± 0.04	p < 0.001	1.09 ± 0.02	p < 0.001	XM_006530302	TRAF type zinc finger domain containing 1
Traip	0.04 ± 0.13	1.000	-0.27 ± 0.17	0.426	-1.13 ± 0.30	p < 0.001	NM_011634	TRAF-interacting protein
Traj13	-0.32 ± 0.32	1.000	-0.12 ± 0.51	1.000	1.05 ± 0.57	0.004	ENSMUST00000103728	T cell receptor alpha joining 13
Traj46	0.53 ± 0.21	0.616	0.16 ± 0.27	1.000	1.13 ± 0.44	p < 0.001	ENSMUST00000103696	T cell receptor alpha joining 46

Trav14-1	-0.37 ± 0.05	1.000	-0.16 ± 0.06	1.000	1.18 ± 0.96	0.001	ENSMUST00000103652	T cell receptor alpha variable 14-1
Trem2	-0.14 ± 0.05	0.608	-0.46 ± 0.07	p < 0.001	-1.24 ± 0.04	p < 0.001	NM_001272078	triggering receptor expressed on myeloid cells 2
Trex1	0.63 ± 0.11	0.097	1.15 ± 0.05	p < 0.001	1.48 ± 0.05	p < 0.001	NM_001012236	three prime repair exonuclease 1
Trib3	0.49 ± 0.17	0.004	0.86 ± 0.20	p < 0.001	1.84 ± 0.13	p < 0.001	NM_175093	tribbles homolog 3 (Drosophila)
Trim21	0.66 ± 0.08	0.001	1.26 ± 0.05	p < 0.001	1.19 ± 0.07	p < 0.001	NM_001082552	tripartite motif-containing 21
Trim28	-0.10 ± 0.05	1.000	-0.36 ± 0.06	0.003	-1.91 ± 0.07	p < 0.001	NM_011588	tripartite motif-containing 28
Trim30a	1.45 ± 0.74	p < 0.001	2.21 ± 0.72	p < 0.001	2.01 ± 0.72	p < 0.001	AK089726	tripartite motif-containing 30A
Trim30c	0.69 ± 0.33	0.268	0.54 ± 0.53	0.359	1.63 ± 0.57	p < 0.001	XR_378392	tripartite motif-containing 30C
Trim30d	-0.07 ± 0.38	1.000	0.28 ± 0.72	0.948	1.63 ± 0.36	p < 0.001	NM_001167828	tripartite motif-containing 30D
Trim34a	0.72 ± 0.20	0.072	1.74 ± 0.15	p < 0.001	2.14 ± 0.21	p < 0.001	ENSMUST00000106854	tripartite motif-containing 34A
Trim37	-0.18 ± 0.15	0.579	-0.25 ± 0.13	0.111	-1.19 ± 0.13	p < 0.001	NM_197987	tripartite motif-containing 37
Trim46	0.26 ± 0.32	1.000	0.36 ± 0.09	0.416	1.03 ± 0.22	p < 0.001	NM_183037	tripartite motif-containing 46
Trim59	-0.18 ± 0.07	0.738	-0.36 ± 0.13	0.006	-1.54 ± 0.08	p < 0.001	NM_025863	tripartite motif-containing 59
Trim65	-0.21 ± 0.26	1.000	-0.52 ± 0.29	0.016	-1.69 ± 0.32	p < 0.001	NM_178802	tripartite motif-containing 65
Trip13	0.01 ± 0.15	1.000	-0.43 ± 0.04	0.002	-1.28 ± 0.09	p < 0.001	NM_027182	thyroid hormone receptor interactor 13
Trmt2a	-0.13 ± 0.03	1.000	-0.36 ± 0.01	0.007	-1.14 ± 0.10	p < 0.001	NM_001080999	TRM2 tRNA methyltransferase 2A
Trmt2b	-0.25 ± 0.04	0.499	-0.34 ± 0.04	0.060	-1.07 ± 0.14	p < 0.001	NM_001167994	TRM2 tRNA methyltransferase 2B
Trmt61a	-0.10 ± 0.11	1.000	-0.53 ± 0.09	0.001	-1.10 ± 0.17	p < 0.001	NM_177374	tRNA methyltransferase 61A
Troap	0.04 ± 0.15	1.000	-0.48 ± 0.08	p < 0.001	-2.12 ± 0.09	p < 0.001	NM_001162506	trophinin associated protein
Trp53cor1	0.11 ± 0.13	1.000	0.84 ± 0.13	p < 0.001	2.07 ± 0.13	p < 0.001	NR_036469	tumor protein p53 pathway corepressor 1
Trp53inp1	-0.02 ± 0.30	1.000	0.12 ± 0.21	1.000	1.63 ± 0.42	p < 0.001	NM_001199105	transformation related protein 53 inducible nuclear protein 1
Trp53inp2	0.12 ± 0.02	1.000	-0.19 ± 0.07	0.954	1.20 ± 0.02	p < 0.001	ENSMUST00000043237	transformation related protein 53 inducible nuclear protein 2
Trps1	0.26 ± 0.14	0.995	0.41 ± 0.14	0.185	1.06 ± 0.18	p < 0.001	ENSMUST00000183421	trichorhinophalangeal syndrome I (human)
Tspan13	-0.01 ± 0.18	1.000	-0.01 ± 0.05	1.000	1.05 ± 0.04	p < 0.001	ENSMUST00000128644	tetraspanin 13
Ttf2	0.06 ± 0.08	1.000	-0.25 ± 0.15	0.304	-1.14 ± 0.19	p < 0.001	NM_001013026	transcription termination factor, RNA polymerase II
Ttk	-0.03 ± 0.04	1.000	-0.44 ± 0.04	p < 0.001	-2.26 ± 0.23	p < 0.001	NM_001110265	Ttk protein kinase
Ttyh3	-0.27 ± 0.13	0.122	-0.49 ± 0.08	p < 0.001	-1.60 ± 0.09	p < 0.001	NM_175274	tweety homolog 3 (Drosophila)
Tuba1a	0.02 ± 0.22	1.000	-0.15 ± 0.20	0.996	-1.13 ± 0.21	p < 0.001	NM_011653	tubulin, alpha 1A
Tuba1c	0.02 ± 0.08	1.000	-0.33 ± 0.09	0.015	-1.05 ± 0.20	p < 0.001	NM_009448	tubulin, alpha 1C
Tuba4a	-0.21 ± 0.02	0.169	-0.66 ± 0.02	p < 0.001	-1.72 ± 0.02	p < 0.001	NM_009447	tubulin, alpha 4A
Tubb4b	-0.01 ± 0.20	1.000	-0.41 ± 0.19	0.457	-1.18 ± 0.20	p < 0.001	NM_146116	tubulin, beta 4B class IVB
Tubb5	-0.09 ± 0.06	0.915	-0.42 ± 0.07	p < 0.001	-2.72 ± 0.08	p < 0.001	ENSMUST00000134978	tubulin, beta 5 class I
Tubb6	-0.11 ± 0.04	1.000	-0.58 ± 0.07	p < 0.001	-2.05 ± 0.05	p < 0.001	NM_026473	tubulin, beta 6 class V
Tubgcp2	-0.08 ± 0.07	1.000	-0.26 ± 0.06	0.126	-1.08 ± 0.07	p < 0.001	NM_001286007	tubulin, gamma complex associated protein 2
Tufm	-0.10 ± 0.13	1.000	-0.23 ± 0.11	0.155	-1.04 ± 0.11	p < 0.001	NM_001163713	Tu translation elongation factor, mitochondrial
Twf2	-0.22 ± 0.10	0.264	-0.31 ± 0.12	0.013	-1.17 ± 0.10	p < 0.001	NM_011876	twinfilin, actin-binding protein, homolog 2 (Drosophila)
Txlnb	0.00 ± 0.07	1.000	0.00 ± 0.28	1.000	1.78 ± 0.40	p < 0.001	NM_138628	taxilin beta
Txnip	0.04 ± 0.01	1.000	0.23 ± 0.02	0.020	1.09 ± 0.07	p < 0.001	NM_001009935	thioredoxin interacting protein
Tyms	-0.21 ± 0.12	0.917	-0.24 ± 0.03	0.462	-1.13 ± 0.29	p < 0.001	NM_021288	thymidylate synthase
Uba2	-0.02 ± 0.10	1.000	-0.08 ± 0.06	0.989	-1.13 ± 0.05	p < 0.001	NM_016682	ubiquitin-like modifier activating enzyme 2
Uba7	0.47 ± 0.07	0.046	1.62 ± 0.10	p < 0.001	1.70 ± 0.08	p < 0.001	NM_023738	ubiquitin-like modifier activating enzyme 7

Ube2c	0.20 ± 0.04	0.442	-0.18 ± 0.08	0.399	-1.65 ± 0.05	$p < 0.001$	NM_026785	ubiquitin-conjugating enzyme E2C
Ube2cbp	-0.06 ± 0.33	1.000	-0.75 ± 0.23	0.021	-1.21 ± 0.22	$p < 0.001$	NM_027394	ubiquitin-conjugating enzyme E2C binding protein
Ube2g2	-0.05 ± 0.03	1.000	-0.18 ± 0.10	0.538	1.26 ± 0.15	$p < 0.001$	NM_019803	ubiquitin-conjugating enzyme E2G 2
Ube2o	0.04 ± 0.05	1.000	0.00 ± 0.05	1.000	-1.07 ± 0.05	$p < 0.001$	NM_173755	ubiquitin-conjugating enzyme E2O
Ube2t	0.03 ± 0.05	1.000	-0.05 ± 0.05	1.000	-1.34 ± 0.12	$p < 0.001$	NM_001278115	ubiquitin-conjugating enzyme E2T (putative)
Ubqln2	-0.14 ± 0.08	1.000	-0.22 ± 0.05	0.376	-1.08 ± 0.27	$p < 0.001$	NM_018798	ubiquilin 2
Ubqln4	0.08 ± 0.09	1.000	-0.25 ± 0.11	0.232	-1.43 ± 0.15	$p < 0.001$	NM_033526	ubiquilin 4
Ubr4	0.13 ± 0.07	1.000	0.72 ± 0.03	$p < 0.001$	1.12 ± 0.07	$p < 0.001$	NM_001160319	ubiquitin protein ligase E3 component n-recognin 4
Ubtf	-0.01 ± 0.03	1.000	-0.21 ± 0.08	0.228	-1.45 ± 0.03	$p < 0.001$	NM_001044383	upstream binding transcription factor, RNA polymerase I
Uchl5	0.09 ± 0.02	1.000	-0.11 ± 0.01	0.832	-1.49 ± 0.14	$p < 0.001$	NM_001159866	ubiquitin carboxyl-terminal esterase L5
Uck2	-0.06 ± 0.04	1.000	-0.24 ± 0.08	0.255	-1.21 ± 0.03	$p < 0.001$	XM_006497039	uridine-cytidine kinase 2
Uevld	0.27 ± 0.08	0.659	0.19 ± 0.05	0.738	-1.01 ± 0.11	$p < 0.001$	NM_001040695	UEV and lactate/malate dehyrogenase domains
Uhrf1	-0.25 ± 0.07	0.166	-0.59 ± 0.04	$p < 0.001$	-2.13 ± 0.14	$p < 0.001$	NM_001111078	ubiquitin-like, containing PHD and RING finger domains, 1
Umps	-0.02 ± 0.01	1.000	-0.46 ± 0.05	$p < 0.001$	-1.17 ± 0.04	$p < 0.001$	NM_009471	uridine monophosphate synthetase
Ung	-0.08 ± 0.06	1.000	-0.22 ± 0.16	0.657	-1.10 ± 0.08	$p < 0.001$	NM_001040691	uracil DNA glycosylase
Uqcrh	-0.20 ± 0.03	0.576	-0.47 ± 0.06	$p < 0.001$	-1.07 ± 0.21	$p < 0.001$	NM_025641	ubiquinol-cytochrome c reductase hinge protein
Usp10	0.06 ± 0.02	1.000	-0.15 ± 0.01	0.613	-1.10 ± 0.02	$p < 0.001$	NM_009462	ubiquitin specific peptidase 10
Usp11	0.37 ± 0.01	0.699	0.34 ± 0.30	0.514	1.73 ± 0.03	$p < 0.001$	NM_145628	ubiquitin specific peptidase 11
Usp18	1.75 ± 0.13	$p < 0.001$	2.99 ± 0.14	$p < 0.001$	3.07 ± 0.17	$p < 0.001$	NM_011909	ubiquitin specific peptidase 18
Vaultrc5	0.12 ± 0.51	1.000	0.28 ± 0.39	0.848	1.05 ± 0.36	$p < 0.001$	NR_027885	vault RNA component 5
Vcl	-0.26 ± 0.05	0.129	-0.50 ± 0.03	$p < 0.001$	-1.44 ± 0.14	$p < 0.001$	NM_009502	vinculin
Vcp	-0.10 ± 0.02	1.000	-0.20 ± 0.05	0.168	-1.10 ± 0.05	$p < 0.001$	NM_009503	valosin containing protein
Vegfc	0.63 ± 0.09	0.035	0.89 ± 0.07	$p < 0.001$	2.74 ± 0.07	$p < 0.001$	NM_009506	vascular endothelial growth factor C
Vmn2r109	0.51 ± 0.29	0.650	0.40 ± 0.28	0.626	1.04 ± 0.47	0.001	NM_001104571	vomeronasal 2, receptor 109
Vmn2r113	-0.43 ± 0.86	0.936	-1.00 ± 0.81	0.012	-1.05 ± 0.89	0.005	ENSMUST00000170322	vomeronasal 2, receptor 113
Vps37c	0.10 ± 0.19	1.000	0.12 ± 0.05	0.966	1.23 ± 0.15	$p < 0.001$	NM_181403	vacuolar protein sorting 37C (yeast)
Vwa5a	-0.07 ± 0.05	1.000	0.09 ± 0.02	0.952	1.06 ± 0.11	$p < 0.001$	NM_001145957	von Willebrand factor A domain containing 5A
Wdfy3	0.16 ± 0.05	0.948	0.64 ± 0.02	$p < 0.001$	1.12 ± 0.02	$p < 0.001$	NM_172882	WD repeat and FYVE domain containing 3
Wdhd1	-0.34 ± 0.13	0.022	-0.69 ± 0.05	$p < 0.001$	-2.02 ± 0.25	$p < 0.001$	NM_172598	WD repeat and HMG-box DNA binding protein 1
Wdr6	-0.21 ± 0.01	1.000	-0.46 ± 0.04	0.052	-1.72 ± 0.39	$p < 0.001$	NM_031392	WD repeat domain 6
Wdr62	-0.15 ± 0.18	1.000	-0.21 ± 0.19	0.498	-1.06 ± 0.21	$p < 0.001$	NM_146186	WD repeat domain 62
Wdr66	-0.09 ± 0.27	1.000	0.30 ± 0.31	0.473	2.25 ± 0.28	$p < 0.001$	XM_006530515	WD repeat domain 66
Wdr76	-0.20 ± 0.06	0.650	-0.40 ± 0.13	0.005	-1.32 ± 0.30	$p < 0.001$	NM_001290986	WD repeat domain 76
Wdr77	-0.02 ± 0.05	1.000	-0.29 ± 0.05	0.031	-1.07 ± 0.05	$p < 0.001$	NM_027432	WD repeat domain 77
Wdr90	0.09 ± 0.09	1.000	-0.09 ± 0.05	1.000	-1.38 ± 0.03	$p < 0.001$	NM_001163766	WD repeat domain 90
Wee1	-0.27 ± 0.05	0.200	-0.40 ± 0.07	0.002	-2.77 ± 0.02	$p < 0.001$	NM_009516	WEE 1 homolog 1 (S. pombe)
Wrb	-0.16 ± 0.13	1.000	-0.50 ± 0.06	0.010	-1.48 ± 0.14	$p < 0.001$	NM_207301	tryptophan rich basic protein
Xaf1	1.49 ± 0.34	$p < 0.001$	2.50 ± 0.33	$p < 0.001$	2.84 ± 0.34	$p < 0.001$	NM_001037713	XIAP associated factor 1
Xdh	-0.04 ± 0.06	1.000	0.39 ± 0.06	0.029	1.69 ± 0.13	$p < 0.001$	NM_011723	xanthine dehydrogenase
Xpnpep1	-0.07 ± 0.05	1.000	0.05 ± 0.04	1.000	-1.04 ± 0.03	$p < 0.001$	NM_133216	X-prolyl aminopeptidase (aminopeptidase P) 1, soluble
Xrcc3	-0.18 ± 0.20	0.837	-0.54 ± 0.04	$p < 0.001$	-1.27 ± 0.06	$p < 0.001$	NM_028875	X-ray repair complementing defective repair in Chinese hamster cells 3

Xrcc6bp1	-0.15 ± 0.22	1.000	-0.26 ± 0.21	0.608	-1.20 ± 0.33	p < 0.001	NM_001159559	XRCC6 binding protein 1
Yae1d1	-0.39 ± 0.22	0.359	-0.53 ± 0.48	0.025	-1.15 ± 0.22	p < 0.001	NM_025904	Yae1 domain containing 1
Ypel5	0.20 ± 0.10	1.000	0.50 ± 0.24	0.023	1.85 ± 0.14	p < 0.001	NM_027166	yippee-like 5 (Drosophila)
Zbp1	0.57 ± 0.29	0.181	2.55 ± 0.34	p < 0.001	3.46 ± 0.28	p < 0.001	NM_021394	Z-DNA binding protein 1
Zbtb1	-0.19 ± 0.18	1.000	-0.30 ± 0.16	0.347	-1.07 ± 0.15	p < 0.001	NM_178744	zinc finger and BTB domain containing 1
Zbtb32	-0.17 ± 0.02	0.856	-0.46 ± 0.02	p < 0.001	-1.20 ± 0.08	p < 0.001	NM_021397	zinc finger and BTB domain containing 32
Zbtb38	-0.23 ± 0.22	1.000	0.10 ± 0.05	1.000	1.31 ± 0.12	p < 0.001	ENSMUST00000130078	zinc finger and BTB domain containing 38
Zc3h12a	1.03 ± 0.16	p < 0.001	1.28 ± 0.12	p < 0.001	1.60 ± 0.20	p < 0.001	NM_153159	zinc finger CCCH type containing 12A
Zc3h12c	0.57 ± 0.01	p < 0.001	0.83 ± 0.01	p < 0.001	1.97 ± 0.10	p < 0.001	NM_001162921	zinc finger CCCH type containing 12C
Zc3h6	-0.04 ± 0.21	1.000	0.36 ± 0.11	0.510	1.82 ± 0.11	p < 0.001	NM_178404	zinc finger CCCH type containing 6
Zc3hav1l	-0.29 ± 0.16	0.989	-0.31 ± 0.11	0.595	-1.43 ± 0.24	p < 0.001	NM_172467	zinc finger CCCH-type, antiviral 1-like
Zfand3	0.29 ± 0.08	0.063	0.47 ± 0.05	p < 0.001	1.12 ± 0.02	p < 0.001	NM_148926	zinc finger, AN1-type domain 3
Zfand4	-0.21 ± 0.17	1.000	-0.28 ± 0.12	0.616	-1.40 ± 0.47	p < 0.001	NM_001290338	zinc finger, AN1-type domain 4
Zfand5	-0.05 ± 0.04	1.000	0.12 ± 0.07	0.800	1.22 ± 0.05	p < 0.001	NM_009551	zinc finger, AN1-type domain 5
Zfp119a	0.27 ± 0.72	1.000	0.26 ± 0.89	0.905	1.52 ± 0.69	p < 0.001	NM_144546	zinc finger protein 119a
Zfp324	-0.10 ± 0.15	1.000	-0.25 ± 0.11	0.448	-1.00 ± 0.12	p < 0.001	NM_178732	zinc finger protein 324
Zfp326	-0.08 ± 0.13	1.000	-0.32 ± 0.13	0.091	-1.03 ± 0.15	p < 0.001	NM_018759	zinc finger protein 326
Zfp36	0.42 ± 0.20	0.004	0.41 ± 0.16	0.002	1.72 ± 0.22	p < 0.001	NM_011756	zinc finger protein 36
Zfp36l2	0.01 ± 0.08	1.000	-0.32 ± 0.05	0.019	-1.06 ± 0.09	p < 0.001	NM_001001806	zinc finger protein 36, C3H type-like 2
Zfp516	0.20 ± 0.14	1.000	0.59 ± 0.14	0.015	1.34 ± 0.20	p < 0.001	NM_001177464	zinc finger protein 516
Zfp574	-0.17 ± 0.23	1.000	-0.45 ± 0.06	0.353	-1.17 ± 0.22	p < 0.001	NM_175477	zinc finger protein 574
Zfp608	-0.11 ± 0.41	1.000	0.22 ± 0.19	0.885	1.03 ± 0.21	p < 0.001	NM_175751	zinc finger protein 608
Zfp617	-0.14 ± 0.08	1.000	0.06 ± 0.15	1.000	1.14 ± 0.21	p < 0.001	NM_133358	zinc finger protein 617
Zfp62	-0.03 ± 0.22	1.000	0.05 ± 0.22	1.000	1.02 ± 0.22	p < 0.001	NM_001024846	zinc finger protein 62
Zfp651	-0.29 ± 0.11	0.837	-0.55 ± 0.11	0.037	-1.34 ± 0.10	p < 0.001	NM_001166644	zinc finger protein 651
Zfp710	0.03 ± 0.10	1.000	-0.14 ± 0.11	0.875	-1.18 ± 0.07	p < 0.001	NM_001145999	zinc finger protein 710
Zfp719	-0.09 ± 0.25	1.000	-0.11 ± 0.21	1.000	1.26 ± 0.18	p < 0.001	NM_172482	zinc finger protein 719
Zfp772	-0.06 ± 0.30	1.000	-0.29 ± 0.31	0.790	-1.04 ± 0.41	0.001	NM_145577	zinc finger protein 772
Zfp811	-0.04 ± 0.38	1.000	0.24 ± 0.29	0.979	2.41 ± 0.28	p < 0.001	NM_001267583	zinc finger protein 811
Zfp862-ps	0.00 ± 0.23	1.000	-0.21 ± 0.20	0.883	-1.09 ± 0.35	p < 0.001	NR_015597	zinc finger protein 862, pseudogene
Zfp867	0.35 ± 0.26	1.000	0.41 ± 0.39	0.608	1.04 ± 0.32	0.001	NM_178417	zinc finger protein 867
Zfp874a	-0.01 ± 0.07	1.000	0.37 ± 0.12	0.779	1.10 ± 0.02	0.002	NM_177712	zinc finger protein 874a
Zfp935	0.05 ± 0.21	1.000	0.22 ± 0.30	0.893	1.18 ± 0.48	p < 0.001	NM_001136496	zinc finger protein 935
Zfp945	0.20 ± 0.19	1.000	0.22 ± 0.21	0.896	2.27 ± 0.09	p < 0.001	NM_001110254	zinc finger protein 945
Zfp952	0.01 ± 0.32	1.000	-0.14 ± 0.32	1.000	1.00 ± 0.32	p < 0.001	NM_001045559	zinc finger protein 952
Zfp955a	-0.17 ± 0.27	1.000	-0.04 ± 0.19	1.000	1.20 ± 0.50	p < 0.001	NM_029952	zinc finger protein 955A
Zfp964	0.19 ± 0.27	1.000	0.47 ± 0.09	0.552	2.18 ± 0.17	p < 0.001	NM_001177527	zinc finger protein 964
Zfyve1	0.16 ± 0.17	1.000	0.27 ± 0.22	0.561	1.09 ± 0.17	p < 0.001	NM_183154	zinc finger, FYVE domain containing 1
Zfyve16	-0.02 ± 0.18	1.000	0.26 ± 0.21	0.460	1.18 ± 0.17	p < 0.001	NM_173392	zinc finger, FYVE domain containing 16
Zmynd19	-0.05 ± 0.07	1.000	-0.20 ± 0.13	0.458	-1.49 ± 0.13	p < 0.001	ENSMUST00000028350	zinc finger, MYND domain containing 19
Znfx1	0.51 ± 0.05	0.041	1.13 ± 0.05	p < 0.001	0.55 ± 0.04	0.006	NM_001033196	zinc finger, NFX1-type containing 1

Zswim4	0.51	±	0.14	0.006	0.41	±	0.19	0.023	1.72	±	0.15	$p < 0.001$	NM_172503	zinc finger SWIM-type containing 4
Zwilch	-0.13	±	0.03	1.000	-0.42	±	0.07	0.001	-1.31	±	0.17	$p < 0.001$	NM_026507	zwilch kinetochore protein
