# Supplementary figures and images for: Global gene-expression profiles of intracellular survival of the BruAb2_1031 gene mutated Brucella abortus in professional phagocytes, RAW 264.7 cells
Source: BMC Microbiol. 2018 Jul 31;18:82. doi: 10.1186/s12866-018-1223-7 (PMC6069796; doi:10.1186/s12866-018-1223-7)

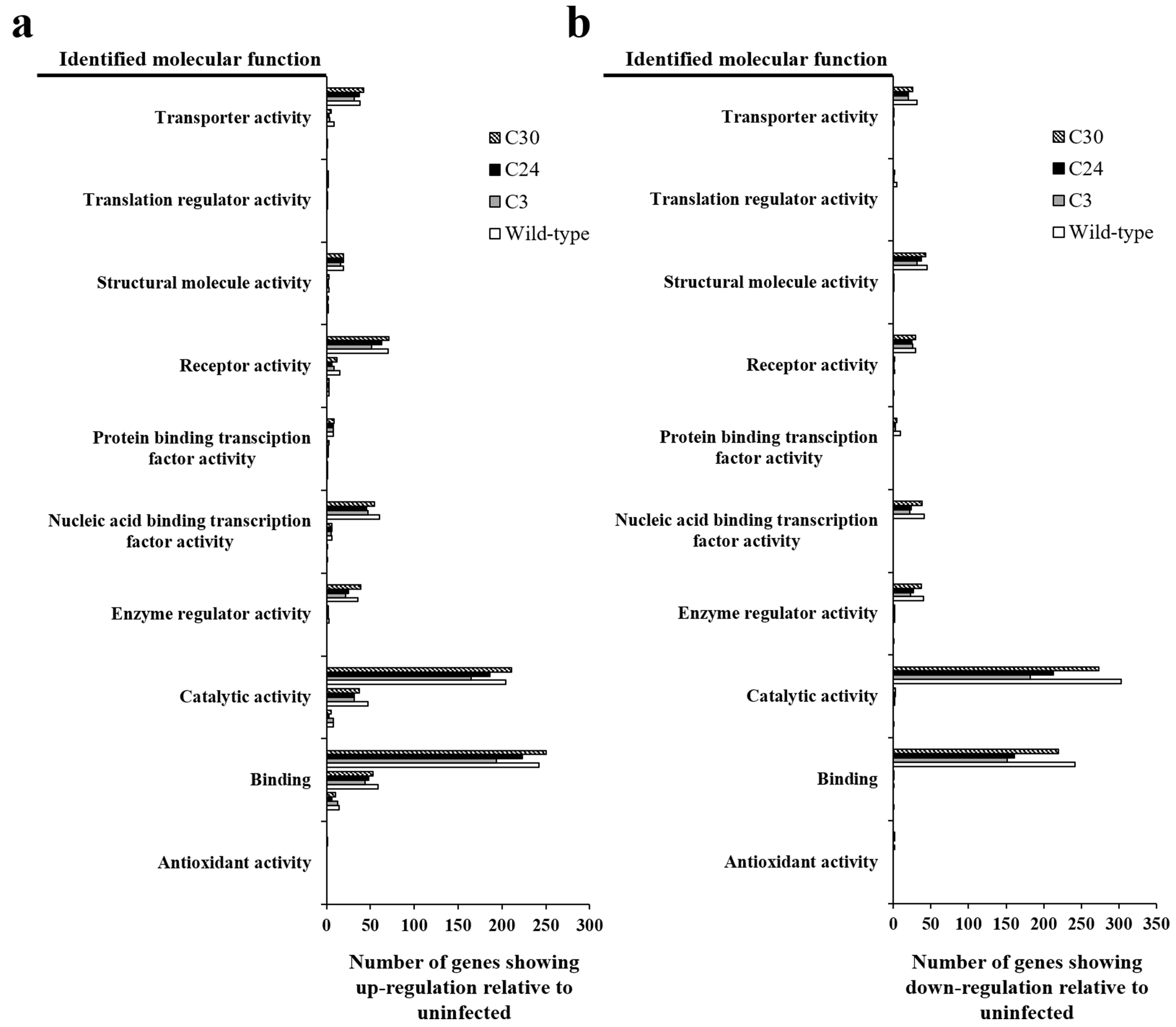

Supplement: Supplementary file 5 — Figure S3. Categorization by molecular function of genes showing different expression levels after infection. The different expression levels in B. abortus wild-type and mutant strain infected RAW 264.7 cells were compared to uninfected cells. (a) Up-regulated genes. (b) Down regulated genes. (TIF 922 kb) [file 12866_2018_1223_MOESM5_ESM.tif]

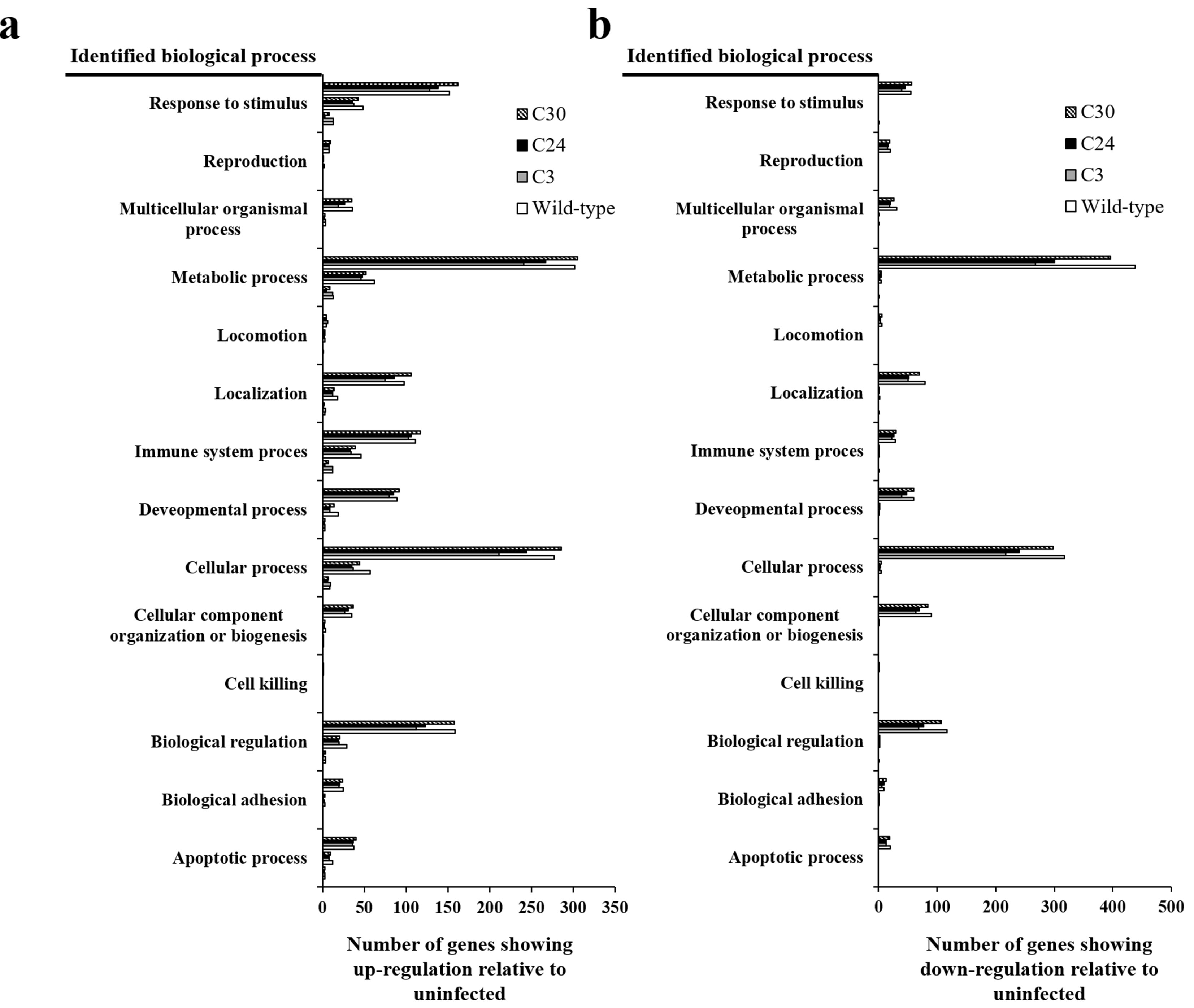

Supplement: Supplementary file 6 — Figure S4. Categorization by biological process of genes showing different expression levels after infection. The different expression levels in B. abortus wild-type and mutant strain infected RAW 264.7 cells were compared to uninfected cells. (a) Up-regulated genes. (b) Down regulated genes. (TIF 936 kb) [file 12866_2018_1223_MOESM6_ESM.tif]

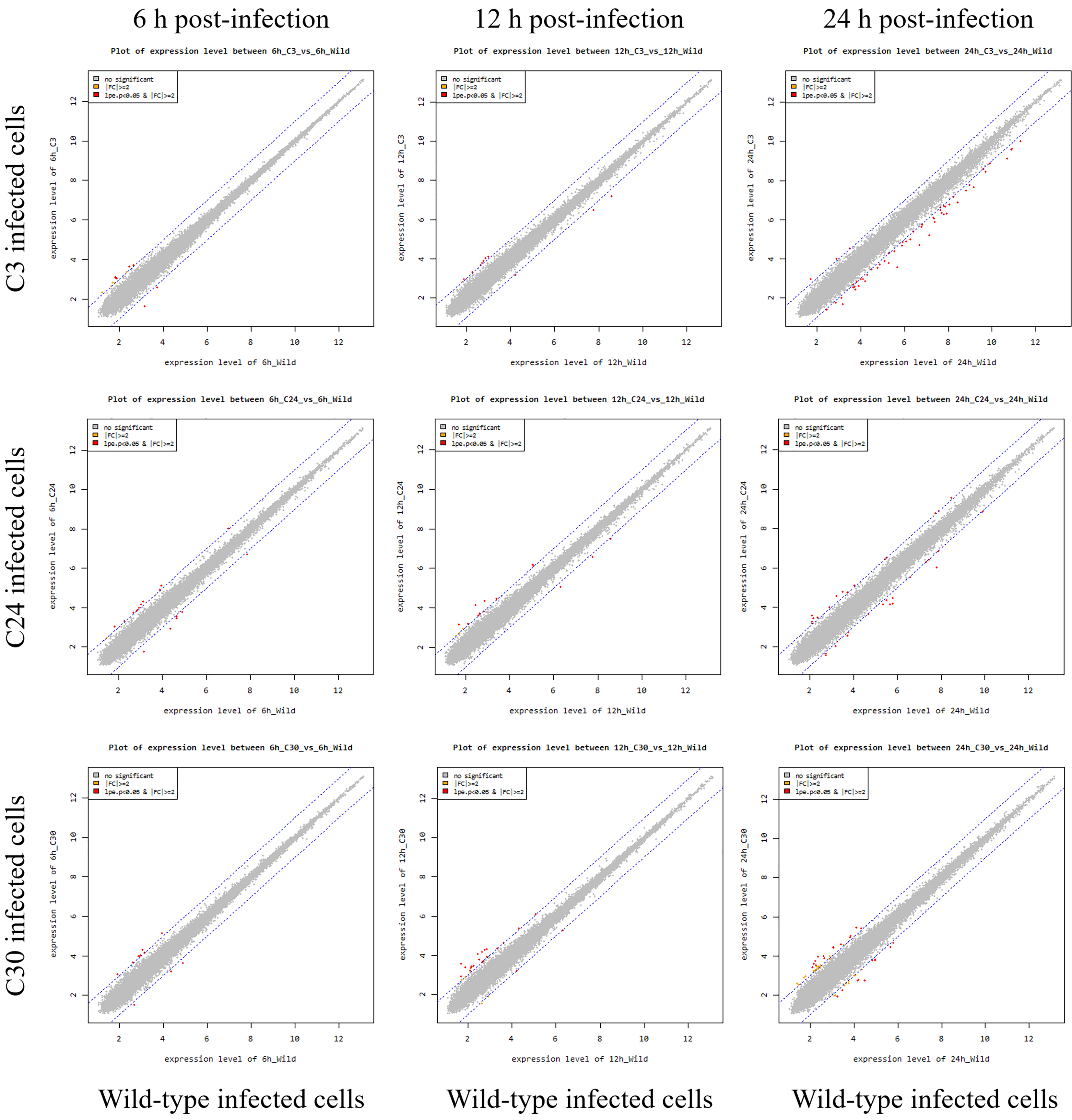

Supplement: Supplementary file 7 — Figure S5. Scatter plots showing different gene expressions. The different gene expression levels in B. abortus mutant strain infected RAW 264.7 cells were compared to cells infected with wild-type at 6 h, 12 h, and 24 h after infection. Genes showing different expression levels are indicated by red dots. (TIF 2217 kb) [file 12866_2018_1223_MOESM7_ESM.tif]
