# Supplementary material for: Global gene-expression profiles of intracellular survival of the BruAb2_1031 gene mutated Brucella abortus in professional phagocytes, RAW 264.7 cells
Source: BMC Microbiol. 2018 Jul 31;18:82. doi: 10.1186/s12866-018-1223-7 (PMC6069796; doi:10.1186/s12866-018-1223-7)
Supplement: Supplementary file 8 — Table S3. The genes showing altered expression in RAW 264.7 cells after C3 mutant strain infection. The different expression levels in B. abortus C3 mutant strain infected RAW 264.7 cells were compared to wild-type infected cells. (PDF 52 kb) [file 12866_2018_1223_MOESM8_ESM.pdf]

Additional Table 3. The genes showing altered expression in RAW 264.7 cells after C3 mutant strain infection. The different expression levels in *B. abortus* C3 mutant strain infected RAW 264.7 cells were compared to wild type infected cells.

Gene symbol	6 h		12 h		24 h		Gene accession No.	Gene description
	Fold changes (Log2 ratio)	*p* -value	Fold changes (Log2 ratio)	*p* -value	Fold changes (Log2 ratio)	*p* -value		
1700023H06Rik	-0.31 ± 0.45	1.000	-0.06 ± 0.12	1.000	-1.14 ± 0.17	0.007	ENSMUST00000161006	RIKEN cDNA 1700023H06 gene
4933432I03Rik	0.02 ± 0.33	1.000	0.06 ± 0.04	1.000	-1.19 ± 0.21	$p < 0.001$	NR_045657	RIKEN cDNA 4933432I03 gene
Adm	0.21 ± 0.16	1.000	-0.40 ± 0.20	0.003	-1.01 ± 0.19	$p < 0.001$	NM_009627	adrenomedullin
Atp13a4	0.16 ± 0.08	1.000	0.04 ± 0.30	1.000	-1.00 ± 0.24	$p < 0.001$	NM_001164612	ATPase type 13A4
Ccl2	0.01 ± 0.05	1.000	-0.66 ± 0.05	$p < 0.001$	-1.57 ± 0.09	$p < 0.001$	NM_011333	chemokine (C-C motif) ligand 2
Ccl5	-0.27 ± 0.43	1.000	-0.45 ± 0.22	0.008	-1.27 ± 0.13	$p < 0.001$	NM_013653	chemokine (C-C motif) ligand 5
Ccl7	-0.04 ± 0.23	1.000	-0.64 ± 0.12	0.003	-1.52 ± 0.21	$p < 0.001$	NM_013654	chemokine (C-C motif) ligand 7
Ccrl2	-0.06 ± 0.69	1.000	-0.51 ± 0.09	0.235	-1.29 ± 0.01	$p < 0.001$	NM_017466	chemokine (C-C motif) receptor-like 2
Cd83	-0.04 ± 0.11	1.000	-0.27 ± 0.07	1.000	-1.48 ± 0.05	$p < 0.001$	NM_001289915	CD83 antigen
Csf2	-0.07 ± 0.14	1.000	-0.21 ± 0.17	1.000	-1.33 ± 0.37	$p < 0.001$	NM_009969	colony stimulating factor 2 (granulocyte-macrophage)
Csf3	-0.13 ± 0.65	1.000	-0.99 ± 0.05	$p < 0.001$	-1.90 ± 0.20	$p < 0.001$	NM_009971	colony stimulating factor 3 (granulocyte)
Csrnp1	0.17 ± 0.12	1.000	-0.34 ± 0.07	0.836	-1.33 ± 0.11	$p < 0.001$	NM_153287	cysteine-serine-rich nuclear protein 1
Cxcl10	0.10 ± 0.22	1.000	0.02 ± 0.13	1.000	-1.34 ± 0.32	$p < 0.001$	NM_021274	chemokine (C-X-C motif) ligand 10
Cxcl11	0.18 ± 0.16	1.000	-0.32 ± 0.34	1.000	-1.29 ± 0.17	$p < 0.001$	NM_019494	chemokine (C-X-C motif) ligand 11
Cyp2d11	1.32 ± 0.25	0.008	0.11 ± 0.54	1.000	0.12 ± 0.40	1.000	NM_001104531	cytochrome P450, family 2, subfamily d, polypeptide 11
Dusp1	-0.05 ± 0.12	1.000	-0.24 ± 0.11	0.587	-1.01 ± 0.05	$p < 0.001$	NM_013642	dual specificity phosphatase 1
Fabp4	-0.07 ± 0.07	1.000	-0.47 ± 0.16	$p < 0.001$	-1.53 ± 0.18	$p < 0.001$	NM_024406	fatty acid binding protein 4, adipocyte
Fbxo15	0.04 ± 0.16	1.000	0.06 ± 0.32	1.000	-1.01 ± 0.24	0.004	NM_015798	F-box protein 15
Gadd45b	0.08 ± 0.08	1.000	-0.33 ± 0.08	0.042	-1.31 ± 0.02	$p < 0.001$	NM_008655	growth arrest and DNA-damage-inducible 45 beta
Gbp5	-0.29 ± 0.20	1.000	-0.11 ± 0.30	1.000	-1.14 ± 0.37	0.002	NM_153564	guanylate binding protein 5
Gm11517	-0.24 ± 0.32	1.000	0.22 ± 0.43	1.000	-1.30 ± 0.88	0.001	NR_033523	ubiquitin A-52 residue ribosomal protein fusion product 1 pseudogene
Gm12840	-0.26 ± 0.49	1.000	-0.24 ± 0.52	1.000	-1.21 ± 0.06	$p < 0.001$	ENSMUST00000156081	predicted gene 12840
Gm13822	-0.02 ± 0.14	1.000	0.11 ± 0.09	1.000	-1.07 ± 0.25	$p < 0.001$	ENSMUST00000127563	predicted gene 13822
Gm14060	0.02 ± 0.28	1.000	1.09 ± 1.37	0.010	0.70 ± 0.70	0.316	ENSMUST00000117747	predicted gene 14060
Gm22107	-1.14 ± 0.26	0.025	-0.34 ± 0.09	1.000	0.45 ± 0.68	0.716	ENSMUST00000122548	predicted gene, 22107
Gm22229	0.32 ± 0.72	1.000	0.02 ± 0.54	1.000	-1.01 ± 0.57	0.024	ENSMUST00000082987	predicted gene, 22229
Gm22327	0.44 ± 1.04	1.000	0.79 ± 1.04	0.236	-1.11 ± 0.49	0.010	ENSMUST00000158899	predicted gene, 22327
Gm22364	-0.45 ± 0.78	1.000	-0.38 ± 0.19	1.000	-1.03 ± 0.35	0.050	ENSMUST00000083097	predicted gene, 22364
Gm22645	0.25 ± 0.36	1.000	0.49 ± 0.36	1.000	-1.47 ± 0.46	$p < 0.001$	ENSMUST00000180010	predicted gene, 22645
Gm22805	1.17 ± 1.06	0.016	0.57 ± 0.62	0.569	0.03 ± 0.61	1.000	ENSMUST00000082739	predicted gene, 22805
Gm22867	-0.44 ± 0.55	1.000	-0.77 ± 0.93	0.369	1.25 ± 0.51	0.006	ENSMUST00000157499	predicted gene, 22867
Gm23294	-0.22 ± 0.30	1.000	0.15 ± 0.43	1.000	-1.14 ± 0.30	0.004	ENSMUST00000104150	predicted gene, 23294
Gm23432	0.02 ± 0.07	1.000	0.21 ± 0.28	1.000	-1.00 ± 0.36	0.028	ENSMUST00000157181	predicted gene, 23432
Gm23957	-1.54 ± 0.44	0.001	0.76 ± 0.46	0.471	-0.54 ± 0.25	0.683	ENSMUST00000157209	predicted gene, 23957
Gm24517	-0.22 ± 0.91	1.000	0.62 ± 0.88	0.603	-1.32 ± 1.18	0.001	ENSMUST00000122560	predicted gene, 24517
Gm24916	0.49 ± 0.49	1.000	0.08 ± 0.59	1.000	1.04 ± 0.35	0.010	ENSMUST00000157287	predicted gene, 24916
Gm25482	0.20 ± 0.35	1.000	1.04 ± 0.31	0.020	0.05 ± 0.80	1.000	ENSMUST00000083225	predicted gene, 25482

Gm26179	-0.50 ± 0.58	1.000	1.09 ± 0.86	0.032	-0.19 ± 0.61	1.000	ENSMUST00000177723	predicted gene, 26179
Gprc5a	0.06 ± 0.29	1.000	-0.43 ± 0.19	0.418	-1.06 ± 0.16	p < 0.001	NM_181444	G protein-coupled receptor, family C, group 5, member A
Hcar2	-0.21 ± 0.14	1.000	-0.61 ± 0.31	0.054	-1.28 ± 0.15	p < 0.001	NM_030701	hydroxycarboxylic acid receptor 2
Heatr9	0.15 ± 0.41	1.000	-0.31 ± 0.21	1.000	-1.20 ± 0.36	0.001	NM_001045543	HEAT repeat containing 9
Hist1h2bj	0.60 ± 0.59	0.758	1.17 ± 0.89	0.002	0.23 ± 0.43	1.000	ENSMUST00000110452	histone cluster 1, H2bj
Icosl	0.09 ± 0.09	1.000	-0.13 ± 0.08	1.000	-1.63 ± 0.07	p < 0.001	NM_015790	icos ligand
Id2	-0.02 ± 0.07	1.000	-0.36 ± 0.07	0.157	-1.13 ± 0.15	p < 0.001	NM_010496	inhibitor of DNA binding 2
Igkv1-122	1.23 ± 0.19	0.019	-0.50 ± 0.69	1.000	-0.03 ± 0.14	1.000	ENSMUST00000103314	immunoglobulin kappa chain variable 1-122
Il1a	0.13 ± 0.52	1.000	-0.29 ± 0.50	1.000	-1.97 ± 0.62	p < 0.001	NM_010554	interleukin 1 alpha
Il1b	0.41 ± 0.11	1.000	-0.01 ± 0.10	1.000	-2.09 ± 0.51	p < 0.001	NM_008361	interleukin 1 beta
Il6	0.00 ± 0.05	1.000	-0.22 ± 0.08	1.000	-1.06 ± 0.23	p < 0.001	NM_031168	interleukin 6
Irg1	-0.15 ± 0.08	1.000	-1.27 ± 0.09	p < 0.001	-1.30 ± 0.11	p < 0.001	NM_008392	immunoresponsive gene 1
LOC101055855	-0.20 ± 0.41	1.000	0.39 ± 0.27	1.000	-1.13 ± 0.67	0.006	XM_006508663	keratin-associated protein 5-5-like
Mcpt9	0.28 ± 0.31	1.000	1.06 ± 0.44	0.028	-0.21 ± 0.22	1.000	NM_010782	mast cell protease 9
Mir155	-0.09 ± 0.29	1.000	-0.06 ± 0.29	1.000	-1.13 ± 0.29	p < 0.001	NR_029565	microRNA 155
Mir466h	-0.03 ± 0.21	1.000	0.17 ± 0.24	1.000	-1.12 ± 0.51	0.001	NR_030570	microRNA 466h
Mir680-1	1.04 ± 0.13	0.044	0.46 ± 0.53	1.000	0.28 ± 0.39	1.000	NR_030447	microRNA 680-1
Nr1d1	0.08 ± 0.13	1.000	-0.28 ± 0.15	1.000	-1.53 ± 0.24	p < 0.001	NM_145434	nuclear receptor subfamily 1, group D, member 1
Nr4a3	0.11 ± 0.07	1.000	-0.27 ± 0.21	1.000	-1.21 ± 0.22	0.001	ENSMUST00000030025	nuclear receptor subfamily 4, group A, member 3
n-R5s183	-0.01 ± 0.47	1.000	-0.57 ± 0.16	0.758	1.09 ± 0.26	0.013	ENSMUST00000083939	nuclear encoded rRNA 5S 183
n-R5s204	-0.28 ± 0.92	1.000	1.12 ± 0.54	0.006	-0.40 ± 0.39	0.877	ENSMUST00000082972	nuclear encoded rRNA 5S 204
Olfr869	-0.47 ± 0.20	1.000	0.24 ± 0.54	1.000	-1.00 ± 0.42	0.014	NM_146557	olfactory receptor 869
Osbp2	-0.08 ± 0.32	1.000	-0.08 ± 0.20	1.000	-1.20 ± 0.32	p < 0.001	NM_152818	oxysterol binding protein 2
Phlda1	-0.16 ± 0.20	1.000	-0.26 ± 0.15	1.000	-1.07 ± 0.10	p < 0.001	NM_009344	pleckstrin homology-like domain, family A, member 1
Ptgs2	-0.23 ± 0.08	0.830	-1.40 ± 0.05	p < 0.001	-0.96 ± 0.06	p < 0.001	NM_011198	prostaglandin-endoperoxide synthase 2
Rasgef1b	-0.16 ± 0.09	1.000	-0.14 ± 0.11	1.000	-1.01 ± 0.08	p < 0.001	NM_145839	RasGEF domain family, member 1B
Rcan1	0.01 ± 0.11	1.000	0.04 ± 0.17	1.000	-1.17 ± 0.19	p < 0.001	NM_001081549	regulator of calcineurin 1
Rps26	0.19 ± 0.18	1.000	-0.04 ± 0.59	1.000	-1.10 ± 0.32	0.017	NM_013765	ribosomal protein S26
Slc39a2	0.03 ± 0.20	1.000	-0.41 ± 0.15	0.944	-1.08 ± 0.13	p < 0.001	NM_001039676	solute carrier family 39 (zinc transporter), member 2
Tnfrsf1b	-0.03 ± 0.22	1.000	-0.75 ± 0.25	p < 0.001	-1.25 ± 0.28	p < 0.001	NM_011610	tumor necrosis factor receptor superfamily, member 1b
Traf1	-0.18 ± 0.14	1.000	-0.23 ± 0.25	1.000	-1.20 ± 0.66	p < 0.001	NM_009421	TNF receptor-associated factor 1
Traj13	1.17 ± 0.31	0.032	-0.08 ± 0.50	1.000	-0.88 ± 0.56	0.093	ENSMUST00000103728	T cell receptor alpha joining 13
Trim30d	0.15 ± 0.25	1.000	0.19 ± 0.68	1.000	-1.05 ± 0.12	0.014	NM_001167828	tripartite motif-containing 30D
Vmn1r183	-0.22 ± 0.18	1.000	-1.02 ± 0.32	0.012	-0.05 ± 0.88	1.000	NM_203489	vomeronasal 1 receptor 183
Zfp811	0.36 ± 0.57	1.000	-0.44 ± 0.47	1.000	-1.50 ± 0.30	p < 0.001	NM_001267583	zinc finger protein 811
