# Supplementary material for: Global gene-expression profiles of intracellular survival of the BruAb2_1031 gene mutated Brucella abortus in professional phagocytes, RAW 264.7 cells
Source: BMC Microbiol. 2018 Jul 31;18:82. doi: 10.1186/s12866-018-1223-7 (PMC6069796; doi:10.1186/s12866-018-1223-7)
Supplement: Supplementary file 9 — Table S4. The genes showing altered expression in RAW 264.7 cells after C24 mutant strain infection. The different expression levels in B. abortus C24 mutant strain infected RAW 264.7 cells were compared to wild-type infected cells. (PDF 41 kb) [file 12866_2018_1223_MOESM9_ESM.pdf]

Additional Table 4. The genes showing altered expression in RAW 264.7 cells after C24 mutant strain infection. The different expression levels in *B. abortus* C24 mutant strain infected RAW 264.7 cells were compared to wild type infected cells.

Gene symbol	6 h		12 h		24 h		Gene accession No.	Gene description
	Fold changes (Log2 ratio)	*p* -value	Fold changes (Log2 ratio)	*p* -value	Fold changes (Log2 ratio)	*p* -value		
1700023H06Rik	-0.52 ± 0.44	0.924	-0.22 ± 0.10	1.000	-1.17 ± 0.10	0.012	ENSMUST00000161006	RIKEN cDNA 1700023H06 gene
2700038G22Rik	-0.07 ± 0.23	1.000	-0.07 ± 0.29	1.000	1.02 ± 0.09	0.012	NR_045040	RIKEN cDNA 2700038G22 gene
4921509C19Rik	0.04 ± 0.91	1.000	1.01 ± 0.40	0.030	-0.21 ± 0.69	1.000	NM_198655	RIKEN cDNA 4921509C19 gene
BC117090	1.14 ± 0.40	0.004	-0.09 ± 0.90	1.000	-0.07 ± 0.06	1.000	NM_001001332	cDNA sequence BC1179090
Cebpd	-0.01 ± 0.12	1.000	-0.04 ± 0.41	1.000	1.02 ± 0.35	$p < 0.001$	NM_007679	CCAAT/enhancer binding protein (C/EBP), delta
Dusp1	-0.36 ± 0.10	0.132	-0.35 ± 0.13	0.083	-1.03 ± 0.01	$p < 0.001$	NM_013642	dual specificity phosphatase 1
Gm10721	0.42 ± 0.43	0.661	0.04 ± 0.19	1.000	-1.33 ± 0.44	$p < 0.001$	ENSMUST00000143083	predicted gene 10721
Gm11084	1.05 ± 1.45	0.004	0.05 ± 0.35	1.000	1.36 ± 0.06	$p < 0.001$	ENSMUST00000112062	predicted gene 11084
Gm11517	-0.49 ± 0.33	1.000	0.62 ± 0.09	0.851	-1.04 ± 0.96	0.036	NR_033523	ubiquitin A-52 residue ribosomal protein fusion product 1 pseudogene
Gm22645	-0.05 ± 0.36	1.000	0.06 ± 0.48	1.000	-1.16 ± 0.46	0.021	ENSMUST00000180010	predicted gene, 22645
Gm23326	1.05 ± 0.38	0.033	-0.36 ± 0.64	1.000	0.17 ± 0.02	1.000	ENSMUST00000157419	predicted gene, 23326
Gm23487	0.10 ± 0.17	1.000	0.12 ± 0.55	1.000	-1.15 ± 0.83	$p < 0.001$	ENSMUST00000104819	predicted gene, 23487
Gm23614	0.05 ± 0.05	1.000	0.11 ± 0.54	1.000	1.47 ± 0.62	0.001	ENSMUST00000082598	predicted gene, 23614
Gm23905	1.04 ± 0.43	0.017	0.26 ± 0.27	1.000	0.45 ± 0.18	0.732	ENSMUST00000157568	predicted gene, 23905
Gm23947	0.25 ± 0.32	1.000	1.09 ± 1.08	0.042	-0.13 ± 1.03	1.000	ENSMUST00000093636	predicted gene, 23947
Gm23957	-1.41 ± 0.44	0.001	0.69 ± 0.49	0.685	0.29 ± 0.38	1.000	ENSMUST00000157209	predicted gene, 23957
Gm24089	1.05 ± 0.29	$p < 0.001$	0.04 ± 0.20	1.000	0.70 ± 0.20	$p < 0.001$	ENSMUST00000093696	predicted gene, 24089
Gm24263	-0.21 ± 0.01	1.000	0.08 ± 0.39	1.000	1.08 ± 0.23	0.039	ENSMUST00000104059	predicted gene, 24263
Gm24916	0.54 ± 0.29	0.534	0.53 ± 0.17	0.567	1.28 ± 0.46	$p < 0.001$	ENSMUST00000157287	predicted gene, 24916
Gm24960	0.11 ± 0.30	1.000	1.02 ± 0.30	0.106	1.27 ± 0.90	0.006	ENSMUST00000157923	predicted gene, 24960
Gm25401	-1.06 ± 0.80	0.005	0.13 ± 0.27	1.000	0.30 ± 0.35	1.000	ENSMUST00000082975	predicted gene, 25401
Gm25482	0.37 ± 0.46	1.000	1.09 ± 0.29	0.016	-0.33 ± 1.10	1.000	ENSMUST00000083225	predicted gene, 25482
Gm25568	-1.42 ± 0.43	$p < 0.001$	0.16 ± 0.28	1.000	-0.34 ± 0.77	0.969	ENSMUST00000104668	predicted gene, 25568
Gm25909	0.25 ± 0.82	1.000	1.04 ± 0.75	0.033	-0.51 ± 0.23	0.652	ENSMUST00000157619	predicted gene, 25909
Gpr84	-0.49 ± 0.06	$p < 0.001$	-0.08 ± 0.02	1.000	1.11 ± 0.22	$p < 0.001$	NM_030720	G protein-coupled receptor 84
Hist1h2bj	0.47 ± 0.64	0.913	1.48 ± 0.68	$p < 0.001$	0.65 ± 0.62	0.455	ENSMUST00000110452	histone cluster 1, H2bj
Hist2h3c2	-0.24 ± 0.14	1.000	1.03 ± 0.07	$p < 0.001$	-0.41 ± 0.51	0.773	ENSMUST00000167403	histone cluster 2, H3c2
Hnrnpu	0.20 ± 0.25	0.944	0.60 ± 0.15	$p < 0.001$	1.02 ± 0.04	$p < 0.001$	XR_373612	heterogeneous nuclear ribonucleoprotein U
Ifi204	-1.14 ± 0.19	0.001	-0.53 ± 0.18	0.015	-0.22 ± 0.25	0.684	NM_008329	interferon activated gene 204
Ighj2	1.02 ± 0.18	0.023	0.57 ± 0.33	0.698	0.54 ± 0.70	0.727	ENSMUST00000103429	immunoglobulin heavy joining 2
Igkv1-122	1.20 ± 0.44	0.011	0.29 ± 0.43	1.000	-0.40 ± 0.28	0.982	ENSMUST00000103314	immunoglobulin kappa chain variable 1-122
Il1b	0.19 ± 0.18	1.000	-0.10 ± 0.46	1.000	-1.51 ± 0.54	$p < 0.001$	NM_008361	interleukin 1 beta
Irg1	-0.96 ± 0.02	0.001	-1.19 ± 0.15	$p < 0.001$	0.02 ± 0.07	1.000	NM_008392	immunoresponsive gene 1
Mamdc2	-0.10 ± 0.34	1.000	0.06 ± 0.10	1.000	1.04 ± 0.10	0.005	NM_174857	MAM domain containing 2
Mir186	-0.60 ± 0.47	0.594	0.37 ± 0.57	1.000	1.11 ± 0.41	0.025	NR_029572	microRNA 186
Mir328	1.17 ± 1.10	$p < 0.001$	0.19 ± 0.32	1.000	-0.32 ± 0.28	0.968	NR_029761	microRNA 328

Nr1d1	-0.06 ± 0.16	1.000	-0.23 ± 0.19	1.000	-1.78 ± 0.20	p < 0.001	NM_145434	nuclear receptor subfamily 1, group D, member 1
n-R5s127	1.01 ± 0.22	0.025	0.21 ± 0.57	1.000	0.92 ± 0.88	0.051	ENSMUST00000179185	nuclear encoded rRNA 5S 127
n-R5s183	-0.14 ± 0.42	1.000	-0.51 ± 0.29	0.981	1.11 ± 0.37	0.014	ENSMUST00000083939	nuclear encoded rRNA 5S 183
n-R5s56	1.16 ± 0.52	0.002	-0.41 ± 0.27	1.000	0.11 ± 0.15	1.000	ENSMUST00000082521	nuclear encoded rRNA 5S 56
Olfr787	0.11 ± 0.10	1.000	-0.01 ± 0.44	1.000	1.17 ± 0.51	0.017	NM_001011822	olfactory receptor 787
Osbp2	-0.08 ± 0.33	1.000	-0.17 ± 0.14	1.000	-1.01 ± 0.41	p < 0.001	NM_152818	oxysterol binding protein 2
Ptgs2	-0.76 ± 0.11	p < 0.001	-1.11 ± 0.15	p < 0.001	-0.06 ± 0.02	1.000	NM_011198	prostaglandin-endoperoxide synthase 2
Pyhin1	-0.61 ± 0.10	0.457	-1.25 ± 0.07	p < 0.001	-0.32 ± 0.22	0.344	NM_175026	pyrin and HIN domain family, member 1
Rcan1	-0.05 ± 0.12	1.000	0.10 ± 0.10	1.000	-1.02 ± 0.16	p < 0.001	NM_001081549	regulator of calcineurin 1
Rhob	-0.55 ± 0.30	0.186	-0.34 ± 0.05	0.930	-1.03 ± 0.28	p < 0.001	NM_007483	ras homolog gene family, member B
Rps15	-1.19 ± 1.28	0.001	0.41 ± 1.04	1.000	0.42 ± 1.11	0.849	ENSMUST00000062674	ribosomal protein S15
Snora73a	0.67 ± 0.10	p < 0.001	0.25 ± 0.06	0.212	1.05 ± 0.10	p < 0.001	NR_028512	small nucleolar RNA, H/ACA box 73a
Snora73b	0.60 ± 0.12	p < 0.001	0.12 ± 0.14	1.000	1.00 ± 0.23	p < 0.001	NR_028513	small nucleolar RNA, H/ACA box 73b
Traj58	1.06 ± 0.32	0.019	-0.59 ± 0.16	0.741	0.47 ± 0.31	0.840	ENSMUST00000103687	T cell receptor alpha joining 58
