# Supplementary material for: Global gene-expression profiles of intracellular survival of the BruAb2_1031 gene mutated Brucella abortus in professional phagocytes, RAW 264.7 cells
Source: BMC Microbiol. 2018 Jul 31;18:82. doi: 10.1186/s12866-018-1223-7 (PMC6069796; doi:10.1186/s12866-018-1223-7)
Supplement: Supplementary file 10 — Table S5. The genes showing altered expression in RAW 264.7 cells after C30 mutant strain infection. The different expression levels in B. abortus C30 mutant strain infected RAW 264.7 cells were compared to wild-type infected cells. (PDF 37 kb) [file 12866_2018_1223_MOESM10_ESM.pdf]

Additional Table 5. The genes showing altered expression in RAW 264.7 cells after C30 mutant strain infection. The different expression levels in *B. abortus* C30 mutant strain infected RAW 264.7 cells were compared to wild type infected cells.

Gene symbol	6 h		12 h		24 h		Gene accession No.	Gene description
	Fold changes (Log2 ratio)	*p* -value	Fold changes (Log2 ratio)	*p* -value	Fold changes (Log2 ratio)	*p* -value		
Ap3s1-ps2	-0.44 ± 1.14	1.000	-0.83 ± 0.53	0.107	-1.16 ± 0.37	0.012	ENSMUST00000178107	adaptor-related protein complex 3, sigma 1 subunit, pseudogene 2
Apol7e	-0.28 ± 0.94	1.000	1.02 ± 0.75	0.017	-0.84 ± 0.42	0.281	NM_001134802	apolipoprotein L 7e
Gm22013	0.34 ± 0.11	1.000	1.22 ± 0.47	0.007	-0.07 ± 0.21	1.000	ENSMUST00000158788	predicted gene, 22013
Gm22069	-0.05 ± 0.49	1.000	1.52 ± 0.96	$p < 0.001$	0.97 ± 0.72	0.179	ENSMUST00000179911	predicted gene, 22069
Gm22265	-0.23 ± 0.49	1.000	-0.08 ± 0.27	1.000	-1.74 ± 0.45	$p < 0.001$	ENSMUST00000082947	predicted gene, 22265
Gm22503	0.55 ± 0.94	1.000	0.95 ± 0.07	0.174	1.25 ± 0.92	0.019	ENSMUST00000158983	predicted gene, 22503
Gm22749	-0.03 ± 0.35	1.000	-0.05 ± 0.18	1.000	-1.44 ± 0.60	0.003	ENSMUST00000082490	predicted gene, 22749
Gm23320	0.59 ± 0.59	0.952	1.28 ± 0.81	0.004	-0.04 ± 0.67	1.000	ENSMUST00000104462	predicted gene, 23320
Gm23322	0.08 ± 0.16	1.000	-0.16 ± 0.16	1.000	1.26 ± 0.29	0.024	ENSMUST00000104464	predicted gene, 23322
Gm23579	-0.05 ± 0.41	1.000	0.69 ± 1.08	0.261	-1.17 ± 0.44	0.010	ENSMUST00000158528	predicted gene, 23579
Gm23730	-0.66 ± 0.55	0.134	0.01 ± 0.35	1.000	-1.20 ± 0.26	0.001	ENSMUST00000083236	predicted gene, 23730
Gm23905	1.06 ± 0.48	0.015	0.13 ± 0.24	1.000	1.13 ± 0.16	0.013	ENSMUST00000157568	predicted gene, 23905
Gm24142	0.58 ± 0.66	0.895	1.01 ± 0.40	0.042	1.36 ± 0.28	0.005	ENSMUST00000101960	predicted gene, 24142
Gm24263	-0.16 ± 0.39	1.000	0.30 ± 0.38	1.000	1.32 ± 0.80	0.017	ENSMUST00000104059	predicted gene, 24263
Gm24292	-1.14 ± 0.46	0.035	0.44 ± 0.30	1.000	-0.66 ± 0.45	0.968	ENSMUST00000082888	predicted gene, 24292
Gm24391	0.17 ± 0.16	1.000	1.12 ± 0.40	0.026	0.39 ± 0.33	1.000	ENSMUST00000158138	predicted gene, 24391
Gm24573	0.38 ± 0.23	1.000	-0.04 ± 1.01	1.000	1.50 ± 0.60	0.002	ENSMUST00000175499	predicted gene, 24573
Gm24575	0.37 ± 0.51	1.000	0.01 ± 0.51	1.000	1.35 ± 0.90	0.003	ENSMUST00000175502	predicted gene, 24575
Gm24783	0.03 ± 0.04	1.000	1.08 ± 0.15	0.015	0.86 ± 0.42	0.277	ENSMUST00000157639	predicted gene, 24783
Gm24817	0.17 ± 0.73	1.000	1.27 ± 0.23	0.010	0.65 ± 0.56	0.911	ENSMUST00000093672	predicted gene, 24817
Gm24955	0.33 ± 0.18	1.000	-0.55 ± 0.43	0.617	-1.05 ± 0.61	0.040	ENSMUST00000178059	predicted gene, 24955
Gm24960	0.56 ± 0.35	1.000	1.03 ± 0.84	0.092	1.41 ± 0.56	0.005	ENSMUST00000157923	predicted gene, 24960
Gm25179	0.39 ± 1.08	1.000	1.30 ± 0.73	0.002	0.42 ± 0.71	1.000	ENSMUST00000122632	predicted gene, 25179
Gm25482	0.52 ± 0.36	0.812	1.55 ± 0.41	$p < 0.001$	-0.15 ± 0.73	1.000	ENSMUST00000083225	predicted gene, 25482
Gm25568	-1.14 ± 0.67	0.005	0.26 ± 0.10	1.000	-0.37 ± 1.47	1.000	ENSMUST00000104668	predicted gene, 25568
Gm25792	1.04 ± 0.94	0.030	0.16 ± 0.29	1.000	0.91 ± 0.22	0.100	ENSMUST00000083289	predicted gene, 25792
Gm26002	1.12 ± 0.73	0.008	0.55 ± 0.44	0.807	0.20 ± 0.68	1.000	ENSMUST00000101915	predicted gene, 26002
Hist1h2bj	0.38 ± 0.60	1.000	1.45 ± 0.74	$p < 0.001$	0.33 ± 0.73	1.000	ENSMUST00000110452	histone cluster 1, H2bj
Hist1h2bm	0.05 ± 0.13	1.000	1.01 ± 0.17	0.011	-0.11 ± 0.58	1.000	BC139381	histone cluster 1, H2bm
Hist2h3c2	-0.02 ± 0.18	1.000	1.02 ± 0.21	$p < 0.001$	-0.29 ± 0.43	1.000	ENSMUST00000167403	histone cluster 2, H3c2
Ifi204	-1.26 ± 0.36	$p < 0.001$	-0.30 ± 0.23	0.656	-0.63 ± 0.22	0.027	NM_008329	interferon activated gene 204
Ighv1-4	-0.37 ± 0.31	1.000	0.01 ± 0.76	1.000	-1.23 ± 0.27	0.046	ENSMUST00000103493	immunoglobulin heavy variable 1-4
Klk1b27	1.17 ± 0.22	0.024	0.50 ± 0.47	1.000	-0.18 ± 0.57	1.000	NM_020268	kallikrein 1-related peptidase b27
Mir186	-0.07 ± 0.12	1.000	0.83 ± 0.59	0.186	1.58 ± 0.89	$p < 0.001$	NR_029572	microRNA 186
Mir328	1.19 ± 1.14	$p < 0.001$	0.16 ± 0.32	1.000	0.48 ± 0.24	1.000	NR_029761	microRNA 328

n-R5s204	-0.36 ± 0.90	1.000	1.53 ± 0.40	p < 0.001	-0.42 ± 0.60	1.000	ENSMUST00000082972	nuclear encoded rRNA 5S 204
n-R5s54	-0.02 ± 0.57	1.000	1.05 ± 0.22	0.001	-0.52 ± 0.78	0.960	ENSMUST00000083837	nuclear encoded rRNA 5S 54
n-R5s80	0.22 ± 0.63	1.000	0.34 ± 0.30	1.000	1.16 ± 0.23	0.008	ENSMUST00000122596	nuclear encoded rRNA 5S 80
Pyhin1	-0.70 ± 0.09	0.285	-1.03 ± 0.37	p < 0.001	-0.76 ± 0.57	0.007	NM_175026	pyrin and HIN domain family, member 1
Snord43	-0.30 ± 0.24	0.986	0.09 ± 0.18	1.000	1.09 ± 0.14	0.009	NR_028281	small nucleolar RNA, C/D box 43
Sult2a3	1.03 ± 0.76	0.021	-0.19 ± 0.54	1.000	-0.30 ± 0.63	1.000	ENSMUST00000098809	sulfotransferase family 2A, dehydroepiandrosterone (DHEA)-preferring, member 3
Tgtp2	-0.52 ± 0.57	1.000	-0.57 ± 0.19	0.969	-1.34 ± 0.72	0.024	ENSMUST00000128411	T cell specific GTPase 2//T cell specific GTPase 1
Trbj1-7	0.34 ± 0.37	1.000	1.24 ± 0.45	0.007	0.96 ± 1.09	0.187	ENSMUST00000103290	T cell receptor beta joining 1-7
Vmn1r183	-0.18 ± 0.27	1.000	-1.02 ± 0.45	0.022	-0.34 ± 0.67	1.000	NM_203489	vomeronasal 1 receptor 183
Zfp944	1.02 ± 0.17	0.019	-0.23 ± 0.20	1.000	-0.35 ± 0.49	1.000	NM_176962	zinc finger protein 944
